# NADPH oxidases in immunometabolism and disease pathology: mechanistic networks, pollutant triggers, and therapeutic frontiers

**DOI:** 10.1038/s41423-026-01447-2

**Published:** 2026-08-24

**Authors:** M. Thakur, D. Mutyala, A. A. Amoliga, M. Contin Ortega, S. Batra

**Affiliations:** 1 Laboratory of Pulmonary Immunotoxicology, Department of Environmental Toxicology, College of Agricultural, Human, and Environmental Sciences, Southern University and A&M College, Baton Rouge, Louisiana USA; 2 Southern University Agricultural Research and Extension Center, Southern University System, Baton Rouge, Louisiana USA

**Keywords:** NADPH oxidases, Redox signaling, Immunometabolism, Environmental exposure, NOX inhibitors, Inflammatory diseases, Predictive markers

## Abstract

NADPH oxidases (NOXs) have emerged as central hubs that link environmental, metabolic, and immune cues through spatially organized redox signaling. However, their roles across tissues and disease states have not been comprehensively evaluated in an integrated manner. This review integrates recent advances in structural biology, immunometabolism, toxicology, and systems biology to provide an updated, comprehensive, and accessible view of NOX biology. Recent advances in high‑resolution cryo-EM, AlphaFold‑based modeling and molecular dynamics studies have provided new insights into NOX architecture, catalytic sites, post‑translational modifications and regulatory mechanisms, and docking interfaces for RAC1 and p47^*phox*^. Emerging evidence further indicates that cellular NOX-derived ROS can reprogram macrophage and T-cell metabolism, stabilize HIF-1α, and tune the balance between effector and regulatory states, thereby linking NOX activity to checkpoint control and tumor immune escape. A second focus is on how real‑world pollutants converge on NOX isoforms as proximal mediators of redox signaling across lung, vascular, hepatic, renal, and neural tissues. NOX activation during cellular injury may contribute to oxidative stress, mitochondrial dysfunction, inflammasome activation, and fibrotic signaling, through extracellular vesicles, lipid rafts, and noncoding RNAs. Finally, the review evaluates emerging therapeutic strategies, including isoform-selective/pan-NOX/peptide inhibitors, and nanozymes. It also discusses emerging approaches such as exosome-based biomarkers, network pharmacology, and machine learning for patient stratification and pharmacodynamic monitoring. By highlighting key mechanistic gaps and translational opportunities, this review establishes NOXs as actionable nodal regulators at the intersection of immunity, metabolism, environmental exposure, and human disease.

## Introduction

Nicotinamide adenine dinucleotide phosphate oxidases (NADPH oxidases), or NOX enzymes, are membrane-associated proteins that are best known for producing reactive oxygen species (ROS) in a well-regulated, signal-dependent manner [1]. Unlike mitochondria, where ROS are generated as byproducts of oxidative metabolism, NOX enzymes are designed to deliberately generate ROS. These molecules act as antimicrobial agents and signaling molecules, depending on the context. Foundational evidence came from classic immunology studies conducted in 1973 by Babior and colleagues, who reported that stimulated neutrophils generate bursts of superoxide (O_2_^•−^) to kill pathogens. These findings established NOX2 activity (phagocyte NADPH oxidase) as a cornerstone of the innate immune response [2]. For many years, the antimicrobial role dominated the understanding of NADPH oxidase. We reviewed the early history of NADPH oxidase in our 2015 article, summarizing how NOX2 activity in phagocytes shaped the understanding of innate immunity [3]. By the time we compiled a 2022 review, this view had expanded to include nonphagocytic functions. NOX enzymes are no longer viewed as being confined to innate immunity but rather as broad regulators of redox biology, with roles in vascular remodeling, fibrosis, metabolism, and cancer progression [4]. We now know that the NOX family comprises five isoforms (NOX1 through NOX5) and the two dual oxidases DUOX1 and DUOX2. Each isoform differs in activation mechanisms, partner proteins, and tissue-specific functions [1]. This diversity allows NOX enzymes to act as modular redox hubs that respond to cytokines, growth factors, metabolic stress, and environmental exposures. A defining feature of NOX signaling is its subcellular specificity and context-dependent activation, which may be transient or sustained depending on the isoform. Unlike mitochondrial ROS, NOX-generated ROS are produced at highly localized subcellular sites, facilitating localized redox signaling. In endothelial cells, this spatiotemporal control allows NOX-derived ROS to regulate angiogenesis, permeability, and inflammatory signaling [5–8]. When tightly regulated, NADPH oxidase-derived ROS supports normal physiological processes, whereas dysregulation contributes to pathological states. In recent years, structural biology has transformed the field of NOX biology. High-resolution cryo-EM studies have substantially advanced our understanding of NOX2 biology by revealing the architecture of the human NOX2-p22^*phox*^ complex in the resting state [9, 10]. Further cryo-EM studies have resolved the structure of the essential for reactive oxygen species (EROS) protein, which associates with NOX2 on the side opposite p22^*phox*^, at near-atomic resolution [10]. These findings provide new insights into the role of EROS in NOX2 maturation and stabilization, processes that are critical for efficient expression of NOX2. These new structures deepen the mechanistic understanding of the assembly and regulation of NOX2-based enzyme complexes, even though the precise activation mechanism involving cytosolic factors remains incompletely resolved [10, 11]. These structural and mechanistic insights into NADPH oxidase biology have enabled the rational design of isoform-selective inhibitors, representing a shift from nonspecific antioxidant therapies. Many conventional antioxidants have shown limited clinical efficacy because they quench both harmful and physiologically essential ROS, whereas isoform-selective NOX inhibitors target the specific enzyme forms underlying pathological ROS signaling in diseases such as fibrosis and hypertension. One of the most advanced compounds, Setanaxib (GKT137831), inhibits NOX1 and NOX4 and has shown promise in fibrotic models, with ongoing clinical evaluation in idiopathic pulmonary fibrosis and other diseases [12]. Its selectivity and favorable pharmacokinetic profile distinguish it from conventional antioxidant approaches and reflect a new strategy for selectively modulating disease-associated ROS rather than broadly suppressing redox signaling. The dual roles of NOX enzymes are now well established. When properly regulated, their activity is protective and essential; when excessive or mislocalized, they contribute to chronic inflammation, cancer, neurodegeneration, and metabolic disorders [13, 14]. For example, NOX4 can remodel the tumor microenvironment in breast cancer [15], whereas NOX1 has been implicated in colitis-associated inflammation [16]. Taken together, these findings establish NADPH oxidases not only as sources of oxidative stress but also as versatile redox signaling hubs that regulate cellular physiology and signal transduction. Building on our earlier reviews [3, 4], this review integrates recent advances in structural biology, immunometabolism, toxicology, and systems biology to provide an updated perspective on NOX-dependent signaling pathways in health and disease.

### Discovery and functional scope of NOX enzymes

The origin of NADPH oxidase research can be traced back to Otto Warburg’s classic work in 1908, in which he reported a six- to sevenfold increase in oxygen consumption in sea urchin eggs immediately after fertilization [17]. These observations provided the early evidence that cells can rapidly upregulate ROS-generating oxidative processes, paving the way for subsequent discoveries in the regulation of ROS generation. The modern era of NOX research began with the recognition of the phagocyte respiratory burst, a process in which NADPH-dependent electron transfer drives superoxide in neutrophils to eliminate invading microbes [18]. Foundational work by Karnovsky, along with the landmark study by Babior in 1973, conclusively established the mechanisms underlying NADPH oxidase-mediated superoxide generation in phagocytes [2, 19]. As summarized in our 2015 review, these early findings established NOX enzymes as central to innate immune defense [3]. The central role of NOX2 was confirmed when genetic defects were linked to chronic granulomatous disease (CGD), a rare immunodeficiency characterized by defective microbial killing [20]. Subsequent expansion in the field revealed the existence of additional isoforms, each characterized by distinct tissue distributions, regulatory partners, and functional outputs. Recent studies in experimental models of lung injury have shown that endothelial cells express multiple NOX isoforms, linking NOX activity to endothelial ROS signaling and vascular dysfunction [21–23]. For instance, NOX4 has been implicated in vascular remodeling and the pathogenesis of diabetic vascular disease [24], as well as in wound healing and fibroblast function [25, 26]. Building on its diverse roles in vascular biology, a recent pancancer analysis highlighted NOX4 as a promising biomarker associated with proliferation and metastasis [27]. Collectively, these studies show that NOX enzymes function far beyond their original characterization as phagocyte oxidases and are now recognized as versatile redox regulators in both health and disease.

### Evolution: from phagocytic ROS to multiorgan signaling

Comparative genomic and evolutionary analyses suggest that NOX enzymes form an ancient family tailored to regulate redox adaptation across diverse life forms. A pivotal study by Kawahara and coworkers used comparative genomics to demonstrate that NOX2 isoforms are conserved across vertebrates; moreover, members of the NOX/DUOX family trace their evolutionary origins to ancestral metazoan lineages, indicating that their origins predate the diversification of modern animal taxa [28]. Subsequent diversification of NOX enzymes paralleled the emergence of complex immune and vascular systems. Specialized regulatory domains, including EF-hand calcium-binding motifs in NOX5 and peroxidase-like domains in DUOX family members, enable fine control of ROS generation in response to calcium or hydrogen peroxide signaling [29, 30]. For example, NOX1, which is predominantly expressed in the gut epithelium, contributes to epithelial homeostasis and the regulation of ROS signaling, which is involved in cellular proliferation, differentiation, and barrier integrity [31]. The heme peroxidase-like domains of DUOX1 and DUOX2 catalyze the production of H_2_O_2_, which supports mucosal defense. In contrast, NOX5 contains EF-hand calcium-binding motifs that couple intracellular calcium signals to generate ROS, integrating calcium-dependent control of redox signaling [30]. NOX enzymes also play vital roles in stress adaptation and developmental signaling, as shown in invertebrates and plants, highlighting the evolutionary conservation of redox signaling. Collectively, these observations indicate that the NOX family has evolved from primitive host defense oxidases into multi-functional regulators of redox signaling.

### Isoform summary and subunit composition

Each NOX enzyme operates as part of a multisubunit complex rather than in isolation. Enzymatic activity depends on precise assembly with specific membrane-bound and cytosolic partners that are unique to each isoform. The coordinated assembly of these components is essential for the tight regulation of ROS generation, as clearly detailed in previous work [3, 4]. NOX serves as the membrane-bound core engine that generates ROS, while Neutrophil Cytosolic Factor (NCF)/*phox* components act as the cytosolic regulatory components that dock onto and activate it. Recent advances in cryo-EM have provided insights into the organization of the NOX core complex, revealing how this architecture facilitates the recruitment and activation of essential cytosolic regulatory partners [9, 11]. In neutrophils, phosphorylation of p47^*phox*^ is essential for the assembly of the NADPH oxidase complex. Upon activation, p47^*phox*^ [Neutrophil Cytosolic Factor 1 (NCF1)] interacts with p67^*phox*^(NCF2), p40^*phox*^(NCF4), and the small GTPase Rac2, driving the production of superoxide radicals. Recent studies have confirmed that phosphorylation of p47^*phox*^ at multiple sites during phagocytic stimulation, together with Rac2 activation, is indispensable for full NOX2 oxidase activity [32, 33]. NOX1 employs a distinct set of organizer and activator proteins, including NOXO1, NOXA1, and Rac. Recent work has demonstrated that NOXO1 critically regulates the magnitude of ROS output by the NOX1 complex and further shows that NOX1-dependent redox signaling interacts with EGFR signaling pathways via adapter proteins such as Erbin [10, 34, 35]. NOX3 plays a critical role in the inner ear, particularly in vestibular development and auditory neuron function. Similar to NOX1 or NOX2, NOX3 also depends on accessory subunits such as p22^*phox*^ and NOXO1 for its enzymatic activity and stability. A 2024 review by Marc Herb focused on emerging genetics and functional data, emphasizing the pivotal role of NOX3-derived ROS in both normal auditory physiology and the pathophysiology of acquired hearing loss [36]. Unlike other NOX isoforms, NOX4 is constitutively active once expressed and requires little or no stimulation by cytosolic organizer proteins. Polymerase δ-Interacting Protein 2 (Poldip2) is a critical regulatory partner that regulates NOX4 activity and localization in vascular smooth muscle cells. Subsequent research has investigated the role of Poldip2, demonstrating its effects on cytoskeletal organization, focal adhesion turnover, and cell migration through Rho-dependent signaling pathways [37, 38]. Unlike from DUOX1 and DUOX2, NOX5 is unique among NOX family members because it is regulated directly by calcium binding. A recent cryo-EM study revealed dynamic structural shifts in full-length human NOX5 upon calcium engagement, demonstrating that these conformational changes activate the enzyme’s electron transfer machinery and thereby promote superoxide production [39]. DUOX1 and DUOX2 contain N-terminal peroxidase-like domains, and their partners, DUOXA1 and DUOXA2, are required for proper maturation and apical membrane localization in epithelial cells. High-resolution cryo-EM structures of full-length DUOX1-DUOXA1 complexes in both high- and low-calcium states revealed a symmetric heterotetrameric architecture stabilized by extensive intersubunit interactions. Notably, recent work by Kohda and colleagues demonstrated that DUOXA not only facilitates complex assembly but also facilitates the apical sorting of DUOX enzymes within epithelial tissue [40–42]. Taken together, the unique molecular composition of each NOX isoform determines its subcellular localization, activation mechanism, and the precise nature of the ROS signals generated within the cell.

### Scope and novelty of this review

**Fig. 1:**
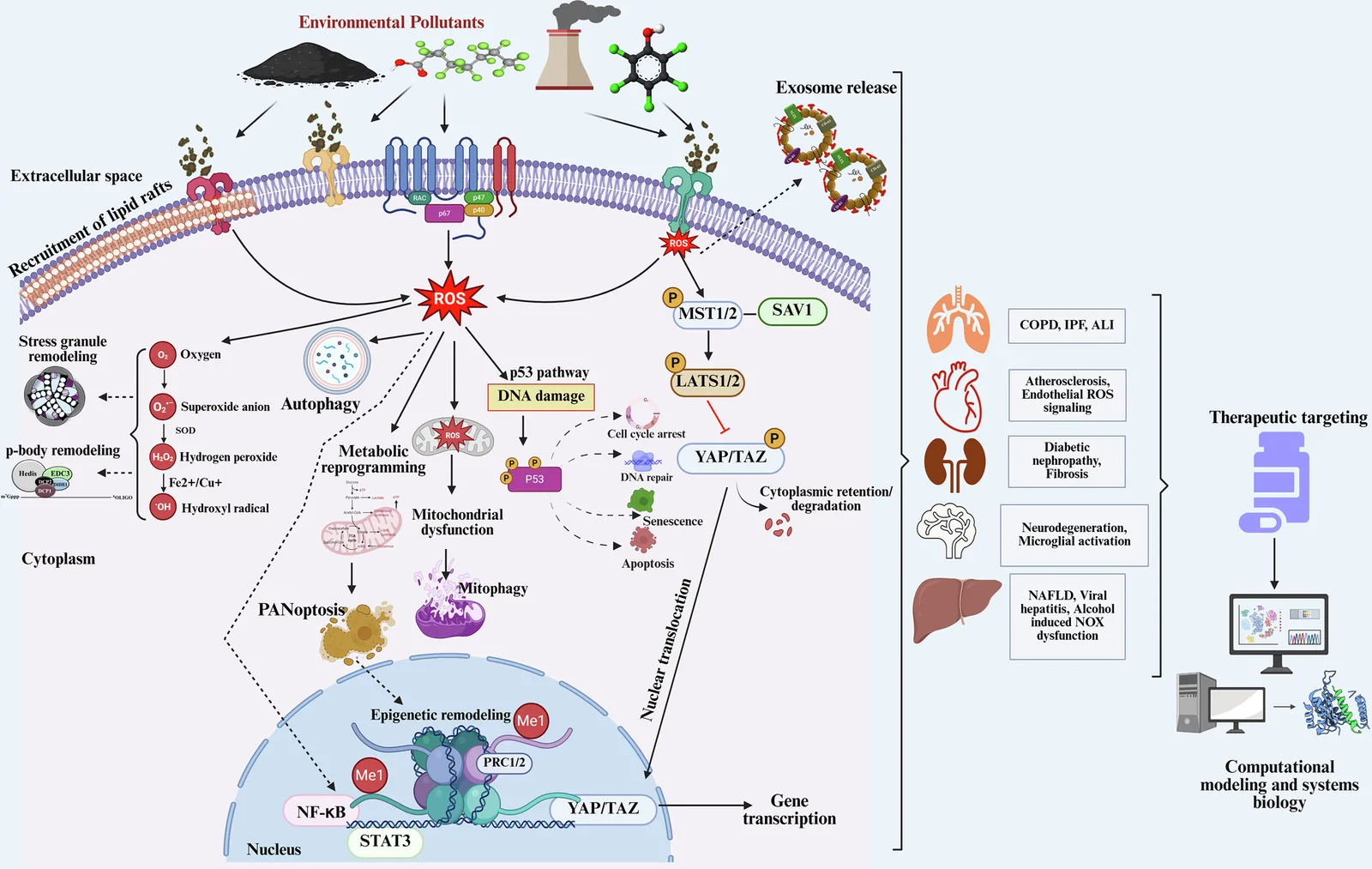
Environmental stress-induced NOX signaling involves redox imbalance, metabolic reprogramming, and stress-response pathways. Schematic illustration of environmental toxicants that activate membrane-associated and intracellular signaling cascades that converge on NADPH oxidase (NOX)-dependent reactive oxygen species (ROS) generation. Excess ROS disrupt redox homeostasis and drive metabolic reprogramming while engaging in autophagy, exosome biogenesis, PANoptosis, and Hippo-YAP/TAZ signaling. These coordinated stress responses promote epigenetic remodeling and reprogramming of inflammatory, survival, and repair pathways, contributing to chronic tissue dysfunction and disease progression

This review builds upon our previous reviews from 2015 and 2022, which summarized the central role of NOX2 in neutrophil-mediated antimicrobial defense and discussed newly emerging nonphagocytic functions across the NOX family [3, 4]. In the present work, we focus on advances made after 2020 that have reshaped our understanding of NOX enzymes, particularly regarding molecular mechanisms and the effects of exposure to various pollutants. One of the most significant advances has been the advent of high-resolution cryo-EM, which captures NOX complexes in both the resting state and the activated state. These advances provide unprecedented detail on the NOX2-p22^*phox*^ core, the recruitment and assembly of cytosolic regulatory partners, and the dynamic conformational changes that underpin oxidase activation. These experimental structures, along with predictive models from AlphaFold, are beginning to elucidate how specific mutations or post-translational modifications affect enzyme stability and signaling. Recent studies on the phosphorylation, SUMOylation, and ubiquitination of NOX highlight how post-translational modifications act as molecular switches that regulate the spatial and temporal activity of each NOX isoform [43, 44]. At the system level, single-cell transcriptomic and spatial transcriptomic studies have further revealed that NOX isoforms are not confined to neutrophils and macrophages but also function within diverse immune microenvironments [45–47]. For example, single-cell tumor datasets demonstrate enrichment of NOX2 in tumor-associated macrophages (TAMs) and correlates with inflammatory programs within TAM clusters [48, 49]. In parallel, recent mechanistic and transcriptomic work links NOX4 to macrophage polarization and tumor microenvironment remodeling in vivo and in patients [50, 51]. NOX4 has also emerged as an important regulator of wound healing and fibroblast remodeling, with studies showing that its activity supports regeneration and influences immune-fibroblast crosstalk [26]. NOX enzymes are also increasingly linked to cancer therapy resistance, immune-metabolic reprogramming, stem-cell behavior, and sterile inflammation [52–55]. Emerging data also connect NOX activity to exosome and extracellular vesicle biogenesis and release, where NOX-derived ROS can shape signaling in the tumor microenvironment and fibrotic tissue formation [56–58]. We also highlight the role of NOX enzymes, which function as key sensors and regulators of redox homeostasis and environmental stress. Their activation by pollutants and xenobiotics places them at the interface of toxicology and redox biology. This dual role of NOX makes them attractive but challenging therapeutic targets. From a broader perspective, emerging research on the circadian regulation of NOX enzymes and the impact of noncoding RNAs, such as miRNAs and circRNAs, on isoform-specific expression represents important areas for future study. These mechanisms may enable more precise therapeutic scheduling and facilitate the identification of novel biomarkers for NOX-related diseases. A conceptual framework illustrating how environmental toxicants converge on NOX-dependent redox signaling to integrate metabolic reprogramming, stress responses, and cell fate decisions is provided in Fig. 1.

## Structural and biochemical complexity of the NOX family

The NOX family is structurally and functionally sophisticated, allowing these enzymes to generate reactive oxygen species (ROS) in a highly regulated manner rather than functioning as indiscriminate oxidant systems. While our previous reviews have outlined their broad organization and regulation, several recent studies have now provided deeper insights into the molecular mechanisms by which individual NOX proteins are assembled, stabilized by partner subunits, and activated to support context-specific ROS signaling [10, 39]. High-resolution cryo-electron microscopy has clarified the structural arrangement of the NOX2-p22^*phox*^ heterodimer, revealing how transmembrane helices and heme groups are positioned to facilitate electron transport [11]. Liu and coworkers visualized the activated human NOX2-p22^*phox*^ complex bound to cytosolic regulators (p47^*phox*^, p67^*phox*^, and Rac1), capturing conformational transitions that enable ROS generation [32]. Cui and colleagues further expanded these structural insights to include other NOX family members. The full-length NOX5 structures in their study revealed how calcium binding to N-terminal EF-hand motifs drives large-scale conformational changes that activate the dehydrogenase core and enable efficient electron transfer and superoxide production [39]. DUOX complexes have been studied in both high- and low-calcium states, highlighting the role of peroxidase-like domains coupled to the regulation of hydrogen peroxide production and release [40, 41]. Another major advancement has been the identification of stabilizing factors for NOX complexes. The ER-resident chaperone EROS was shown to interact with NOX2-p22^*phox*^, protecting newly synthesized NOX2 from premature degradation and ensuring proper maturation and assembly. This interaction reveals an additional layer of complexity in NOX stability [10]. In parallel, several studies have defined post-translational modifications that are critical for tuning the cytosolic subunits involved in docking and enzyme activation [33, 43, 59–61]. Computational approaches now complement experimental data. AlphaFold-based structural predictions and complementary molecular dynamics simulations are increasingly being used to model flexible loops, interpret disease-associated mutations, and probe dynamic membrane interactions that remain challenging to resolve by cryo-EM [62]. These in silico insights are being integrated with structural and biochemical data to further delineate how each isoform operates.

### Domain organization: EF-hand, PX domain, Autoinhibitory region (AIR), NADPH-binding motifs

The structural architecture of NOX enzymes provides a blueprint for how NOX complexes generate and regulate ROS. Each catalytic subunit has six conserved transmembrane alpha helices, with histidine residues coordinating two heme groups. Cryo-EM analysis of NOX2 has confirmed its arrangement at near-atomic resolution, revealing how the transmembrane core forms a conduit for electron flow [11]. The flavin adenine dinucleotide (FAD) and NADPH-binding domains, located within the cytosolic C-terminus of NOX enzymes, facilitate the transfer of electrons to molecular oxygen and promote superoxide generation. This electron transfer mechanism underlies the respiratory burst in human neutrophils, where electron transfer to molecular oxygen generates superoxide and contributes to efficient pathogen elimination [33]. The diversity of NOX isoforms is reflected in their distinct structural domains and regulatory mechanisms. NOX5 contains N-terminal EF-hand motifs that sense intracellular calcium levels, whereas DUOX enzymes possess both EF-hands and a peroxidase-like domain, facilitating the generation of hydrogen peroxide, which is involved in mucosal defense functions [39, 63, 64]. Cytosolic regulators such as p47^*phox*^, and p40^*phox*^ in NOX2, along with NOXO1 in NOX1, use Phox homology (PX) domains for precise lipid binding and targeted membrane recruitment. These proteins also contain autoinhibitory regions and SH3 domains that coordinate complex assembly and activation, thereby preventing excessive or unintended ROS generation [65].

### Post-translational modifications (PTMs)

Post-translational modifications (PTMs) represent a critical layer of regulation for NOX enzymes, shaping the timing, localization, and duration of ROS generation. The major post-translational modifications regulating NOX isoforms, their modifying enzymes, functional outcomes, and their disease relevance are summarized in Table 1. These modifications exert broad control over NADPH oxidases by modulating their assembly, membrane targeting, catalytic activity, and degradation in a context-dependent manner. Our 2022 review emphasized the importance of post-translational regulation; since then, several recent studies have substantially broadened our understanding of these regulatory mechanisms [4, 66–68]. The best-characterized post-translational modification in NOX biology is phosphorylation. Phosphorylation of p47^*phox*^ by MAPKs and AKT promotes the conformational changes that are necessary for cytosolic subunits to translocate and dock onto the membrane-bound NOX2 complex during neutrophil activation, thereby enabling the assembly of an active oxidase complex [33, 66, 69, 70]. Recent work has identified ROCK2 (Rho-associated coiled-coil kinase 2) as a key kinase that interacts with p22^*phox*^ to promote p47^*phox*^ phosphorylation, providing a membrane-proximal route to activate the oxidase [67]. Apart from phosphorylation, other PTMs restrain or fine-tune signaling. SUMO1, for example, negatively regulates the ROS produced by NADPH oxidase, acting as a rapid brake on excessive oxidase activation [43]. Ubiquitination tightly controls the quality, stability and lifetime of the oxidase complexes. ER-associated degradation targets p22^*phox*^ via Derlin-1 when NOX2-p22^*phox*^ assembly fails, thereby eliminating improperly folded or unassembled complexes [60]. In parallel, the deubiquitinase CYLD regulates the ubiquitination and proteasomal turnover of NOXO1, thereby restraining NOX1 signaling in epithelial and cancer cells [61]. Moreover, reactive nitrogen species provide an additional regulatory layer; S-nitrosylation of p47^*phox*^ modulates CK2-dependent phosphorylation, thereby contributing to NADPH oxidase deactivation during nitric oxide bursts [71]. Together, these PTMs integrate upstream signaling with structural control of NOX complexes, ensuring that ROS generation is tailored to the cellular context both spatially and temporally.

**Table 1:** Studies that demonstrate important post-translational modifications of NOX subunits

PTM type	Methods/analytical tool	Target NOX isoform/subunit	Model/system	Major findings	References
Phosphorylation	Metabolic ³²P labeling, immunoprecipitation, SDS-PAGE/autoradiography, phosphoamino acid analysis, phospholipase D manipulation/inhibition, NADPH oxidase activity measurement	NOX2 complex, p22^*phox*^ subunit dependency	Human neutrophils	p22^*phox*^ undergoes stimulus-dependent phosphorylation predominantly on threonine residue(s). p22*phox* phosphorylation correlates with NADPH oxidase activity and is regulated through both phospholipase D-dependent and -independent mechanisms, supporting phosphorylation as a regulatory event associated with NOX2 oxidase function.	[521]
	In vitro kinase assays, phospho-specific antibodies, and immunoblotting	p47^*phox*^, regulatory subunit of NOX2 complex	Human neutrophils undergoing phagocytosis	Phosphorylation of p47^*phox*^ on multiple serine residues (including Ser304, Ser315, Ser320, and Ser328) is essential for NOX2 activation during phagocytosis, providing a mechanistic basis for respiratory burst in innate immunity	[522]
SUMOylation	Co-immunoprecipitation, SUMOylation assays, ROS detection assays	NOX5	HEK293 and COS-7 cells overexpressing NOX isoforms; primary human cells for validation	SUMOylation is an inhibitor of NADPH oxidase activity provides a post-translational “brake” to prevent excessive ROS and oxidative stress	[43]
Ubiquitination	Co-immunoprecipitation, ubiquitination assays, Western blot	NOX2	Pancreatic acinar cells (AR42J)(acute pancreatitis models)	Alleviates NLRP3 inflammasome activation and pyroptosis, suggesting NOX2 ubiquitination as a protective mechanism in inflammatory injury	[523]
	Co-immunoprecipitation, ubiquitination assays (CRISPR/Cas9-mediated knockout), proteasome inhibition, Western blot	NOXO1, NOX organizer subunit	HEK293T and PC-3 prostate cancer cell lines, in vitro and in vivo tumor models	Suppresses ROS-driven prostate cancer progression, identifies CYLD as a tumor suppressor via NOXO1 regulation	[61]
	Immunoprecipitation, ubiquitination assays, and Western blot for protein stability	p22^*phox*^	Mouse cerebrovascular SMCs, RNF34 knockout mice	RNF34 is a negative regulator of NOX2 activity via p22^*phox*^ ubiquitination, relevant to vascular remodeling and hypertension	[524]
	Immunoprecipitation (IP) + Western blot; possibly ubiquitin pull-down assays; mass spectrometry	NOX2/ gp91^*phox*^ subunit	CD8+ regulatory T cells from SLE patients	NOX2 dysfunction contributes to defective suppressive function of CD8^+^ Tregs, promoting autoimmunity in SLE; targeting of the Notch1-NOX2 axis can potentially restore Treg function	[525]
Glutathionylation/Nitrosylation	Biochemical S-nitrosylation assays, site-directed cysteine mutagenesis, phosphorylation assays, Western blot-based signaling analysis	NOX2 and p47^*phox*^	HEK293T cells	S-nitrosylation of p47^*phox*^ at a defined cysteine residue (Cys196) alters its structural conformation and enhances phosphorylation by casein kinase 2 (CK2), establishing cysteine-based redox post-translational modification as a regulatory switch controlling NOX2 complex activity through redox phosphorylation crosstalk	[71]
	Biotin-switch assay, mass spectrometry, site-directed mutagenesis (Cys694), ROS enzyme assays, co-expression with Trx1 & GSNOR	NOX5	COS-7 cells; human endothelial & smooth muscle cells	NO directly S-nitrosylates NOX5, inhibiting activity reversibly; Cys694 critical site (mutation abolishes NO sensitivity); denitrosylation by Trx1/GSNOR restores activity; sensitivity ranking: NOX1 ≥ NOX3 > NOX5 > NOX2 > NOX4 (resistant)	[526]
	Superoxide assays (lucigenin, DHE), DAN assay, anti-nitrosocysteine blot, siRNA (NOX2/NOX4), UV-denitrosylation	p47^*phox*^	HMEC-1 + NO donors (DETA-NONOate, SNAP, SNP)	NO suppresses NOX-derived superoxide via S-nitrosylation of p47^*phox*^, independent of subunit expression, cGMP, or peroxynitrite. UV reversal confirms modification is reversible	[527]
Acetylation/Deacetylation	Western blot using pan-acetyl-lysine antibodies; immunoprecipitation; HAT & HDAC inhibitors; mass spectrometry; cell-free systems	Rac1 and p67^*phox*^	Human monocytes (THP-1 cells), polymorphonuclear granulocytes; mouse model (ApoE-knockout)	Deacetylation of Rac1 at *lysine-166* (K166) by SIRT1 reduces Rac1’s binding affinity for GTP and for p67^*phox*^; this impairs the recruitment of p67^*phox*^, lowering NOX activation and superoxide production. Inhibiting HDACs increases superoxide production, activating SIRT1	[528]

*Cas9* CRISPR-associated protein 9, *CRISPR* clustered regularly interspaced short palindromic repeats, *HAT/HDAC* histone deacetylase, *HEK293T* cells human embryonic kidney 293 cells, *HMEC-1* human endothelial cells, *NAC* N-acetylcysteine, *NADPH* nicotinamide adenine dinucleotide phosphate, *NOX* NADPH oxidase, *p22phox* 22 kDa phagocyte oxidase subunit, *p47phox* 47 kDa phagocyte oxidase subunit, *PTM* post-translational modification, *ROS* reactive oxygen species, *SIRT1* Sirtuin 1 (NAD⁺-dependent deacetylase), *SLE* systemic lupus erythematosus, *SMCs* smooth muscle cells, *DAN* assay Diaminonaphthalene assay, *SDS-PAGE* Sodium dodecyl sulfate-polyacrylamide gel electrophoresis, *siRNA* Small interfering RNA, *GTP* Guanosine triphosphate, COS-7 A fibroblast-like cell line derived from monkey kidney tissue, *ApoE* apolipoprotein E, *CD8+* cluster of differentiation 8-positive, *cGMP* cyclic guanosine monophosphate, *CK2* casein kinase 2, *DETA-NONOate* diethylenetriamine NONOate, *DHE* dihydroethidium, *GSNOR* S-nitrosoglutathione reductase, *NO* nitric oxide, *RNF34*, ring finger protein 34, *SNAP* S-nitroso-N-acetylpenicillamine, *SNP* sodium nitroprusside, *THP-1* human monocytic leukemia cell line 1, *Tregs* regulatory T cells, *Trx1* thioredoxin 1

### Developments in molecular dynamics simulations, AlphaFold, and Cryo-EM

Since 2022, structural biology has greatly expanded our understanding of NADPH oxidases. High-resolution cryo-EM has now captured the NOX2-p22^*phox*^ complex in both the resting and active states, revealing how conformational changes in catalytic core facilitate electron transfer through the FAD and heme co-factors. These structures also map activation-linked conformational changes in cytosolic docking regions and suggest potential targetable binding sites for selective inhibition that could be exploited for isoform-selective NOX inhibition [32]. AlphaFold-Multimer has been applied to predict assemblies of NOX isoforms and their regulatory subunits with NOXO1 and NOXA2, extending the capabilities of standard AlphaFold 2 predictions to NOX complexes. These models now inform site-directed mutagenesis and are beginning to guide structure-based inhibitor design, which helps in filling the gaps where high-resolution experimental structures are still lacking [72–74]. Molecular dynamics simulations have further refined these models by providing a framework for assessing how small molecules might stabilize inactive NOX2 states or disrupt the assembly of the phagocytic complex, thereby probing transmembrane dynamics and lipid interactions [75]. Taken together, cryo-EM, AlphaFold, and MD-based modeling are shifting NOX research toward structure-guided targeting of the enzymes themselves rather than relying on nonspecific ROS scavenging [62].

### Isoform-specific subunit assembly and membrane localization

The spatial distribution of NOX isoforms, together with their dynamic assembly into functional complexes, dictates the qualitative and quantitative features of ROS signaling. In the best-studied prototype, NOX2, activation is initiated by stimulus-dependent recruitment of cytosolic subunits (p47^*phox*^, p67^*phox*^, p40^*phox*^, and Rac) to the membrane-bound flavocytochrome b558 complex [76]. Recent live-cell and superresolution imaging studies have shown that this assembly is dynamic and reversible, with NOX2 nanoclusters growing and redistributing to the plasma and phagosome membranes during phagocytosis, enabling neutrophils to adjust their ROS output as phagocytosis proceeds [76–79]. In epithelial cells, NOX1 forms functional complexes with the organizer NOXO1 and the activator NOXA1. Recent work by Hebchen’s group demonstrated that NOXO1 affects the level of NOX1-derived ROS and can be coupled with EGFR signaling via its interaction with the adapter protein Erbin [80]. Herb and coworkers reported that NOX3 has relatively modest subunit requirements and is strongly dependent on the surrounding membrane environment, particularly within the inner ear sensory epithelium [36]. In contrast, NOX4 is distinct from other NOX isoforms because it remains active once expressed; its localization to the ER, nucleus, and mitochondria depends on binding partners such as Poldip2. Studies in vascular and lung cells confirm that this interaction stabilizes NOX4 and affects downstream signaling [37, 81]. DUOX1 and DUOX2 are targeted to the apical surface of epithelial cells via their specific maturation factors, which are co-expressed via bidirectional promoters. Kohda and coworkers performed a mechanistic study and reported that DUOXA1 glycosylation is crucial for the apical sorting of the DUOX1-DUOXA1 complex, which promotes the secretion of hydrogen peroxide into the lumen for mucosal defense and thyroid hormone biosynthesis [42]. However, DUOXA2-driven apical targeting of DUOX2 depends more heavily on its C-terminal region than on its glycosylation state. Overall, these compartmentalization dynamics create localized redox microdomains. Within these microdomains, hydrogen peroxide can selectively oxidize phosphatases such as phosphatase and tensin homolog (PTEN), thereby tuning kinase signaling cascades and enabling precise control of transcriptional programs and immune responses [82, 83].

### ROS compartmentalization and localized redox signaling

Recent work has reframed ROS from indiscriminate toxins to compartmentalized second messengers which operates within defined microdomains [84]. In immune cells, NOX2-derived ROS in phagosomes not only are antimicrobial but also regulate phagosome maturation, antigen processing, and autophagy by oxidizing key cysteine residues [85]. In addition to phagosomes, other NOX isoforms can shape local vascular signaling. NOX1 generates superoxide, while NOX4 generates hydrogen peroxide in endothelial and vascular smooth muscle cells, where their activity in autocrine and paracrine circuits regulates vasodilation, cell growth and differentiation, and inflammatory signaling [86]. At mitochondria-associated ER membranes (MAMs), NOX4 participates in localized redox pathways that regulate Inositol 1,4,5-trisphosphate receptor (InsP3)-receptor-dependent calcium transfer from the ER into mitochondria and influence cell survival [87]. The ability to track ROS dynamics in living cells has been improved by the use of genetically encoded redox biosensors and advanced superresolution imaging [88, 89]. These approaches reveal that many NOX-derived ROS signals are spatially restricted and rapidly consumed, resulting in highly localized signaling domains. In cancer, NOX-dependent ROS microdomains within the tumor microenvironment influence both tumor-immune cell interactions and proproliferative signaling pathways, thereby shaping immune evasion and tumor progression [90]. Recent research on NOX inhibitors has shown that compartment-specific targeting is both feasible and essential, as indiscriminate ROS scavenging disrupts physiological signaling but fails to suppress pathological ROS-driven processes [62, 91]. Taken together, these advances mark a breakthrough in how NOX enzymes are understood. They are no longer seen as simple electron transporters but are now recognized as multidomain, multifunctional redox regulators whose activity is choreographed by structural modules, post-translational modifications, subunit dynamics, and spatial localization. The integration of cryo-EM, AlphaFold, and molecular dynamics simulations now allows the linkage of structural transitions to functional outcomes with exceptional clarity. This shift deepens the mechanistic framework of NOX biology and opens new therapeutic avenues across immunity, cardiovascular health, fibrosis, and cancer. Moreover, critical gaps remain. These include how NOX-derived signals integrate with metabolic and receptor-driven pathways, how they influence metabolic crosstalk and how the pharmacological control of ROS can be translated into clinical benefit.

## NADPH oxidases in classical and emerging immune signaling (comprehensive coverage of NOX in immunity, cell death, and stress)

**Fig. 2:**
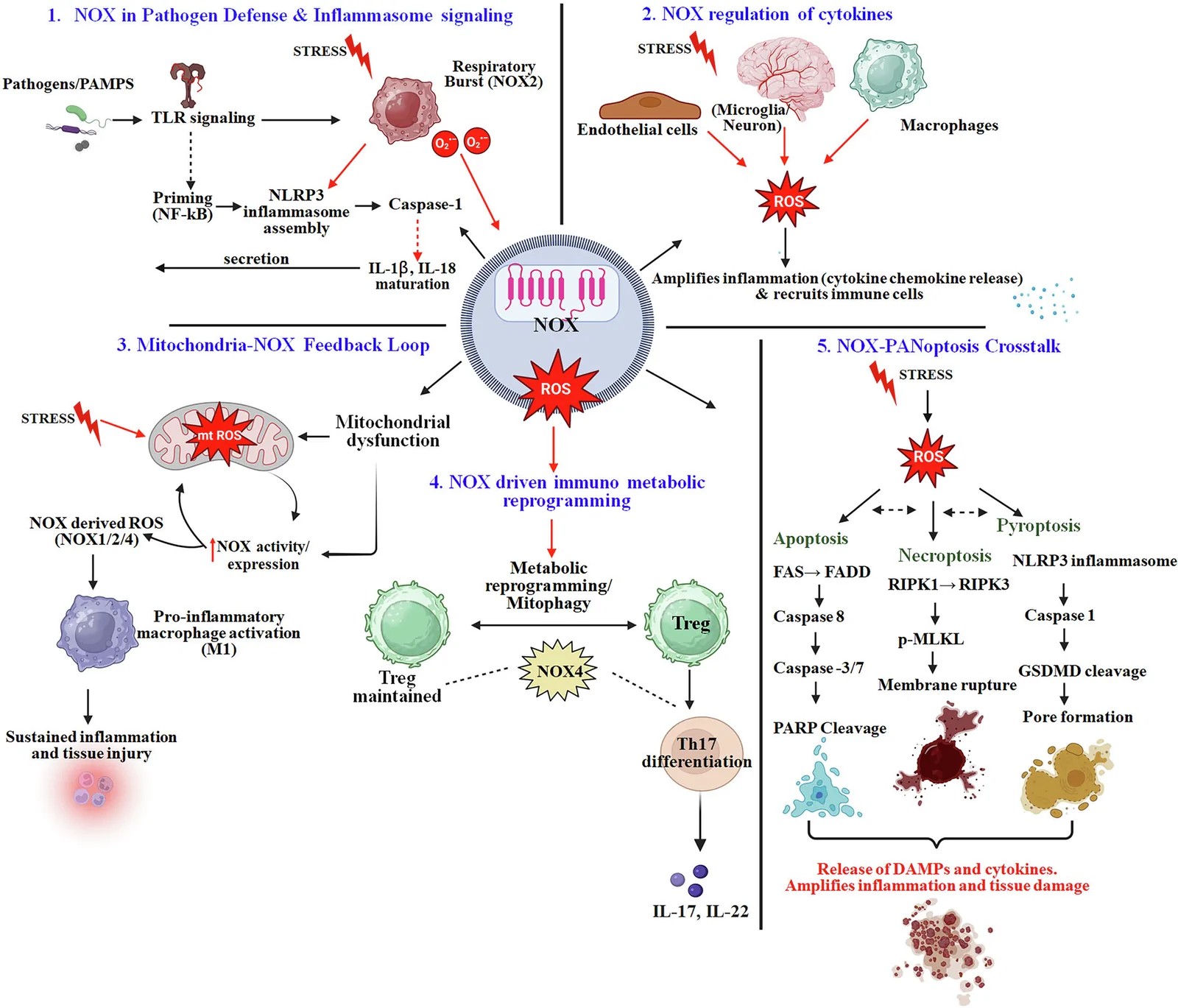
Compartmentalized NOX signaling shapes immune activation and inflammatory cell fate. Mechanisms through which distinct NADPH oxidase (NOX) isoforms generate spatially restricted reactive oxygen species (ROS), regulating immune activation, inflammatory signaling, and programmed cell death. NOX-derived ROS influence macrophage polarization, T-cell differentiation, and cytokine production while amplifying mitochondrial oxidative stress and inflammatory feedback loops. Under physiological conditions, compartmentalized redox signals fine-tune immune responses. However, when dysregulated, under stress responses it promotes chronic inflammation and immune-mediated tissue injury

NOX subunits play crucial roles in modulating host defense mechanisms and cellular signaling, as outlined in our earlier work [4]. Originally, NOX2 was defined for its prominence in the phagocytic “respiratory burst” for direct microbial killing, where ROS also act as a redox second messenger that reshapes neutrophil metabolism [92]. At the plasma membrane, endosomes, or phagosomes, localized NOX-derived ROS modulate innate immune-receptor-mediated signaling, including that of TLRs, and contribute to both the priming and amplification of the NOD-like receptor protein 3 (NLRP3) inflammasome in a stimulus-dependent manner, thereby influencing cellular homeostasis [93, 94]. Recent studies have indicated that ROS can accumulate within stress granules and influence RNA-binding proteins such as the stress granule nucleator G3BP1 and the P-body component GW182, thereby altering RNA granule dynamics and posttranscriptional regulation of stress-responsive transcripts [95]. While direct evidence specifically linking NOX isoforms to stress granule or P-body remodeling remains limited, localized ROS generation by NOX complexes makes this a plausible regulatory mechanism that warrants further investigation. As summarized in Fig. 2, NOX isoforms generate spatially restricted ROS signals that shape immune activation, cytokine production, mitochondrial feedback loop and regulated cell death across innate and adaptive immune compartments. Thus, NOX enzymes are well positioned to integrate pathogen sensing and environmental stress with downstream cell fate, balancing defense, inflammation, and cellular homeostasis.

### NOX in pathogen defense: ROS bursts, TLRs, NLRP3, and inflammasome activation

The host’s innate immune response depends on multiple steps to neutralize invading pathogens, with NOX enzymes serving as a central hub. At its core, the primary function of NOX, particularly NOX2 in phagocytic cells such as neutrophils and macrophages, is to drive the oxidative burst in the phagosome, which generates highly reactive superoxide (O₂^•−^), which is essential for microbial killing. This direct microbicidal action is critical, as demonstrated by the severe immunodeficiency observed in patients with chronic granulomatous disease (CGD), where genetic defects in NOX render phagocytes unable to produce this essential ROS burst [96]. Studies have shown that ROS can mediate Toll-like receptor signaling, which is essential for recognizing pathogen-associated molecular patterns (PAMPs). Functionally, this enhancement often involves the oxidative modification of key signaling intermediates. For example, ROS can inactivate inhibitory phosphatases, such as protein tyrosine phosphatases (PTPs), by oxidizing their catalytic cysteine residues [97, 98]. This disruption of negative regulation allows for the continued phosphorylation and activation of downstream kinases, ultimately leading to robust activation of transcription factors such as NF-κB and AP-1, which drive the expression of proinflammatory cytokines [99, 100]. The NLRP3 inflammasome is a multiprotein complex involved in the innate immune response that is activated by NOX-derived ROS, leading to the production of proinflammatory cytokines. A study by Zhou et al. published in *Nature* demonstrated that mitochondrial dysfunction and the accumulation of ROS-generating mitochondria promote NLRP3 inflammasome activation in macrophages, highlighting mitochondrial ROS as an important signal linking mitochondrial damage to inflammasome activation [101]. Furthermore, additional studies suggest that converging pathways activate the NLRP3 inflammasome via ROS [101–104]. Research has shown that ROS can directly alter NLRP3 inflammasome signaling, leading to a conformational change that promotes its oligomerization and subsequent assembly [105]. A 2013 study by Muñoz-Planillo et al. in *Immunity* demonstrated that in various cell types, including macrophages and dendritic cells, potassium efflux is the common trigger of NLRP3 inflammasome activation by a wide range of stimuli, a process that can be indirectly potentiated by ROS [103]. In essence, NOX-produced ROS serve two distinct purposes: [i] they act as direct weapons to destroy pathogens in the phagosome, while [ii] they function as remote signaling molecules that prime the inflammasome for activation via crosstalk between organelles.

### NOX-mediated modulation of cytokine/chemokine release (IL-6, IL-8, CCL2)

NOX isoforms expressed in parenchymal and immune cells are major sources of ROS generation in inflammatory microenvironments [1, 106]. NOX-derived ROS act as secondary messengers, increasing the expression and secretion of proinflammatory cytokines and chemokines and thereby contributing to chronic liver inflammation, fibrosis, and the development and progression of hepatocellular carcinoma (HCC). Upon exposure to diverse stressors, the constitutively active isoform NOX4 generates elevated levels of ROS. In the context of liver injury, NOX4 expression is upregulated, leading to inflammation. For instance, in alcoholic liver disease (ALD), NOX4 stabilizes CCL2 and CCR2 mRNAs, thereby enhancing the recruitment of inflammatory cells to the liver [107]. In addition, NOX4 has been implicated in vascular inflammation associated with aging and atherosclerosis, where it contributes to the increased expression of IL-6 and CCL2, ultimately leading to lesion development and vascular dysfunction [108]. NOX1 is expressed primarily in colon epithelial cells, where the synergistic binding of TNF-α and IL-17 activates NOX1 via NOXO1 upregulation, generating ROS that drive the production of downstream inflammatory mediators, including lipocalin-2 [109]. IL-8/CXCL8 is a chemokine that recruits neutrophils to sites of infection or injury. ROS generated by NOX enzymes can regulate the expression of IL-8 in epithelial and renal tubular cells. In human proximal tubular HK-2 cells, hypoxia-reoxygenation-induced ROS generation activates Transient Receptor Potential Ankyrin 1 (TRPA1) and downstream MAPK/NF-κB signaling, leading to the induction of IL-8, which contributes to the recruitment of neutrophils and the propagation of inflammation [110].

Collectively, these studies demonstrate that NOX-derived ROS are important modulators of cytokine and chemokine networks. By regulating the production of proinflammatory mediators such as IL-6, IL-8, and CCL2 in both parenchymal and immune cells, NOX enzymes shape the inflammatory microenvironment and contribute to disease progression, fibrosis, and tumor development. Consequently, targeting NOX-dependent signaling pathways has emerged as a promising therapeutic strategy for mitigating excessive inflammation in a variety of pathological conditions, including cancer. In addition to their roles in tumors and inflamed tissues, NOX-dependent regulation of cytokine and chemokine responses is evident in other immune-relevant organs, such as the spleen and lungs. NOX2-derived ROS generated by macrophages and dendritic cells modulate cytokine production and immune cell activation, thereby influencing systemic inflammatory responses [111]. In the lung, epithelial and endothelial NOX isoforms, particularly NOX1, NOX2, and NOX4, regulate the production of IL-6, IL-8, and CCL2 in response to inflammatory cytokines, hypoxia, particulate matter, and environmental toxicants, promoting neutrophil recruitment, airway inflammation, and tissue remodeling [22, 23, 110]. These observations highlight that NOX-driven cytokine networks represent a conserved mechanism of inflammation across organs, with tissue-specific outcomes shaped by local cell populations and microenvironmental cues [112, 113].

### Crosstalk between NOX and PANoptosis: pyroptosis, apoptosis, and necroptosis integration

**Fig. 3:**
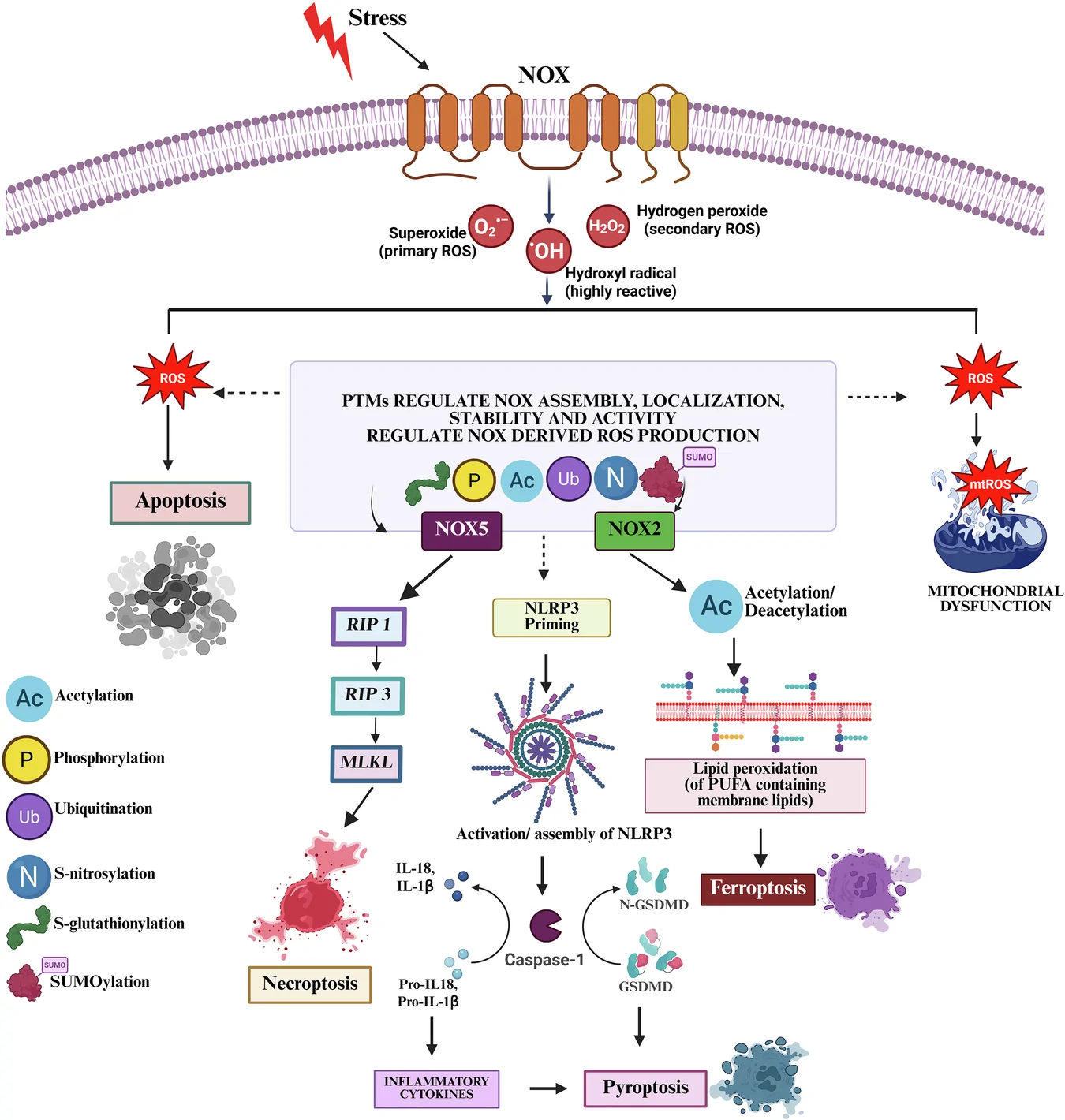
NOX-derived reactive oxygen species orchestrate PANoptotic cell-death programs. Schematic representation of the role of oxidative PTMs as central regulators of PANoptosis through the coordination of apoptotic, necroptotic, pyroptotic, and ferroptotic signaling pathways. NOX-driven redox signals modulate death-receptor activation, inflammasome assembly, mitochondrial dysfunction, and lipid peroxidation, thereby integrating multiple regulated cell-death mechanisms into a unified stress-response outcome during inflammation and toxicant exposure

Recent advancements related to NOX-derived ROS and their integration with cell-death pathways have received significant attention. In parallel, the term PANoptosis has been introduced to describe an inflammatory programmed cell-death pathway that integrates pyroptosis, apoptosis, and necroptosis [114]. An integrated overview of NOX isoforms involved in pyroptosis, apoptosis, necroptosis, and PANoptosome signaling across tissues is provided in Table 2. This pathway is coordinated by the assembly of the PANoptosome, a supramolecular complex that concurrently activates all three cell death modalities, inducing immunogenic cell death and counteracting immune evasion mechanisms. Pharmacological modulation of these interconnected cell-death pathways highlights the therapeutic potential of targeting dysregulated PANoptosis and associated oxidative stress [115]. Understanding the crosstalk between NOX isoforms and PANoptosis is crucial for developing therapeutic strategies that target excessive cell death in diseases such as cancer, neurodegenerative disorders, and inflammatory conditions, where dysregulated PANoptosis amplifies tissue damage and immune dysfunction [115]. How NOX-derived ROS coordinate apoptotic, necroptotic, pyroptotic, and ferroptotic signaling within Panoptotic complexes is illustrated in Fig. 3.

**Table 2:** NOX isoforms in ROS-driven PANoptosis and cell-death pathways

PANoptosis component/type	NOX isoform/subunit	Regulatory mechanism	Biological model/system	Functional outcome	References
Pyroptosis	NOX4	ROS-mediated activation	Neurons in an intracerebral hemorrhage (ICH) model (likely murine or rat)	NOX4 knockdown reduces mitochondrial ROS, suppressing inflammasome activation and pyroptotic neuronal death	[529]
	NOX2/NOX4	ROS-mediated activation	H9c2 cardiomyocytes under stress	NOX-derived ROS activates the NLRP3 inflammasome, leading to caspase-1 activation and pyroptotic cell death	[530]
	NOX	ROS-mediated activation	Fischer 344 (F344) rats, human cardiomyocytes AC16 / cardiac tissue exposed to silica nanoparticles	Activation of NLRP3 inflammasome and caspase-1, leading to pyroptotic cell death and cardiac hypertrophy	[531]
	DUOX1	ROS-mediated activation triggers NLRP3/caspase-1	Human cardiomyocytes AC16	Increased DUOX1-derived ROS induces caspase-1-dependent pyroptosis	[532]
	NOX2	ROS-mediated activation (lipid-raft localization enhances NOX2 activity; specific PTMs not reported)	THP-1 cells/vascular tissue in apoE^−/−^ mice treated with homocysteine (Hcy)	Activation of NLRP3 inflammasome and caspase-1, promoting pyroptosis and atherosclerotic lesion formation	[533]
Apoptosis	NOX1	ROS-mediated activation	Human gastric cancer AGS, MKN74, MKN1, SNU-668 and rat gastric epithelial cell line RGM-1	Lutein activates NOX, increasing ROS levels and inducing caspase-dependent apoptosis	[534]
	NOX4	ROS-mediated activation	H9c2 cell line, Specific pathogen-free (SPF) male C57BL/6 mice, ASMase^*flox/flox*^ (ASMase^*fl/fl*^) mice and myosin heavy chain 6 (Myh6)-Cre mice	ASMase activates NOX4, increasing ROS and triggering caspase-dependent apoptosis	[535]
	NOX1	miR-145-5p-mediated downregulation of NOX1	Male Sprague-Dawley (SD) rats, and the H9c2 cell line	Suppression of NOX1 reduces ROS generation, attenuates inflammatory cytokine release, and decreases caspase-dependent apoptosis	[536]
	NOX2	ROS-mediated activation	Oligodendrocytes exposed to ultrafine diesel exhaust particles	Increased NOX-derived ROS triggers caspase-dependent apoptosis	[186]
	NOX1	ROS-mediated activation	Human colonic epithelial cells (NCM460) exposed to alcohol	NOX1-derived ROS induces oxidative stress, leading to apoptosis and intestinal barrier injury	[330]
	NOX2/p47^*phox*^	ROS-mediated activation	HEK293 cells exposed to patulin	NOX-derived ROS induces oxidative stress, leading to cytotoxicity and apoptosis	[537]
Necroptosis	NOX2	ROS-mediated activation	C57BL/6J mice and H9c2 cell line	NOX-derived ROS promotes RIP1-RIP3-MLKL pathway activation, triggering necroptotic cell death; GSK2795039 inhibits NOX and suppresses necroptosis	[120]
	NOX1	ROS-mediated activation	Human gastric cancer AGS cells	Astaxanthin induces NOX-derived ROS, activating RIPK1-mediated necroptosis	[538]
	NOX1	ROS-mediated activation	Human colorectal cancer cells (CRC)	Rubiarbonol B activates NOX1, leading to ROS generation and RIPK1-dependent necroptosis	[116]
PANoptosome formation	NOX1, NOX2, NOX4, NOX5	ROS-mediated activation; can involve PTMs such as phosphorylation, acetylation, or ubiquitination when studying complex formation	Various cell types exposed to TNF and other death ligands; in vitro cell models	Cell exposure to TNF and other death ligands allows the assessment of NOX-containing protein complexes that regulate ROS generation during regulated cell death	[539]
Ferroptosis	NOX4	ROS-mediated lipid peroxidation (activation of ferroptotic pathways)	Cardiomyocytes under oxidative stress	Lipid ROS accumulation triggers ferroptotic cell death	[539]
	NOX2/NOX4	ROS-mediated activation	C57BL/6 mice, renal tubular epithelial cells	NOX-derived ROS promotes lipid peroxidation, leading to ferroptotic cell death; inhibition of NOX reduces ferroptosis	[540]
	NOX-mimicking nanozyme (inorganic catalyst, not classical NOX isoform)	Artificial NOX catalytic activity, ROS (lipid peroxidation); blocks glutathione regeneration	4T1 cancer cells	Enhanced lipid ROS accumulation and ferroptotic death in tumor cells	[541]

*DUOX1* dual oxidase 1, *HEK293T* cells human embryonic kidney 293 cells, *ICH* intracerebral hemorrhage, *MLKL* mixed lineage kinase domain-like protein, *NLRP3* NOD-, LRR- and pyrin domain-containing protein 3, *NOX* NADPH oxidase, *p47phox* 47-kDa phagocyte oxidase subunit, *PTM* post-translational modification, *RIPK1* receptor-interacting protein kinase 1, *RIPK3* receptor-interacting protein kinase 3, *ROS* reactive oxygen species, *TNF* tumor necrosis factor, *4T1* murine mammary carcinoma cell line, *AC16* human ventricular cardiomyocyte cell line, *AGS* human gastric adenocarcinoma cell line, *apoE*^−/−^ apolipoprotein E knockout, *ASMase* acid sphingomyelinase, *C57BL/6* mouse strain, *CRC* colorectal cancer, *Cre* Cre recombinase, *F344* Fischer 344 rat strain, *H9c2* rat embryonic cardiomyoblast cell line, *Hcy* homocysteine, *miR-145-5p* microRNA-145-5p, *MKN1* human gastric cancer cell line MKN1, *MKN74* human gastric cancer cell line MKN74, *NCM460* normal human colon mucosal epithelial cell line, *RGM-1* rat gastric mucosal cell line 1, *SD* Sprague-Dawley rat strain, *SNU-668* human gastric cancer cell line SNU-668, *SPF* specific pathogen-free

In particular, ROS produced by NOX enzymes can be influenced by PANoptosome activation, and vice versa. NOX1-derived superoxide anions contribute to inflammatory signaling and have been implicated in driving apoptosis and necroptosis under stress conditions in colorectal cancer cells [116]. Genetic deletion of NOX2 strikingly reduced NLRP3 formation, cleavage of caspase-1, and IL-1β release after traumatic brain injury, demonstrating that NOX2-driven ROS are functional upstream of the NLRP3/caspase-1 axis in vivo [117]. Concurrently, inflammasome-dependent caspase-1 activity in macrophages has been shown to regulate NOX2 function at the phagosomal membrane, linking inflammasome activation to the spatial control of ROS generation during innate immune responses [118], thereby demonstrating bidirectional crosstalk between inflammasomes and NOX complexes. In parallel, RIPK3, a central mediator of necroptotic and PANoptotic signaling, promotes mitochondrial dysfunction and oxidative stress, contributing to tissue injury during sepsis-induced acute kidney injury [119]. Studies on HK-2 cells have shown that lipopolysaccharide (LPS)-induced necroptotic injury depends on RIP3-MLKL signaling and is accompanied by the upregulation of NOX1 and NOX4 expression, which results in the activation of NOX enzymes downstream of necroptosis drivers [119]. Additionally, inhibition of NOX2 with the selective inhibitor GSK2795039 suppressed RIP1-RIP3-MLKL necroptotic signaling and cardiomyocyte death in doxorubicin and ischemia-reperfusion models, indicating that NOX-derived ROS are critical nodes in necroptosis/PANoptosis crosstalk [120]. Consistent with this, vascular studies clearly reveal specific roles that demonstrate how different ROS moieties influence downstream death programs [121, 122]. A study by Dikalov and colleagues in rat aortic smooth muscle cells (RASMCs) demonstrated that NOX4 provides basal hydrogen peroxide (H_2_O_2_), whereas NOX1 is the principal source of angiotensin II-stimulated superoxide (O₂^•−^). Additionally, the results from the same study underscore how different ROS species generated from different NOX isoforms differentially feed into downstream pathways, such as inflammasome activation or mitochondrial damage, both of which are related to PANoptotic signaling [123].

In cerebral endothelial cells, TNF-α rapidly activates NOX4 to produce O₂^•−^ and induce apoptotic cell death [124], providing a vascular example in which NOX-driven ROS drive cells toward apoptotic death. Reviews and mechanistic studies in microglia and endothelial systems further confirm the role of NOX4 in NLRP3 regulation [1, 125–128]. Taken together, these data support a feed-forward model in which (i) PANoptosome activation (RIPKs, caspase-8/-1, inflammasome components) induces NOX expression and activity (particularly NOX4 and NOX2); (ii) NOX-derived ROS, in turn, promotes inflammasome assembly, caspase activation and mitochondrial injury, thereby committing cells to pyroptosis, apoptosis, and (iii) isoform-selective inhibition of NOX enzymes (e.g., GKT-family compounds targeting NOX1/4 and GSK2795039 targeting NOX2), combined with ROS profiling approaches such as DHE and MitoSOX for superoxide detection, Amplex Red for extracellular H₂O₂ measurement, and ESR/CPH spin-trapping techniques for radical mapping, offers valuable experimental and therapeutic tools for dissecting NOX-PANoptosis interactions and their pathological consequences. For instance, gypensapogenin I (GI), which targets the NOX2/AMPK signaling pathway, has been shown to attenuate PANoptosis, ferroptosis, and oxidative stress in myocardial ischemia-reperfusion injury models, highlighting the therapeutic potential of targeting NOX-mediated cell-death pathways [115], illustrating how pharmacological inhibition of NOX-centered pathways can blunt multiple regulated cell-death pathways simultaneously. Collectively, these studies reinforce the view that NOX enzymes act as central regulators of PANoptosis, forming a self-amplifying loop in which ROS generation drives cell death, and in turn, cell-death signaling further enhances NOX activation. This insight is crucial because it identifies NOX as a promising therapeutic target; therefore, selectively inhibiting specific NOX isoforms could be a powerful strategy to disrupt this vicious cycle of inflammation.

Emerging evidence suggests that mitochondria and NOX enzymes engage in bidirectional feedback loops that generate sustained reactive oxygen species (ROS) signals-often described as “ROS-induced ROS release” or redoxosome-like assemblies-which are crucial for inflammasome activation and other regulated cell-death pathways [129, 130]. mtROS can induce NOX isoforms, and NOX-derived ROS can, in turn, exacerbate mitochondrial dysfunction, leading to further mtROS release and the generation of a self-amplifying cycle of redox stress that promotes cell death and inflammation. Multiple studies have provided evidence that supports this model. One such study by a group investigating hepatocellular carcinoma revealed that after sublethal heat stress, NOX4 expression increased in mitochondria, promoting an mtROS burst [131]. Pharmacologic or genetic inhibition of NOX4 reduced mitochondrial ROS levels, disrupted Nrf2/PINK1-mediated mitophagy, and shifted cells toward apoptosis rather than survival [131]. In vascular endothelial cells, angiotensin II stimulation was shown to trigger NOX2 activation and superoxide production, which reverse electron transport (RET) and enhanced mitochondrial superoxide production, creating a feed-forward amplification loop of oxidative stress [132]. These events not only increase inflammatory gene expression but also promote vascular dysfunction, suggesting the importance of redoxosomes in cardiovascular pathology. In diabetic cardiomyopathy, upregulated mitochondrial NOX4 drives excessive mtROS generation and electron transport chain dysfunction, triggering apoptosis and fibrosis; however, its deletion or inhibition reduces oxidative damage and preserves mitochondrial integrity [126, 133]. Taken together, these findings reinforce the concept that NOX-mitochondrial redoxosomes act as central platforms where mitochondrial dysfunction and NOX activity are synchronized to determine whether a cell engages in survival signaling, inflammatory cytokine release, or terminal inflammatory cell death. This spatially organized redox signaling not only shapes immune-receptor pathways but also rewires the metabolic programs that underlie immune and parenchymal cell fate decisions.

### Reprogramming of NOX and immunometabolism

Recent studies have begun to elucidate how NOX-derived immunometabolism shifts, particularly how ROS influence whether immune cells adopt pro- or anti-inflammatory phenotypes and modulate immune checkpoint pathways in cancer and chronic inflammation [134–136]. Immune cell activation is tightly coupled with metabolic reprogramming, with cells dynamically transitioning between glycolysis, oxidative phosphorylation (OXPHOS) and lipid metabolism, depending on cellular state, tissue context and lineage (for example, M1-like, effector and/or cytotoxic cells favor glycolysis, whereas many regulatory or tissue-repair phenotypes rely more on OXPHOS and fatty acid oxidation). Key examples of how NOX expression shapes metabolic states are found in macrophages. Classically activated (M1) macrophages, which are proinflammatory, rely heavily on glycolysis and pentose phosphate pathway (PPP) flux, and NOX2-derived ROS are essential for this shift. In murine macrophages, pharmacologic or genetic inhibition of glycolysis or PPP reduces NOX2 activation, along with NOX2 and p47^phox^ expression, thereby blunting the oxidative burst and dampening LPS-induced inflammation [55]. A complementary mechanism linking redox signaling to macrophage immunometabolism involves nitric oxide-mediated metabolic reprogramming. In M1 macrophages, nitric oxide suppresses mitochondrial oxidative metabolism by targeting aconitase 2 and pyruvate dehydrogenase, resulting in TCA-cycle fragmentation and reduced OXPHOS, thereby reinforcing a glycolytic, proinflammatory metabolic state [137]. Complementing these findings, a study by Liu et al. revealed that classical proinflammatory signaling shifts the macrophage metabolic program from a Myc-dependent, proliferative program to a HIF-1α-dependent, glycolysis-driven state in bone marrow-derived macrophages. Deletion of HIF-1α dampened the expression of glycolytic genes and associated bioenergetic support for the inflammatory response [138]. Together, these data highlight NOX enzymes as central regulators that fine-tune macrophage inflammatory and antimicrobial responses by integrating redox signaling with immunometabolic programs. In conjunction with metabolic pathways, inducible nitric oxide synthase (iNOS) further reshapes mitochondrial metabolism. Palmieri and coworkers used ^13^C tracing and respiration assays to map nitric oxide-mediated suppression of the TCA cycle, primarily at mitochondrial aconitase 2, causing fragmentation of the TCA cycle, a mechanism by which M1 macrophages suppress OXPHOS. These events lock M1 macrophages into a glycolytic, proinflammatory state rather than reducing inflammatory mediator production [137]. In parallel, nitric oxide-driven metabolic rewiring suppresses mitochondrial oxidative metabolism in inflammatory macrophages, reinforcing the M1 phenotype, while NOX4 signaling contributes to macrophage polarization and inflammatory activation [134, 137, 138]. The therapeutic restoration of mitochondrial function may promote the reprogramming of inflammatory macrophages into an anti-inflammatory phenotype to control disease.

### NOX and T-cell polarization

NOX-derived ROS play a critical role in balancing effector and regulatory programs in T cells. A study by Lee and colleagues demonstrated that NOX2 deficiency decreased CD25^+^ FoxP3^+^ Treg cells and increased the differentiation of effector Th cells in vitro and in vivo, thereby establishing the crucial role of ROS in maintaining the Th17/Treg balance and restraining excessive inflammatory responses [139]. Similarly, NOX2 has emerged as an important regulator of adaptive immunity, influencing T-cell differentiation, Th/Treg balance, and immune homeostasis, thereby linking redox signaling to immune regulation and tolerance [112, 139]. Taken together, these studies suggest that T-cell metabolism and T-cell polarization are tied to NOX activity, with consequences for both host defense against pathogens and chronic disease. Growing evidence indicates that NOX-derived ROS modulate immune checkpoint signaling by promoting PD-L1 expression and immunosuppressive macrophage phenotypes, thereby contributing to immune evasion within the tumor microenvironment [140, 141]. Paclitaxel treatment or glutathione depletion, both of which induce ROS accumulation, increases programmed death-ligand 1 (PD-L1) expression in macrophages through NF-κB signaling. PD-L1⁺ macrophages adopt an immunosuppressive, proangiogenic phenotype, and combining paclitaxel with anti-PD-L1 antibodies restored tumor control in a murine model of triple-negative breast cancer [140]. In addition to myeloid cells, NOX4 from stromal cells plays an immunosuppressive role. A study by Ford and coworkers revealed that NOX4 activity in cancer-associated fibroblasts (CAFs) maintains a myofibroblastic phenotype that restricts CD8^+^ T-cell infiltration into the tumor core [141]. Genetic or pharmacological inhibition of NOX4 normalizes CAFs to a more quiescent state, disrupts physical and functional barriers, increases intratumoral CD8⁺ T-cell infiltration, and synergizes with PD-1/PD-L1 blockade to restore antitumor immunity [141]. Given these studies, NOX enzymes not only regulate immunometabolic programs within immune cells but also sculpt the tumor microenvironment and immune evasion mechanisms.

Collectively, these findings identify NOX enzymes as central regulators of immunometabolic reprogramming, immune checkpoint signaling, and tumor immune evasion, underscoring their promise as therapeutic targets for cancer and inflammatory disorders.

In M1 macrophages, nitric oxide (NO) promotes metabolic reprogramming by inhibiting mitochondrial oxidative metabolism, thereby reinforcing HIF-1α-dependent glycolysis and sustaining the pro-inflammatory phenotype [138]. NO/iNOS-dependent nitric oxide production disrupts the TCA cycle by inhibiting the activity of key mitochondrial enzymes, leading macrophages to enter a proinflammatory state that predisposes T cells toward effector or regulatory phenotypes depending on the redox milieu. NOX isoforms, such as NOX1 and NOX4, are expressed in hypoxic lactate-rich environments in which the myofibroblastic stromal barrier excludes effector T cells, thereby increasing the expression of checkpoint ligands, including PD-L1, in tumors as well as in chronically inflamed tissues [141]. All these studies spanning metabolic rewiring, cytokine and chemokine stimulation, and stromal remodeling emphasize that NOX enzymes sit at a critical transition point, which can shift from protective host defense to pathological ROS signaling.

## Crosstalk between NOX and emerging cellular pathways

In this section, “emerging pathways” refer to the conserved regulatory pathways and cellular processes that intersect with redox biology and have gained increasing attention in recent years, including epigenetic chromatin regulators, Hippo/YAP-TAZ signaling, membrane microdomains (e.g., lipid rafts), extracellular vesicle (EV) communication, and circadian and metabolic programs. These pathways expand the established view of NOX enzymes into broader frameworks in which NOX isoforms are modified by transcriptional regulation, membrane modulations, cell-cell communication, and circadian rhythms. The clinical implications are twofold: (1) NOX activity can alter cell fate and the microenvironment through EV-mediated or paracrine signaling and epigenetic remodeling, and (2) cell fate, membrane compartmentalization, and circadian rhythms are accounted for in therapeutic targeting of NOX to avoid unintended consequences.

### Epigenetic crosstalk: NOX-EZH2-H3K27me3 loop and PRC2 regulation

A growing body of research indicates that NOX-derived ROS and Polycomb repressive complex 2 (PRC2), particularly its catalytic subunit EZH2 and the associated H3K27me3 histone mark, engage in bidirectional communication that can drive cells into maladaptive states, including senescence, fibrosis, and protumorigenic programs, depending on the cellular context. Early mechanistic demonstration of a positive feedback loop was reported in nucleus pulposus (NP) cells, where EZH2-mediated H3K27me3-dependent regulation of the NOX4 promoter increased NOX4 expression and ROS generation. In turn, NOX4-derived ROS activated Wnt/β-catenin signaling, which further upregulated EZH2 expression, creating a self-reinforcing circuit [142]. This positive feedback circuit provides a conceptual framework in which the chromatin state (via PRC2/H3K27me3) programs NOX4 expression and ROS output. Moreover, NOX-dependent oxidative stress feeds back onto epigenetic regulators to reshape chromatin and gene expression programs. Recent studies published since 2023 have provided strong disease-related confirmation and further refined the NOX-EZH2-H3K27me3 axis. Espinosa-Sotelo and colleagues used CRISPR/Cas9 and biochemical approaches in hepatocellular carcinoma (HCC) cells to show that NOX4 is functionally involved in TGF-β-induced SMAD2/3 phosphorylation, cytoskeletal remodeling, growth arrest, and apoptosis. The CRISPR-Cas9-mediated knockdown of NOX4 shifts TGF-β signaling from tumor-suppressive toward protumorigenic programs [143]. A 2023 study published in *Cell Death Discovery* demonstrated that a decrease in EZH2 activity reduced H3K27me3 occupancy at gene promoters. This loss of repressive histone markers leads to the overactivation of genes involved in transcriptional regulation and apoptosis, triggering granulosa cell (GC) apoptosis. Notably, treatment with GSK-J4, an inhibitor of the H3K27me3-removing demethylase KDM6B, can partially attenuate GC apoptosis [144]. In 2024, Guo et al. defined a methylation-phosphorylation switch that can control EZH2 stability and catalytic activity [145]. Post-translational regulation of EZH2 modulates its stability and catalytic activity, thereby affecting H3K27me3 deposition. This provides a plausible biochemical link through which NOX-derived ROS and ROS-sensitive kinases may regulate PRC2 function and downstream epigenetic responses [145]. A study by Sánchez-Ceinos et al. (2024) in human aortic endothelial cells provided direct functional evidence that hyperglycemia enhances EZH2 recruitment and H3K27me3 enrichment at antioxidant gene loci, leading to the repression of antioxidant defenses and subsequent NOX4-dependent ROS accumulation and NF-κB activation [146]. Pharmacologic inhibition of EZH2 with GSK126 reversed these effects and reduced oxidative and inflammatory outputs. Collectively, these studies demonstrate that targeting EZH2 may attenuate oxidative stress and inflammation, thereby preventing vascular disease in diabetes [146]. Furthermore, redox-sensitive signaling and kinase activity can increase the expression of EZH2 and PRC2, whereas EZH2-mediated H3K27me3 programs reinforce NOX-driven oxidative stress and pathology in patients with vascular and hepatic diseases. Taken together, these findings underscore that the NOX-EZH2-H3K27me3 axis constitutes a redox-epigenetic feedback loop that is central to maintaining maladaptive transcriptional memory in the context of oxidative stress, fibrosis, and oncogenesis. By mutually reinforcing ROS generation and chromatin repression, this circuit contributes to phenotypic persistence even after the initial stimulus is removed. However, the dynamic reversibility of EZH2 methyltransferase activity and NOX-derived ROS also presents a therapeutic opportunity in which pharmacological modulation of either node may restore transcriptional plasticity and redox balance.

### NOX-Hippo pathway interaction: YAP/TAZ-driven NOX transcription and feedback

Recent studies have shown a dynamic, bidirectional interaction between NOX enzymes and the Hippo pathway, in which oxidant signals modulate YAP/TAZ activity [147, 148]. Reciprocally, YAP/TAZ-dependent transcription upregulates NOX expression or NOX-activating ligands, establishing feed-forward loops that reshape metabolism, ferroptosis sensitivity, and tissue remodeling. Additionally, mitochondria-derived superoxide activates YAP/TAZ by directly oxidizing RhoA, leading to PP2A-mediated dephosphorylation and inactivation of LATS1/2 kinases, thereby releasing YAP/TAZ from cytoplasmic sequestration. Complementarily, NOX4-derived H₂O₂ activates YAP/TAZ both through redox-mediated suppression of upstream Hippo kinase activity and through direct H₂O₂-dependent YAP protein stabilization, collectively establishing ROS as convergent activators of the YAP/TAZ transcriptional program [149, 150]. In our recent study, which investigated perfluorooctanoic acid (PFOA)-induced lung epithelial toxicity, we demonstrated that ROS accumulation following PFOA exposure disrupted core Hippo signaling components, including MST1/2 and LATS1, and promoted nuclear translocation and increased the expression of YAP/TAZ in both 2D and 3D lung epithelial models. Mechanistically, in this study we demonstrated oxidative stress induced by PFOA was associated with enhanced inflammatory signaling, mitochondrial dysfunction, and epigenetic dysregulation, whereas antioxidant intervention with N-acetylcysteine (NAC) partially restored Hippo pathway homeostasis and attenuated YAP/TAZ expression, supporting the role of ROS as critical upstream regulators of Hippo signaling during toxin-induced epithelial injury [151]. In parallel, a clear functional consequence of NOX -YAP signaling has been described in breast cancer, where NOX4 overexpression increases ROS levels, activates YAP, and drives glycolytic reprogramming and tumor cell proliferation and migration. Conversely, ROS scavenging with NAC, pharmacological YAP inhibition, or NOX1/4 blockade reversed these effects. These findings suggest that targeting the NOX4/ROS/YAP axis may offer a therapeutic strategy for inhibiting metabolic reprogramming in patients with breast cancer [148]. Together, these findings support a unified model in which ROS act upstream of the Hippo pathway to activate YAP/TAZ transcriptional programs under pathological stress, whereas NOX4-dependent ROS provide a sustained input that couples metabolic reprogramming to YAP-driven tumor and injury responses.

Hippo effectors can also function upstream of NOX, as exemplified by TAZ-driven transcriptional programs that regulate mediators of NOX activity. In an epithelial ovarian cancer model, Yang and colleagues defined the TAZ-ANGPTL4-NOX2 axis, in which TAZ directly upregulates angiopoietin-like 4 (ANGPTL4), leading to NOX2 activation, increased lipid peroxidation, and ferroptosis. Consequently, TAZ knockdown confers ferroptosis resistance, while constitutively active TAZ (TAZ-S89A) and ANGPTL4 overexpression sensitize cells to ferroptosis, highlighting a potential therapeutic opportunity in TAZ-activated ovarian tumors [152]. Consistent with these findings, recent work in cancer biology continues to underscore the functional consequences of TAZ-ANGPTL4-NOX signaling. ANGPTL4 promotes ovarian cancer progression via JAK/STAT signaling and proangiogenic remodeling of the tumor microenvironment, further underscoring the dual pro-ferroptotic and protumorigenic roles of this axis [153]. Mechanistically, similar axes (TAZ-EMP1-NOX4) function in renal cancer and ferroptosis models, indicating that YAP/TAZ/NOX link functions across tissues to synchronize the transcriptional state with redox homeostasis and ferroptosis susceptibility. Feed-forward NOX-YAP/TAZ loops amplify and sustain pro-oxidant cellular conditions: NOX-derived ROS sustain YAP/TAZ activation and their pro-growth and pro-ferroptotic transcriptional programs, whereas YAP/TAZ transcriptionally regulate NOX expression or NOX-activating factors that stabilize reactive species production. The downstream consequences include metabolic rewiring (enhanced glycolysis and altered NADPH utilization), changes in lipid peroxidation sensitivity, and modified interactions with immune and stromal cells. Recent translational work further supports this functional relevance. For instance, Xiong and colleagues demonstrated that NOX4 modulates metabolic reprogramming and antitumor immunity in breast cancer models [154], revealing that Hippo-NOX signaling exerts both cell-intrinsic effects and microenvironmental effects. To dissect pathway directionality in such systems, time series ROS measurements, YAP/TAZ localization assays, and transcriptional profiling of NOX isoforms and their YAP/TAZ targets should be combined with redox-insensitive mutants of upstream GTPases (e.g., RhoA) to block ROS-YAP signaling or with YAP/TAZ loss- or gain-of-function with NOX rescue experiments to assess epistasis. Collectively, these studies support a biologically significant feedback architecture that positions the Hippo signaling pathway as a central hub in redox biology and a promising therapeutic target in NOX-driven pathology.

### Lipid rafts as NOX localization hubs: toxicant-induced raft disruption

Cholesterol- and sphingolipid-enriched membrane microdomains, such as lipid rafts and caveolae, act as organizing platforms that concentrate receptors, scaffolds, and signaling enzymes [4]. For NOX enzymes, this membrane organization is critical, as toxicants that oxidize or deplete lipid rafts can disrupt NOX assembly and activation, leading to pathophysiological consequences [155–158]. Anagnostopoulou and colleagues (2020) investigated the partitioning of NOX isoforms among membrane microdomains in human vascular smooth muscle cells (VSMCs) and demonstrated that NOX1 and NOX5 preferentially localize to cholesterol-rich lipid rafts and caveolar fractions, whereas NOX4 is largely excluded from these membrane microdomains. This distinct subcellular compartmentalization indicated that lipid rafts function as specialized signaling platforms for specific NOX isoforms, influencing localized ROS generation and downstream redox signaling in human vascular cells. All together these findings provide mechanistic insights into how NOX isoform localization contributes to vascular oxidative stress and pathology [158]. These findings are consistent with studies showing that environmental and particulate toxicants trigger oxidative damage in rafts, consequently altering their composition and function [159–161]. In the nervous system, chronic exposure to traffic-related particulate matter induces oxidative stress, lipid peroxidation, and altered lipid-raft-associated processing of amyloid precursor protein (APP) in mouse cortical tissue and neuronal cell models. These effects are accompanied by increased expression of markers of oxidative and nitrosative stress, including 4-hydroxynonenal (4-HNE) and 3-nitrotyrosine (3-NT), and are significantly attenuated by the antioxidant N-acetylcysteine (NAC). This study quantified detergent-resistant membrane (lipid raft) fractions and demonstrated pollution-induced changes in APP/Aβ biochemistry within these domains, establishing lipid rafts as vulnerable targets of toxin-driven redox injury that contribute to neurodegenerative pathology [162]. Earlier studies in leukocytes, renal cells, and cancer cells demonstrated that cholesterol-rich lipid rafts are required for the proper assembly and activation of NADPH oxidase complexes and that cholesterol depletion redistributes NOX subunits out of raft fractions, impairing ROS generation. These effects are largely reversible upon cholesterol repletion [155, 163, 164].

**Fig. 4:**
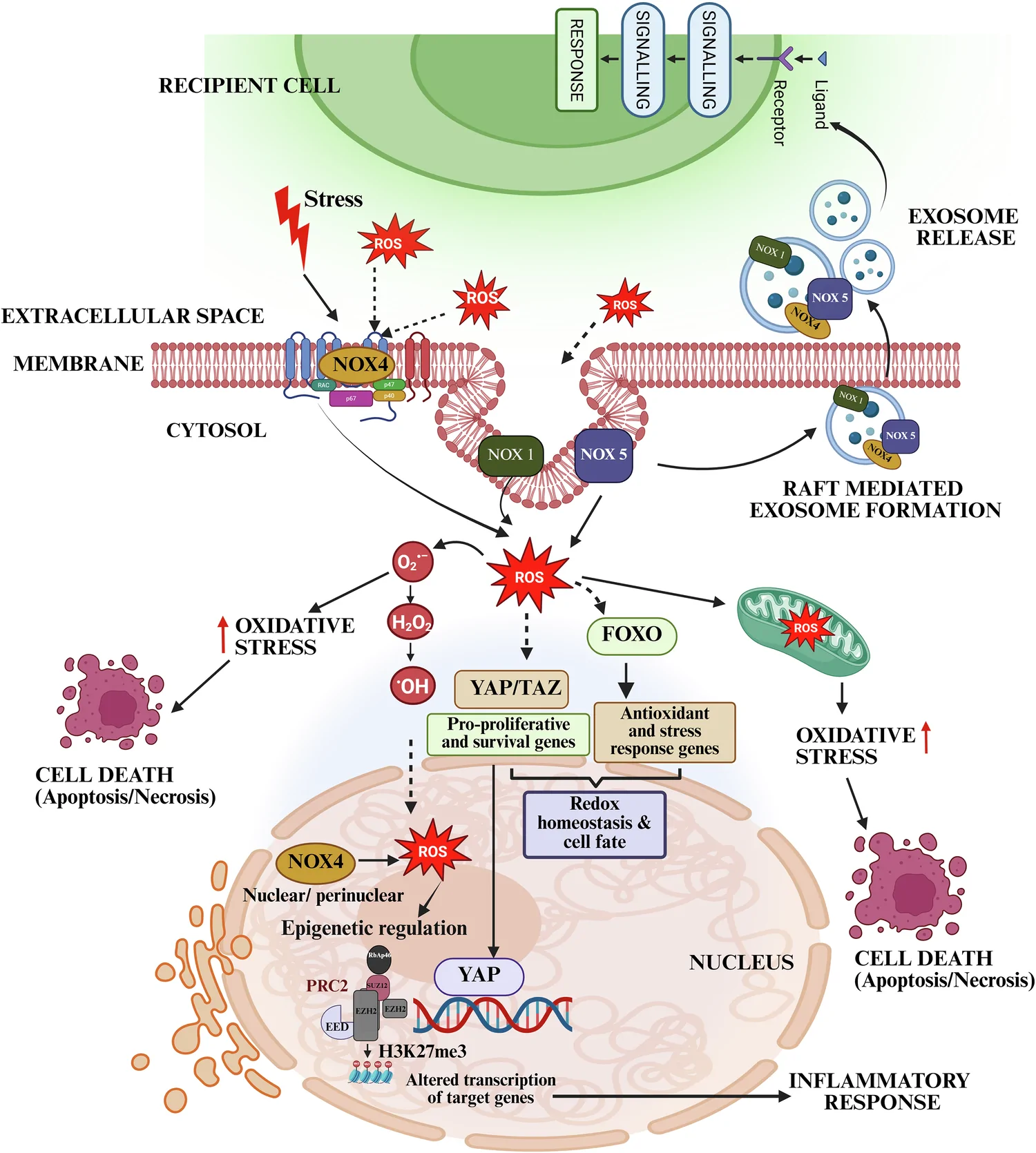
Plasma membrane and endosomal NOX isoforms coordinate membrane trafficking, intracellular signaling, and epigenetic reprogramming. NADPH oxidase (NOX) isoforms localized at the plasma membrane and in endosomal compartments generate spatially restricted reactive oxygen species (ROS) that regulate membrane trafficking, receptor signaling, and intracellular communication. Redox-dependent modulation of endocytosis, vesicular sorting, and signal transduction links NOX activity to stress-adaptive and inflammatory signaling pathways. In parallel, NOX-derived ROS intersect with the Hippo pathway effectors YAP and TAZ, promoting their activation and nuclear translocation, where they engage chromatin-modifying complexes such as polycomb repressive complex 2 (PRC2). Through PRC2-mediated epigenetic remodeling, NOX-YAP/TAZ signaling reprograms transcriptional networks that govern cell fate, inflammation, and tissue homeostasis under pathological conditions

Collectively, these findings establish lipid rafts as critical spatial determinants of NOX assembly, activity, and redox specificity. By governing the assembly and localization of oxidase complexes, rafts serve as redox gateways that translate membrane composition into signaling outcomes. How NOX isoforms are spatially organized within membrane and endosomal compartments is shown in Fig. 4, providing a structural basis for raft-dependent redox signaling and its disruption by toxicants. Disruption of raft integrity, whether by toxicants, oxidative stress, or lipid imbalance, reconfigures this signaling landscape, converting localized, protective ROS generation into diffuse oxidative injury.

### Exosomes and microvesicles as vehicles for NOX-dependent paracrine signaling

In recent years, extracellular vesicles (EVs), including exosomes and microvesicles, have emerged as potent mediators of redox communication. Beyond their role in intercellular signaling, EVs can carry enzymatically active NOX isoforms and NOX-regulating molecules that modulate oxidative and inflammatory tone in recipient cells. This mechanism enables localized redox perturbations to be transmitted across tissues, shaping endothelial integrity, immune responses, and tumor microenvironments. Recent research provides strong evidence for this model. The concept of EVs as biologically active molecules dates to the mid-20th century. Chargaff and West first described minute, lipid-rich vesicle-like particles in blood associated with coagulation and visualized them by electron microscopy [165]. Johnstone and coworkers later performed a detailed biochemical and ultrastructural analysis of vesicle release during reticulocyte maturation and reported that these vesicles exhibit plasma membrane-like functions in a regulated manner [166]. Although these early studies did not link exosomes to NOX-related biology, they established a foundation for understanding vesicle function under physiological conditions. The recognition of EVs as mediators of NOX-dependent redox signaling has emerged more recently [167].

A series of primary investigations has provided compelling evidence that EVs carry enzymatically active NOX isoforms that act as cargos of oxidative signaling between cells. In cancer-related studies, Gu and colleagues reported that cervical tumor-derived exosomes enriched in NOX1 subunits promote M2-like macrophage polarization, thereby promoting tumor progression and immune evasion [58]. Similarly, studies by Zheng and Zhao demonstrated that fibroblast-derived exosomes carrying NOX4 exacerbate oxidative stress and drive myofibroblast differentiation in models of pulmonary fibrosis [168, 169]. Silencing NOX4 expression or inhibiting exosome release with GW4869 significantly alters H_2_O_2_ levels and suppresses profibrotic transcriptional programs, illustrating a causal role for exosomal NOX in disease propagation. At the vascular interface, Tian and coworkers reported that endothelial cells exposed to cigarette smoke release NOX2-enriched microvesicles containing oxidized phospholipids, triggering mitochondrial dysfunction, barrier disruption, and increased permeability in endothelial monolayers [170]. Additional studies have further demonstrated how environmental stressors are mechanistically linked to endothelial oxidative injury through EV-mediated redox communication [171]. Mechanistically, exosomal NOX enzymes retain their membrane orientation and catalytic competence because of the vesicle lipid environment, which is rich in sphingomyelin and cholesterol, features reminiscent of membrane rafts. Circulating EVs in myocardial ischemia exhibit increased sphingomyelins under pathological stress [172]. Moreover, studies on membrane rafts have shown that lipid peroxidation disrupts both lipid packing and protein partitioning, indicating that raft integrity is essential for maintaining the correct membrane milieu [160]. EVs may therefore function as lipid-raft-derived platforms that preserve redox enzyme activity and propagate oxidative signaling [173]. This perspective reframes NOX biology as not only cell-restricted but also membrane-propagated and vesicle-borne, opening new opportunities to therapeutically target redox communication at its structural source. Collectively, the results of recent studies have established EV-associated NOX signaling as an integrative hub that connects redox signaling with transcriptional, mechanical, and membrane-associated regulation. Rather than acting as simple ROS generators, NOX enzymes participate in a complex regulatory network involving chromatin modifiers (EZH2/H3K27me3), mechanotransducers (Hippo-YAP/TAZ), lipid microdomains (rafts and caveolae), and extracellular vesicles (exosomes and microvesicles) [173–176]. Through these interactions, NOX-derived ROS influence epigenetic states, transcriptional programs, and intercellular communication, generating feedback circuits that sustain oxidative signaling and cellular phenotypes even after the initial stress is removed. The NOX-EZH2-H3K27me3 axis exemplifies a redox-epigenetic memory mechanism, while Hippo-YAP-TAZ coupling integrates oxidative and mechanical cues to drive metabolic and ferroptotic outcomes. Lipid rafts provide a spatial framework for NOX complex assembly and signaling precision but are vulnerable to disruption by toxicants, converting compartmentalized ROS signaling into diffuse oxidative injury. Furthermore, exosome-mediated export of NOX isoforms extends this influence across tissues, enabling paracrine propagation of redox signals that modulate vascular, immune, and fibrotic responses [56]. Together, emerging studies redefine NOX enzymes as central regulators of redox communication that extend beyond classical ROS generation. Rather than functioning as isolated oxidant sources, NOX enzymes are now understood as dynamic nodes that integrate chromatin architecture, mechanotransduction, membrane compartmentalization, and vesicular trafficking [142, 158, 177]. The convergence of these pathways reveals a multilayered network in which ROS act as both effectors and regulators of cellular structure and fate. These findings have significant implications for therapeutic targeting. Future therapeutic interventions must account for this contextual complexity and aim to restore redox signaling fidelity without disrupting the physiological crosstalk that underlies cellular adaptation and tissue homeostasis.

## Environmental and chemical pollutants as drivers of NOX dysregulation

Environmental and chemical pollutant exposure is increasing globally, and many converge on NADPH oxidases as central redox signaling. Persistent organic pollutants (POPs) such as PFOA and other PFASs, pentachlorophenol (PCP) and its metabolites tetrachlorohydroquinone (TCHQ) and hexachlorobenzene (HCB), and inhaled mixtures such as particulate matter, diesel exhaust, cigarette smoke, and ozone have been linked to elevated levels of reactive oxygen species in experimental and epidemiological studies. These exposures disturb the cellular redox balance, activate or dysregulate NOX isoforms, and drive oxidative stress that damages membranes, proteins, and DNA, often in parallel with mitochondrial dysfunction and inflammation. Over the past few years, mechanistic studies across lung, vascular, neural, hepatic, renal, and cutaneous models have strengthened the link between pollutant exposure and NOX2 or NOX4 activation as key proximal events in toxin-induced pathology. These organ-specific outcomes are consistent with the pollutant-NOX relationships summarized in Table 3.

**Table 3:** Environmental and chemical pollutants implicated in oxidative stress and redox-sensitive signaling pathways

Pollutant type	Pollutant	Redox mediators examined	Redox sensitive pathways/mechanisms	Biological/pathophysiological effects	References
Inhaled pollutants	Gasoline exhaust particle (GEPs)	NOX4-derived ROS, ATF3	ROS generation increases ATF3 dependent MMP-1 expression	Upregulation of MMP-1expression in HUVECs indicating matrix-remodeling response	[555]
	DEPs	ROS/oxidative stress	ROS-driven NF-κB activation; neutrophil chemotaxis signaling	Lung neutrophilic infiltration, airway inflammation, tissue damage (attenuated by progranulin derivative)	[556]
	DEPs	TLR2/TLR4 (ROS generation implicated)	TLR2/TLR4 inflammatory signaling; reduced claudin-5 and ZO-1	Disruption of blood-retina barrier integrity, vascular permeability, neuroinflammation	[557]
	1,8-Dinitropyrene (DNP) and metabolite 1-amino-8-nitropyrene	Indirect ROS contribution via AKRs, Nrf2	Aldo-keto reductases (AKR)-mediated nitroreduction; Nrf2 activation; redox cycling	Elevated ROS, oxidative DNA damage, and mutagenesis in human lung cells (A549 and HBEC3-KT cells)	[558]
	Ultrafine-DEPs	Iron accumulation; lipid peroxidation	ROS overproduction → lipid peroxidation → ferroptosis; mitigated by antioxidant α-lipoic acid	Ferroptotic neuronal death in substantia nigra pars compacta; Parkinson’s disease-like neurodegeneration	[559]
	PM_2.5_	ROS, NLRP3, eNOS	ROS → NLRP3 inflammasome activation → IL-1β release	Endothelial dysfunction, vascular inflammation, and increased cardiovascular risk	[560]
	Gasoline engine exhaust GEPM_2.5)_	Stress-responsive transcriptional programs	ROS signaling → altered transcriptional programs (inflammation, apoptosis, vascular remodeling genes)	Differential gene expression is linked to cardiovascular disease risk and endothelial dysfunction	[555, 561]
	PM_2.5_	ROS-sensitive MAPK/AP-1; AT1R	MAPK/AP-1 activation → AT1R upregulation	Endothelial dysfunction, vascular inflammation, and hypertension-related vascular injury	[562]
	PM2.5	NADPH oxidase activity; AKT/eNOS/NO	ROS-mediated activation of the AKT/eNOS/NO signaling pathway	Induced oxidative stress and inflammation in endothelial cells, leading to endothelial dysfunction	[181]
Occupational/ chemical pollutants	Rubber factory emissions (VOCs, PAHs, particulate matter)	ROS biomarkers	ROS generation, NF-κB activation, oxidative stress pathways	Elevated ROS levels in exposed workers, oxidative DNA damage, and increased risk of chronic respiratory and cardiovascular disease	[563]
	Carbon monoxide (CO)	NADPH oxidase activity	Inhibition of NADPH oxidase activity in macrophages and neutrophils; suppression of ROS generation; modulation of TLR4-associated inflammatory responses; inhibition of macrophage polarization toward M1 phenotype	Alleviation of endotoxin-induced acute lung injury; reduction in sepsis-related ARDS/ALI; suppression of oxidative stress and inflammation in lung tissues	[564]
	Inorganic nitrite (NO₂−)	NADPH oxidase-derived superoxide	Reduction of superoxide generation via nitric oxide (NO) production; inhibition of iNOS expression	Attenuation of oxidative stress in activated macrophages; potential modulation of inflammatory responses	[565]
Nanomaterials	Engineered ROS-modulating nanomaterials	Nanomaterial-dependent ROS	Modulation of ROS levels; alleviation of hypoxia; normalization of tumor pH; activation of immune effector cells; enhancement of antigen presentation	Reprogramming of tumor microenvironment; reduction of immunosuppression; increased T cell infiltration and activity; enhanced efficacy of immune checkpoint blockade therapy	[566]
	Fe (II)-PW₁₁ catalyst	NOX-mimetic catalytic activity	Catalytic ROS generation and ferroptosis	Induction of ferroptosis in tumor cells; selective toxicity to cancer cells; degradation into low-toxic debris in normal tissues	[567]
Persistent organic pollutants (POPS)	PCBs, PAHs, organochlorinated pesticides	ROS pathway	Activation of aryl hydrocarbon receptor (AhR); induction of cytochrome P450 enzymes (CYP1A1, CYP1A2, CYP1B1); oxidative stress; DNA damage; apoptosis	Neurodevelopmental disorders; endocrine disruption; increased apoptosis; DNA damage; oxidative stress	[568]
	Organophosphates, organochlorines, pyrethroids	NOX-derived ROS pathways	Pesticide-associated oxidative stress	Potential neurotoxicity	[569]
	Glyphosate (GLY)	NOX4; ROS; FOXO1	Increased expression of FOXO1 and NOX4 genes; induction of oxidative stress	Reduced granulosa-cell viability, Induction of apoptosis; DNA fragmentation; reduced expression of antioxidant genes (SOD, CAT, GPX); decreased expression of VNN1 and STAR genes	[570]

*AhR* Aryl hydrocarbon receptor, *CAT* catalase, *DEPs* diesel exhaust particles, *DNA* deoxyribonucleic acid, *FOXO1* forkhead box O1, *GEPs* gasoline exhaust particles, *GPM*_*2.5*_ gasoline engine exhaust PM_2.5_, *GPX* glutathione peroxidase, *IL-1β* interleukin-1 beta, *MAPK* mitogen-activated protein kinase, *NADPH* nicotinamide adenine dinucleotide phosphate, *NLRP3* NOD-, LRR- and pyrin domain-containing protein 3, *NOX* NADPH oxidase, *Nrf2* nuclear factor erythroid 2-related factor 2, *PAHs* polycyclic aromatic hydrocarbons, *PCBs* polychlorinated biphenyls, *PM*_*2.5*_ fine particulate matter ≤2.5 μm, *ROS* reactive oxygen species, *SOD* superoxide dismutase, *STAR* steroidogenic acute regulatory protein, *UF-DEPs* ultrafine diesel exhaust particles, *AKRs* aldo-keto reductases, *AKT* protein kinase B, *ALI* acute lung injury, *AP-1* activator protein 1, *ARDS* acute respiratory distress syndrome, *AT1R* angiotensin II type 1 receptor, *ATF3* activating transcription factor 3, *CO* carbon monoxide, *CYP1A1* cytochrome P450 family 1 subfamily A member 1, *CYP1A2* cytochrome P450 family 1 subfamily A member 2, *CYP1B1* cytochrome P450 family 1 subfamily B member 1, *DNP* 1,8-dinitropyrene, *eNOS* endothelial nitric oxide synthase, *GLY* glyphosate, *HUVECs* human umbilical vein endothelial cells, *iNOS* inducible nitric oxide synthase, *M1* classically activated macrophage phenotype, *MMP-1* matrix metalloproteinase-1, *NO* nitric oxide, *NOX4* NADPH oxidase 4, *POPs* persistent organic pollutants, *TLR2/TLR4* Toll-like receptors 2 and 4, *VNN1* vanin 1, *VOCs* volatile organic compounds, *ZO-1* zonula occludens-1

### Inhaled pollutants: PM_2.5_, diesel, cigarette smoke, ozone

Fine particulate matter (PM_2.5_) consists of complex mixtures of solid and liquid particles in the submicron size range that can reach distal airways and even systemic circulation. Chronic PM_2.5_ exposure is associated with chronic obstructive pulmonary disease (COPD), asthma, lung cancer, and cardiovascular disease. Xu and colleagues reviewed how PM_2.5_ disrupts epithelial barrier function and induces ROS generation and mitochondrial dysfunction in the airway epithelium [178]. Fan and colleagues demonstrated that PM_2.5_ exposure in bronchial epithelial cells (BEAS-2B) and in a cigarette smoke-induced COPD mouse model exacerbated oxidative stress and mitochondrial dysfunction through NOX4-Nrf2-mediated redox imbalance. Genetic or pharmacologic suppression of NOX4 expression restores antioxidant defenses, reduces mitophagy, and protects against acute exacerbation of COPD, thereby positioning NOX4 as a central driver of PM_2.5_-induced lung toxicity [179]. In COPD patients and in mice exposed to cigarette smoke, multiple NOX isoforms are induced in lung tissue [180]. Human COPD lungs exhibit increased expression of NOX1, NOX2, NOX4, and NOX5, whereas acute cigarette smoke exposure in mice induces NOX1, NOX2, and NOX4 expression. Additionally, knockdown of NOX1 or NOX4 reduces ROS generation and proinflammatory cytokine expression via suppression of the NF-κB/COX-2 axis. In contrast, NOX2 knockdown reduces ROS levels but paradoxically enhances NF-κB/COX-2 activation, highlighting isoform-specific roles in COPD pathogenesis [180]. The vascular endothelium is another sensitive target of airborne particles. In this context, Hu and coworkers reported that PM_2.5_ exposure in endothelial cells increases NOX1 and NOX4 expression, leading to excessive ROS generation and impairment of the AKT/Endothelial nitric oxide (eNOS)/NO signaling axis [181]. The inhibition of NOX activity with the dual NOX1/4 inhibitor GKT137831 restored AKT/eNOS activation, reduced oxidative damage, and attenuated inflammatory responses, indicating that the NOX-AKT/eNOS-NO pathway is a central mediator of PM_2.5_-induced vascular injury [181]. PM_2.5_ also reprograms NOX expression in the skin. Kang and colleagues reported that PM_2.5_ stimulates NOX1, NOX4, and DUOX1 expression in human keratinocytes through activation of the aryl hydrocarbon receptor (AhR), which binds to the promoters of NOX1 and DUOX1 [182]. PM_2.5_ increases the expression of NOX1, NOX4, and DUOX1 in human keratinocytes. The AhR-NOX axis mediates the epigenetic upregulation of NOX1 and DUOX1, with PM_2.5_-activated AhR acting as a transcription factor at their promoter regions; separately, NOX4 promotes its interaction with Ca²⁺ channels, leading to increased Ca²⁺ release and elevated ROS generation [182]. Together, these findings identify epigenetic and Ca²⁺ signaling as critical mechanisms of PM_2.5_-induced NADPH oxidase activation in keratinocytes. In HaCaT keratinocytes, Shin and coworkers reported that PM_2.5_ exposure upregulates NOX1 and NOX4 expression, driving ROS generation and triggering DNA damage and apoptosis [183]. Treatment with C-peptide markedly reduces PM_2.5_-induced apoptosis by suppressing NOX activity and intracellular ROS levels, suggesting that targeted NOX modulation can protect barrier tissues from particulate stress [183].

DPE (Diesel particulate extract) exposure in human keratinocytes caused increased activation of NADPH oxidases and subsequent ROS generation; blockade of NOX activation with a specific inhibitor attenuated DPE-induced keratinocyte apoptosis, supporting the conclusion that NADPH oxidase activation is a critical upstream event in DPE-induced keratinocyte death [184]. NOX-mediated keratinocyte apoptosis is attributable mainly to the neutral sphingomyelinase-mediated stimulation of ceramides, a well-known pro-apoptotic lipid, and the sequential pathway is DPE-NOX activation-neutral sphingomyelinase stimulation-ceramide accumulation-apoptosis [185]. Diesel exhaust particles can also induce NOX-dependent oxidative stress and cell death in the central nervous system. Kim and coworkers demonstrated that ultrafine diesel particles induce apoptosis in oligodendrocyte precursor cells by increasing intracellular ROS levels via NOX2 activation [186]. This is accompanied by the upregulation of p53, Bax, and cleaved-caspase-3 expression, linking NOX2-dependent oxidative stress to oligodendroglia and white matter vulnerability.

Chronic cigarette smoke (CS) exposure in mice upregulates the expression of CYBA (p22^*phox*^) and CYBB (NOX2) in the lung and vasculature, contributing to NADPH oxidase-driven superoxide production and oxidative stress [187, 188]. Treatment with the NOX inhibitor apocynin attenuates oxidative stress, slows COPD progression, and limits vascular injury, supporting a NOX2-dependent component in CS-induced pathology [189]. Complementing these findings, Li and colleagues reported that CS increases NOX4 expression in airway epithelial cells and in CS-induced COPD mouse models, activating NF-κB signaling and enhancing inflammation [190]. Treatment with luteolin, a flavonoid, suppresses NOX4-mediated NF-κB activation and reduces inflammatory cytokine production, further highlighting NOX4 as a druggable driver of CS-induced lung injury [191].

Recent work by Sul and coworkers revealed that cigarette smoke upregulates heme oxygenase-1 in an NOX-dependent manner and promotes Fe(II) accumulation and ferroptotic cell death. The inhibition or knockdown of NOX components reduces ROS generation, Fe(II) overload, and ferroptosis, directly linking NADPH oxidase activity to CS-induced ferroptotic injury [192]. Collectively, these studies support a model in which inhaled pollutants such as PM_2.5_, diesel exhaust, and cigarette smoke activate specific NOX isoforms in lung, vascular, cutaneous, and neural tissues. The resulting ROS amplify inflammatory, lipid, and ferroptotic pathways that shape disease progression in COPD, cardiovascular disease, neurotoxicity, and skin injury.

### Nanomaterials: silica, TiO₂, silver nanoparticles, and related materials

With respect to engineered nanomaterials, direct NOX-centric mechanistic studies remain relatively limited. However, studies of nanoparticles, nanomaterial-associated particulates, and mineral dust strongly support a central role for NOX signaling. Free crystalline silica dust provides a prototypical example. Recent work by Xiang and colleagues revealed that silicon dioxide (SiO₂) exposure in BEAS-2B cells and C57BL/6 mice increases NOX2 expression, ROS generation, JNK signaling activation, and epithelial-mesenchymal transition (EMT). Genetic deletion of NOX2 reduces ROS levels, blocks JNK activation, and attenuates EMT, indicating that NOX2 acts as an upstream driver of silica-induced fibrotic remodeling [193]. Similarly, Wang and colleagues reported that the use of titanium (Ti) wear nanoparticles in orthopedic implants promotes bone destruction through NOX4 activation in osteoclasts. Pharmacologic inhibition of NOX4 reduces intracellular ROS levels, suppresses excessive osteoclast differentiation, enhances Nrf2/HO-1 (heme oxygenase-1)/SOD2 antioxidant responses, and significantly protects mice from Ti nanoparticle-induced bone loss, indicating that NOX4 is a key mediator of titanium nanoparticle-driven osteoclastogenesis and osteolysis [194]. Silver nanoparticles (AgNPs) have also been linked to NADPH oxidase activation in the vasculature. A recent study by Sun and coworkers revealed that AgNP exposure increases NOX4 expression and ROS generation and mitochondrial dysfunction, disrupts endothelial barrier integrity, and activates p38 MAPK signaling in human venous endothelial cells [195]. NOX inhibition or NOX4 knockdown attenuated ROS generation and improved endothelial barrier function, indicating that the NOX4-Nrf2 redox imbalance is involved in AgNP-induced vascular toxicity [195].

### Persistent Organic Pollutants (POPs) and NOX activation

Evidence linking POP to NOX activation remains relatively fragmented and often indirect; however, several recent and foundational studies support a mechanistic link. PFOA exposure induces oxidative damage and mitochondrial dysfunction in cardiac tissue, as demonstrated in zebrafish and chicken embryo heart models, in which PFOA causes excessive ROS generation, lipid peroxidation, and suppression of antioxidant defenses, leading to cardiac malformations [196–199]. In hepatocellular models, PFOA initiates a vicious cycle between endoplasmic reticulum stress and oxidative stress, increasing the expression of GRP78, CHOP, and TXNIP while decreasing the mitochondrial membrane potential [200]. While many NADPH oxidase inhibitors, such as DPI, have been used in cardiac models to induce NOX activity in ROS-dependent injury, their lack of isoform specificity means that NOX involvement in PFOA-induced cardiac toxicity remains inferred rather than directly demonstrated, and specific NOX isoforms have not yet been characterized in this context [201–203]. Similarly, in pancreatic β-cells, PFOA exposure causes mitochondrial dysfunction and ROS generation, potential collapse of the mitochondrial membrane, and apoptosis in RIN-m5F cells. These effects are mitigated by NADPH oxidase inhibition, further supporting a NOX-dependent component in endocrine POP toxicity, even though NOX isoforms were not explicitly resolved [204]. More broadly, Elumalai et al. reported that perfluorooctane sulfonate (PFOS) induces oxidative stress and developmental toxicity in zebrafish larvae, which is consistent with redox and metabolic disruption, though direct in vivo evidence of NOX pathway upregulation by PFAS remains to be established [205].

Recent work in lung and liver cells has shown that PCP exposure increases the expression of multiple NADPH oxidase subunits (NCF1, NCF2, NOX2, and Rac), increases ROS levels, and activates the Hsp70-Beclin-1 autophagy axis [206]. These findings provide direct evidence that PCP modulates NOX2 signaling in mammalian cells. Previously, mechanistic studies of the PCP metabolite tetrachlorohydroquinone (TCHQ) have demonstrated strong ROS generation, redox cycling, and DNA damage and have identified TCHQ as a potent ROS-producing metabolite in pesticide-induced oxidative stress [114, 107]. However, the specific NOX isoforms involved in TCHQ-induced toxicity remain poorly characterized [114, 207]. Hexachlorobenzene (HCB) has also been linked to ROS-driven injury. Chiappini and coworkers reported that HCB induces ROS and ERK1/2 activation in FRTL-5 rat thyroid cells, leading to both caspase-dependent and caspase-independent apoptosis. Pharmacologic antioxidant interventions suggest that NADPH oxidase is a significant contributing source [208]. A more recent toxicology study in mice demonstrated that coadministration of thymol mitigated HCB-induced testicular damage by reducing ROS levels and restoring antioxidant defenses, underscoring the role of redox pathways, including NADPH oxidase, as key drivers of HCB toxicity [209]. In brief, POP studies indicate that PFOA and other PFAS, PCP and its metabolites, and HCB induce oxidative stress that is at least partly dependent on NADPH oxidase activity. However, isoform-specific attribution (for example, NOX2 versus NOX4) and detailed structural mechanisms remain underdeveloped for most POPs.

### Combined exposures, pathways, and epigenetic or mechanical alterations

Studies that formally dissect NOX activation under true combined-exposure conditions remain relatively rare, but several patterns are emerging. Early studies in aquatic models demonstrated that combined xenobiotic and salinity stress amplifies oxidative responses and alters NADPH oxidase activity in immune cells, suggesting that coexposure can nonlinearly modulate NOX-dependent defense and cell-death pathways [210–212]. In mammalian systems, diverse classes of pollutants converge on common signaling hubs, such as collagen α1(I) and TGF-β, where NOX-derived ROS act as upstream regulators of proinflammatory and profibrotic programs [213].

The epigenetic and mechanical consequences of pollutant-driven NOX activation are being increasingly recognized. In this context, Kang and coworkers linked the PM_2.5_-induced upregulation of NOX1 and DUOX1 in keratinocytes to AhR-dependent promoter binding and associated chromatin remodeling, providing a clear example of the pollutant-induced epigenetic regulation of NOX expression [182]. On the metabolic side, Mustieles and colleagues described how mixtures of POPs reshape the redox microenvironment and lipid metabolism in adipose tissue, increasing ROS generation and promoting metabolic dysfunction and cancer risk [214]. They identified NADPH oxidase as a plausible upstream mediator that links environmental exposure to obesity and metabolic disease [182, 214].

Pollutants that alter cytoskeletal organization or extracellular matrix stiffness can increase integrin- and growth factor-mediated signaling. In many contexts, these pathways are known to activate NOX1 and NOX4 in vascular cells and fibroblasts, thereby altering redox homeostasis and TGF-β-driven remodeling [215, 216]. Although explicit pollutant-mechanotransduction-NOX signaling pathways are only beginning to be mapped, current evidence supports the positioning of NOX at the intersection of mechanical and chemical stress in tissues exposed to chronic environmental insults.

Overall, the emerging body of work indicates that NOX enzymes function as central redox mediators and amplifiers of environmental and chemical pollutant responses. Inhaled toxicants such as PM_2.5_, diesel exhaust, and cigarette smoke strongly support the involvement of NOX1, NOX2, and NOX4, affecting lung, vascular, skin, and neural tissues. NOX activation associated with nanomaterials is best characterized for silica, titanium particles, and silver nanoparticles, whereas POP-related NOX mechanisms remain less defined, especially for isoform specificity, although accumulating evidence suggests convergence on NOX2 and NOX4 [217]. Key knowledge gaps include pollutant exposure combinations, precise isoform mapping, and the epigenetic and mechanotransduction pathways through which pollutants reprogram NOX responses. Future research should integrate multiomics approaches, single-cell imaging, and real-world pollutant mixtures to better understand how environmental stress reshapes NOX biology across tissues.

## Organ-specific roles and disease pathologies

Understanding the organ-specific roles of NADPH oxidases in disease development and progression is essential for elucidating their contributions to human pathology and therapeutic responses. Sustained oxidative stress is central to the development and progression of many chronic diseases [218]. In addition to the electron transport chain system, the NADPH oxidase family of enzymes represents a major source of reactive oxygen species involved in cell signaling, host defense, and the maintenance of redox homeostasis [62, 219]. However, an imbalance between ROS generation and antioxidant defenses can lead to the development of many pathologies, including cardiovascular disease, diabetes, neurodegeneration, chronic lung disease, and cancer [220, 221]. In the following subsections, we discuss emerging findings on the roles of NOX family enzymes in the development and progression of various diseases.

### Lung: roles of NADPH oxidase in pulmonary health

Under normal conditions in a healthy lung, NOX isoforms support pulmonary homeostasis. NOX2 (gp91^*phox*^) in resident and recruited phagocytes generates superoxide for antimicrobial killing, whereas DUOX1/2 in airway epithelia maintains epithelial barrier integrity and orchestrates mucociliary clearance [63, 222, 223]. NOX4, expressed predominantly in mesenchymal and vascular cells, promotes TGF-β and NF-κB signaling and regulates other redox-sensitive pathways, thereby influencing smooth muscle tone, matrix production, and barrier function [224]. Despite the important roles these enzymes play in maintaining normal lung function, chronic overactivation or mislocalization drives oxidative stress, inflammation, and fibrosis, which underpin major lung diseases such as COPD, IPF, and acute lung injury (ALI), often in synergy with environmental pollutants [180, 225, 226].

#### Chronic Obstructive Pulmonary Disease (COPD)

COPD is one of the most prevalent pollutant-associated chronic lung disease and is currently the third leading cause of death worldwide. It is projected to claim approximately 4.4 million lives annually by 2040 [227]. COPD is characterized by persistent airflow limitation resulting from emphysema, small-airway remodeling, chronic bronchitis, and tissue destruction, typically due to prolonged exposure to harmful substances [227]. Patients with advanced COPD exhibit elevated expression of NOX1, NOX2, NOX4, and NOX5 in bronchial and alveolar epithelium, vascular endothelium, and inflammatory cells, indicating broad engagement of NOX enzymes in advanced disease [180]. Consistent with the central role of cigarette smoke in COPD pathogenesis, experimental subchronic cigarette smoke exposure induces pronounced pulmonary inflammation characterized by macrophage and neutrophil recruitment and enhanced NF-κB-dependent inflammatory signaling [228]. Altered expression and activation of multiple NOX isoforms have been reported in bronchial and alveolar tissues, vascular endothelium, and inflammatory cells during chronic lung disease, indicating broad engagement of NOX enzymes in disease progression [6, 180, 190, 229]. Collectively, these findings support NOX1/4-NF-κB signaling as a key driver of early smoke-induced pulmonary inflammation and injury. In addition to catalytic subunits, organizer proteins play critical roles. In smoke-exposed lungs, increased expression and activation of the regulatory NADPH oxidase subunit NOXO1 enhance nonphagocytic ROS production contributing to oxidative stress, emphysema development, and chronic lung injury, highlighting NOXO1 as a promising therapeutic target in COPD [180, 189, 230]. Seimetz and colleagues demonstrated that NOXO1-null mice had significantly preserved lung structure and were less susceptible to smoke-induced emphysema, pulmonary hypertension, peroxynitrite formation, and elastin degradation, underscoring the role of NOXO1 in the development of lung disease [230]. Pharmacologic and nutraceutical studies further support the therapeutic potential of targeting NOX in COPD. For instance, the isoflavone puerarin mediates NOX1/4 downregulation, restores PTEN/Akt signaling, and attenuates NF-κB-p65 nuclear translocation and COX-2/IL-6 expression in CS-exposed mice and human small-airway epithelial cells (HSAECs) [229]. These findings demonstrate that puerarin suppresses NOX isoform-dependent ROS generation and inflammation in CS-exposed mice [229]. Similarly, luteolin has been shown to target NOX4-NF-κB signaling in cigarette smoke-exposed COPD models, reducing MDA levels, restoring GSH levels, and decreasing TNF-α/IL-1β production, indicating the alleviation of oxidative stress [191]. In both cases, targeting NOX activity alleviated oxidative stress. Collectively, these studies highlight the central role of NOXs in COPD pathogenesis and the therapeutic potential of NOX inhibition.

Beyond cigarette smoke, other inhaled environmental pollutants, such as PFAS compounds, likely engage overlapping NOX-dependent oxidative pathways in the lung. While direct evidence linking PFOA to specific NOX isoform activation in the lung remains limited, exposure to PFOA and other PFASs has been shown to induce oxidative stress, epithelial injury, and inflammatory responses in respiratory and cardiovascular models, often with features reminiscent of CS-induced NOX activation. Given that CS-mediated epithelial damage is driven by NOX-dependent ROS overproduction, it is plausible that PFAS may similarly compromise epithelial barrier integrity through NOX-associated mechanisms.

#### Idiopathic pulmonary fibrosis (IPF)

IPF is a progressive interstitial lung disease characterized by the accumulation of extracellular matrix proteins, the aberrant recruitment of fibroblasts and myofibroblasts, and the distortion of alveolar architecture, which ultimately impairs gas exchange, leading to respiratory failure [231]. Among the NOX isoforms, NOX4 has emerged as a central mediator of fibroblast activation and pulmonary fibrosis. TGF-β upregulates NOX4 expression in primary human lung fibroblasts, and NOX4-derived H_2_O_2_ sustains Smad2/3 phosphorylation and α-SMA expression [232]. Consistent with this, Sato and colleagues reported that NOX4 plays a crucial role in IPF pathogenesis by modulating TGF-β-induced myofibroblast differentiation, as evidenced by its increased expression in the lungs of IPF patients [233]. Bleomycin-induced fibrosis models further support the profibrotic role of NOX4 [215]. Knockdown or genetic deletion of NOX4 reduces fibroblast senescence, decreases collagen I/III deposition, and decreases senescence-associated β-galactosidase activity in fibroblasts, underscoring its contribution to fibrotic remodeling [12]. Dual NOX1/4 blockade with setanaxib (GKT137831) reduces TGF-β-induced H₂O₂ production, fibroblast differentiation, and profibrotic gene expression in human lung fibroblasts. Moreover, setanaxib attenuates established fibrosis in precision-cut lung slices and reduces epithelial-to-mesenchymal transition without toxicity, supporting its progression to clinical testing for IPF [12]. DUOX1 complements these mechanisms in epithelial-driven fibrosis by amplifying ERK1/2 signaling for persistent TGF-β autocrine signaling. Silencing DUOX1 in organoid and spheroid models reduces fibrotic epithelial remodeling, suggesting that epithelial and mesenchymal NOX activities cooperate to maintain fibrotic progression [234].

#### Acute lung injury (ALI)

ALI pathogenesis is strongly driven by oxidative stress, with NOX enzymes serving as major sources of ROS that contribute to disease progression. ALI is characterized by acute inflammation and increased alveolar capillary barrier permeability, resulting in pulmonary edema [235]. Hyperoxia increases NOX4 expression in mouse lung tissue and in type II epithelial cells (MLE-12). This upregulation promotes mitochondrial ROS generation and epithelial apoptosis, effects that are mitigated by NOX4 silencing [236]. Similarly, NOX4 KO preserves alveolar architecture, significantly reducing TUNEL-positive cells and bronchoalveolar lavage (BAL) protein levels [236]. These findings identify epithelial NOX4 as a major source of hyperoxia-induced ROS and cell death in the alveolar epithelium. In COVID-19-associated ALI, the SARS-CoV-2 spike protein activates endothelial NOX2 via the ACE2-Ang II axis, impairing mitochondrial function and increasing thromboxane and IL-6 production, thereby contributing to coagulopathy and respiratory failure [237]. In addition, NOX2-mediated neutrophil extracellular traps exacerbate hypoxemia and promote microvascular occlusion in severe viral pneumonia; however, inhibition of gp91^*phox*^ is associated with a risk of immunosuppression [111]. CSE exposure in bronchial epithelial cells (BEAS-2B) induces the oxidation of PTEN at Cys71/124 via DUOX1/2-derived ROS, leading to the inhibition of Akt signaling, increased cell survival signaling and IL-8 production. This mechanism may also contribute to viral-bacterial coinfections and chronic airway inflammation [238]. Broader inhaled exposures, such as organic dust, further implicate NOX and xanthine oxidase crosstalk in NF-κB-driven mucus metaplasia and epithelial barrier dysfunction [239].

### Brain: roles of NADPH oxidases in neurological health and pathology

In the central nervous system (CNS), NOXs exhibit cell type-specific expression patterns that support both neuroprotective signaling and pathological oxidative damage. NOX2 is predominantly expressed in microglia and, to a lesser extent, in neurons because of rapid ROS bursts during immune responses and synaptic plasticity [240]. NOX4 is constitutively active in astrocytes and endothelial cells, generating H₂O₂ for redox modulation of signaling pathways involved in HIF-1α signaling, brain-derived neurotrophic factor (BDNF) signaling, glucose transport, and neurotrophic factor expression [241–243]. Physiologically, low-level, compartmentalized NOX-derived ROS generation facilitates neuronal migration, dendritic spine formation, and blood-brain barrier (BBB) maintenance [244]. However, in disease states, pathological activation and upregulation of NOX isoforms triggered by Aβ oligomers, α-synuclein aggregates, or ischemic insults amplify superoxide and H₂O₂, promoting lipid peroxidation, protein carbonylation, DNA damage, and ferroptosis, which exacerbate neurodegeneration, inflammation, and vascular permeability [245–247]. Endothelial and neuronal NOX4, in particular, mediate BBB breakdown and autotoxic neuronal death after ischemic stroke, and NOX4 induction has been reported in the hippocampus, cortex, substantia nigra, and cerebellum across multiple neurodegenerative disease models [248]. In parallel, NOX2-driven microglial activation sustains NF-κB and NLRP3 inflammasome signaling, linking chronic oxidative stress to neuroinflammation and progressive neuronal loss [249]. Airborne pollutants such as PM_2.5_ activate the NOX2 enzyme complex in microglia, leading to increased ROS generation and the activation of multiple proinflammatory pathways, including TLR4/Syk/PLCγ/PKC signaling, the NF-κB pathway, the NLRP3 inflammasome, and the PI3K/Akt pathway [117, 243, 250, 251]. The following subsections examine how individual NOX isoforms contribute to specific neurological disorders.

#### NOX isoforms in neurodegenerative diseases

Alzheimer’s disease (AD): AD is a progressive neurodegenerative disorder characterized by amyloid-β (Aβ)-induced oxidative stress and tau hyperphosphorylation, with NOX enzymes amplifying microglial activation and neuronal apoptosis [247]. NOX2, expressed in microglia, is rapidly activated by Aβ oligomers and protofibrils, generating superoxide that activates the NLRP3 inflammasome and promotes the release of IL-1β, thereby perpetuating a vicious cycle of neuroinflammation in aging and AD models [251]. NOX4 is upregulated in astrocytes and neurons. In support of this observation, the integration of AD transcriptomic datasets with ferroptosis-related gene signatures identified NOX4 as a central ferroptosis-associated gene in astrocytes and revealed that its silencing in vitro mitigates these effects [252, 253]. Since ferroptosis is mechanistically driven by iron-dependent lipid peroxidation, iron dysregulation represents a critical downstream consequence of NOX4-driven oxidative stress, which is further linked to synaptic loss in AD. Consistent with this, NOX4 inhibition by GKT137831 reduces Aβ-induced astrocyte ferroptosis and preserves hippocampal LTP in vitro, and NOX4 silencing improves AD pathology in APP/PS1 transgenic mice [252]. Emerging evidence also links NOX5 to neuronal calcium dysregulation in sporadic AD, with Ca²⁺-dependent superoxide bursts promoting tau aggregation [254].

#### Parkinson’s disease (PD) and α-synucleinopathies

PD is characterized by progressive loss of dopaminergic neurons in the substantia nigra and misfolding of the protein α-synuclein, resulting in motor dysfunction [255]. Both NOX2 and NOX4 have been shown to drive mitochondrial ROS generation and α-synuclein fibrillization, thereby promoting neuroinflammation and neuronal cell death [256]. In coculture systems and α-syn-preformed fibril models, misfolded alpha-synuclein (α-syn) engages microglial receptors and triggers NADPH-dependent superoxide release, resulting in the degradation of dopaminergic neurons [257]. NOX2 inhibition with apocynin reduces nigral neuron loss and relieves motor deficits via NRF2 activation [258]. NOX4 plays a complementary and, in some respects, broader role in PD-associated oxidative injury. It exacerbates PD pathology by promoting astrocyte-derived lipid peroxidation, resulting in astrocyte ferroptosis [242]. In 2025, Lin and colleagues demonstrated a connection between NOX4 and PD in a mouse model, reporting that NOX4 inhibition alleviated neuroinflammation and dopaminergic neuron loss and reduced ferroptosis and iron accumulation [242]. Additionally, elevated levels of NOX4 in the hippocampus of MPTP-induced PD mice drive the expression of inflammatory mediators, including myeloperoxidase (MPO) and osteopontin (OPN), contributing to mitochondrial dysfunction and lipid peroxidation in the hippocampal astrocytes of a PD mouse model and ultimately leading to ferroptosis [259]. This study further revealed that increased MPO and OPN expression during PD is NOX4-dependent, reinforcing its detrimental role in disease progression. Consistent with these findings, selective NOX4 inhibition in MPTP mice reduces iron accumulation and lipid peroxidation in the substantia nigra, preserves dopaminergic neurons, dampens neuroinflammatory cytokines, and improves motor performance, identifying NOX4 as a central effector of ferroptosis and neurodegeneration in PD models [242]. Taken together, these studies support a model in which microglial NOX2 links extracellular α-synuclein to neuroinflammatory oxidative stress, whereas neuronal and astrocytic NOX4 exacerbates mitochondrial dysfunction, iron-dependent lipid peroxidation, and ferroptosis. This coordinated NOX2-NOX4 activation helps explain how α-syn pathology, inflammation and oxidative injury are intertwined in PD and highlights isoform-selective NOX inhibition as a promising strategy to slow dopaminergic neuron loss and disease progression.

#### Microglial activation and NOX2/4 in neuroinflammation

Through the activation of redox-sensitive signaling pathways and enhanced ROS generation, NOX2 renders microglia more responsive to inflammatory triggers and enhances their production of neurotoxic cytokines; when this state is sustained, it drives chronic, self-perpetuating neuroinflammation and neuronal injury [260]. Recent studies have identified several receptor-NOX2 signaling axes that regulate microglial activation. In this context, complement receptor 3 (CR3) signaling has been shown to upregulate NOX2 in microglia, thereby promoting neuronal injury and apoptosis in neurodegenerative disorders, including Parkinson’s disease. Silencing CR3 or inhibiting the CD11b-NOX2 axis suppresses exosome-mediated toxicity and preserves synaptic integrity [261]. In microglia, NOX4 regulates inflammation, at least in part through H₂O₂-mediated Nrf2 stabilization. However, when this response exceeds cellular capacity, cells shift to a pro-inflammatory state [262]. In mixed glial and microglial cultures, exposure to LPS upregulates NOX4 alongside NLRP3; pharmacological NOX4 inhibition (GKT136901) or NOX4 KO reduces IL-1β release and normalizes NLRP3 expression, indicating that NOX4-derived ROS fine-tune inflammasome activity and influence cognitive outcomes in systemic inflammation models [262]. In LPS-exposed BV2 microglia, NOX4 siRNA reduces IL-6 release, further supporting a proinflammatory role for NOX4-derived ROS [125, 263]. A recent study revealed that inhibiting the CD11b-NOX2 axis, which is responsible for neuroinflammation, oxidative stress, and nerve damage, attenuates microglial activation and neurodegeneration [264]. In amyotrophic lateral sclerosis (ALS), SOD1-G93A microglia exhibit hyperactive NOX2, leading to motor neurotoxicity driven by oxidative stress from inflammation and ROS generation [265].

#### NOX in stroke, ischemia, and vascular pathologies

Ischemic stroke causes rapid activation of NOX isoforms, which triggers a cascade of oxidative stress and inflammation that contributes to BBB disruption and neuronal cell death [266]. In cerebral ischemia-reperfusion models, NOX2 and NOX4 are consistently upregulated in neurons, endothelial cells, and glia, and their suppression limits infarct size, BBB leakage, and apoptotic signaling [248, 267]. Vitamin D₃ pretreatment has provided mechanistic evidence supporting the therapeutic targeting of NOX-dependent signaling pathways. In a global ischemia model in Mongolian gerbils, transient ischemia-reperfusion increased the expression of oxidative stress markers and upregulated the expression and activity of NOX2 subunits and MMP-9, as well as changes associated with BBB breakdown and tissue edema. NOX2 promotes secondary injury by facilitating NETosis in infiltrating neutrophils and activating proinflammatory responses in perivascular macrophages [268]. This exacerbates microvascular occlusion, hemorrhagic transformation, and delayed cognitive deficits, whereas pharmacological inhibition of NOX2 with VAS2870 mitigates hemorrhagic transformation and cognitive impairment [269]. Chronic cerebral hypoperfusion induces white matter rarefaction through oxidative stress generated by dysregulated NOX1/4-NOS crosstalk. Dual NOX1/4 inhibition reverses these effects, restores oligodendrocyte myelination, and improves behavioral outcomes [270]. Collectively, these studies identify NOX2 and NOX4 as pivotal mediators of both acute ischemic stroke and chronic hypoperfusion-related injury, operating at the interface of endothelial dysfunction, BBB breakdown, and neurovascular inflammation, thereby making them attractive yet challenging therapeutic targets.

#### Amyotrophic lateral sclerosis (ALS)

Microglial NOX2 is a major source of ROS in amyotrophic lateral sclerosis (ALS) and contributes to progressive motor neuron degeneration. Experimental studies in SOD1-G93A-mutant ALS mice have demonstrated that genetic deletion or pharmacological inhibition of NOX2 slows disease progression and reduces motor neuron loss, supporting a pathogenic role of microglia-derived ROS [271, 272]. Riluzole, an FDA-approved therapy for ALS with a modest survival benefit, not only reduces glutamatergic excitotoxicity but also demonstrates antioxidant properties by attenuating ROS-mediated injury in neuronal cell models [273]. Furthermore, recent studies using cerebral organoid systems have shown that pathogenic TDP-43 can be seeded and propagated in human C9orf72 (Chromosome 9 Open Reading Frame 72)-derived cerebral organoids, reinforcing the link between oxidative stress, microglial activation, and protein aggregation [274]. However, direct evidence linking NOX2 activity to the initiation or propagation of TDP-43 aggregation in these models remains limited. Overall, the ALS literature supports a model in which NOX2 acts as a redox amplifier in microglia and other glial cells, transforming an initial genetic insult (mutant SOD1, TDP-43, C9orf72) into chronic oxidative stress, neuroinflammation, and progressive motor neuron degeneration.

#### NOX in other brain pathologies: MS, epilepsy, and beyond

In multiple sclerosis, NOX2 is upregulated in inflammatory lesions and contributes to demyelination and axonal damage [275]. NOX2-deficient mice exhibit reduced experimental autoimmune encephalomyelitis (EAE) severity and preserved myelin, accompanied by diminished Th17 cell infiltration into the CNS. In experimental epilepsy models, NOX2 activity contributes to seizure-associated ROS generation, mitochondrial dysfunction, neuronal injury, and epileptiform activity. In a kainic acid-induced status epilepticus model, selective NOX2 inhibition with gp91ds-tat reduced NOX2 expression, overall NOX activity, oxidative stress, and seizure burden, supporting a pathogenic role for NOX2 in epilepsy progression [276]. Pharmacological inhibition of NOX2 with gp91ds-tat or GSK2795039 reduces Ca²⁺ oscillations, ROS generation, and neuronal death while significantly decreasing seizure frequency and severity [277]. In epileptic rat models, compared with vehicle or scrambled peptide controls, gp91ds-tat reduces the frequency of spontaneous recurrent seizures [276]. These findings reinforce a unifying theme across ALS, MS/EAE, and epilepsy: NOX2-mediated ROS in microglia and other CNS cells act as key amplifiers of neuroinflammation and oxidative injury, whereas isoform-selective NOX2 inhibition represents a promising, mechanism-based strategy to complement existing disease-modifying and symptomatic therapies.

### Heart: role of NADPH oxidases in cardiovascular health and disease

NOXs exhibit isoform-specific expression and function in the cardiovascular system, balancing redox signaling with pathological oxidative stress. NOX2 and NOX4 are predominantly expressed in cardiomyocytes and are responsible for acute ROS bursts that support contractility and vasoregulation [278, 279]. NOX4 constitutively generates relatively low levels of H₂O₂ in fibroblasts and endothelial cells, modulating hypertrophy and angiogenesis [280]. NOX1 is enriched in vascular smooth muscle and contributes to proliferative and migratory responses, whereas NOX5 modulates Ca²⁺-activated ROS generation in coronary endothelium and vascular smooth muscle, influencing vascular tone and remodeling under stress conditions [281, 282]. Under physiological conditions, NOX4-derived ROS serve important signaling functions by promoting eNOS activity, preserving vascular homeostasis, and supporting adaptive mitochondrial responses during acute exercise [283, 284]. In pathological states, however, overactivation of this pathway by Ang II, hyperlipidemia or hyperglycemia leads to superoxide-mediated eNOS uncoupling, lipid peroxidation, and inflammasome activation, promoting endothelial dysfunction, plaque instability, and maladaptive remodeling [93, 101–104, 285, 286]. In this section, the effects of NOX isoforms on atherosclerosis, hypertension, heart failure (HF), and ischemia-reperfusion (IR) injury are discussed.

#### NOX isoforms in atherosclerosis and endothelial ROS signaling

Endothelial dysfunction acts as the primary trigger for the initiation of atherosclerosis. Disturbed shear stress and oxidized LDL activate NOX1, NOX2, and NOX5 in endothelial cells and vascular smooth muscle. This increases superoxide production, promotes LDL oxidation, and upregulates VCAM-1/ICAM-1 expression, monocyte recruitment, and plaque formation [287, 288]. In ApoE^⁻/⁻^ mice, genetic deletion of NOX2 reduces the aortic lesion area and improves the endothelial ROS/NO balance, which is consistent with a proatherogenic role for these isoforms [289]. Selective pharmacological inhibition of NOX2 with GSK2795039 in a vulnerable-carotid plaque model stabilized established lesions by decreasing necrotic core formation, fibrous cap thinning, intraplaque hemorrhage, and oxidative stress [290]. It also enhances macrophage efferocytosis via the MerTK/PI3K/AKT axis, highlighting the central role of NOX2 in atherosclerosis pathogenesis. Furthermore, endothelial-specific NOX2 overexpression in ApoE^-/-^ mice increases vascular superoxide production, upregulates VCAM-1 expression, and promotes macrophage recruitment during early lesion formation, supporting a role for endothelial NOX2 in the initiation of atherosclerosis [291]. In contrast, NOX4 has been reported to exert vasoprotective effects through H₂O₂ stabilization, flow-mediated dilation, and maintenance of endothelial function [292]. Endothelial-specific NOX4 overexpression attenuated oxLDL-induced senescence and eNOS uncoupling in vitro, whereas GKT137831 (a dual NOX1/4 inhibitor) reduced aortic plaque size in hyperlipidemic mouse models, in part by decreasing the 4-HNE adduct [293–296]. NOX5, a uniquely Ca²⁺-activated isoform, amplifies ROS generation in the human coronary endothelium under oscillatory shear stress, promoting calcification. Silencing NOX5 in human vascular smooth muscle cells (hVSMCs) attenuated both migration and osteogenic differentiation, linking NOX5 to advanced plaque instability and further supporting its role in disease progression [297, 298].

#### NOX in hypertension and vascular remodeling

Hypertension is characterized by NOX-mediated vascular stiffening and endothelial dysfunction, with Ang II stimulating NOX1 and NOX2 in endothelial cells to generate superoxide, thereby reducing NO bioavailability and increasing the expression of ET-1 and TP receptors [278, 285, 299]. In a study using a mouse model, NOX2 was shown to play a pivotal role in the development of Ang II-induced vascular remodeling and hypertension through the paracrine release of growth differentiation factor 6 (GDF6), which promotes vascular smooth muscle cell proliferation [300]. In Dahl salt-sensitive (SS) rats, compared with control rats, NOX4 KO rats have lower blood pressure, as reduced NOX4 activity decreases renal H₂O₂ levels and epithelial sodium channel (ENaC) activity, modulating vascular tone [301]. However, with prolonged salt intake, SS rats develop albuminuria and sodium retention, highlighting that NOX4 exerts both protective and maladaptive effects depending on context and duration [301]. NOX5 has been linked to endothelial NOS uncoupling and impaired vasodilation in humans, and in VSMCs, NOX5 activation produces O₂^•−^ and H₂O₂, which lead to Ang II-induced oxidative stress and vascular dysfunction; notably, NOX5 silencing restores cellular function. NOX4 in PASMCs promotes hypoxia-induced proliferation via HIF-1α stabilization, further supporting the role of NOX4 in pulmonary vascular remodeling associated with pulmonary hypertension [302].

#### NOX in heart failure and cardiac hypertrophy

In heart failure, maladaptive NOX activation contributes to cardiomyocyte death through apoptosis, chronic inflammation, fibrotic remodeling, and ultimately diastolic dysfunction. In mouse models, NOX2 in infiltrating macrophages promotes mitochondrial dysfunction, apoptosis, and adverse remodeling [303]. Selective inhibition of NOX2 with GSK2795039 reduces ROS generation and NOX2 expression, limits cardiomyocyte apoptosis and infarct size, increases the ejection fraction, and preserves mitochondrial respiratory chain function, demonstrating a direct cardioprotective effect of NOX2 blockade [304, 305]. Similar benefits of GSK2795039 have been reported in doxorubicin-induced cardiomyopathy, where NOX2 inhibition attenuates oxidative stress, sympathetic nerve-terminal abnormalities, and myocyte autophagy, further implicating NOX2 in chemotherapy-related heart failure [120]. NOX4 is upregulated in cardiac hypertrophy and failure, where pressure overload increases NOX4 expression in cardiomyocytes, leading to RyR2 oxidation, Ca²⁺ leak, and increased susceptibility to arrhythmia [305–307]. Moreover, cardiomyocyte-specific NOX4 knockout attenuates transverse aortic constriction-induced hypertrophy and diastolic stiffness in mice, whereas NOX2 inhibition attenuates doxorubicin-induced cardiac atrophy in mice [305–307]. Conversely, some models suggest that basal NOX4 activity may be protective under moderate stress, so the net impact of NOX4 appears to depend on expression level, subcellular localization, and the nature of the insult [87, 308]. Importantly, NOX5 adds a Ca²⁺-sensitive layer to cardiac redox signaling in humans. NOX5 activation increases ROS and MAPK signaling activation and promotes left ventricular hypertrophy, fibrosis, and contractile dysfunction in response to Ang II and pressure overload. These findings support a model in which NOX5 amplifies Ca²⁺-driven stress signaling and adverse remodeling in susceptible hearts [309]. Recent studies suggest a synergistic effect of NOX2/NOX4-derived oxidative stress, markedly increasing ROS generation and exacerbating cardiomyocyte injury in doxorubicin-induced cardiotoxicity [310–313]. Overall, these studies highlight NOX2 as a primary therapeutic target in heart failure and cardiotoxicity, with NOX4 and NOX5 contributing important context-dependent layers to hypertrophic and fibrotic remodeling.

#### NOX in cardiac fibrosis and other pathologies

Cardiac fibrosis is a major determinant of adverse remodeling after myocardial injury, and NOX4 plays a key role as a profibrotic driver in this context. NOX4 mediates cardiac fibrosis by generating TGF-β-induced H₂O₂ in cardiac fibroblasts, which activates the Akt-mTOR and NF-κB pathways and increases the expression of TGF-β, α-SMA, collagen I, and other matrix genes [216, 314]. NOX4 upregulation has been observed in the myocardium of cardiac-specific human NOX4 transgenic mice (c-hNox4Tg), leading to cardiac remodeling characterized by hypertrophy and fibrosis as a result of oxidative stress from NOX4 activity [314]. Pharmacological inhibition or genetic KO of NOX4 reduces fibrosis, underscoring its central role [314]. Furthermore, fibroblast-specific NOX2 deficiency in mouse models markedly attenuated Ang II-induced vascular remodeling and hypertension via paracrine signaling mechanisms that promote vascular smooth muscle cell growth [300]. These findings provide a strong experimental support for a critical role of NOX2 in fibroblast activation and extracellular matrix remodeling in cardiovascular disease. The presence of NOX enzymes under other pathological conditions can exacerbate this process. Additionally, NOX1 upregulation in diabetic cardiomyopathy has been shown to drive myocardial fibrosis and cardiac dysfunction through TLR2/NF-κB-mediated oxidative stress [315]. In terms of crosstalk between cardiovascular disease and diabetes, NOX1 inhibition has been reported to reduce fibrosis, preserve cardiac function, and affect the loss of pericytes and capillary rarefaction, which are hallmark features of diabetic microvascular disease [315]. NOX5 has also been reported to be prominently upregulated in the cardiac microvascular endothelium of COVID-19 patients, where its ROS-generating activity promotes microvascular thrombosis and endothelial dysfunction, underlying key features of COVID-19 myocarditis [316]. Collectively, these studies support a unifying view that NOX4 in cardiomyocytes and fibroblasts, fibroblast NOX2, NOX1 in diabetic hearts, and inducible NOX2/NOX5 in the microvasculature form an interconnected redox network that drives cardiac fibrosis and vascular remodeling, making isoform-specific NOX modulation an attractive strategy to limit structural heart disease in both primary and comorbid conditions.

### Liver: role of NADPH oxidases in hepatic health and pathologies

In the liver, NOX isoforms exhibit cell type-specific expression patterns that support physiological processes, including detoxification and tissue regeneration under homeostatic conditions while contributing to pathological oxidative stress during liver disease [317]. NOX2 is highly abundant in Kupffer and endothelial cells and is responsible for ROS-mediated antimicrobial defense and cytokine signaling [317, 318]. In contrast, NOX4 is predominantly expressed in hepatocytes and hepatic stellate cells (HSCs), where it constitutively generates H_2_O_2_ to support redox homeostasis, promote controlled apoptosis of damaged hepatocytes downstream of TGF-β signaling, and drive HSC activation and transdifferentiation to myofibroblasts [319]. NOX4 can play both protective and pathogenic roles depending on the disease stage, cell type, and model system. During chronic liver injury, such as nonalcoholic steatohepatitis (NASH), NOX4 upregulation is observed in hepatocytes, and genetic deletion of NOX4 attenuates liver injury and fibrosis, suggesting that NOX4 contributes importantly to NASH pathogenesis in experimental models [320]. In parallel, NOX1-derived ROS in liver sinusoidal endothelial cells disrupt nitric oxide bioavailability, promoting endothelial dysfunction and impaired hepatic microcirculation, whereas NOX1 deficiency has been shown to preserve vascular homeostasis and limit nonalcoholic fatty liver disease (NAFLD) progression [321]. Experimental evidence indicates that physiological NOX activity tightly regulates cellular signaling and homeostasis. However, in disease states, excessive NOX activation exacerbates inflammation and tissue damage. Genetic KO or pharmacological inhibition of NOX isoforms improves outcomes across multiple models of NAFLD/NASH, toxic hepatitis, and liver fibrosis, highlighting NOX isoforms as essential regulators of hepatic homeostasis and promising therapeutic targets [322].

#### NOX isoforms in NAFLD and steatohepatitis

NAFLD frequently progresses to NASH, a process driven in part by NOX-dependent oxidative stress, as evidenced by the upregulation of NOX1, NOX2, and NOX4 expression in hepatocytes, Kupffer cells, and hepatic stellate cells, thus fostering insulin resistance, de novo lipogenesis, inflammatory signaling, and matrix remodeling [319, 323]. In obese db/db mice, hepatic NOX1 expression is correlated with steatosis severity. In contrast, NOX1 KO reduces intrahepatic triglyceride levels, oxidative stress, and SREBP1, FASN, and SCD1 transcript levels without altering adiposity or glycemia, indicating that hyperinsulinemia is an upstream driver [324]. Clinical studies have demonstrated that circulating NOX2-derived peptide (sNOX2-dp) levels are elevated in patients with NASH, suggesting that sNOX2-dp is a potential biomarker of disease severity [94, 325–327]. NOX4 adds a hepatocyte-HSC axis to this circuitry. In this context, Gabbia and colleagues reported that NOX4 in hepatocytes is required for TGF-β-induced apoptosis and for HSC activation and transdifferentiation into myofibroblasts, thereby promoting fibrosis. Hepatocyte-specific NOX4 deletion or inhibition attenuates lipid peroxidation, inflammation, and insulin resistance in high-fat diet models [323, 328]. Organoid and precision-cut liver slice studies further revealed that dual NOX1/4 inhibition with setanaxib reduces HSC activation and extracellular matrix (ECM) deposition in NASH-like environments while improving microcirculation [94]. Together, these findings indicate that NOX1, NOX2, and NOX4 are not mere bystanders but are active orchestrators of steatosis, inflammation, and fibrogenesis in NAFLD/NASH, supporting the use of selective NOX inhibition as a promising strategy to interrupt disease progression.

#### NOX in alcohol-induced liver disease

As in NAFLD, NOX enzymes play a central role in alcohol-associated liver disease (ALD) by linking ethanol exposure to oxidative stress, barrier dysfunction, and inflammation. Chronic ethanol intake activates NOX1 and NOX2 and induces CYP2E1 in intestinal epithelial cells and hepatocytes, disrupting barrier integrity and increasing portal delivery of ROS and endotoxin to the liver [329]. Ethanol also upregulates colonic NOX1, resulting in increased ROS generation, caspase-3-mediated apoptosis, and the loss of tight junction proteins (ZO-1, occludin, and claudin-1), thereby weakening the barrier [330]. NOX1 silencing in NCM460 cells attenuated TNF-α-induced oxidative injury, while short-chain fatty acid propionate (SCFA) downregulated NOX1 through an HDAC2-mediated epigenetic mechanism, preserving barrier function independent of free fatty acid receptor 2/3 (FFAR2/3) [330]. In the liver, NOX2 in Kupffer cells drives steatosis and inflammation via ERK1/2 activation and TNF-α release; mice lacking the cytosolic NOX subunit p47^*phox*^ are resistant to chronic ethanol-induced liver injury, underscoring the importance of NOX2-derived ROS in ALD pathogenesis [94]. NOX4 contributes to mitochondrial and cytosolic ROS generation in hepatocytes during both acute and chronic ethanol exposure, and its pharmacological inhibition protects against injury [328]. Collectively, these findings indicate that ethanol-induced oxidative stress sensitizes Kupffer cells and hepatocytes through NOX-dependent mechanisms, thereby amplifying inflammatory responses and promoting alcohol-associated liver injury [331]. These insights highlight isoform-selective NOX inhibition and Nrf2-directed therapies as rational strategies to limit alcohol-related liver injury.

#### NOX in viral hepatitis

In chronic hepatitis C, NOX4 expression and oxidative stress markers are upregulated in parallel with fibrogenic pathways, which is consistent with mechanisms whereby hepatitis C virus (HCV) proteins amplify ROS generation and promote liver fibrosis in experimental models. HSCs exposed to TGF-β exhibit increased NOX4 expression, and NOX4-derived ROS are required for stellate-cell activation, collagen production, and survival [332]. In vivo, NOX4 expression is markedly elevated in the livers of patients with HCV-related fibrosis and increases with fibrosis stage, whereas pharmacological or genetic inhibition of NOX4 attenuates experimental liver fibrosis [333]. These findings support a model in which HCV-induced inflammatory and profibrotic signaling converges on NOX4, sustaining oxidative stress, hepatocyte death, and stellate-cell activation in chronic hepatitis [334].

#### NOX activity in hepatic fibrosis and cirrhosis

Multiple lines of evidence indicate that hepatic fibrogenesis is driven by NOX1-, NOX2-, and NOX4-mediated HSC activation and ECM deposition [12, 335–337]. NOX1 is upregulated in fibrotic livers and in activated HSCs, where it promotes redox-dependent modulation of Phosphatase and tensin homolog (PTEN), leading to activation of PI3K/Akt and downstream cell-cycle pathways [338]. Notably, NOX1 KO mice resist carbon tetrachloride (CCl_4_)-induced fibrosis and exhibit reduced α-SMA/collagen I expression and lower levels of inflammatory cytokines [94, 213]. NOX2 also contributes to HSC activation and fibrogenesis [339]. In CCl_4_ or bile duct ligation (BDL) models, NOX2-derived ROS activate collagen gene transcription, HSC migration, and survival [336]. NOX2 KO or deficiency attenuates BDL/CCl_4_ fibrosis, curbing HSC survival/migration [323]. NOX4 mediates the TGF-β-Smad3 axis for myofibroblast differentiation, and NOX4-derived H₂O₂ is required for the full induction of α-SMA, collagen, and other fibrogenic genes [216]. The natural compound ursolic acid has been shown to reverse liver fibrosis in CCl4-treated mice by inhibiting the NOX4/NLRP3 inflammasome axis and reducing collagen and fibronectin expression via activation of the Nrf2/HO-1 pathway [317, 328, 340]. Similarly, dual NOX1/4 inhibition with setanaxib reduces ROS generation, HSC chemotaxis and activation, and ECM accumulation while improving the expression of markers of microvascular and parenchymal function [328]. Collectively, these studies underscore that coordinated activation of NOX1, NOX2, and NOX4 in HSCs and surrounding cells results in hepatic fibrosis and cirrhosis. Isoform-selective NOX blockade, particularly targeting NOX1 and NOX4 with agents such as setanaxib, holds substantial promise as an antifibrotic strategy in chronic liver disease.

#### NOX in cholestatic liver diseases

In bile duct ligation (BDL) models, NOX2 is robustly activated in HSCs and drives ROS-mediated inflammation and fibrosis, whereas NOX2 KO cells exhibit lower levels of hydroxyproline, α-SMA, and collagen I [94]. NOX4 also contributes to cholestatic injury by mediating hepatocyte apoptosis and HSC activation, while NOX1/4-dependent oxidative signaling promotes myofibroblast activation and fibrogenic responses in cholestatic liver disease [341]. Consistent with these roles, the dual inhibition of NOX1 and NOX4 with setanaxib attenuates BDL fibrosis via HSC quiescence [328]. Across NAFLD/NASH, alcohol-associated liver disease, viral hepatitis, and cholestatic liver disease, a consistent pattern emerges: NOX1, NOX2, and NOX4 function as nodal redox integrators that link metabolic, inflammatory, and cholestatic cues to hepatocyte injury, Kupffer cell activation, and stellate-cell-driven matrix deposition [213, 334]. Hepatic NOX isoforms therefore influence disease progression and define therapeutic windows, making isoform-selective NOX inhibition a compelling strategy to slow or reverse chronic liver disease while preserving essential host defense and regenerative signaling.

### Kidney: roles of NADPH oxidases in renal health and pathologies

In the kidney, NOX isoforms play critical roles in maintaining renal homeostasis through the regulation of ROS generation, with isoform-specific expression across glomerular, tubular, and vascular compartments. NOX4 is highly expressed in podocytes and tubular cells, where it primarily generates H₂O₂, which is required for redox signaling, adaptation to metabolic and hemodynamic stress, and normal mitochondrial function [342]. NOX1 and NOX2 are expressed in mesangial, endothelial, and immune cells, where they support vascular tone regulation, innate defense, and inflammatory responses. Under physiological conditions, NOXs support podocyte integrity, tubular reabsorption, and glomerular filtration. However, in pathological states such as diabetic nephropathy, acute kidney injury (AKI), and chronic kidney disease (CKD), NOX hyperactivation, driven by hyperglycemia, ischemia, or toxins, leads to excessive ROS generation, promoting inflammation, fibrosis, and apoptosis. This section examines how individual NOX isoforms contribute to specific kidney pathologies.

#### NOX isoforms in diabetic nephropathy

Diabetic nephropathy (DN) involves NOX-driven oxidative stress in glomeruli and tubules, leading to albuminuria, mesangial expansion, and fibrosis. NOX5 plays a key role in human DN [343]. Endothelial NOX5 further exacerbates diabetic kidney injury by amplifying oxidative stress, nitrotyrosine formation, and inflammatory gene expression (e.g., MCP-1 and TLR4), thereby worsening mesangial expansion and albuminuria in a streptozotocin (STZ)-induced NOX5 transgenic diabetic mouse model [344]. In contrast, proximal tubular NOX4 deletion does not affect DN progression in STZ models, suggesting a limited role for tubular NOX4 in hyperfiltration or glomerulosclerosis and indicating that it may not be an effective therapeutic target for managing DN [345]. However, NOX4 contributes to glomerular injury under hyperglycemic conditions through posttranscriptional mechanisms. In glomerular mesangial cells, high glucose rapidly increases NOX4 protein levels via HuR (human antigen R)-mediated translational control: HuR is upregulated and translocates to the cytoplasm, where it binds to the 3′ UTR of NOX4 mRNA and promotes its translation, thereby contributing to NOX4 upregulation, ROS generation, and the accumulation of matrix proteins such as fibronectin and collagen [346]. Inhibiting HuR or disrupting HuR-NOX4 mRNA binding reduces NOX4 expression and the expression of ROS and fibrotic markers in mesangial cells, identifying an RBP-NOX4 axis that links hyperglycemia to glomerular fibrogenesis [346]. In db/db mice, NOX4 expression is significantly upregulated in renal tubular cells, which is correlated with increased lipid peroxidation and elevated expression of hypoxia-inducible factor-1α (HIF-1α) and HO-1 and reduced expression of antioxidant enzymes and tubular injury, which are consistent with ferroptotic cell death [347]. Treatment with the ferroptosis inhibitor ferrostatin-1 reduces NOX4 levels along with HIF-1α and HO-1 expression, oxidative stress, and tubular damage, indicating that NOX-dependent ROS and HIF-1α/HO-1 signaling cooperate to drive ferroptosis-associated tubular injury in DN [347]. Finally, crosstalk between xanthine oxidase (XO) and NOX amplifies oxidative stress in DN. The inhibition of XO with febuxostat attenuated DN by lowering ROS levels and modulating VEGF/VEGFR/NOX-FoxO3a-eNOS signaling [348].

#### NOX in tubulointerstitial fibrosis and chronic kidney disease (CKD)

Tubulointerstitial fibrosis in CKD is exacerbated by NOX2 and NOX4 activity in fibroblasts and tubules, driving TGF-β-mediated epithelial-mesenchymal transition (EMT) [349]. In unilateral ureteral obstruction (UUO) models, NOX integrates with the Na/K-ATPase/Src/ROS signaling axis, where Src activates p47^*phox*^ for oxidative amplification [350]. Pharmacological disruption of the Na⁺/K⁺-ATPase/Src/ROS axis reduces tubular injury and fibrotic remodeling in UUO models [350]. Indoxyl sulfate, a protein-bound uremic toxin, induces fibrosis via the OAT/NOX/ROS/mTORC1 pathway in human kidney-2 (HK-2) tubular cells, whereas treatment with the flavoprotein inhibitor DPI blocks epithelial-mesenchymal transition (EMT) [351]. Additionally, C/EBPα upregulates NOX4-ROS in CKD fibroblasts, promoting EMT, and its knockdown reduces UUO fibrosis [352]. Overall, these findings place NOX enzymes activated by the Na⁺/K⁺-ATPase/Src signaling axis, uremic toxins, and transcriptional reprogramming at the core of tubulointerstitial fibrogenesis and highlight NOX and mTORC1-targeted strategies as promising avenues to slow CKD progression.

#### NOX in acute kidney injury (AKI) and AKI-to-chronic kidney disease (CKD)

AKI is characterized by NOX hyperactivation, increased necrosis, and inflammation, and often progresses to CKD [353]. Hydralazine, which inhibits xanthine oxidase- and NADPH oxidase-associated oxidative stress, attenuates ischemia/reperfusion-induced acute kidney injury and prevents its progression to chronic kidney disease by reducing oxidative stress, inflammation, glomerulosclerosis, and fibrosis. Furthermore, activation of the NLRP3 inflammasome and IL-1β signaling contributes to CKD progression following ischemia/reperfusion injury, underscoring the importance of oxidative stress and inflammation in both acute and chronic renal injury [354, 355]. The gut-kidney axis introduces an additional NOX-linked dimension to AKI-to-CKD progression. Gut dysbiosis promotes AKI-to-CKD progression through TMAO-mediated activation of NOX2 and downstream TGF-β/Smad3 fibrotic signaling, suggesting that gut-derived metabolites act upstream of renal NOX signaling during disease progression [337, 356–358]. At the tubular level, NOX4 is a key mediator of endotoxin-induced AKI. In LPS-induced AKI models, SH3 domain-containing YSC84-like protein 1 (SH3YL1) interacts with NOX4 and p22^*phox*^ to promote H₂O₂ generation, proinflammatory signaling, and tubular epithelial apoptosis; however, SH3YL1 KO abrogates these effects [353]. Additional studies have indicated that matricellular proteins, such as SPARC, and natural products, such as *Poria cocos* polysaccharides, modulate NADPH oxidase signaling in I/R- and LPS-induced injury [359]. Suppression of secreted protein acidic and rich in cysteine (SPARC) decreases NADPH oxidase activation and subsequent chronic fibrosis, while *Poria cocos* polysaccharides inhibit NF-κB-NOX4 in LPS-induced renal injury [296, 353]. Together, these findings position NOX2 and NOX4 as central redox hubs in AKI and in the AKI-to-CKD continuum, integrating ischemic, inflammatory, and microbiota-derived signals into maladaptive ROS responses and supporting the use of NOX-centered or NOX-modulating strategies to prevent the chronic sequelae of acute renal injury.

#### NOXs in hypertensive nephropathy

Hypertensive nephropathy, characterized by hypertension-induced renal vascular damage, glomerular sclerosis, tubular atrophy, and interstitial fibrosis, represents a major route to CKD. Accumulating evidence places NOX isoforms at the center of oxidative stress amplification, which accelerates this process [358]. In Dahl salt-sensitive (SS) rats deficient in T cells (SS^CD247−/−^), adoptive transfer of T cells from SSp67^*phox*−/−^ rats (which lack functional NOX2 due to deletion of the p67^*phox*^ subunit) attenuated the high-salt-induced increase in mean arterial pressure and albuminuria compared with the effects of transfer of wild-type SS T cells despite equal renal T-cell reconstitution, directly demonstrating that NOX2-derived ROS within T cells amplifies salt-sensitive hypertension and renal damage independent of T-cell infiltration levels [360]. Histological analysis revealed less glomerular injury and fibrosis; these results indicate that NOX2-derived ROS in T cells amplifies salt-sensitive hypertension and renal damage [358]. Deliyanti and colleagues demonstrated that, under hypertensive conditions, NOX1/NOX4-driven vascular permeability and angiogenic and inflammatory responses are exacerbated in spontaneously hypertensive rats (SHRs) compared with normotensive diabetic models and that the expression of NOX5 in transgenic endothelial cells further exacerbates microvascular injury [361]. Pharmacological inhibition of NOX1 and NOX4 reduces ROS levels, ICAM-1 expression, and vascular leakage in both normotensive and hypertensive diabetic models. Additionally, endothelial NOX5 knockdown (e.g., via siRNA) in bovine retinal endothelial cells attenuated high-glucose-induced ROS generation and vascular endothelial growth factor (VEGF) upregulation in human retinal endothelial cells, indicating that NOX1, NOX4, and NOX5 cooperate to drive hypertension-exacerbated microvascular injury. Although these data are derived from the retinal circulation, they are conceptually aligned with renal and systemic findings: under hypertensive conditions, NOX1-, NOX4-, and NOX5-driven ROS amplify vascular permeability, inflammation, and remodeling. This finding supports the idea that isoform-selective inhibition of NOX1, NOX4, and NOX5 may be a promising strategy for preventing or slowing hypertension-related microvascular and renal complications [361].

### Role of NADPH oxidases in oncogenesis and tumor progression

Across a wide range of malignancies, NOX isoforms sustain cancer cell proliferation, survival, invasion, and immune evasion through ROS-dependent activation of oncogenic and stress-response pathways. In colon cancer, NOX2 and NOX1 provide complementary growth signals. In HCT116 and RKO cells, tumor cell-intrinsic NOX2 expression generates ROS that promote proliferation and G1-S cell-cycle progression [362]. Similarly, a previous study demonstrated that the NOX1-NOX2 redox switch increases ROS levels, which in turn activate the MAPK (p38/ERK/JNK) and NF-κB/AP-1 signaling pathways, upregulate MMP-7, and suppress AMPK activity, thereby enhancing the invasiveness and metastatic potential of HCT116 cells [363]. Furthermore, Wiktorin and colleagues reported that NOX2-expressing tumor-infiltrating myeloid cells suppress cytotoxic T and NK-cell responses via ROS generation. an immunosuppressive microenvironment and promotes metastasis [364]. In hepatocellular carcinoma (HCC), NOX1 interacts with peroxiredoxin 6 (PRDX6) to stabilize NOXA1, increasing superoxide production and promoting epithelial-mesenchymal transition (EMT), whereas site-directed mutagenesis of PRDX6 in SNU475 cells suppresses NOX1-mediated N-cadherin and MMP2 upregulation, thereby reducing migration and invasion [35]. In the same cancer type, NOX2-derived ROS generated downstream of a tumor-secreted HMGB1/TLR2 axis drive autophagy-mediated NF-κB modulation and M2 macrophage polarization, thereby establishing an immunosuppressive tumor microenvironment that promotes tumorigenesis [51, 365, 366]. In addition, tumoral NOX4 in non-small cell lung cancer leads to the recruitment of M2 TAMs through ROS/PI3K signaling-dependent cytokine production, further amplifying tumor growth [51]. NOX4 is particularly involved in pancreatic ductal adenocarcinoma (PDAC) and therapy resistance. NOX4-derived ROS promote gemcitabine resistance and invasiveness in PDAC by upregulating the expression of MMP-3, as evidenced by the diminished chemoresistance and tumor progression associated with inhibition of NOX4 activity in hyperglycemic mice [367]. Additionally, NOX4 has been shown to stabilize and activate the nuclear factor erythroid 2-related factor 2 (NRF2) pro-survival response in stromal cells via a baculoviral IAP repeat-containing 5 (BIRC5)/Survivin, providing a mechanistic rationale for NOX4-mediated therapeutic resistance in a NOX4-/- syngeneic mouse model [15]. In breast cancer, p22^*phox*^ (a NOX subunit) stabilizes SERCA2a against oxidation, supporting calcium homeostasis and cell survival. p22^*phox*^-deficient MDA-MB-231 cells exhibit elevated sarcoplasmic/endoplasmic reticulum calcium ATPase 2a (SERCA2a) oxidation and reduced proliferation under oxidative stress [368]. These findings support a cohesive model in which tumor cells and stromal NOX isoforms function as redox hubs that integrate metabolic, inflammatory, and mechanical cues into oncogenic signaling, metastatic spread, and immune evasion, reinforcing the rationale for isoform-selective NOX inhibition as an adjunct strategy in cancer therapy.

#### NOX in the tumor microenvironment: ROS, immune escape, and checkpoint regulation

**Fig. 5:**
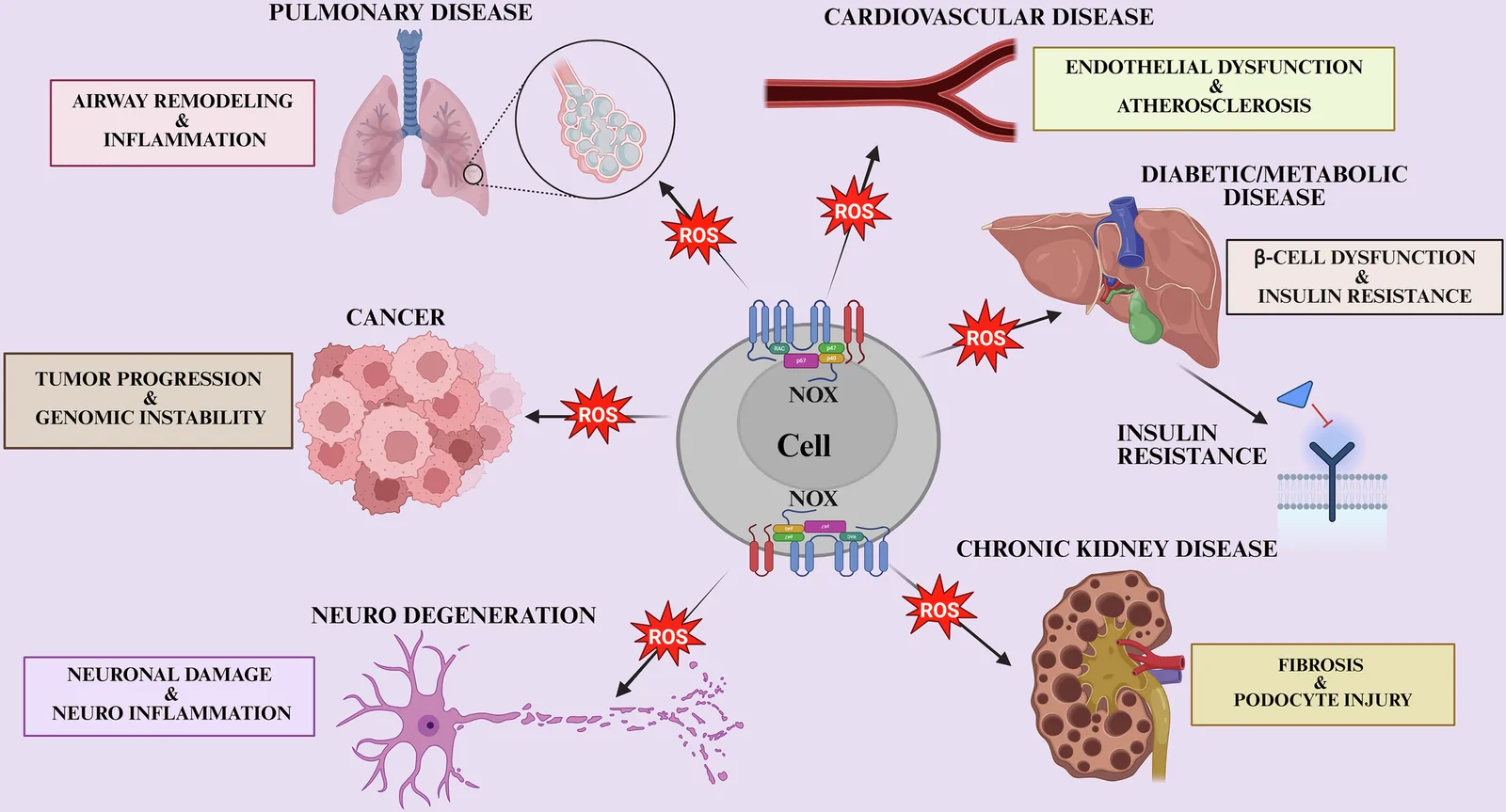
Pathological consequences of excessive NOX activation and ROS generation. NOX-mediated reactive oxygen species (ROS) generation and its contribution to oxidative stress-driven disease pathogenesis. Excessive NOX activation promotes ROS generation, leading to airway inflammation and remodeling, endothelial dysfunction, insulin resistance, kidney fibrosis, neuronal injury, and tumor progression through redox-dependent signaling pathways

NOX-generated ROS are now recognized as key sculptors of the tumor microenvironment (TME), where they reprogram cancer and stromal metabolism, dampen antitumor immunity, and modulate checkpoint signaling. In acute myeloid leukemia (AML), Ijurko and colleagues reported that NOX2 (CYBB) expression stratifies metabolic phenotypes and prognosis: NOX2-deficient AML cells display reduced basal glycolysis and oxidative phosphorylation, altered metabolite pools, and broad changes in metabolic gene expression [369]. Pancancer evaluation across the Cancer Genome Atlas (TCGA) revealed that NOX4 overexpression is correlated with poor prognosis and a protumorigenic immune microenvironment, marked by increased cancer-associated fibroblast, macrophage, and T-cell infiltration; enrichment of the TGF-β, PI3K-Akt, MAPK, and RAGE pathways; and TNF signaling [370]. These findings indicate that NOX4-driven ROS generation supports tumor progression and immune dysregulation. At the checkpoint level, NOX2 and NOX4 modulate programmed death-ligand 1 (PD-L1) and T-cell cytotoxicity in several models [371, 372]. In non-small cell lung cancer cells (NSCLCs), NOX4-derived ROS increase PD-L1 expression on tumor cells, thereby inhibiting CD8^+^ T-cell-mediated cytotoxicity [371]. Genetic and pharmacological inhibition of NOX4 reduces PD-L1 expression and restores T-cell death in cocultures [371]. In breast and other solid cancers, NOX2-derived ROS generation by immune cells has been implicated in immunosuppression via mechanisms that include arginase-1 induction, cytokine skewing, and promotion of an M2-like phenotype, suggesting that NOX2 may act as an immune checkpoint axis [373]. The diverse roles of ROS and NOX enzymes across multiple pathological conditions are schematically summarized in Fig. 5.

#### NOX in therapy resistance and novel interventions

NOX-driven oxidative stress contributes to chemoresistance and modulates the efficacy of nonconventional antitumor therapies. Two independent cohorts totaling 672 patients with diffuse large B-cell lymphoma (DLBCL) show that NOX-related polymorphisms predict clinical outcomes. Analysis of a 335 patient discovery cohort identified RAC2 variants associated with treatment toxicity, whereas a validation in an independent cohort of 337-patients identified NCF4 variants associated with survival [374, 375]. Nanozymes mimicking NOX activity offer therapeutic promise. In breast cancer, high-entropy alloy/carbon nanoflowers exhibit NADPH oxidase-like activity, depleting NADPH and inducing ferroptosis in 4T1 xenografts, resulting in significant tumor inhibition [376]. Plasma-activated hydrosols generate reactive oxygen and nitrogen species (RONS) through NOX-like mechanisms, thereby enhancing immunogenic cell death in melanoma. In B16F10 models, this treatment increased CD8^+^ T-cell infiltration and macrophage repolarization toward a more antitumor M1-like phenotype and PD-1 response [377]. While these systems are not strictly NOX-dependent, their ability to deliver controlled RONS in a manner analogous to physiological oxidase cascades complements NOX-targeted strategies and may synergize with chemotherapy, ferroptosis inducers, and immunotherapy within integrated anticancer strategies.

#### Tissue-specific NOX profiles in cancer

NOX isoform expression is highly tissue-specific, resulting in distinct and in some cases opposing-roles in tumor biology. In hepatocellular carcinoma (HCC), NOX4 deletion accelerates tumorigenesis via M2 (TAM) polarization. In Diethylnitrosamine (DEN)/CCl_4_ murine models of fibrosis-associated HCC, NOX4 deficiency attenuated hepatic fibrosis but paradoxically increased tumor number and HCC cell proliferation, with the absence of NOX4 resulting in macrophage polarization toward protumorigenic M2-type TAMs, supporting the concept that NOX4-derived ROS can suppress hepatocarcinogenesis in the chronically injured liver [50]. In contrast, NOX4 acts as a pro-oncogenic driver in most other solid tumors: Pancancer TCGA analyses revealed that NOX4 is significantly overexpressed in breast, colon, esophageal, lung, prostate, and thyroid cancers but is paradoxically downregulated in renal clear cell carcinoma, where it has been linked to von Hippel-Lindau (VHL)-dependent HIF-2α signaling [370]. NOX5, which is absent from the rodent genome, is specifically overexpressed in prostate cancer cell lines, breast tumors, and Barrett’s esophageal adenocarcinoma, where Ca²⁺-dependent superoxide production promotes ROS-driven proliferation and progression to adenocarcinoma [90]. In thyroid cancer, NOX4 and DUOX1/2 exhibit distinct expression patterns across papillary, follicular, and anaplastic subtypes, with upregulation of NOX4 expression in BRAF(V600E)-mutant papillary thyroid carcinoma linked to oxidative DNA damage and dedifferentiation [378]. These tissue-specific profiles underscore that NOX isoform targeting in cancer requires careful contextualization by tumor type, microenvironment, and genetic background [379].

#### NOX in hepatocellular carcinoma (HCC)

HCC often develops on a background of chronic liver injury and fibrosis, with NOX isoforms exerting isoform- and compartment-specific effects. NOX4 acts as a tumor suppressor; however, its deficiency in DEN/CCl_4_ models reduces fibrosis while increasing the incidence and multiplicity of HCC through M2-TAM polarization (CD206^+^, IL-6, TNF-α), along with increasing the expression of the proliferation markers Ki67 and PCNA (proliferating cell nuclear antigen) [50]. Mechanistically, NOX4 loss upregulates Nrf2 and MYC expression, reprogramming mitochondrial biogenesis; shifting metabolism toward oxidative phosphorylation, gluconeogenesis, and fatty acid oxidation; and enhancing ATP levels and tumorigenicity [380]. Conversely, NOX1 promotes invasion and metastasis. Recent studies have demonstrated that the NOXA1-PRDX6 (peroxiredoxin 6) interaction stabilizes the NOX1 complex and increases superoxide production in HCC cell lines, resulting in the upregulation of N-cadherin and MMP2 expression and increased invasion in SNU475 cells [381–383]. In contrast, PRDX6 mutants destabilize NOXA1, decreasing NOX1 activity and markedly reducing NOX1-dependent migration and invasion [35, 384]. Additionally, NOX1 inhibition sensitizes HCC to sorafenib and radiotherapy by reversing the EMT [323]. NOX2, in turn, promotes angiogenesis and VEGF signaling within the TME.

### Aging: roles of NADPH oxidases in aging processes

NOX isoforms contribute to multiple hallmarks of aging, including cellular senescence, inflammation, and age-related organ dysfunction, primarily through controlled or dysregulated generation of ROS, oxidative stress, and DNA damage. Moreover, several studies highlight context-dependent, adaptive roles of specific NOX isoforms in maintaining redox homeostasis, supporting NRF2-mediated antioxidant signaling, and preserving tissue integrity under stress. These findings underscore the dual role of NOX enzymes as both detrimental and protective mediators, depending on the isoform, cell type, and stage of injury. This section focuses on how distinct NOX isoforms modulate aging processes across different organs and cell types, including the vasculature, kidney, lung, and immune system, and how their activities intersect with broader mechanisms of aging, including mitochondrial dysfunction, impaired autophagy, and senescence-associated secretory phenotypes.

#### NOX in cellular senescence

NOX4-derived ROS have been directly linked to disc-cell senescence in experimental models of IDD. In nucleus pulposus and annulus fibrosus cells, NOX4 upregulation increases ROS generation, activates DNA damage responses, and triggers p53/p21 signaling, leading to cell-cycle arrest, senescence, and matrix catabolism that accelerate age-related disc degeneration [142]. NOX isoforms also contribute to aging-related diabetic kidney disease. In aging diabetic mice and rats, renal expression of NOX1, NOX2, and NOX4 is markedly increased, accompanied by elevated oxidative stress, inflammation, mesangial expansion, and fibrosis, along with impaired renal function [385]. Emerging work links NOX1-derived ROS to prothrombotic phenotypes in aging red blood cells (RBCs). Chauhan et al. identified a novel RBC pathway in which Rpl13a-associated snoRNAs regulate NOX1-dependent ROS generation, promote complement C3a deposition on red blood cells, increase prothrombin activation via NOX1 and GRK2 coactivation, and contribute to thrombosis in aging and venous thromboembolism models, with in vivo Rpl13a snoRNA KO in aged mice reducing thrombus size by decreasing C3a deposition and RBC-triggered prothrombin activation [386], indicating that this red cell-specific redox control pathway is involved in age-associated vascular dysfunction [387]. In the aging retina, NOX2 has been identified as a principal source of oxidative and inflammatory injury. Experimental models of glaucomatous or stress-exposed retinas from older mice exhibit upregulated NOX2 levels; increased ROS levels; microglial and vascular activation; and progressive neurodegeneration of retinal ganglion cells, resembling age-associated glaucoma [388–390]. Genetic deletion or pharmacologic inhibition of NOX2 markedly reduces retinal oxidative stress, dampens inflammatory signaling, and preserves neuronal structure and function, indicating that NOX2-driven ROS are central to age-related retinal pathology and may represent a tractable target for neuroprotective interventions in glaucoma and retinal aging [388].

#### NOX in age-related diseases

Age-related pathologies, including neurodegeneration, cardiac dysfunction, and renal fibrosis, are closely linked to dysregulated NADPH oxidase activity. In diabetic murine kidneys, pan-NOX inhibitors significantly improve renal outcomes by dampening NOX-dependent fibrogenesis [385]. Evidence from models of cardiac senescence indicates that NOX4-mediated eNOS uncoupling contributes to age-related myocardial dysfunction [391]. Mitochondrial dysfunction and apoptosis, which are regulated by NOX enzymes, underpin cochlear aging [131]. Environmental and tissue-specific stressors also converge on NOX to promote organ-specific aging phenotypes. For instance, diesel particulate extract activates NOX in human dermal fibroblasts, increasing ROS generation, ceramide-1-phosphate signaling, MMP-1/MMP-3 expression, collagen degradation, and senescence markers, thereby accelerating premature skin aging [392]. More broadly, NOX-derived ROS, together with eNOS uncoupling, play a central role in endothelial dysfunction and impaired vasodilation, which are key features of vascular aging.

### Interactions between NOXs and the microbiome: bidirectional crosstalk between the gut and lung health and disease

NOX enzymes interact bidirectionally with the microbiome: microbial signals influence NOX activity, while NOX-derived ROS, in turn, modulate the microbial composition and associated inflammatory processes. This crosstalk is critical for maintaining homeostasis but can also drive pathology in diseases such as COPD (COPD) and idiopathic pulmonary fibrosis (IPF), where dysregulation amplifies oxidative stress and inflammation. Despite the growing recognition of this relationship, mechanistic studies specifically investigating NADPH oxidase isoform-microbiome coupling remain limited. This section focuses on how gut microbiota-derived signals regulate NOX expression and activity and how NOX-generated ROS provide feedback to remodel microbial communities and inflammatory responses along the gut-lung axis in health and disease.

#### Gut microbiota metabolites modulate NOX expression via TLR/NOD signaling

Gut-derived short-chain fatty acids (SCFAs) and LPS are sensed by pattern recognition receptors (PRRs), including Toll-like receptors (TLRs) and NOD-like receptors (NLRs), and modulate NOX enzyme activity and ROS generation. These responses are context-dependent and can be either pro- or anti-inflammatory. While the net effect often supports gut homeostasis, dysregulation can also drive systemic inflammation [393]. In colonic stem cells (CSCs), NOX1 is primarily active in proliferating cells and is stimulated by the intestinal microbiota through TLR activation [394]. TLR activation leads to increased NOX1-dependent reactive oxygen species (ROS) production, which promotes CSC proliferation and promotes adaptation to changes in the microbial environment. Mechanistically, NOX1-derived ROS induce redox-dependent phosphorylation of EGFR, sustaining proliferative signaling by inactivating regulatory phosphatases and ensuring stem-cell expansion in response to microbial cues [394]. In germ-free models, NOX1 expression levels are reduced, leading to impaired CSC renewal and disrupted epithelial homeostasis, highlighting the role of the microbiota in fine-tuning NOX activity for gut repair and barrier integrity [394]. In addition to the intestine, NOD1 provides an additional link between microbial sensing and NOX-derived ROS. In adipocytes, NOD1 activation induces oxidative stress via NOX1 and NOX4, engaging PKCδ and downstream NF-κB-MAPK pathways. This NOX1/NOX4-dependent ROS generation contributes to inflammatory signaling, insulin resistance, and metabolic dysfunction [395]. Collectively these findings illustrate how PRR-NOX signaling integrates microbiota-derived cues with redox signaling across multiple tissues, producing outcomes that range from adaptive tissue remodeling to chronic inflammation, depending on the context and magnitude of activation.

#### Gut-lung axis: microbiome crosstalk in health and lung diseases

The gut-lung axis is a critical bidirectional communication pathway through which the gut microbiota modulates pulmonary immunity and, reciprocally, lung conditions influence gut microbial communities, primarily via immune cell trafficking and circulating metabolites [396]. In murine sepsis models induced by CLP, gut dysbiosis, characterized by increased Proteobacteria, promotes intestinal IL-33 production and IL-25-driven inflammatory ILC2 (iILC2) accumulation in the intestine, representing a microbiota-regulated gut-lung immune axis [396, 397]. These cytokines promote the migration of group 2 innate lymphoid cells (ILC2s) from the gut to the lung, where ILC2-derived cytokines limit lung injury and support barrier protection. This process illustrates how microbiota-dependent signals calibrate distal lung responses, attenuating pulmonary inflammation and tissue damage during infections such as *Pseudomonas aeruginosa* [396]. Fecal microbiota transplantation and cohousing experiments further demonstrated that restoring or transferring protective microbial communities normalizes ILC2 dynamics and reduces sepsis-associated lung damage, providing a mechanistic framework linking dysbiosis to protective type 2 lung immunity through ILC2 trafficking along the gut-lung axis [398]. These findings highlight a previously unrecognized role of gut microbial communities in regulating distal lung immunity through ILC2 trafficking along the gut-lung axis. The gut microbiota also modulates susceptibility to fibrotic lung injury in a strain- and community-dependent manner. Recent work has shown that the gut microbiota profile of specific mouse substrains is causally associated with the severity of bleomycin-induced lung fibrosis and that the transfer of protective taxa can reduce mortality [399]. Additionally, antibiotic-induced microbiota depletion has been shown to impair type 2 immunity, potentially contributing to a profibrotic environment in tissue repair [400]. The influence of the gut microbiota on lung oxidative damage, including effects potentially mediated by NOX enzymes, is likely driven by gut-derived metabolites and immune signals rather than simply by the absence of microbes, highlighting a more nuanced relationship between the gut microbiota and distant organ responses. Although the studies discussed here do not directly examine NOX enzymes, they demonstrate that gut microbiota regulate pulmonary immunity through microbial metabolites, cytokine signaling, and immune cell trafficking. Given the established role of NOX-derived ROS in inflammatory signaling and tissue injury, future studies should determine how microbiota-dependent immune pathways intersect with NOX-mediated redox signaling along the gut-lung axis.

#### NOX-generated ROS shape microbiome composition and inflammation

The NADPH oxidase family, particularly NOX1, plays a pivotal role in regulating host-microbiota interactions and epithelial homeostasis through redox-dependent signaling. Several studies have demonstrated that NOX1-derived H₂O₂ at the intestinal surface influences bacterial fitness and spatial organization. Specifically, NOX1 activity in colonocytes supports bacterial respiration under microaerophilic conditions, thereby influencing microbial persistence at the mucosal interface [401]. These findings suggest that epithelial ROS generated by NOX activity can create selective oxidative niches that allow certain bacterial taxa to thrive while restricting others.

Microbiota-derived and cytokine-mediated signaling further modulates epithelial NOX1 expression, forming a dynamic feedback loop between the host and microbiome. In mouse models, microbial and TLR signaling induce NOX1 expression in colonic stem and progenitor cells, with NOX1-dependent ROS driving epithelial proliferation, maintaining epithelial barrier integrity and promoting regeneration [394, 402]. Complementary studies have shown that TNF-α stimulation of intestinal epithelial cells triggers NOX1-mediated ROS generation, which fine-tunes epithelial differentiation and immune readiness. Loss of NOX1 disrupts these pathways, leading to impaired cytokine signaling and altered epithelial composition [16, 109]. Together, these findings establish that epithelial NOX1 functions as both a sensor and an effector of inflammatory and microbial cues, maintaining the balance between proliferation and inflammation.

Beyond the colon, the combined activity of epithelial NOX1 and inducible nitric oxide synthase (iNOS) provides a redox shield that stabilizes mucosal microbial communities. In mice deficient in both enzymes, bacterial overgrowth and compositional shifts occur in the ileum, demonstrating that NOX- and iNOS-derived reactive species are essential for maintaining bacterial homeostasis and preventing dysbiosis [403]. Mechanistically, NOX-derived ROS regulate microbial colonization through the oxidation of bacterial proteins and lipids, affecting biofilm formation, whereas low physiological ROS levels promote epithelial renewal, thereby preserving mucosal integrity and host-microbe balance.

#### NOX in rare diseases and regenerative medicine

NADPH oxidases (NOXs) play a dual, context-dependent role in regenerative biology and rare monogenic NOX disorders. At the physiological level, NOX-derived ROS act as instructive signaling molecules that regulate stem-cell self-renewal, differentiation, and tissue repair, positioning NOX isoforms as key regulators of redox homeostasis in regenerative medicine strategies [84].

#### Chronic granulomatous disease (CGD) and clinical correction of NOX2 deficiency

X-linked CGD, caused by mutations in the cytochrome b-245 beta chain (CYBB/gp91^*phox*^), abolishes the oxidative burst of the phagocyte, leading to life-threatening infections and granulomatous inflammation. In first-in-human lentiviral gene therapy trials, ex vivo correction of patient CD34⁺ hematopoietic stem and progenitor cells (HSPCs) has been shown to restore measurable oxidase activity, with surviving patients maintaining stable vector copy numbers and oxidase-positive neutrophils at 12 months, along with reduced infection rates [404]. Subsequent translational work has identified a practical barrier: preexisting hematopoietic inflammation can compromise the engraftment and persistence of corrected HSPCs, a finding demonstrated by single-cell and transcriptomic analyses of trial samples. These findings underscore the importance of controlling marrow inflammation during gene and cell therapy [405]. Finally, advanced genome-editing approaches have achieved high-efficiency correction of CYBB mutations in HSPCs with sustained engraftment and persistence after transplantation in preclinical models, offering a promising alternative to integrating viral vector-based gene therapy [406].

#### NOX and stem-cell fate

Multiple studies have shown that NOX isoforms act as signaling nodes during differentiation and stress adaptation [407, 408]. Human iPSC (HiPSC)-based models demonstrate NOX4 upregulation and NOX4-dependent oxidative stress during cardiomyocyte differentiation as well as in disease contexts [409–411]. Duelen and colleagues reported that in hiPSC-derived cardiomyocyte models, NOX4 modulation alters mitochondrial stress, contractile function, oxidative stress levels, and overall disease-related phenotypes [409]. NOX4 and related ROS signaling have also been experimentally implicated in osteogenic and osteoblastic differentiation in vertebrate models [412, 413]. The perturbation of NOX4 alters osteoblast numbers and bone mineralization in zebrafish and in vitro cell models [413]. In addition, extracellular vesicle (EV) studies have shown that mesenchymal stem-cell-derived extracellular vesicles (MSC-EVs) regulate recipient cell NOX4 expression and ROS generation, thereby protecting cardiomyocytes from doxorubicin-induced injury [414, 415]. Conversely, experimental manipulation of NOX4 levels in zebrafish and human osteoblast cell models alters the ROS balance, osteoblast numbers, and bone mineralization outcomes [415]. Collectively, these findings support a model in which tightly regulated NOX4 activity promotes differentiation and adaptive stress responses, whereas sustained or excessive NOX4 signaling drives maladaptive oxidative injury and cellular dysfunction.

#### NOX modulation in tissue repair

Preclinical models have shown that transient modulation of NOX activity reshapes repair outcomes in a context-dependent manner [416]. In models of neural and pulmonary injury, selective NOX2 inhibition with the peptide gp91ds-tat or small molecules reduces injury-associated inflammation and improves functional recovery in rodents [417, 418]. Khayrullina and colleagues demonstrated differential effects of NOX2 versus NOX4 inhibition after spinal cord injury, including distinct effects on functional recovery outcomes [419]. In influenza A virus infection, intranasal delivery of an endosome-targeted NOX2 inhibitor, alone or in combination with the mitochondrial ROS scavenger Mito-TEMPO, reduces lung cytokine production, limits neutrophilic inflammation, and improves survival in mice. These findings demonstrate that targeted NOX2 blockade can fine-tune antiviral immunity without abolishing adaptive immune responses [420].

Cutaneous wound-healing studies further underscore a nuanced balance between beneficial and detrimental ROS signaling. Complete genetic loss of NOX isoforms often impairs tissue repair, whereas temporary pharmacologic inhibition can be beneficial in specific inflammatory contexts [421]. For example, several studies report that transient NOX2 inhibition after acute injury reduces oxidative damage and improves recovery [419, 422, 423]. In contrast, genetic NOX4 deficiency delays wound closure, impairs angiogenesis and ECM dityrosine cross-linking, and reduces effective wound contraction, indicating that NOX4-derived ROS are required for normal tissue repair [424]. In MSC-based cardiac repair, preclinical studies have shown that MSCs or MSC-derived products that modulate NOX4-dependent ROS signaling improve myocardial outcomes after myocardial injury [409, 415]. Table 4 summarizes conserved NOX-regulated stress response pathways across multiple organs, providing an integrated view of redox signaling beyond tissue-specific disease contexts. Collectively, these data support a model in which carefully timed, isoform-specific NOX targeting can enhance regeneration and limit maladaptive inflammation, whereas complete or sustained NOX suppression may impair physiological repair processes.

**Table 4:** In vitro evidence for redox signaling crosstalk in cellular pathways, and functional outcomes

Nox crosstalk category	Specific pathway/mechanism	Key players/molecules	Biological/functional outcome	Recent evidence	References
ER stress	UPR branches (ATF4-CHOP) upregulate NOX2/NOX4 expression	GRP78, CHOP, ATF6; NOX2, NOX4	Increased ROS generation, reduced NO bioavailability, endothelial dysfunction, and elevated vascular tone	In angiotensin II-infused mice, ER stress markers (BiP, CHOP) and NOX2/4 were upregulated in aorta/mesenteric arteries; ER stress inhibition (4-PBA, TUDCA) normalized BP and reduced NOX expression	[571]
Autophagy/mitophagy	Engagement of TLRs or Fcγ receptors during phagocytosis activates NOX2, producing ROS that trigger LC3 recruitment to single-membrane phagosomes (LC3-associated phagocytosis)	NOX2 complex (gp91^*phox*^, p22^*phox*^, p47^*phox*^, p67^*phox*^), LC3 (ATG 4,5,12,16), Beclin-1, ATG5, TLRs, FcγRs	Enhanced maturation of phagosomes and antibacterial clearance (*Salmonella* sp.)	NOX2-deficient cells fail to recruit LC3 and show impaired killing; identifies a direct NOX-autophagy link in innate immunity and inflammation	[572]
	Pentylenetetrazole (PTZ) kindling activates NOX2 in hippocampal neurons, generating ROS that stimulate autophagic pathways	NOX2 (gp91^*phox*^), ROS, LC3-II, Beclin-1	Autophagy induction in the hippocampus during epileptogenesis; supports neuronal remodeling under seizure stress	Pharmacological inhibition of NOX (apocynin) or genetic knockdown reduces LC3-II/Beclin-1 and attenuates autophagy; shows NOX2-ROS is essential for seizure-triggered autophagy	[573]
	Upon microbial infection or TLR2 stimulation, Rubicon interacts with the p22^*phox*^ subunit of the NOX2 complex, facilitating its trafficking to phagosomes and boosting ROS generation	Rubicon, p22^*phox*^, gp91^*phox*^ (NOX2 complex), TLR2, Beclin-1/Vps34 complex	Enhanced ROS burst, inflammatory cytokine production, and antimicrobial activity via phagosomal ROS	Rubicon’s dual role in autophagy and phagocytic ROS generation is genetically and functionally separable; manipulating Rubicon profoundly affects ROS, cytokine levels, and microbial killing	[574]
	In persistent pulmonary hypertension PAEC, increased NOX2 activity elevates ROS, which enhances autophagy; conversely, increased autophagy promotes NOX2 (gp91^*phox*^-p47^*phox*^) association and activity, creating a positive feedback loop	NOX2 complex (gp91^*phox*^, p47^*phox*^), ROS, LC3-II/LC3-I ratio (autophagy marker), Beclin-1 (autophagy mediator)	Excessive autophagy leads to impaired angiogenesis and increased apoptosis in PPHN-PAEC	Autophagy inhibition (3-MA, chloroquine, Beclin-1 knockdown) reduced NOX2 activity and ROS, improving angiogenesis; NOX inhibition also decreased autophagy	[575]
SIRT1-Autophagy crosstalk	Hydroxytyrosol (HT) activates SIRT1, which induces autophagy ( ↑ LC3, ATG5, ATG7; ↓p62). HT-SIRT1 signaling downregulates AOPP-induced NOX expression and suppresses inflammatory cytokine production	HT (hydroxytyrosol), SIRT1, LC3, ATG5, ATG7, p62/SQSTM1, AOPP-induced NOX, inflammatory cytokines	Enhanced autophagy; reduced ROS, decreased NADPH oxidase expression, and attenuated inflammation	In chondrocytes pretreated with HT and then exposed to AOPPs: increased autophagy markers via SIRT1; NOX expression and cytokines were significantly reduced through this pathway	[576]
Epigenetic regulation	PM activates the aryl hydrocarbon receptor (AhR), which triggers epigenetic modifications (DNA methylation, histone acetylation) at NADPH oxidase gene loci, enhancing transcription and ROS generation	PM, AhR, DNA methyltransferases (DNMTs), histone acetyltransferases (HATs), NOX1, NOX2, ROS	Upregulated NOX expression → increased ROS generation → oxidative stress and endothelial dysfunction	Epigenetic inhibitors (DNMT or HAT inhibitors) attenuate NOX upregulation and ROS, confirming the AhR-epigenetic-NOX axis, showing a mechanistic link between environmental PM exposure and redox imbalance in vascular cells	[182]
	Histone modifications regulate NOX4 transcription during cellular senescence; enrichment of the activating H4K16Ac mark and reduced association of the repressive H4K20Me3 mark favor NOX4 expression	NOX4, H4K16Ac, H4K20Me3, MOF, DNMTs, methyl-CpG-binding proteins	Increased NOX4 expression/activity and ROS-generating capacity associated with the senescent fibroblast phenotype	Senescent human lung fibroblasts showed increased NOX4 expression/activity and H4K16Ac enrichment at the NOX4 gene; MOF silencing reduced NOX4 expression, supporting direct epigenetic regulation of NOX4	[577]
	NADPH directly binds to HDAC3, inhibiting its association with the NCoR complex, leading to reduced histone deacetylation and increased histone acetylation	NADPH, HDAC3, NCoR (Nuclear Receptor Co-Repressor), histones (H3, H4), acetyl groups	Enhanced histone acetylation, leading to a more open chromatin structure and increased gene transcription	NADPH competes with Ins(1,4,5,6)P₄ for binding to HDAC3, with NADPH exhibiting a higher affinity. This interaction disrupts the HDAC3-NCoR complex, highlighting a metabolism-independent role of NADPH in epigenetic regulation	[578]
Hippo pathway	Hypoxia activates NADPH oxidases (NOX), leading to increased ROS generation. ROS activate the Hippo pathway coactivator Yes-Associated Protein (YAP), which translocates to the nucleus and promotes the cardiac response to hypoxia	NOX isoforms, ROS, YAP	Enhanced cardiac adaptation to hypoxic conditions through YAP-mediated transcriptional activation	The study investigates the interplay between NOX-derived ROS and YAP activation in cardiomyoblasts and murine hearts under hypoxic conditions	[579]
	Activation of the Hippo pathway leads to mitochondrial dysfunction by repressing mitochondrial genes, which causally promotes the development of dilated DCM	Hippo pathway, YAP, TEAD1, mitochondrial genes	Mitochondrial dysfunction leads to DCM in mice	The study provides evidence that Hippo signaling activation mediates mitochondrial damage by repressing mitochondrial genes, which causally promotes the development of DCM	[580]
	β-adrenoceptor (βAR) stimulation activates the Hippo signaling pathway, leading to mitochondrial dysfunction characterized by decreased ATP levels, increased ROS generation, and altered mitochondrial morphology	βAR, Hippo pathway, Mst1 kinase, YAP, TEAD1, mitochondrial proteins, ATP, ROS	Acute mitochondrial damage and energy insufficiency, contributing to transient cardiac dysfunction observed in Takotsubo syndrome.	Inhibition of Mst1 kinase mitigated mitochondrial damage and improved cardiac function in the acute phase of Takotsubo syndrome in mice	[581]
	Bone marrow mesenchymal stem-cell-derived exosomes (BMSC-Exos) deliver long noncoding RNA GAS5 to cardiomyocytes, where GAS5 interacts with UL3. This interaction inhibits the Hippo pathway, leading to reduced ferroptosis and improved cardiac function	BMSC-Exos, GAS5, UL3, Hippo pathway, YAP, TAZ, ferroptosis markers (ACSL4, GPX4, Fe²⁺, MDA, ROS, SOD, GSH)	Reduced ferroptosis, decreased oxidative stress, improved myocardial function, and attenuation of heart failure symptoms	GAS5 overexpression in BMSC-Exos enhanced myocardial protection in rats with heart failure, evidenced by improved cardiac function and reduced ferroptosis markers	[582]
Lipid rafts	Naringenin inhibits acid sphingomyelinase (ASMase), reducing lipid-raft clustering and preventing the membrane recruitment of TLR4 and NADPH oxidase subunit p47^*phox*^. This disruption attenuates downstream NF-κB activation and proinflammatory cytokine release	Naringenin, ASMase, lipid rafts, TLR4, p47^*phox*^, NF-κB, IL-1β, IL-6, TNF-α	Reduced macrophage infiltration, decreased vascular inflammation, and attenuation of atherosclerotic progression	In ApoE-deficient mice on a high-fat diet, naringenin treatment led to decreased aortic cytokine levels and inhibited macrophage activation and vascular inflammation	[583]
	Environmental toxicants such as polychlorinated biphenyls (PCBs), benzo[a]pyrene (B[a]P), and metals/metalloids accumulate in lipid rafts, leading to the activation of raft-localized NOX enzymes, aryl hydrocarbon receptor (AhR), caveolin-1, and protein kinases. This activation triggers oxidative stress, inflammation, and amyloidogenesis	NOX, AhR, caveolin-1, protein kinases, PCBs, B[a]P, metals/metalloids	Increased oxidative stress, inflammation, and amyloidogenesis in hepatocytes, macrophages, and neurons	Studies have shown that the accumulation of toxicants in lipid rafts leads to the activation of signaling pathways that contribute to cellular dysfunction and disease progression	[159]
	Activation of P2Y13 purinergic receptors by ADP leads to Ca²⁺ mobilization and subsequent ROS generation via NADPH oxidase. This process is dependent on lipid-raft integrity and Gi protein signaling	P2Y13 receptors, Gi proteins, Ca²⁺, ROS, NADPH oxidase, lipid rafts	Reduced synaptic vesicle exocytosis and neurotransmitter release at the neuromuscular junction	The inhibitory effect of ADP on neurotransmitter release is suppressed by antagonists of P2Y13 receptors (MRS 2211), Ca²⁺ mobilization (TMB8), protein kinase C (chelerythrine), NADPH oxidase (VAS2870), and antioxidants	[584]
	NOX2-derived ROS activate the NLRP3 inflammasome in hematopoietic stem and progenitor cells (HSPCs). This activation facilitates the assembly of membrane lipid rafts (MLRs), which are crucial for the proper chemotactic response to migratory signals	NOX2, ROS, NLRP3 inflammasome, membrane lipid rafts (MLRs), CXCR4, SDF-1, S1P, eATP	Enhanced migration and homing of HSPCs to bone marrow niches and improved engraftment post-transplantation	NOX2-deficient HSPCs exhibit impaired migration, reduced Nlrp3 inflammasome activation, and defective MLR formation, leading to compromised HSPC trafficking and engraftment	[585]
Circadian regulation	Circadian rhythms influence NADPH levels, which in turn regulate mitochondrial quality control mechanisms, including mitophagy and mitochondrial biogenesis. Disruption of these processes is implicated in the pathogenesis of Parkinson’s disease	NADPH, PINK1, Parkin, DJ-1, mitophagy, mitochondrial biogenesis	Impaired mitochondrial function, increased oxidative stress, and neurodegeneration are associated with Parkinson’s disease	Circadian-based therapies, such as fasting and exercise, have been shown to modulate NADPH levels and enhance mitochondrial quality control, potentially slowing the progression of Parkinson’s disease	[586]
	Proinflammatory stimuli, such as lipopolysaccharide (LPS), disrupt circadian rhythms in pulmonary endothelial cells via NOX2-derived ROS. These ROS activate the NLRP3 inflammasome, leading to sustained inflammation	NOX2, ROS, NLRP3 inflammasome, BMAL1, Cry1/2, ICAM-1, PMN adherence	Disrupted circadian rhythms, sustained inflammation, and impaired resolution of inflammatory responses in the pulmonary endothelium	Inhibition of NOX2 with diphenyleneiodonium (DPI) restored circadian rhythmicity and reduced inflammation in LPS-treated mice. Disruption of clock genes Bmal1 and Cry1/2 exacerbated inflammation and disrupted circadian control	[587]
	Sleep fragmentation (SF) leads to persistent activation of NOX4 in the liver through SIRT1-dependent epigenetic modulation. SF induces acetylation of histone H3 at lysine 27 (H3K27ac) at the NOX4 promoter, enhancing NOX4 expression. This epigenetic modification persists even after sleep recovery, contributing to sustained oxidative stress and inflammation in both the liver and the heart	NOX4, SIRT1, H3K27ac, BMAL1, Resveratrol (SIRT1 activator), GLX351322 (NOX4 inhibitor)	Persistent hepatic steatosis, oxidative damage, and cardiac dysfunction, even after sleep recovery	Pharmacological interventions targeting NOX4 (GLX351322) or SIRT1 (Resveratrol) effectively reduce H3K27ac levels at the Nox4 promoter, ameliorating liver pathology and mitigating associated cardiovascular damage	[588]

*AhR* aryl hydrocarbon receptor, *AOPP* advanced oxidation protein product, *BMSC*-Exos bone marrow mesenchymal stem-cell-derived exosomes, *DCM* dilated cardiomyopathy, *DNMT* DNA methyltransferase, *ER* endoplasmic reticulum, *FcγR* Fc gamma receptor, *HAT* histone acetyltransferase, *HDAC* histone deacetylase, *3-MA* 3-methyladenine, *4-PBA* 4-phenylbutyric acid, *ASMase* acid sphingomyelinase, *B[a]P* benzo[a]pyrene, *BMAL1* brain and muscle ARNT-like protein 1, *Cry1/2* cryptochrome 1/2, *eATP* extracellular adenosine triphosphate, *GAS5* growth arrest-specific 5, *MLRs* membrane lipid rafts, *P2Y13* purinergic receptor P2Y13, *PCBs* polychlorinated biphenyls, *PMN* polymorphonuclear neutrophils, *PTZ* pentylenetetrazole, *S1P* sphingosine-1-phosphate, *SDF-1* stromal cell-derived factor 1, *SF* sleep fragmentation, *TUDCA* tauroursodeoxycholic acid, *βAR* beta-adrenergic receptor, *HSPC* hematopoietic stem and progenitor cell, *LC3* microtubule-associated protein 1 light chain 3, *LPS* lipopolysaccharide, *NADPH* nicotinamide adenine dinucleotide phosphate, *NO* nitric oxide, *NOX* NADPH oxidase, *PAEC* pulmonary artery endothelial cells, *PM* particulate matter, *PPHN* persistent pulmonary hypertension of the newborn, *ROS* reactive oxygen species, *TLR* toll-like receptor, *UPR* unfolded protein response, *SQSTM1* Sequestosome 1

## Therapeutic targeting of NOX isoforms

Therapeutic approaches targeting NOX isoforms have evolved significantly, with isoform-specific strategies now being prioritized across pulmonary, cardiovascular, renal, hepatic, neurological, oncologic, and aging-related pathologies. These approaches include the use of selective small-molecule inhibitors, peptide inhibitors that disrupt NOX complex assembly, gene silencing strategies, nanoparticle-mediated delivery systems, ROS modulators, and combination therapies. Collectively, these strategies aim to reduce pathological reactive oxygen species (ROS) generation while preserving physiological redox signaling. Achieving isoform specificity, ensuring efficient drug delivery to target tissues, maintaining on-target safety, particularly with respect to host immune defense, and translating promising preclinical findings into clinically meaningful outcomes remain key challenges.

### Small-molecule inhibitors

Small-molecule inhibitors remain the most intensively explored therapeutic approach for modulating NOX activity. Structurally and mechanistically, NOX enzymes shuttle electrons from NADPH to FAD and subsequently to transmembrane hemes to reduce O₂, defining two principal pharmacological intervention points: (i) catalytic blockade of electron transfer at the flavin/heme catalytic machinery and (ii) disruption of protein-protein interaction regions required for assembly of the active NOX-p22^*phox*^ complex [11]. A diverse chemical toolbox has emerged, ranging from broad-spectrum flavin/heme catalytic inhibitors to isoform-selective antagonists and small-molecule protein-protein interaction (PPI) antagonists. Representative compounds include setanaxib (GKT137831), a dual NOX1/NOX4 inhibitor with reproducible antifibrotic and antioxidant effects in cardiac, hepatic, renal, and pulmonary models, as supported by preclinical pharmacokinetic (PK) and pharmacodynamic (PD) data [411, 425–428]. APX-115, which is a pan-NOX inhibitor, has been shown to have renal-protective and anti-inflammatory effects in diabetic nephropathy and related preclinical models [429]. VAS2870 and its analog VAS3947, which are both classified as early pan-NOX tool compounds, are widely used for mechanistic studies; they inhibit cellular ROS generation and show in vivo activity but exhibit documented off-target effects and noncatalytic modes of action in certain assays [430]. ML171 (2-acetylphenothiazine), an early NOX1-based inhibitor, has also been extensively used to dissect NOX1 biology [431]. Older, nonselective flavoprotein inhibitors that suppress multiple oxidases have largely been restricted to in vitro studies because of mitochondrial toxicity and poor specificity, limiting their clinical utility [202, 432]. Collectively, these compounds illustrate the evolution of NOX inhibition from broad-spectrum oxidase inhibitors towards increasingly isoform-selective agents with therapeutic potential for oxidative stress-driven disease.

#### Setanaxib (GKT137831)

Setanaxib (GKT137831), a dual NOX1/4 inhibitor and the first NOX-targeted compound to advance into clinical trials, suppresses ROS generation from NOX1 and NOX4 [426]. Numerous preclinical studies have demonstrated that pharmacological inhibition of NOX1 and NOX4 with GKT137831 attenuates oxidative stress, cardiac remodeling, fibrosis, and cardiotoxicity, highlighting its therapeutic potential in cardiovascular diseases [314, 411, 433, 434]. Compared with treatment with doxorubicin alone, pretreatment with GKT137831 in models of doxorubicin-induced cardiotoxicity reduces the number of TUNEL-positive cardiomyocytes and decreases the levels of cleaved-PARP and cleaved-caspase-3, suggesting that the cardioprotective effects of GKT137831 occur through the attenuation of oxidative stress-linked MAPK signaling [92, 411]. In ocular disease models, setanaxib exerts pronounced neuroprotective and anti-inflammatory effects. In experimental ischemic retinopathies, NOX1/4 inhibition with setanaxib reduces hypoxia-induced ROS (as measured by DHE signaling), dampens microglial and Müller cell activation, decreases the gene and protein expression of VEGF and leukocyte adhesion molecules, and mitigates vascular leakage [435, 436]. More recently, a retinal ischemia-reperfusion study in mice revealed that setanaxib decreased the expression of markers of oxidative stress, apoptosis, and senescence in retinal ganglion cells while improving structural and functional outcomes [437]. In pulmonary fibrosis, setanaxib has demonstrated consistent antifibrotic activity in both bleomycin-induced and age-associated models. Targeting NOX4 with GKT137831 reduces myofibroblast accumulation, collagen deposition (including lung hydroxyproline content), and α-SMA expression and partially reverses persistent fibrosis and senescence markers (p16 and p21) in aged mice [438]. Taken together, these findings demonstrate that setanaxib attenuates NOX1/4-driven ROS generation, apoptosis, and fibrotic remodeling across cardiac, retinal, and pulmonary systems, supporting the therapeutic potential of dual NOX1/4 inhibition in oxidative stress-driven pathology.

#### APX-115 (pan-NOX inhibitor)

APX-115 (3-phenyl-4-propyl-1-(pyridin-2-yl)-1H-pyrazol-5-ol hydrochloride) is an orally active pan-NOX inhibitor (NOX1/2/4) that, in head-to-head diabetic kidney disease models, suppresses renal NOX1, NOX2, and NOX4 expression and multiple injury readouts more effectively than the dual NOX1/4 inhibitor setanaxib [439]. In aging diabetic mice, compared with no treatment, APX-115 treatment significantly reduces mesangial expansion, decreases the glomerular area, attenuates macrophage infiltration (F4/80^+^ cells), and decreases collagen-IV expression [385]. In both type 1 and type 2 diabetic nephropathy models, pan-NOX inhibition with APX-115 consistently improved structural kidney injury and preserved renal function, supporting its renoprotective and anti-inflammatory potential in NOX-driven kidney disease [440].

#### VAS2870 (pan-NOX tool compound)

VAS2870 is a pan-NOX inhibitor with varying potency across isoforms [202, 441]. It exerts its inhibitory effect at least in part, through covalent alkylation of a cysteine residue within the dehydrogenase domain of the nicotinamide-binding region of the enzyme. Consistent with this inhibitory activity, VAS2870 has also been shown to suppress NOX-dependent ROS generation in cellular models [442, 443]. Several studies suggest that VAS2870 is a promising therapeutic compound for managing NOX-related pathologies. In LPS-stimulated human alveolar epithelial A549 cells, VAS2870 inhibited the LPS-induced upregulation of NOX2 expression, reduced NOX2-derived ROS generation, restored epithelial barrier integrity by preserving the distribution of ZO-1 and tight junction proteins, and preserved cell viability through the NOX2/ROS/ZO-1 axis [222, 430, 444, 445]. These findings suggest that VAS2870-mediated suppression of NOX2 activity and downstream ROS generation may represent a viable therapeutic strategy for LPS-induced acute respiratory distress syndrome (ARDS). These findings indicate that VAS2870 protects the alveolar epithelial barrier by inhibiting NOX2-induced ROS generation and preserving tight junction protein integrity [430]. In another study, VAS2870 and its analog VAS3947 attenuated platelet activation and thrombus formation in mouse thrombosis models; the results revealed that VAS2870 delayed the time to occlusive thrombus formation and reduced platelet aggregation [430]. Although VAS2870 has demonstrated therapeutic efficacy in numerous preclinical models, it is generally considered a research tool rather than an optimized therapeutic agent, due to its limitations related to specificity and pharmacological development.

#### TG15-132

TG15-132 is a potent and selective NOX2 inhibitor that effectively suppresses superoxide generation in activated microglia and endothelial cells. It demonstrates excellent brain penetration, favorable pharmacokinetic properties, and no observable toxicity in mice. Additionally, it reduces NOX2-derived ROS and inflammatory gene expression in vitro, indicating potential anti-inflammatory and neuroprotective activity [446]. TG15-132 is a brain-permeable NOX2 inhibitor that has a relatively long plasma half-life (5.6 h) and a high brain-to-plasma ratio (>5-fold) in rodents, with no detectable systemic toxicity after repeated dosing [446]. These properties support its development as a NOX2-targeted anti-inflammatory and neuroprotective candidate, particularly in disorders in which microglial and myeloid NOX2 activity drives oxidative stress and neurodegeneration. In vitro, TG15-132 suppressed NOX2-derived ROS and downregulated NOX2 subunit (CYBB/gp91^*phox*^) as well as proinflammatory cytokine gene expression in human myeloid cells, further supporting its potential as an anti-inflammatory and neuroprotective agent [446].

### Peptide inhibitors

Peptide-based inhibitors of NOX, including the p47^*phox*^/p22^*phox*^ and NOXA1-NOX1 interfaces, primarily act by disrupting the essential protein-protein interactions required for enzyme assembly [447]. Prototypical examples include NOX2ds-tat, which attenuates LPS-induced lung injury and ischemia-reperfusion damage in vivo [448], and recently developed NOXA1-derived peptidomimetics that inhibit NOX1-dependent O_2_^•−^ formation in cells and show antithrombotic efficacy after stabilization of the peptide scaffold [449]. Complementary work has revealed that low-molecular-weight ligands that compete with p22^*phox*^ for the p47^*phox*^ SH3 groove, such as compounds C6 and C14, inhibit NOX2 with submicromolar IC₅₀ values (~1 µM), reduce NOX2-derived oxidative stress in cardiomyocytes, and protect against ischemia-reperfusion-induced cardiac injury in mice, validating p47^*phox*^ as a druggable PPI hub for NOX2 inhibition [450]. Current pharmacological strategies targeting NOX isoforms, including isoform-selective inhibitors, peptides, and redox-active nanotherapeutics, are summarized in Table 5. Together, these data indicate that targeting NOX activation via PPI interfaces using stabilized peptides or peptide-derived small molecules can modulate isoform-specific ROS signaling.

**Table 5:** NOX subunits as therapeutic targets

NOX isoform/subunit	Inhibitor/modulator	Mechanism of action	Biological model/system	Functional outcome	Therapeutic implications	References
NOX2	S100A8/A9 inhibitor (ABR-238901)	Inhibits S100A8/A9-driven TLR4-dependent induction of NOX1/2/4, reducing NADPH oxidase-derived ROS, NF-κB activation, and NLRP3 inflammasome priming	Male C57BL/6J mice, RAW 264.7 mouse Mac cell line and Human THP-1 monocytic cell line	Reduced oxidative stress, suppressed the inflammasome, and improved myocardial cell survival.	Targeting S100A8/A9 prevents NOX2-driven ROS and inflammation in ischemic myocardium	[542]
	Fyn kinase inhibitor	Inhibition of Fyn kinase prevents phosphorylation of PKCδ, leading to reduced NF-κB-mediated transcription of proinflammatory cytokines, iNOS, and NOX2 in microglia	PKCδ knockout (KO) mice and primary microglial cultures	Decreased oxidative stress and neuroinflammation	Targeting the SFK-iNOS-NOX2 axis may offer therapeutic benefits in neurodegenerative diseases by modulating microglial activation and reducing ROS generation	[543]
	MPO and MPO-NP oral administration	MPO is a mitochondrial-targeted NADPH oxidase inhibitor that reduces oxidative stress by decreasing the production of free radicals	Sprague Dawley rat model of organophosphate-induced neurotoxicity, DFP exposure	MPO-NP and MPO-oral treatments led to significant weight loss, abnormal liver and kidney parameters, and elevated gp91^*phox*^ expression and astrocyte activation in the brain	While MPO effectively mitigates DFP-induced reactive astrogliosis and protects neurons, the study highlights the complexity of dosing regimens and the potential for off-target effects when combining DFP and MPO treatments	[544]
	Saracatinib	Inhibits Src kinases → reduces phosphorylation-dependent activation of microglia/astrocytes → ↓ iNOS, ↓ NOX2 (gp91^*phox*^) expression → ↓ ROS and nitro-oxidative stress	Sprague-Dawley rats	Reduced microgliosis, astrogliosis, neurodegeneration, and markers of nitro-oxidative stress; no significant improvement in behavioral deficits	Targeting the Src-NOX2 axis may reduce neuroinflammation and oxidative stress, providing a disease-modifying approach in OP-induced or other ROS-driven neurodegenerative conditions	[545]
	gp91^*phox*^ knockout (genetic deletion)	Eliminates gp91^*phox*^ expression, attenuated NOX2-mediated oxidative stress and ferroptosis	Male C57BL/6 mice	Enhanced cognitive function, preserved neuronal integrity	Genetic deletion of gp91^*phox*^ offers insights into NOX2’s role in neurodegenerative processes	[546]
	Compound 9a (VAS2870 derivative)	Covalent inhibition of NOX2 through binding to the conserved catalytic cysteine (Cys668); dual MAO-B inhibition	PLB-985-derived NOX2 assays; BV2 microglial cells	Reduced NOX2-derived ROS and decreased iNOS, IL-1β, and IL-6 expression	Potential therapies for neuroinflammation, oxidative stress disorders	[547]
	siRNA-mediated knockdown	Reduces expression of NOX2 in HUVECs	HUVECs	Abrogates histamine- and VEGF-induced H₂O₂ production	Potential therapeutic approach for modulating endothelial oxidative stress	[548]
NOX4	S100A8/A9 inhibitor (ABR-238901)	Blockade of S100A8/A9 signaling attenuates NOX4 expression → ↓ mitochondrial and cytosolic ROS → ↓ NLRP3 inflammasome priming.	Male C57BL/6J mice, RAW 264.7 mouse Mac cell line and Human THP-1 monocytic cell line	Decreased ROS burden, preserved cardiac tissue function	Inhibition of the S100A8/A9-NOX4 axis could protect against oxidative damage and fibrosis in ischemic heart disease	[542]
	GLX351322	Inhibits NOX4 → reduces mitochondrial oxidative stress → enhances mitochondrial membrane potential (Δψm), ATP/ADP ratio, and insulin secretion in response to high glucose	EndoC-βH1 human beta-cell line and human islets	Increased mitochondrial function and insulin release; protection against cell death under glucolipotoxic conditions	NOX4 inhibition may be a therapeutic strategy in type 2 diabetes to protect beta-cell function and survival	[549]
	GLX7013114/ Lenvatinib, GLX351322	Inhibits NOX4 → reduces ROS generation → activates Nrf2 signaling → preserves mitochondrial function and reduces apoptosis in renal tubular cells	Male C57BL/6 J mice	Improved GFR, reduced BUN levels, decreased tubular injury and apoptosis, and preserved mitochondrial function	NOX4 inhibition during reperfusion is a potential therapeutic strategy to mitigate ischemia-reperfusion-induced AKI	[550]
		Inhibits NOX4 → reduces ROS generation → disrupts TGFβ1/SMAD3 signaling → attenuates ERK and PI3K/AKT pathways → enhances apoptosis and sensitizes cells to chemotherapy and lenvatinib	Human BCPAP and TPC-1 PTC cell lines	Decreased cell viability, increased apoptosis, prolonged G1 phase arrest, and altered energy metabolism	NOX4 inhibition can overcome resistance to chemotherapy and lenvatinib in PTC by modulating redox signaling and cell survival pathways	[551]
	GKT137831 (S7171)	Inhibits NOX4 → reduces ROS generation and NF-κB activation → attenuates mitochondrial dysfunction, inflammation, and apoptosis in renal tubular epithelial cells	Male C57BL/6J mice and Mouse kidney tubular epithelium cells (TCMK-1)	Improved renal function, reduced tubular injury, decreased inflammation, preserved mitochondrial integrity	NOX4 inhibition serves as a potential novel therapeutic strategy for S-AKI by targeting oxidative stress and mitochondrial dysfunction	[552]
NOX1	GKT137831 (Setanaxib)	Inhibits NOX4 → reduces ROS generation → activates AKT/CREB signaling → increases SMN expression in motor neurons	SMA-like mice (SMN2^+/−^) and SMA-like mice (FVB/NRj-SMN^Δ7/Δ7^, huSMN2^+/−^)	Enhanced motor neuron survival, improved motor behavior, and increased lifespan	NOX4 inhibition is a potential complementary therapeutic strategy to SMN-upregulating treatments in SMA	[553]
	ML171	Inhibits NOX1	Human-derived HCC cell lines, HepG2, HCC-LM3 (LM3) and the human normal hepatocyte cell line LO2	Sensitized HCC cells to sorafenib and radiotherapy; promoted ROS-mediated programmed cell death; suppressed tumor growth	NOX1 inhibition is a potential complementary therapeutic strategy to enhance the efficacy of sorafenib and radiotherapy in HCC patients	[554]
NOX2	VAS2870, MAOB inhibitor	Covalent, preferential inhibition of preactivated NOX2 through targeting of a conserved active-site cysteine; also covalently inhibits MAOB by forming an adduct with FAD	PLB-985 cells; LPS/IFN-γ-stimulated BV2 microglial cells	Potent NOX2 inhibition; reduced ROS and decreased iNOS, IL-1β, and IL-6 expression in stimulated BV2 microglia	First-in-class dual covalent NOX2/MAOB inhibitor; potential lead for targeting NOX2- and MAOB-associated oxidative stress and neuroinflammatory/neurodegenerative processes	[547]

*AKI* acute kidney injury, *BCPAP* B-Papillary Thyroid Carcinoma, *BUN* blood urea nitrogen, *DUOX* dual oxidase, *GFR* glomerular filtration rate, *gp91* phox catalytic subunit of NOX2, *HUVECs* human umbilical vein endothelial cells, *iNOS* inducible nitric oxide synthase, *LPS* lipopolysaccharide, *MPO* mitoapocynin, *MPO-NP* MPO encapsulated in polyanhydride nanoparticles, *NADPH* nicotinamide adenine dinucleotide phosphate, *NF-κB* nuclear factor kappa B, *NLRP3* NOD-, LRR-, and pyrin domain-containing protein 3, *NOX* NADPH oxidase, *PTC* papillary thyroid cancer, *ROS* reactive oxygen species, *S-AKI* sepsis-associated acute kidney injury mouse model, *siRNA* small interfering RNA, *TCMK* transformed C3H mouse kidney-1, *TPC-1* thyroid papillary carcinoma 1, *VEGF* vascular endothelial growth factor, *AKT* protein kinase B, *ATP* adenosine triphosphate, *C57BL/6J C57BL/6J* mouse strain, *EndoC-βH1* human pancreatic beta-cell line, *ERK* extracellular signal-regulated kinase, *FAD* flavin adenine dinucleotide, *H2O2* hydrogen peroxide, *HCC* hepatocellular carcinoma, *HCC-LM3* human hepatocellular carcinoma cell line, *IFN-γ* interferon gamma, *IL-1β* interleukin-1 beta, *IL-6* interleukin-6, *KO* knockout, *L02* human normal hepatocyte cell line, *PKCδ* protein kinase C delta, *RAW 264.7* murine macrophage cell line, S100A8/A9 S100 calcium-binding proteins A8/A9, *SMAD3* SMAD family member 3, *SMN* survival motor neuron, *TGFβ1* transforming growth factor beta 1, *THP-1* human monocytic leukemia cell line, *TPC-1* human papillary thyroid carcinoma cell line, *Δψm* mitochondrial membrane potential

### Gene silencing: siRNA/shRNA against NOX2/p47^*phox*^

Silencing NOX components significantly reduces ROS generation. In particular, NOX2 (CYBB/gp91^*phox*^) silencing using siRNA or shRNA consistently suppresses NOX2-dependent ROS generation and downstream pathological responses in multiple cell and preclinical models [451]. In human hepatoma (HepG2) cells challenged with *Entamoeba histolytica*, compared with scrambled siRNA, transfection with NOX2-siRNA markedly reduced *Entamoeba*-induced ROS and decreased host-cell lactate dehydrogenase (LDH) release, demonstrating a direct cytoprotective effect of NOX2 knockdown [452]. In human microvascular endothelial cells, NOX2-specific siRNA abolished angiotensin II-stimulated upregulation of endothelin-converting enzyme-1 (ECE1) mRNA and blocked the associated increase in superoxide levels, directly linking the Ang II-NOX2-superoxide-ECE1 signaling axis [453]. Complementary p47^*phox*^-targeted silencing studies further validated this approach: in human SH-SY5Y neuronal cells, p47^*phox*^-siRNA reversed bupivacaine-induced neurotoxicity by abolishing the burst production of NOX2-derived ROS [454]; in atherosclerotic apoE^-/-^ mouse adventitial fibroblasts, p47^*phox*^-siRNA reduced NADPH oxidase activity and superoxide generation by >80% and attenuated pathological cell proliferation and migration [455]; and in nonphagocytic engineered HEK293 cells, p47^*phox*^-siRNA abolished PMA-stimulated ROS generation, confirming its nonredundant role in NOX2 complex activation [456]. Collectively, these findings identify NOX2 and its regulatory subunit p47^*phox*^ as a proximal, druggable source of pathological ROS and support siRNA/shRNA approaches as valuable target validation tools and potential therapeutic strategies.

### ROS modulators

Several naturally derived antioxidant compounds have been shown to modulate NOX expression or activity in preclinical models; however, their effects on individual NOX isoforms are often context-dependent. This section focuses on how N-acetylcysteine and anthocyanins influence NOX-driven ROS signaling and related pathophysiological processes across different tissues.

N-acetylcysteine (NAC) remains one of the most widely used antioxidants for modulating NOX-dependent oxidative stress in experimental models. Although not a direct enzyme inhibitor, NAC replenishes intracellular glutathione and scavenges ROS, thereby disrupting redox-sensitive signaling loops that sustain NOX expression and activity. In LPS-driven neuroinflammatory models, NAC reduced LPS-induced NOX2 upregulation and ROS accumulation in neuron-glia midbrain cultures and protected against neuronal injury in vivo [243]. Similarly, in inflammatory macrophage models relevant to periodontitis, NAC reversed N-acetyltransferase 10 (NAT10, an RNA cytidine acetyltransferase involved in ribosome biogenesis and mRNA stabilization)-driven NOX2 expression and downstream NF-κB activation, further reducing inflammatory cytokine production [243]. In osteoarthritic chondrocytes, NAC inhibited IL-1β-induced NOX2-dependent ROS generation and MAPK activation, thereby mitigating apoptosis and prostaglandin E₂ release [457]. Similarly, in mesenchymal stem cells, NAC reduced LPS-induced ROS and NLRP3 inflammasome activation by suppressing the TXNIP/NOX axis [458]. Collectively, these findings demonstrate that NAC indirectly reduces NOX-derived ROS levels and associated inflammatory or degenerative responses by restoring cellular redox homeostasis and interrupting ROS-mediated feedback loops that amplify NOX signaling.

Anthocyanins, particularly malvidin- and petunidin-class compounds, and anthocyanin-rich berry extracts consistently reduce NOX expression and NOX-dependent ROS generation in experimental vascular and cardiac models. Blueberry anthocyanin extract and isolated malvidin derivatives prevented high-glucose-induced NOX4 upregulation and ROS accumulation in human umbilical vein endothelial cells [459]. In cardiac ischemia and anoxia-reoxygenation models, anthocyanin derivatives (including petunidin and malvidin species) attenuate myocardial injury, reduce apoptosis markers, and suppress oxidative and inflammatory signaling, with several studies reporting concomitant decreases in NOX4 expression or activity in cardiomyocytes and perfused hearts [459, 460]. These cellular and ex vivo/in vivo studies support a model in which anthocyanins blunt NOX4 (and in some settings NOX2) induction under pathological stimuli, thereby lowering ROS levels, limiting MAPK/NF-κB activation, and reducing cell death.

### Combination therapies: NOX inhibitors and other pathway-targeted agents

Emerging preclinical evidence indicates that combining NOX inhibition with oncogenic, inflammatory, or fibrotic pathway inhibitors can produce synergistic therapeutic effects across multiple disease models. In solid tumors enriched in cancer-associated fibroblasts (CAFs), pharmacologic NOX4 inhibition with setanaxib or NOX4 knockdown reprogrammed CAFs toward a less activated phenotype, normalized the stromal architecture, increased intratumoral CD8⁺ T-cell infiltration, and restored responsiveness to anti-PD-1 immunotherapy in otherwise resistant murine tumors [141]. Likewise, in non-small cell lung cancer, NOX4 inhibition synergized with EGFR-targeted tyrosine kinase inhibitors to overcome drug resistance by blocking NOX4 -YY1-IL-8-PD-L1 signaling axis, which supports EGFR-TKI resistance; genetic or pharmacologic NOX4 inhibition downregulated YY1, IL-8, and PD-L1 expression, resensitized resistant cells to EGFR inhibitors, and produced synergistic antitumor effects when NOX4 was combined with gefitinib or osimertinib in vitro and in vivo [371]. In hematologic malignancies, particularly AML, dual NOX1/4 blockade with setanaxib enhanced daunorubicin-induced cytotoxicity, increased apoptosis, and reduced leukemic burden in preclinical models, which is consistent with a redox-modulating, chemosensitizing role for NOX1/4 inhibition [461]. The above findings show that targeting other pathways along the NOX pathway represents a rational therapeutic approach for managing chronic and malignant diseases.

### Preclinical NOX inhibitor studies in animal models

Preclinical studies utilizing compounds such as setanaxib and APX-115, together with research tool compounds such as VAS2870, have generated important proof-of-concept data in cardiac, renal, hematologic, and tumor models. Notably, these foundational studies were conducted exclusively in rodents. In a murine doxorubicin cardiotoxicity model, oral setanaxib (60 mg/kg/day) administered throughout a 6-week protocol attenuated doxorubicin (DOX)-induced cardiac injury [411]. Compared with those in the control group, the myocardial ROS levels (DHE fluorescence and 4-HNE), levels of apoptotic markers, including cleaved-PARP and cleaved-caspase-3, and TUNEL-positive cell counts in the cardiac tissue in the treated group were reduced, and histological fibrosis was reduced, and echocardiographic indices of systolic function were preserved in the treated group. Survival improved from 55% (11/20) in the DOX group to 65% (13/20) in the DOX + setanaxib group, although this difference was not statistically significant [411]. The pan-NOX inhibitor APX-115 produced durable renal protection in NOX5 transgenic and diabetic rodent models when it was orally administered at 60 mg/kg/day for extended periods, approximately 12-14 weeks [429]. The results revealed that APX-115 ameliorated albuminuria, significantly reduced urinary albumin-to-creatinine ratios, improved metabolic parameters such as lower fasting glucose levels, improved insulin measures, corrected dyslipidemia, and restored podocyte markers and renal architecture compared with those in vehicle-treated diabetic animals; these findings are consistent with the suppression of NOX-driven renal oxidative injury [429, 439, 440]. VAS2870, an early pan-NOX research compound, demonstrated redox-modulatory effects in vivo in a murine UV-induced mesenteric venule microthrombosis model. A single intravenous dose of 3.7 mg/kg increased the time to occlusive thrombus formation in mesenteric venules and inhibited agonist-induced platelet aggregation in response to agonists, with a reduction of approximately 40%. Mechanistic analyses indicated that the compound’s antithrombotic actions were at least partly mediated downstream of PKC via IKKβ/p38 MAPK signaling rather than exclusively by direct NOX catalytic blockade [430]. Importantly, preclinical cancer studies have shown that NOX1/4 inhibition with setanaxib reprograms the tumor stroma [15, 141, 462, 463]. In CAF-rich murine tumors, GKT137831 converted myofibroblastic cancer-associated fibroblasts to a less immunosuppressive phenotype, increased intratumoral CD8⁺ T-cell infiltration, and restored responsiveness to anti-PD-1 therapy in otherwise resistant tumors. This combined effect reflects a dual mechanism: GKT137831 acts on the tumor stroma by inhibiting NOX4-driven ROS generation in CAFs, normalizing their activated phenotype and dismantling the physical barrier responsible for CD8⁺ T-cell exclusion; simultaneously, the resulting intratumoral T-cell infiltration creates a permissive immune environment in which anti-PD-1 checkpoint blockade can reactivate cytotoxic CD8⁺ T cells that would otherwise remain suppressed [141]. Neither intervention alone is sufficient in CAF-rich tumors-GKT137831 permits immune cell entry-while anti-PD-1 therapy restores their effector function once inside, providing a mechanistic rationale for their synergistic combination to overcome ROS-driven stromal immune exclusion.

## Computational modeling and systems biology approaches

The diverse roles of NOX isoforms in health and disease, together with their isoform-specific regulation and interactions with multiple signaling pathways, require an integrated computational and systems biology approaches for effective mechanistic understanding and therapeutic development. Traditional methods often fail to capture the full complexity of NOX regulation and focus mainly on isoform-specific dynamics, post-translational modifications, and crosstalk with other signaling networks. Computational modeling, including molecular docking, molecular dynamics, and artificial intelligence, provides structural predictions that provide descriptive insights into enzyme-ligand interactions, molecular dynamics, and inhibitor mechanisms [464–466]. Deep-learning-based models of NOX1 and related isoforms, refined by docking and molecular dynamics, have been used to map FAD-, heme-, and NADPH-binding sites; predict small-molecule binding poses; and rationalize structure-activity relationships for isoform-selective inhibitors [62, 467]. Systems biology approaches integrate multiomics data to address NOX-related networks, identify regulatory nodes, and predict context dependent activation patterns under varying physiological and pathological situations. Machine learning and network pharmacology further enhance these strategies by predicting NOX activity and identifying multitarget inhibitors, respectively. Together, these approaches (i) prioritize NOX-linked targets and pathways from large biological and chemical datasets and (ii) predict multitarget drug combinations that optimally modulate NOX activity within broader disease networks [468, 469]. Collectively, these computational and systems approaches provide a mechanistic and predictive framework for rational NOX-targeted drug design and for translating NOX biology into clinically actionable interventions.

### Molecular docking of NOX inhibitor complexes

Molecular docking is now a routine first step for exploring how small molecules may interact with NOX enzymes and for generating testable hypotheses that can subsequently be evaluated using biochemical and cellular assays. NOX enzymes are membrane-spanning hemoproteins with a multidomain architecture, consisting of an N-terminal transmembrane domain of six helices containing two heme b groups and a C-terminal cytosolic dehydrogenase domain that binds FAD and NADPH [9, 470–472]. Because of this structural complexity, in silico investigations of NOX enzymes require careful model construction (including prosthetic groups), a docking strategy tailored to membrane proteins, and orthogonal biophysical and biological validation.

A robust NOX docking workflow typically involves three logical stages:

1. Model building that accurately captures the transmembrane heme environment and cytosolic dehydrogenase domain remains a significant challenge, since high-resolution experimental structures for full-length human NOX isoforms remain limited. Consequently, most contemporary NOX docking studies employ homology models, AlphaFold-predicted models, or hybrid constructs that combine experimentally resolved cores with predicted transmembrane regions. The incorporation of prosthetic groups (heme groups, FAD, NADPH, where relevant) as well as resolved lipids or membrane mimetics in the initial model is essential because these prosthetic groups shape the electrostatic and steric environment of plausible ligand sites; their omission can lead to artifactual docking poses. Illustrative examples include the NOX1 structural model and docking pipeline described by Liu and colleagues, who used predicted 6-TM plus dehydrogenase models to map cofactor pathways and probe inhibitor interactions [467].
2. Docking should be conducted using strategies appropriate for the intended mechanism of inhibition (noncovalent and covalent, where applicable) and with docking tools suited for membrane proteins and flexible binding sites (e.g., AutoDock Vina, Glide, and GOLD). In addition, ensemble docking across multiple receptor conformations should be considered to account for the uncertainty in TM helix packing. If an inhibitor is suspected to act covalently (e.g., via cysteine alkylation), dedicated covalent docking protocols or reactive docking workflows are needed rather than a standard noncovalent protocol, as covalent mechanisms demand explicit treatment of warhead chemistry and reaction geometry. Candidate compounds should also be screened for redox-active and assay-interfering chemotypes, as many historically reported NOX “inhibitors” function primarily as ROS scavengers or nonspecific thiol-reactive agents rather than bona fide active-site ligands [473].
3. Refinement and validation of top hits using molecular dynamics (MD), free-energy calculations, and experimental approaches. Top-ranked docking poses should be refined through MD in an explicit membrane environment (e.g., POPC/cholesterol bilayer) to evaluate binding pose stability and to examine potential effects on channel gating, heme accessibility, or ligand-translocation pathways. Relative binding affinities may be estimated using end-point methods (MM-PBSA/MM-GBSA), whereas high-priority candidates may be further evaluated using more rigorous alchemical free-energy methods, such as free-energy perturbation (FEP) or thermodynamic integration (TI) [474]. Computational predictions should subsequently be validated experimentally using orthogonal approaches: recombinant isoform panels (to assess isoform selectivity), biochemical assays measuring heme-proximal electron transport or NADPH consumption, site-directed mutagenesis (e.g., cysteine residues), washout and reversibility studies, and cellular ROS assays to confirm functional NOX inhibition [475–477]. Established MD and free-energy protocols are now standard practices for advancing docking hits toward robust mechanistic conclusions.

The importance of rigorous validation is underscored by the cautionary example that VAS2870 is widely used as a pan-NOX inhibitor in cell and animal studies across vascular, inflammatory, and neuronal models [445], although its precise mechanism of action remains under debate. Mass spectrometry and biochemical studies revealed that VAS2870 can alkylate cysteine residues on off-target proteins, most notably cysteine thiols within the ryanodine receptor RyR1, and modulate redox-sensitive thiols more broadly, indicating substantial off-target reactivity in vascular and inflammatory models [478]. These findings underscore why the validation stage of the docking workflow (Stage 3) must include thiol-reactivity profiling, washout and reversibility studies, and isoform-selective biochemical assays, since compounds that alkylate non-NOX cysteines can produce apparent NOX inhibition through indirect redox perturbation rather than bona fide active-site engagement. Within the computational framework described above, docking can be used to generate hypotheses about how VAS2870 might engage NOX isoforms. For example, docking may identify plausible binding pockets for VAS2870 near heme-access tunnels or transmembrane cavities that sterically or electrostatically hinder electron flow or indicate proximity to conserved cysteines that might undergo covalent modification. However, docking poses are only suggestive and cannot by themselves establish a covalent mechanism, target engagement, or physiological relevance. Reviews and methodological papers emphasize that good docking scores must be followed by biochemical isoform panels, washout/reversibility experiments, cysteine- or other site-directed mutation testing, and preferably direct binding evidence (thermal shift, SPR/ITC if a soluble construct is available) to avoid misclassifying general redox-active compounds as specific NOX inhibitors [474, 479].

### Molecular dynamics of the effects of PTMs on NOX conformation

Post-translational modifications (PTMs) act as molecular switches that reshape the dynamics, conformation, and assembly of NOX complexes. Phosphorylation of the cytosolic subunit p47^*phox*^ plays a central role in the transition of NOX isoforms from an inactive state to an active state by relieving autoinhibitory interactions and promoting their translocation to the membrane. Structural and biochemical studies have shown that phosphorylation of p47^*phox*^ at multiple serine residues disrupts its intramolecular SH3-PX domain interactions, exposing phosphoinositide-binding sites and facilitating membrane translocation and assembly with p22^*phox*^ and NOX2 [65, 480]. MD simulations have provided atomistic insights into how specific phosphorylation events drive this transition. MD and docking studies revealed that Ser379 in the C-terminal tail of p47^*phox*^ is a critical regulatory switch: phosphorylation of Ser379 disrupts hydrogen bonds linking the C-terminal tail to the SH3 domains and autoinhibitory region, allowing the tail to move away, further destabilizing the closed conformation and facilitating p22^*phox*^ binding and oxidase activation [481]. In parallel, phosphorylation of NOX1 and its activator NOXA1 has been shown to modify their binding interface and the orientation of subunits, which provides control over ROS generation [482]. Modifications of membrane subunits also contribute to regulation. For example, phosphorylation of p22^*phox*^ at threonine 147 enhances the stability of the NOX2 and p22^*phox*^ complex, further underscoring the allosteric role of PTMs in complex integrity [483]. In addition to phosphorylation, redox-based regulation can modulate NADPH oxidase assembly and activity. In human neutrophils, S-nitrosothiol treatment inhibits the membrane translocation of p47^*phox*^ and p67^*phox*^, thereby attenuating NADPH oxidase-dependent superoxide production [484]. Nitric oxide suppresses NADPH oxidase-dependent superoxide production by S-nitrosylation of p47^*phox*^ in human endothelial cells [484]. Additionally, ubiquitination of NOX4 modulates both protein turnover and redox homeostasis [485]. Recent cryo-EM structures of human NOX2 have revealed the flexibility of transmembrane helices and the coupling between the dehydrogenase (FAD/NADPH binding) and heme domains [11], providing a structural framework to interpret how PTMs, by altering charge distribution or subunit interfaces, can allosterically remodel the catalytic core. Collectively, structural, MD, and biochemical evidence supports a model in which PTMs are key determinants of NOX conformational plasticity, orchestrating enzyme activation, localization, and redox output with high spatial and temporal precision.

### AlphaFold predictions for full-length NOX isoforms

The emergence of AlphaFold 2 (AF2) has transformed the structural exploration of the NOX family by providing high-coverage, full-length models that complement experimental cryo-EM and X-ray structures and inform functional interpretation. Recent advances in in silico protein structure prediction, particularly through AlphaFold 2 and AlphaFold-Multimer, have enabled near-atomic visualization of full-length NOX isoforms, crucial in the spatial organization of transmembrane helices, FAD/NADPH-binding sites, and regulatory loops that coordinate electron transfer.

Recent studies have begun integrating AlphaFold-derived structural models along with pollutant-response data to probe environmentally driven NOX activation. For instance, molecular docking and dynamics simulations using AlphaFold-predicted NOX4 structures suggest that certain pollutants may destabilize redox-regulatory loops and FAD-binding regions [486]. Fine particulate matter (PM₂.₅) and cigarette smoke have also been associated with upregulation of NOX1 and NOX2, with AlphaFold-based models enabling mapping of key oxidation-sensitive residues along their cytosolic loops and NADPH-interacting regions [466, 487]. This convergence of structural prediction and toxicology supports a mechanistic framework in which environmental or metabolic stressors perturb redox-sensitive regions at NOX regulatory protein interfaces (e.g., RAC1/p47^*phox*^ docking surfaces), promoting the conformational activation of NOX complexes and amplifying ROS generation, inflammation, and tissue injury. Moreover, AlphaFold-based models of full-length NOX isoforms and their complexes provide structurally informed templates for the rational design and virtual screening of isoform-selective NOX inhibitors, particularly when integrated with MD and experimental validation, as outlined in the previous section.

### Machine learning (ML) for NOX activation pattern prediction from transcriptomes

Recent advances in toxicogenomics, computational biology and machine learning have opened the possibility of predicting enzyme activation states, including those of the NOX family, directly from high-dimensional transcriptomic datasets. In toxicology contexts, machine learning workflows applied to pollutant-exposed tissues or cells provide a powerful strategy for deriving “NOX-activation signatures” without relying solely on individually targeted assays. A representative example from the broader field of toxicological transcriptomics is the 2023 study by an ensemble ML approach that analyzed rat brain and placental RNA-seq data following organophosphate-ester (OPE) exposure, identifying mitochondria-transcription and cation channel-related genes as key predictive features [488]. Another recent study integrated bulk and single-cell transcriptomics with ML to dissect oxidative- stress-associated inflammation in carotid atherosclerosis [489]. Collectively, these studies illustrate the core components of this approach: (i) annotated datasets (e.g., exposed versus control) used as labels; (ii) high-throughput transcriptomic profiles as input features; (iii) ML classifiers (e.g., support-vector machine, random forest, and neural network) to distinguish activation states; and (iv) feature-importance ranking to highlight mechanistic pathways.

To model NOX isoform activation in response to environmental pollutants such as fine particulate matter (PM₂.₅), per- and polyfluoroalkyl substances (PFAS), and e-cigarette aerosols, among other airborne toxicants, a supervised machine learning framework can be developed using integrated transcriptomic and functional datasets. Transcriptomic profiles derived from relevant cell populations, including lung epithelial cells, vascular endothelial cells, and macrophages, should be collected under controlled exposure conditions, together with matched measurements of NOX isoform expression (NOX1, NOX2, and NOX4) and corresponding reactive oxygen species production. Samples can be annotated according to functional activation states, distinguishing high NOX or ROS activity from baseline conditions before being divided into training and validation cohorts. Supervised learning approaches, including random forest models, support-vector machines, and neural networks, can subsequently be trained to classify activation states on the basis of gene expression patterns. Interpretation of model features allows the identification of upstream regulatory pathways, including aryl hydrocarbon receptor signaling, intracellular calcium signaling, and RAC1-dependent NADPH oxidase complex assembly. Model performance should be assessed using independent datasets or time-course experiments, with evaluation metrics such as the area under the receiver operating characteristic curve, accuracy, and sensitivity. Once validated, the model can be applied to new exposure datasets or experimental systems to enable in silico screening and stratification of pollutant conditions that drive NOX-mediated oxidative responses [490, 491].

A major advantage of this ML-based paradigm is its scalability across pollutants and tissues, enabling data-driven prediction of NOX activation rather than relying solely on targeted, hypothesis-driven assays. However, a major limitation in the current NOX toxicology literature is the lack of publicly accessible transcriptome datasets annotated for NOX isoform activity. To accelerate progress, the field would benefit from establishing shared datasets that integrate pollutant exposures, transcriptomics, and NOX/ROS readouts, as well as from applying ML to construct generalized “NOX-response classifiers”.

### Network pharmacology for multitarget inhibitors within NOX-mediated redox networks

While much of NOX inhibitor discovery has historically focused on single isoforms, the emerging paradigm of network pharmacology recognizes that pollutant-driven oxidative stress arises not from an isolated target but from a web of interacting nodes: NOX isoforms (e.g., NOX2 and NOX4), their regulatory subunits (p22^*phox*^ and p47^*phox*^), upstream activators (RAC1 and PKC), ROS-responsive signaling pathways (the NLRP3 inflammasome, NF-κB, and MAPK), and downstream mediators of injury (cytokines and cell-death pathways). Network pharmacology aims to systematically map these interactions and identify compounds capable of modulating multiple nodes simultaneously. For instance, a 2023 study identifying a novel brain-permeable NOX2 inhibitor (TG15-132) demonstrated in cell-based assays that the compound not only acutely inhibited NOX2-dependent superoxide and hydrogen peroxide production in differentiated HL-60 cells but also downregulated the expression of transcripts encoding NOX2 subunits, multiple inflammatory cytokines, and iNOS in THP-1-derived myeloid cells upon longer exposure, effectively dampening a broader inflammatory network [492]. Similarly, the NOX2 inhibitor GSK2795039 prevented chronic doxorubicin-induced cardiac atrophy and dysfunction in rabbits by attenuating cardiac sympathetic nerve- terminal abnormalities, reducing myocyte autophagy, and improving left ventricular function, indicating that NOX2 blockade can beneficially rewire redox, neuronal, and autophagy pathways in the failing heart [306]. In a NOX-centric environmental toxicology context, a network-pharmacology workflow can be conceptualized as follows: (a) construct a protein-protein interaction (PPI) network centered on NOX isoforms, regulatory subunits, upstream activators (RAC1, p47^*phox*^), ROS-sensitive transcription factors (NF-κB, AP-1), inflammasome components (NLRP3), and cytokines (IL-1β, IL-18) [1]; (b) integrate pollutant-exposure transcriptomic and proteomic datasets to identify which nodes are dysregulated under each exposure for example, PM₂.₅ exposure has been shown to induce NOX1 and NOX4 expression and ROS generation in airway epithelial cells, while PFAS exposure broadly dysregulates oxidative stress and inflammatory signaling networks [180, 493, 494]; (c) use compound-target databases (natural products, repurposed drugs) to identify molecules capable of modulating multiple nodes within the network (e.g., inhibit NOX, suppress RAC1 activation, block NLRP3) [495]; (d) prioritize multitarget candidates through molecular docking to NOX regulatory domains (for instance, docking to RAC1-p47^*phox*^ interface) and downstream nodes [496]; (e) validate the leads experimentally in pollutant-exposure models and refine the network model accordingly. This systems-level approach is particularly well suited for studying environmental redox injury, where pollutants trigger complex cascades rather than a single event, and inhibition of one node often leads to compensatory activation elsewhere. By adopting a system-level, network-informed inhibitor search, researchers can comprehensively identify more robust therapeutic candidates that inhibit the NOX-ROS-inflammation axis. A current challenge is that most network pharmacology studies remain disease-oriented (e.g., cancer, metabolic syndrome) rather than explicitly addressing pollutant-driven oxidative stress. Thus, future work should extend these frameworks to pollutant-exposure models, integrate multiomics datasets with PPI networks, and prioritize multitarget modulators tailored to environmental-NOX activation pathways.

## Extracellular vesicles, lipid rafts, and NOX-linked diagnostics

Exosomes carry distinct molecular fingerprints reflective of their cellular origin and pathophysiological state. A defining feature of exosome membranes is their enrichment in lipid rafts, which are dynamic, cholesterol-rich and sphingolipid-rich microdomains that serve as signaling hubs and sorting platforms for specific proteins. This lipid microdomain organization is critical for exosome biogenesis, cargo loading, and intercellular communication, thereby offering a unique opportunity for clinical diagnostics based on lipid-raft-associated markers [497, 498]. The rafts are enriched in cholesterol, sphingomyelin, saturated phospholipids, glycosphingolipids (e.g., GM1 and GM3), and raft-resident proteins such as flotillin-1/2, caveolin-1, stomatin, and glycosylphosphatidylinositol (GPI)-anchored proteins. These components are selectively incorporated into exosome membranes during endosomal maturation, particularly within multivesicular bodies (MVBs), reflecting the microdomain structure of the donor cell [499]. Proteomic profiling studies consistently identify raft scaffolds as dominant components of exosome protein signatures, confirming their stability and diagnostic value across diverse sample types, including plasma, urine, cerebrospinal fluid (CSF), and BAL [500]. The diagnostic potential of exosome lipid-raft profiling lies in its ability to provide both disease specificity and stability. Unlike soluble biomarkers, raft proteins and lipids are protected within the exosome bilayer, making them highly resistant to degradation and therefore well suitable for long-term storage and repeated analysis. Moreover, changes in raft composition mirror pathological alterations in membrane trafficking and redox balance. For instance, altered caveolin-1 and flotillin-2 levels in plasma-derived exosomes have been reported in patients with Alzheimer’s disease and lung carcinoma and are correlated with altered cholesterol homeostasis and redox imbalance [497, 498]. Similarly, ceramide-rich exosomes serve as signatures of apoptosis and cellular stress, while altered ganglioside content has been linked to tumor progression and drug resistance [501]. Integration of multiomics datasets (proteins, lipids, and miRNAs) from raft-enriched exosome fractions enhances both sensitivity and specificity for disease diagnosis. Computational models combining raft-protein intensity with lipidomic signatures can classify cancers, neurodegenerative disorders, and metabolic diseases with >90% accuracy [502]. Such combinatorial biomarker panels may soon enable point-of-care liquid biopsy assays for early disease detection and therapeutic monitoring.

Importantly, standardization of EV isolation, characterization, and reporting remains a major challenge. Lipid composition and raft marker abundance vary depending on the isolation technique, centrifugation speed, sample type, and even the anticoagulant used. Adherence to MISEV2023 guidelines and implementation of internal reference standards (e.g., exosome-mimetic liposomes spiked with known markers) are strongly recommended for clinical validation [503, 504]. Furthermore, interlaboratory harmonization of lipidomic pipelines and quantitative reporting of raft-protein ratios (e.g., flotillin/CD9 and caveolin/CD63) will enhance reproducibility and regulatory acceptance for diagnostic applications [505–507]. Overall, exosome profiling with a focus on lipid-raft markers represents a powerful and minimally invasive diagnostic tool for disease detection, prognosis, and treatment monitoring. By linking the molecular architecture of exosomes to cellular pathophysiology, lipid-raft profiling provides a translational bridge between basic membrane biology and personalized medicine.

EVs provide a practical readout of the cellular redox state, as both their release and cargo composition are regulated by redox-sensitive biogenesis pathways and by upstream enzymatic sources of ROS, such as NOX isoforms. Mechanistic studies have established that key pathways involved in the biogenesis of exosomes, such as Endosomal Sorting Complex Required for Transport (ESCRT) components, neutral sphingomyelinase/ceramide pathways, and Rab GTPases, which control EV formation and release and are modulated by oxidative signals, link NOX activity to EV output [508]. Lipid-microdomain (raft) composition is a stable feature of many exosomes and is associated with the accumulation of signaling proteins and redox enzymes, as discussed earlier. Lipidomic and proteomic mapping have shown enrichment of cholesterol, sphingomyelin, ceramides, and raft scaffolds (flotillins and caveolins) on exosome membranes, providing robust markers for EV subtyping in redox and NOX studies [498]. Tumor cells can package NOX components or NOX-regulated cargo into EVs. Recent work has demonstrated that NOX1 is present in tumor-derived exosomes and that NOX1-carrying exosomes stimulate ROS generation and M2 macrophage polarization in cervical cancer models, highlighting a direct EV-mediated mechanism through which NOX signaling remodels the tumor microenvironment [58]. More broadly, exosome raft composition (e.g., flotillin- and caveolin-enriched domains) may serve as a surrogate marker of cellular redox status and NOX-dependent signaling activity in aging, neurodegeneration, and cancer, making raft-enriched EV fractions a logical compartment to investigate for NOX-related diagnostic markers [509]. Complementing these observations, recent work from Begum and colleagues revealed that proteasomes are compartmentalized within lipid rafts and actively packaged into exosomes under e-cigarette vapor exposure, linking raft remodeling, proteostasis, and vesicle cargo composition in a redox-associated cellular response to vaping-induced stress [510]. This compartmentalization provides an additional layer of biomarker information, as changes in exosome proteasome content and associated raft proteins can be linked to both oxidative stress and altered protein-degradation capacity in exposed cells [510].

EV-associated miRNAs are particularly promising noninvasive biomarkers of NOX activity and oxidative stress because redox perturbations consistently change miRNA packaging and abundance. In airway models, cigarette smoke-induced oxidative stress increases the release of miR-21-enriched exosomes from bronchial epithelial cells, altering fibroblast activation and profibrotic signaling and functionally linking environmental oxidants, EV cargo, and tissue remodeling [511]. Epidemiological and mechanistic environmental studies report that particulate air pollution exposure remodels plasma/serum EV counts and EV-miRNA content (for example, altered let-7, miR-143, and miR-218 families) and links EV-miRNA changes to coagulation and inflammatory endpoints, supporting the use of EVs as exposure biomarkers that reflect pollutant-driven NOX/ROS biology [512, 513].

Aging and cellular senescence also exhibit characteristic shifts in EV abundance and miRNA cargo. EVs released from aged muscle and senescent cells are enriched in miRNAs such as miR-34a and miR-31, which propagate senescence signals, correlate with inflammatory markers, and are inversely associated with SIRT1 activity, thereby providing an EV-miRNA signature that likely reflects chronic NOX-associated redox imbalance in older individuals. Similar patterns have been described for miR-146a, miR-210, and related stress-responsive miRNAs in age-associated cardiovascular and neurodegenerative conditions [514, 515]. Taken together, these mechanistic, translational, and population studies support a practical diagnostic framework that is fully compatible with raft-enriched exosome profiling. This includes coupling standardized exosome isolation and raft-aware fractionation (e.g., density or detergent-resistant membrane-enriched EV subfractions) with (i) orthogonal physical/particle characterization using nanoparticle tracking analysis (NTA) and flow cytometry; (ii) protein panels combining tetraspanins, raft scaffolds, and NOX subunits and regulators where appropriate; and (iii) targeted EV-miRNA panels (e.g., miR-21, miR-34a, miR-146a, and miR-210) plus a minimal lipidomic raft fingerprint to generate composite NOX-relevant scores. Collectively, such an integrated EV-based platform yields sensitive, exposure- and disease-relevant readouts that can be used for patient stratification before NOX-targeted interventions and as pharmacodynamic markers of inhibitor response in aging, cancer, and pollutant-exposure settings.

## Case studies from clinical trials with patient-specific NOX targeting

Building on the preclinical evidence summarized previously, clinical and late-stage translational studies support isoform-selective NOX1/4 inhibition as a viable therapeutic strategy in fibrotic, cardiovascular, and oncologic settings. Setanaxib, a first-in-class orally active dual NOX1/NOX4 inhibitor, has shown broad antifibrotic, anti-inflammatory, and antioxidant activity across multiple organ systems, providing a leading example of patient-specific NOX targeted therapy [12]. In fibrosis, a comprehensive preclinical and translational program from Thannickal and colleagues demonstrated that NOX1/4 inhibition reduces ROS-driven fibrosis, inflammation, and tissue remodeling in hepatic, renal, and pulmonary models, confirming the rationale for targeting NOX isoforms in fibrotic disease [12]. A landmark phase II randomized, placebo-controlled trial by Invernizzi et al. evaluated setanaxib in patients with primary biliary cholangitis (PBC), a chronic autoimmune cholestatic liver disease characterized by oxidative and inflammatory injury. This trial demonstrated that setanaxib significantly reduced the expression of markers of cholestasis (γ-glutamyl transferase and alkaline phosphatase) and liver stiffness, with the most pronounced benefit in patients who exhibited high baseline oxidative stress and NOX4 expression, underscoring the importance of biomarker-guided patient selection [426]. In diabetic nephropathy, Gray and colleagues demonstrated that pharmacologic inhibition of NOX1/4 using setanaxib ameliorated renal inflammation, decreased TGF-β and collagen-IV expression, and improved renal function in murine models, forming the mechanistic basis for subsequent human trials [428]. Their findings established NOX-dependent ROS as upstream triggers of glomerular fibrosis. Early experimental work by Aoyama et al. in chronic liver injury similarly demonstrated that dual NOX1/NOX4 blockade suppresses stellate-cell activation, matrix deposition, and TGF-β signaling, which are hallmarks of hepatic fibrogenesis, further defining NOX1/4 as actionable antifibrotic targets [516]. In models of doxorubicin-induced cardiotoxicity, setanaxib treatment attenuated MAPK (ERK1/2, JNK, and p38) activation, suppressed cardiomyocyte apoptosis, and preserved mitochondrial function [411]. These findings suggest that NOX1- and NOX4-derived ROS are critical mediators of chemotherapeutic oxidative injury and that their inhibition can protect the myocardium from cumulative oxidative stress [411]. Consistent with this, setanaxib has been shown in cancer-associated fibroblast (CAF)-rich tumor models to convert myofibroblastic cancer-associated fibroblasts to a less immunosuppressive phenotype, increase intratumoral CD8⁺ T-cell infiltration, and restore anti-PD-1 responsiveness, providing an experimental foundation for ongoing combination trials in oncology [141].

Insights into patient-specific targeting:

These case studies collectively highlight several guiding principles for personalized NOX-targeted therapy:

1. Biomarker stratification matters: In patients with primary biliary cholangitis (PBC), a phase 2 randomized controlled trial of setanaxib demonstrated that patients with advanced baseline liver fibrosis (liver stiffness ≥9.6 kPa) experienced the greatest reduction in liver stiffness following 24 weeks of treatment with 400 mg BID of setanaxib, suggesting that patients with more advanced hepatic disease and presumably higher NOX-driven oxidative stress may benefit most, although this requires confirmation in larger trials [426, 517]. Pretreatment stratification using NOX isoform mRNA/protein levels, systemic redox indicators (e.g., H₂O₂ and oxidized/reduced glutathione), and EV-derived redox cargo is therefore a rational strategy to increase therapeutic predictability.
2. Isoform specificity and tissue context: NOX isoforms differ in tissue distribution and activation context. NOX1 is predominant in epithelial and vascular smooth muscle cells, whereas NOX4 is enriched in fibroblasts, hepatocytes, and renal cells. Hence, selecting isoform-appropriate inhibitors on the basis of tissue pathology optimizes efficacy and minimizes off-target effects. Dual NOX1/4 inhibition with setanaxib and related inhibitors has demonstrated efficacy in liver and kidney fibrosis models [12, 518, 519]. In contrast, NOX2-selective agents such as GSK2795039 or TG15-132 appear better suited to conditions where microglial or cardiomyocyte NOX2 is dominant, including neuroinflammation and anthracycline cardiotoxicity [306, 446].
3. Pharmacodynamic integration with EV/raft biomarkers: In fibrosis and cardiotoxicity models, NOX1/4 inhibition consistently reduces ROS generation and fibrosis markers, leading to measurable improvements in tissue function [411, 519, 520]. These findings support the use of EV-based redox markers may serve as useful pharmacodynamic indicators of treatment efficacy. Incorporating exosome-based readouts, tetraspanins plus raft scaffolds (flotillin and caveolin), NOX subunits and EV-miRNAs such as miR-21, miR-34a and miR-200c would provide minimally invasive pharmacodynamic markers of NOX inhibition and help refine dose, timing and patient selection.

Safety and tolerability: Early-phase clinical and pharmacological studies have indicated that the safety and pharmacokinetic profile of setanaxib are favorable for chronic oral dosing, supporting its continued development for fibrotic, oncologic, and cardiovascular indications [411, 426].

Implications for translational research**:** Investigators studying exosome biology, lipid rafts, and pollutant-mediated NOX activation can bridge these challenges through translation. Profiling patients or experimental models based on NOX isoform expression, exosomal raft marker enrichment, and EV-miRNA patterns before and after NOX inhibition can reveal not only therapeutic responsiveness but also mechanistic insight into redox-dependent intercellular communication. This approach directly complements ongoing research into exosome biogenesis, p38/MAPK modulation, and environmental pollutant toxicity, linking molecular redox mechanisms with precision diagnostics and patient-specific interventions.

## Future perspectives

Despite substantial progress in defining NADPH oxidases as central regulators of redox biology, immunity, and pollutant-induced injury, several knowledge gaps remain that now shape the next phase of NOX research. A top priority is the development of isoform-specific and compartment-specific inhibitors, since current approaches often lack selectivity and risk suppressing physiologically beneficial ROS. Recent structural advances enabled by cryo-EM and AlphaFold should be leveraged to design small molecules that distinguish between NOX1, NOX2, and NOX4 on the basis of conformational states or PTM-dependent surfaces. Equally important is the exploration of combinatorial strategies that jointly target NOX activity, epigenetic regulators (EZH2-enhancer of Zeste homolog 2/PRC2- polycomb repressive complex 2), and inflammatory signaling pathways, including Hippo, NF-κB, and STAT3, particularly in diseases where pollutants or metabolic stress converge on shared redox nodes.

**Fig. 6:**
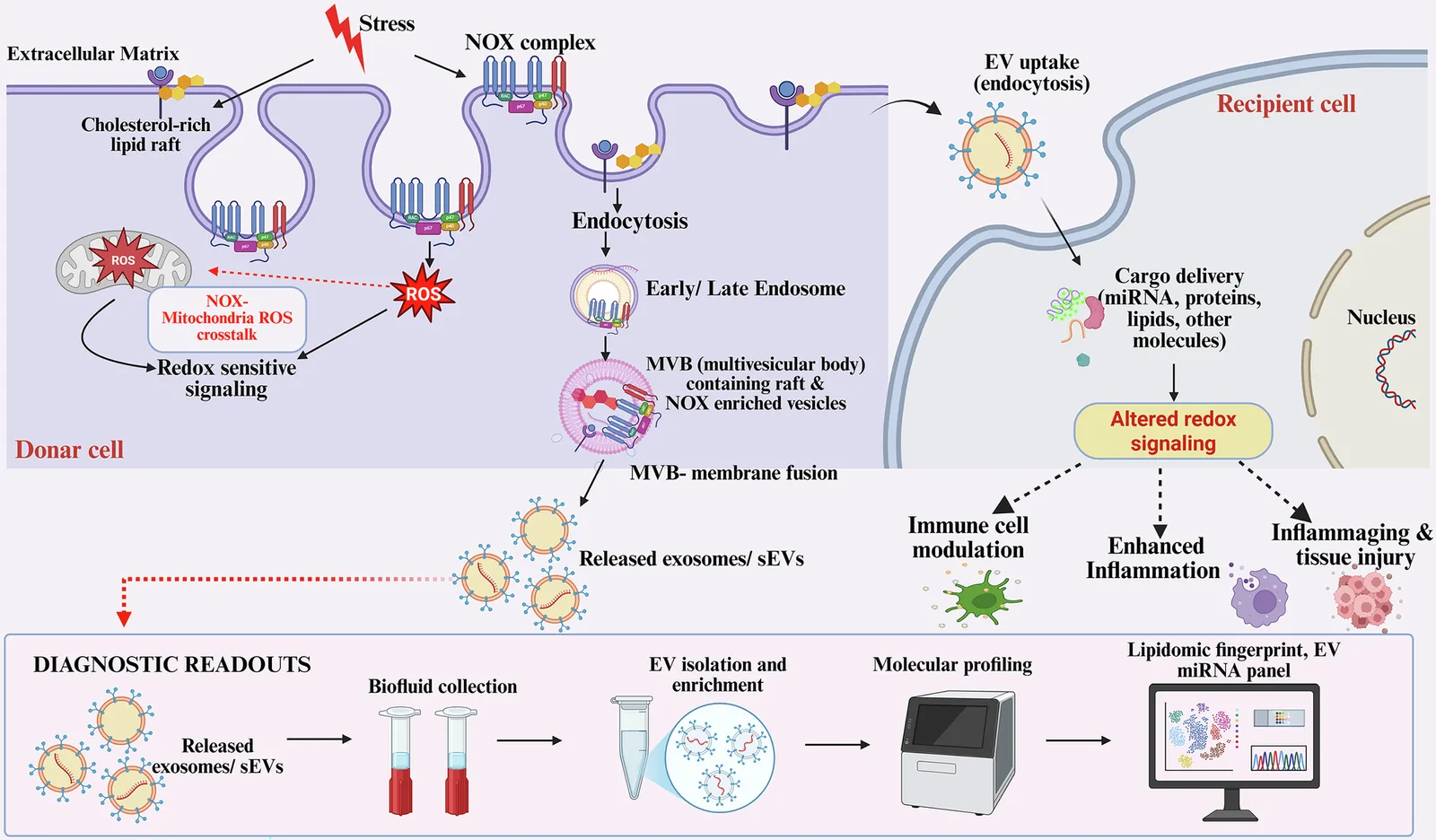
Lipid-raft-enriched exosomes serve as NOX-linked carriers of redox information and diagnostic signatures. Exosomes possess distinct molecular fingerprints that reflect the cellular origin and redox state of donor cells. A defining feature of exosome membranes is their enrichment in cholesterol- and sphingolipid-rich lipid-raft microdomains that function as signaling hubs and selective cargo-sorting platforms during multivesicular body (MVB) maturation. Raft-resident proteins (e.g., flotillin-1/2 and caveolin-1), saturated lipids (e.g., cholesterol, sphingomyelin, and ceramides), NOX-regulated proteins, and stress-responsive microRNAs are preferentially incorporated into these vesicles in a redox-sensitive manner. Because exosome biogenesis and cargo loading are regulated by NOX-derived reactive oxygen species (ROS) through ESCRT components, ceramide pathways, and Rab GTPases, raft-enriched exosomes provide a stable and protected readout of cellular redox imbalance. Once released, these vesicles transmit inflammatory and metabolic signals to recipient cells and can be recovered from biofluids, positioning lipid-raft-enriched exosomes as robust, minimally invasive biomarkers for monitoring NOX-driven pathophysiology and therapeutic responses

Another area requiring systematic investigation is the role of noncoding RNAs, chromatin architecture, and long-range enhancer-promoter interactions in regulating NOX isoforms under stress. Emerging evidence suggests that miRNAs, lncRNAs, and circRNAs can fine-tune NOX activity, but comprehensive regulatory maps remain incomplete. Similarly, exosome-based diagnostics represent a promising frontier, in which lipid-raft-enriched EVs carrying NOX-linked signatures may serve as minimally invasive biomarkers of pollutant exposure, systemic inflammation, and early organ injury. Standardization of EV isolation, lipidomics, and quantitative raft profiling will be essential for clinical translation according to MISEV2023 and related guidelines. Lipid-raft-enriched extracellular vesicles offer a scalable and minimally invasive platform for monitoring NOX-driven redox pathology across tissues, as illustrated in Fig. 6.

In the environmental health domain, future work should focus on longitudinal models that track pollutant-NOX-disease trajectories, incorporating real-world exposures and multipollutant mixtures. The integration of microbiome shifts, immunometabolism, and aging with NOX biology is another pressing need, as these variables profoundly alter host resilience and inflammatory tone. The concept of chrono-NOX regulation, in which circadian rhythms modulate redox signaling and pollutant susceptibility, remains largely unexplored but could have therapeutic implications for the timing of NOX inhibitor treatments. Together, these directions position the field to move beyond descriptive toxicology toward predictive, mechanism-based interventions.

## Conclusions

This review integrates recent advances in redox biology, immunology, structural biochemistry, and toxicology to provide an updated, systems-level view of NADPH oxidases. Building on our earlier work, the scope is expanded by incorporating new high-resolution structural studies, including cryo-EM and AlphaFold-based models, post-2022 transcriptomic and mechanistic studies, and emerging evidence linking NOX activity with environmental pollution, immune plasticity, and chromatin remodeling. By organizing these findings across classical immune functions and newly recognized pathways, this review offers a broader framework that reflects the complexity of redox signaling in modern cell biology.

A central theme is that diverse classes of pollutants converge on NOX1, NOX2, NOX4, and, in selected contexts, NOX5 as key redox sensors and amplifiers in lung, vascular, hepatic, renal, and neural tissues. NOX isoforms act as stress-responsive redox enzymes that are coupled with real-world exposure to oxidative stress, mitochondrial dysfunction, barrier disruption, and downstream inflammatory and fibrotic circuits and are often coordinated through extracellular vesicles and noncoding RNAs. This review further advances the discussion by placing NOX enzymes within the contexts of spatial redox signaling, exosome biology, and epigenetic control, which together shape how tissues respond to both physiological cues and toxic insults.

Taken together, the evidence synthesized in this review provides a comprehensive and updated perspective of NADPH oxidases, highlighting how their functions extend across immunity, metabolism, environmental toxicology, and disease progression. By defining key mechanistic gaps and translational opportunities, this article provides a foundation for future work on isoform-specific targeting, pollutant-responsive redox pathways, and the development of NOX-based diagnostics and therapeutics.

## Supplementary information


Supplementary Table 1


## References

[1] Bedard K, Krause K-H. The NOX family of ROS-generating NADPH oxidases: physiology and pathophysiology. Physiol Rev. 2007;87:245–313. 17237347 10.1152/physrev.00044.2005

[2] Babior BM, Kipnes RS, Curnutte JT. Biological defense mechanisms. The production by leukocytes of superoxide, a potential bactericidal agent. J Clin Investig. 1973;52:741–4. 4346473 10.1172/JCI107236 PMC302313

[3] Panday A, Sahoo MK, Osorio D, Batra S. NADPH oxidases: an overview from structure to innate immunity-associated pathologies. Cell Mol Immunol. 2015;12:5–23. 25263488 10.1038/cmi.2014.89 PMC4654378

[4] Begum R, Thota S, Abdulkadir A, Kaur G, Bagam P, Batra S. NADPH oxidase family proteins: signaling dynamics to disease management. Cell Mol Immunol. 2022;19:660–86. 35585127 10.1038/s41423-022-00858-1 PMC9151672

[5] Miyano K, Okamoto S, Yamauchi A, Kawai C, Kajikawa M, Kiyohara T, et al. The NADPH oxidase NOX4 promotes the directed migration of endothelial cells by stabilizing vascular endothelial growth factor receptor 2 protein. J Biol Chem. 2020;295:11877–90. 32616654 10.1074/jbc.RA120.014723 PMC7450136

[6] Jiang T, Zhang Y, Guo Z, Ren H, Hu W, Yao Q, et al. Mechanical stress induced NOX2 promotes endothelial dysfunction in ventilator-induced lung injury: potential treatment with quercetin. Adv Sci. 2025;12:2502639. 10.1002/advs.202502639 PMC12302586 40391857

[7] Brown DI, Griendling KK. Regulation of signal transduction by reactive oxygen species in the cardiovascular system. Circ Res. 2015;116:531–49. 25634975 10.1161/CIRCRESAHA.116.303584 PMC4392388

[8] Frey RS, Ushio–Fukai M, Malik AB. NADPH oxidase-dependent signaling in endothelial cells: role in physiology and pathophysiology. Antioxid Redox Signal. 2009;11:791–810. 18783313 10.1089/ars.2008.2220 PMC2790033

[9] Liu R, Song K, Wu J-X, Geng X-P, Zheng L, Gao X, et al. Structure of human phagocyte NADPH oxidase in the resting state. eLife. 2022;11:e83743. 36413210 10.7554/eLife.83743 PMC9711523

[10] Liang S, Liu A, Liu Y, Wang F, Zhou Y, Long Y, et al. Structural basis for EROS binding to human phagocyte NADPH oxidase NOX2. Proc Natl Acad Sci USA. 2024;121:e2320388121. 38805284 10.1073/pnas.2320388121 PMC11161758

[11] Noreng S, Ota N, Sun Y, Ho H, Johnson M, Arthur CP, et al. Structure of the core human NADPH oxidase NOX2. Nat Commun. 2022;13:6079. 36241643 10.1038/s41467-022-33711-0 PMC9568551

[12] Thannickal VJ, Jandeleit-Dahm K, Szyndralewiez C, Török NJ. Pre-clinical evidence of a dual NADPH oxidase 1/4 inhibitor (setanaxib) in liver, kidney and lung fibrosis. J Cell Mol Med. 2023;27:471–81. 36658776 10.1111/jcmm.17649 PMC9930438

[13] St. Clair DK, Jordan JA, Wan XS, Gairola CG. Protective role of manganese superoxide dismutase against cigarette smoke-induced cytotoxicity. J Toxicol Environ Health Part A Curr Issues. 1994;43:239–49. 10.1080/15287399409531918 7932852

[14] Drummond GR, Selemidis S, Griendling KK, Sobey CG. Combating oxidative stress in vascular disease: NADPH oxidases as therapeutic targets. Nat Rev Drug Discov. 2011;10:453–71. 21629295 10.1038/nrd3403 PMC3361719

[15] Mir S, Ormsbee Golden BD, Griess BJ, Vengoji R, Tom E, Kosmacek EA, et al. Upregulation of Nox4 induces a pro-survival Nrf2 response in cancer-associated fibroblasts that promotes tumorigenesis and metastasis, in part via Birc5 induction. Breast Cancer Res. 2022;24:48. 35836253 10.1186/s13058-022-01548-6 PMC9281082

[16] Hsu N-Y, Nayar S, Gettler K, Talware S, Giri M, Alter I, et al. NOX1 is essential for TNFα-induced intestinal epithelial ROS secretion and inhibits M cell signatures. Gut. 2023;72:654–62. 36191961 10.1136/gutjnl-2021-326305 PMC9998338

[17] Bücher T. Otto Warburg: a personal recollection. In: Biological oxidations. Berlin, Heildelberg: Springer; 1983;34:1–29.

[18] Thomas DC. The phagocyte respiratory burst: Historical perspectives and recent advances. Immunol Lett. 2017;192:88–96. 28864335 10.1016/j.imlet.2017.08.016

[19] Karnovsky ML. Metabolic basis of phagocytic activity. Physiol Rev. 1962;42:143–68. 14454025 10.1152/physrev.1962.42.1.143

[20] Dinauer MC. Chronic granulomatous disease and other disorders of phagocyte function. ASH Educ Program Book. 2005;2005:89–95. 10.1182/asheducation-2005.1.89 16304364

[21] Qiao X, Yin J, Zheng Z, Li L, Feng X. Endothelial cell dynamics in sepsis-induced acute lung injury and acute respiratory distress syndrome: pathogenesis and therapeutic implications. Cell Commun Signal. 2024;22:241. 38664775 10.1186/s12964-024-01620-y PMC11046830

[22] Pendyala S, Gorshkova IA, Usatyuk PV, He D, Pennathur A, Lambeth JD, et al. Role of Nox4 and Nox2 in hyperoxia-induced reactive oxygen species generation and migration of human lung endothelial cells. Antioxid Redox Signal. 2009;11:747–64. 18783311 10.1089/ars.2008.2203 PMC2850303

[23] Carnesecchi S, Deffert C, Pagano A, Garrido-Urbani S, Métrailler-Ruchonnet I, Schäppi M, et al. NADPH oxidase-1 plays a crucial role in hyperoxia-induced acute lung injury in mice. Am J Respir Crit Care Med. 2009;180:972–81. 19661248 10.1164/rccm.200902-0296OC PMC2778156

[24] Wang D, Li J, Luo G, Zhou J, Wang N, Wang S, et al. Nox4 as a novel therapeutic target for diabetic vascular complications. Redox Biol. 2023;64:102781. 37321060 10.1016/j.redox.2023.102781 PMC10363438

[25] Siedlar AM, Seredenina T, Faivre A, Cambet Y, Stasia M-J, André-Lévigne D, et al. NADPH oxidase 4 is dispensable for skin myofibroblast differentiation and wound healing. Redox Biol. 2023;60:102609. 36708644 10.1016/j.redox.2023.102609 PMC9950659

[26] Hunt M, Torres M, Bachar-Wikstrom E, Wikstrom JD. Cellular and molecular roles of reactive oxygen species in wound healing. Commun Biol. 2024;7:1534. 39562800 10.1038/s42003-024-07219-w PMC11577046

[27] Sun J, Gong Z, Zheng L, Ding Z, Wang L, Tang Y, et al. NOX4 serves as a pancancer prognostic biomarker and therapeutic target in tumorigenesis. Sci Rep. 2025;15:24612. 40634441 10.1038/s41598-025-09690-9 PMC12241599

[28] Kawahara T, Quinn MT, Lambeth JD. Molecular evolution of the reactive oxygen-generating NADPH oxidase (Nox/Duox) family of enzymes. BMC Evol Biol. 2007;7:109. 17612411 10.1186/1471-2148-7-109 PMC1940245

[29] Gandara ACP, Torres A, Bahia AC, Oliveira PL, Schama R. Evolutionary origin and function of NOX4-art, an arthropod specific NADPH oxidase. BMC Evol Biol. 2017;17:92. 28356077 10.1186/s12862-017-0940-0 PMC5372347

[30] Vermot A, Petit-Härtlein I, Smith SM, Fieschi F. NADPH oxidases (NOX): an overview from discovery, molecular mechanisms to physiology and pathology. Antioxidants. 2021;10:890. 34205998 10.3390/antiox10060890 PMC8228183

[31] Moll F, Walter M, Rezende F, Helfinger V, Vasconez E, De Oliveira T, et al. NoxO1 controls proliferation of colon epithelial cells. Front Immunol. 2018;9:973. 29867954 10.3389/fimmu.2018.00973 PMC5951971

[32] Liu X, Shi Y, Liu R, Song K, Chen L. Structure of human phagocyte NADPH oxidase in the activated state. Nature. 2024;627:189–95. 38355798 10.1038/s41586-024-07056-1

[33] Liu J, Pintard C, Thieblemont N, Dang PM-C, El-Benna J. Deciphering opsonized zymosan–induced phosphorylation of p47phox and NADPH oxidase activation in human neutrophils. Blood. 2025;146:1601–11. 40561248 10.1182/blood.2024027018

[34] Hebchen DM, Schader T, Spaeth M, Müller N, Graumann J, Schröder K. NoxO1 regulates EGFR signaling by its interaction with Erbin. Redox Biol. 2024;77:103396. 39426288 10.1016/j.redox.2024.103396 PMC11536020

[35] Lagal DJ, Bárcena JA, Requejo-Aguilar R, Padilla CA, Leto TL. NOX1 and PRDX6 synergistically support migration and invasiveness of hepatocellular carcinoma cells through enhanced NADPH oxidase activity. Adv Redox Res. 2023;9:100080. 37900981 10.1016/j.arres.2023.100080 PMC10611439

[36] Herb M. NADPH oxidase 3: beyond the inner ear. Antioxidants. 2024;13:219. 38397817 10.3390/antiox13020219 PMC10886416

[37] Lyle AN, Deshpande NN, Taniyama Y, Seidel-Rogol B, Pounkova L, Du P, et al. Poldip2, a novel regulator of Nox4 and cytoskeletal integrity in vascular smooth muscle cells. Circ Res. 2009;105:249–59. 19574552 10.1161/CIRCRESAHA.109.193722 PMC2744198

[38] Ogboo BC, Grabovyy UV, Maini A, Scouten S, van der Vliet A, Mattevi A, et al. Architecture of the NADPH oxidase family of enzymes. Redox Biol. 2022;52:102298. 35334249 10.1016/j.redox.2022.102298 PMC8956913

[39] Cui C. Structural basis of human NOX5 activation. Biophys J. 2024;123:50a. 10.1038/s41467-024-48467-y PMC11088703 38734761

[40] Wu J-X, Liu R, Song K, Chen L. Structures of human dual oxidase 1 complex in low-calcium and high-calcium states. Nat Commun. 2021;12:155. 33420071 10.1038/s41467-020-20466-9 PMC7794343

[41] Sun J. Structures of mouse DUOX1–DUOXA1 provide mechanistic insights into enzyme activation and regulation. Nat Struct Mol Biol. 2020;27:1086–93. 32929281 10.1038/s41594-020-0501-x PMC7644671

[42] Kohda A, Kamakura S, Hayase J, Sumimoto H. The NADPH oxidases DUOX1 and DUOX2 are sorted to the apical plasma membrane in epithelial cells via their respective maturation factors DUOXA1 and DUOXA2. Genes Cells. 2024;29:921–30. 39126279 10.1111/gtc.13153 PMC11555622

[43] Pandey D, Chen F, Patel A, Wang C-Y, Dimitropoulou C, Patel VS, et al. SUMO1 negatively regulates reactive oxygen species production from NADPH oxidases. Arterioscler Thromb Vasc Biol. 2011;31:1634–42. 21527745 10.1161/ATVBAHA.111.226621 PMC3464053

[44] Fraga CG, Oteiza PI, Hid EJ, Galleano M. (Poly)phenols and the regulation of NADPH oxidases. Redox Biol. 2023;67:102927. 37857000 10.1016/j.redox.2023.102927 PMC10587761

[45] Bucheli OT, Rodrigues D, Ulbricht C, Hauser AE, Eyer K. Dynamic activation of NADPH oxidases in immune responses modulates differentiation, function, and lifespan of plasma cells. Eur J Immunol. 2025;55:e202350975. 39931760 10.1002/eji.202350975 PMC11811814

[46] Suh Y-A, Arnold RS, Lassegue B, Shi J, Xu X, Sorescu D, et al. Cell transformation by the superoxide-generating oxidase Mox1. Nature. 1999;401:79–82. 10485709 10.1038/43459

[47] Lassègue B, Sorescu D, Szöcs K, Yin Q, Akers M, Zhang Y, et al. Novel gp91 phox homologs in vascular smooth muscle cells: nox1 mediates angiotensin II–induced superoxide formation and redox-sensitive signaling pathways. Circ Res. 2001;88:888–94. 11348997 10.1161/hh0901.090299

[48] Hou Y, Qiu W, Ling Y, Qi X, Liu J, Yang H, et al. The role of tumor-associated macrophages in glioma cohort: through both traditional RNA sequencing and single cell RNA sequencing. Front Oncol. 2023;13:1249448. 37781198 10.3389/fonc.2023.1249448 PMC10539593

[49] Han Y, Wang Y, Dong X, Sun D, Liu Z, Yue J, et al. TISCH2: expanded datasets and new tools for single-cell transcriptome analyses of the tumor microenvironment. Nucleic Acids Res. 2023;51:D1425–31. 36321662 10.1093/nar/gkac959 PMC9825603

[50] Kim JY, Kang W, Yang S, Park SH, Ha SY, Paik Y-H. NADPH oxidase 4 deficiency promotes hepatocellular carcinoma arising from hepatic fibrosis by inducing M2-macrophages in the tumor microenvironment. Sci Rep. 2024;14:22358. 39333166 10.1038/s41598-024-72721-4 PMC11437090

[51] Zhang J, Li H, Wu Q, Chen Y, Deng Y, Yang Z, et al. Tumoral NOX4 recruits M2 tumor-associated macrophages via ROS/PI3K signaling-dependent various cytokine production to promote NSCLC growth. Redox Biol. 2019;22:101116. 30769285 10.1016/j.redox.2019.101116 PMC6374999

[52] Shanmugasundaram K, Nayak BK, Friedrichs WE, Kaushik D, Rodriguez R, Block K. NOX4 functions as a mitochondrial energetic sensor coupling cancer metabolic reprogramming to drug resistance. Nat Commun. 2017;8:997. 29051480 10.1038/s41467-017-01106-1 PMC5648812

[53] Whitmore LC, Hilkin BM, Goss KL, Wahle EM, Colaizy TT, Boggiatto PM, et al. NOX2 protects against prolonged inflammation, lung injury, and mortality following systemic insults. J Innate Immun. 2013;5:565–80. 23635512 10.1159/000347212 PMC6741530

[54] Morimoto H, Kanatsu-Shinohara M, Shinohara T. ROS-generating oxidase Nox3 regulates the self-renewal of mouse spermatogonial stem cells. Biol Reprod. 2015;92:147 1–10. 25947060 10.1095/biolreprod.114.127647

[55] Erlich JR, To EE, Luong R, Liong F, Liong S, Oseghale O, et al. Glycolysis and the pentose phosphate pathway promote LPS-induced Nox2 oxidase-and IFN-β-dependent inflammation in macrophages. Antioxidants. 2022;11:1488. 36009206 10.3390/antiox11081488 PMC9405479

[56] Rathi D, Rossi C, Pospíšil P, Ramalingam Manoharan R, Talarico L, Magnani A, et al. NOX2 and NOX4 expression in monocytes and macrophages-extracellular vesicles in signaling and therapeutics. Front Cell Dev Biol. 2024;12:1342227. 38690564 10.3389/fcell.2024.1342227 PMC11058225

[57] Park MN, Kim M, Lee S, Kang S, Ahn C-H, Tallei TE, et al. Targeting redox signaling through exosomal MicroRNA: insights into tumor microenvironment and precision oncology. Antioxidants. 2025;14:501. 40427384 10.3390/antiox14050501 PMC12108341

[58] Gu L, Feng C, Li M, Hong Z, Di W, Qiu L. Exosomal NOX1 promotes tumor-associated macrophage M2 polarization-mediated cancer progression by stimulating ROS production in cervical cancer: a preliminary study. Eur J Med Res. 2023;28:323. 37679792 10.1186/s40001-023-01246-9 PMC10483767

[59] Graham DB, Becker CE, Doan A, Goel G, Villablanca EJ, Knights D, et al. Functional genomics identifies negative regulatory nodes controlling phagocyte oxidative burst. Nat Commun. 2015;6:7838. 26194095 10.1038/ncomms8838 PMC4518307

[60] Miyano K, Okamoto S, Kajikawa M, Kiyohara T, Kawai C, Yamauchi A, et al. Regulation of Derlin-1-mediated degradation of NADPH oxidase partner p22phox by thiol modification. Redox Biol. 2022;56:102479. 36122532 10.1016/j.redox.2022.102479 PMC9486109

[61] Haq S, Sarodaya N, Karapurkar JK, Suresh B, Jo JK, Singh V, et al. CYLD destabilizes NoxO1 protein by promoting ubiquitination and regulates prostate cancer progression. Cancer Lett. 2022;525:146–57. 34742871 10.1016/j.canlet.2021.10.032

[62] Cipriano A, Viviano M, Feoli A, Milite C, Sarno G, Castellano S, et al. NADPH oxidases: from molecular mechanisms to current inhibitors. J Med Chem. 2023;66:11632–55. 37650225 10.1021/acs.jmedchem.3c00770 PMC10510401

[63] Fischer H. Mechanisms and function of DUOX in epithelia of the lung. Antioxid Redox Signal. 2009;11:2453–65. 19358684 10.1089/ars.2009.2558 PMC2823369

[64] Grasberger H, Refetoff S. Genetic causes of congenital hypothyroidism due to dyshormonogenesis. Curr Opin Pediatr. 2011;23:421–8. 21543982 10.1097/MOP.0b013e32834726a4 PMC3263319

[65] Groemping Y, Lapouge K, Smerdon SJ, Rittinger K. Molecular basis of phosphorylation-induced activation of the NADPH oxidase. Cell. 2003;113:343–55. 12732142 10.1016/s0092-8674(03)00314-3

[66] Dang PM-C, Stensballe A, Boussetta T, Raad H, Dewas C, Kroviarski Y, et al. A specific p47 phox-serine phosphorylated by convergent MAPKs mediates neutrophil NADPH oxidase priming at inflammatory sites. J Clin Investig. 2006;116:2033–43. 16778989 10.1172/JCI27544 PMC1479423

[67] Tlili A, Pintard C, Hurtado-Nedelec M, Liu D, Marzaioli V, Thieblemont N, et al. ROCK2 interacts with p22phox to phosphorylate p47phox and to control NADPH oxidase activation in human monocytes. Proc Natl Acad Sci USA. 2023;120:e2209184120. 36626553 10.1073/pnas.2209184120 PMC9934299

[68] Yu W, Zhuang S, Zhan M, Chen Y, Zhang J, Chen L, et al. Deubiquitinating enzyme USP19 regulates ferroptosis and mitochondrial damage in SH-SY5Y cells by targeting the NOX4 protein. J Alzheimers Dis. 2024;100:799–808. 38943386 10.3233/JAD-231193

[69] Hoyal CR, Gutierrez A, Young BM, Catz SD, Lin J-H, Tsichlis PN, et al. Modulation of p47 PHOX activity by site-specific phosphorylation: Akt-dependent activation of the NADPH oxidase. Proc Natl Acad Sci USA. 2003;100:5130–5. 12704229 10.1073/pnas.1031526100 PMC154310

[70] Fontayne A, Dang PM-C, Gougerot-Pocidalo M-A, El Benna J. Phosphorylation of p47phox sites by PKC α, βΙΙ, δ, and ζ: effect on binding to p22phox and on NADPH oxidase activation. Biochemistry. 2002;41:7743–50. 12056906 10.1021/bi011953s

[71] Kil IS, Lee JH, Yoon SH, Bae YS, Kim S, Shin SW, et al. S-Nitrosylation of p47phox enhances phosphorylation by casein kinase 2. Redox Rep. 2015;20:228–33. 26018922 10.1179/1351000215Y.0000000014 PMC6837347

[72] Evans R, O’Neill M, Pritzel A, Antropova N, Senior A, Green T, et al. Protein complex prediction with AlphaFold-Multimer. biorxiv. 2021;4:2021–10.

[73] Yu D, Chojnowski G, Rosenthal M, Kosinski J. AlphaPulldown—a Python package for protein–protein interaction screens using AlphaFold-Multimer. Bioinformatics. 2023;39:btac749. 36413069 10.1093/bioinformatics/btac749 PMC9805587

[74] Omidi A, Møller MH, Malhis N, Bui JM, Gsponer J. AlphaFold-Multimer accurately captures interactions and dynamics of intrinsically disordered protein regions. Proc Natl Acad Sci USA. 2024;121:e2406407121. 39446390 10.1073/pnas.2406407121 PMC11536093

[75] Aimeur S, Fas BA, Serfaty X, Santuz H, Sacquin-Mora S, Bizouarn T, et al. Structural profiles of the full phagocyte NADPH oxidase unveiled by combining computational biology and experimental knowledge. J Biol Chem. 2024;300:107943. 39481598 10.1016/j.jbc.2024.107943 PMC11647612

[76] Paclet M-H, Laurans S, Dupré-Crochet S. Regulation of neutrophil NADPH oxidase, NOX2: a crucial effector in neutrophil phenotype and function. Front Cell Dev Biol. 2022;10:945749. 35912108 10.3389/fcell.2022.945749 PMC9329797

[77] Joly J, Hudik E, Lecart S, Roos D, Verkuijlen P, Wrona D, et al. Membrane dynamics and organization of the phagocyte NADPH oxidase in PLB-985 cells. Front Cell Dev Biol. 2020;8:608600. 33365312 10.3389/fcell.2020.608600 PMC7751761

[78] Casbon A-J, Allen L-AH, Dunn KW, Dinauer MC. Macrophage NADPH oxidase flavocytochrome B localizes to the plasma membrane and Rab11-positive recycling endosomes. J Immunol. 2009;182:2325–39. 19201887 10.4049/jimmunol.0803476 PMC2666390

[79] Dingjan I, Linders PT, van den Bekerom L, Baranov MV, Halder P, Ter Beest M, et al. Oxidized phagosomal NOX2 complex is replenished from lysosomes. J Cell Sci. 2017;130:1285–98. 28202687 10.1242/jcs.196931 PMC5399780

[80] Hebchen DM, Spaeth M, Müller N, Schröder K. NoxO1 determines the level of ROS formation by the Nox1-centered NADPH oxidase. Antioxidants. 2024;13:1113. 39334772 10.3390/antiox13091113 PMC11428687

[81] Wang Y, Wang W, Zhou S, Wang Y, Cudjoe O, Cha Y, et al. Poldip2 knockdown protects against lipopolysaccharide-induced acute lung injury via Nox4/Nrf2/NF-κB signaling pathway. Front Pharmacol. 2022;13:958916. 36120334 10.3389/fphar.2022.958916 PMC9471427

[82] Trinh VH, Nguyen Huu T, Sah DK, Choi JM, Yoon HJ, Park SC, et al. Redox regulation of PTEN by reactive oxygen species: its role in physiological processes. Antioxidants. 2024;13:199. 38397797 10.3390/antiox13020199 PMC10886030

[83] Veal EA, Kritsiligkou P. How are hydrogen peroxide messages relayed to affect cell signaling? Curr Opin Chem Biol. 2024;81:102496. 38959751 10.1016/j.cbpa.2024.102496

[84] Sies H, Jones DP. Reactive oxygen species (ROS) as pleiotropic physiological signaling agents. Nat Rev Mol Cell Biol. 2020;21:363–83. 32231263 10.1038/s41580-020-0230-3

[85] Savina A, Jancic C, Hugues S, Guermonprez P, Vargas P, Moura IC, et al. NOX2 controls phagosomal pH to regulate antigen processing during crosspresentation by dendritic cells. Cell. 2006;126:205–18. 16839887 10.1016/j.cell.2006.05.035

[86] Cai H, Griendling KK, Harrison DG. The vascular NAD (P) H oxidases as therapeutic targets in cardiovascular diseases. Trends Pharmacol Sci. 2003;24:471–8. 12967772 10.1016/S0165-6147(03)00233-5

[87] Beretta M, Santos CX, Molenaar C, Hafstad AD, Miller CC, Revazian A, et al. Nox4 regulates InsP3 receptor-dependent Ca2+ release into mitochondria to promote cell survival. EMBO J. 2020;39:e103530. 33001475 10.15252/embj.2019103530 PMC7527947

[88] Frei MS, Mehta S, Zhang J. Next-generation genetically encoded fluorescent biosensors illuminate cell signaling and metabolism. Annu Rev Biophys. 2024;53:275–97. 38346245 10.1146/annurev-biophys-030722-021359 PMC11786609

[89] Gest AM, Sahan AZ, Zhong Y, Lin W, Mehta S, Zhang J. Molecular spies in action: genetically encoded fluorescent biosensors light up cellular signals. Chem Rev. 2024;124:12573–660. 39535501 10.1021/acs.chemrev.4c00293 PMC11613326

[90] Xiong B, Zhang Y, Liu S, Liao S, Zhou Z, He Q, et al. NOX family: regulators of reactive oxygen species balance in tumor cells. FASEB J. 2025;39:e70565. 40266050 10.1096/fj.202500238RRR PMC12017260

[91] Konate MM, Antony S, Doroshow JH. Inhibiting the activity of NADPH oxidase in cancer. Antioxid Redox Signal. 2020;33:435–54. 32008376 10.1089/ars.2020.8046 PMC7370979

[92] Britt EC, Lika J, Giese MA, Schoen TJ, Seim GL, Huang Z, et al. Switching to the cyclic pentose phosphate pathway powers the oxidative burst in activated neutrophils. Nat Metab. 2022;4:389–403. 35347316 10.1038/s42255-022-00550-8 PMC8964420

[93] Zhao J, Li J, Li G, Chen M. The role of mitochondria-associated membranes mediated ROS on NLRP3 inflammasome in cardiovascular diseases. Front Cardiovasc Med. 2022;9:1059576. 36588561 10.3389/fcvm.2022.1059576 PMC9794868

[94] Matuz-Mares D, Vázquez-Meza H, Vilchis-Landeros MM. NOX as a therapeutic target in liver disease. Antioxidants. 2022;11:2038. 36290761 10.3390/antiox11102038 PMC9598239

[95] Hu K, Relton E, Locker N, Phan NT, Ewing AG. Electrochemical measurements reveal reactive oxygen species in stress granules. Angew Chem Int Ed. 2021;60:15302–6. 10.1002/anie.202104308 PMC8456511 33876544

[96] Lambeth JD. NOX enzymes and the biology of reactive oxygen. Nat Rev Immunol. 2004;4:181–9. 15039755 10.1038/nri1312

[97] Lee S-R, Kwon K-S, Kim S-R, Rhee SG. Reversible inactivation of protein-tyrosine phosphatase 1B in A431 cells stimulated with epidermal growth factor. J Biol Chem. 1998;273:15366–72. 9624118 10.1074/jbc.273.25.15366

[98] Meng T-C, Fukada T, Tonks NK. Reversible oxidation and inactivation of protein tyrosine phosphatases in vivo. Mol Cell. 2002;9:387–99. 11864611 10.1016/s1097-2765(02)00445-8

[99] Xiang M, Fan J, Fan J. Association of toll-like receptor signaling and reactive oxygen species: a potential therapeutic target for posttrauma acute lung injury. Mediators Inflamm. 2010;2010:916425. 20706658 10.1155/2010/916425 PMC2913855

[100] Ortiz C, Caja L, Bertran E, Gonzalez-Rodriguez Á, Valverde ÁM, Fabregat I, et al. Protein-tyrosine phosphatase 1B (PTP1B) deficiency confers resistance to transforming growth factor-β (TGF-β)-induced suppressor effects in hepatocytes. J Biol Chem. 2012;287:15263–74. 22427664 10.1074/jbc.M111.303958 PMC3346142

[101] Zhou R, Yazdi AS, Menu P, Tschopp J. A role for mitochondria in NLRP3 inflammasome activation. Nature. 2011;469:221–5. 21124315 10.1038/nature09663

[102] Zhou R, Tardivel A, Thorens B, Choi I, Tschopp J. Thioredoxin-interacting protein links oxidative stress to inflammasome activation. Nat Immunol. 2010;11:136–40. 20023662 10.1038/ni.1831

[103] Muñoz-Planillo R, Kuffa P, Martínez-Colón G, Smith BL, Rajendiran TM, Núñez G. K+ efflux is the common trigger of NLRP3 inflammasome activation by bacterial toxins and particulate matter. Immunity. 2013;38:1142–53. 23809161 10.1016/j.immuni.2013.05.016 PMC3730833

[104] Abais JM, Xia M, Zhang Y, Boini KM, Li P-L. Redox regulation of the NLRP3 inflammasome: ROS as trigger or effector? Antioxid Redox Signal. 2015;22:1111–29. 10.1089/ars.2014.5994 PMC4403231 25330206

[105] Gao P, Li L, Han L, Zhang Y, Quan Z, Ding B, et al. Effects of temperature stress on genome-wide DNA methylation levels in the sea cucumber, Apostichopus japonicus. Iran J Fish Sci. 2024;23:437–56.

[106] Sylvester AL, Zhang DX, Ran S, Zinkevich NS. Inhibiting NADPH oxidases to target vascular and other pathologies: an update on recent experimental and clinical studies. Biomolecules. 2022;12:823. 35740948 10.3390/biom12060823 PMC9221095

[107] Sasaki Y, Dehnad A, Fish S, Sato A, Jiang J, Tian J, et al. NOX4 regulates CCR2 and CCL2 mRNA stability in alcoholic liver disease. Sci Rep. 2017;7:46144. 28383062 10.1038/srep46144 PMC5382722

[108] Lozhkin A, Vendrov AE, Pan H, Wickline SA, Madamanchi NR, Runge MS. NADPH oxidase 4 regulates vascular inflammation in aging and atherosclerosis. J Mol Cell Cardiol. 2017;102:10–21. 27986445 10.1016/j.yjmcc.2016.12.004 PMC5625334

[109] Makhezer N, Ben Khemis M, Liu D, Khichane Y, Marzaioli V, Tlili A, et al. NOX1-derived ROS drive the expression of Lipocalin-2 in colonic epithelial cells in inflammatory conditions. Mucosal Immunol. 2019;12:117–31. 30279516 10.1038/s41385-018-0086-4

[110] Kumar A, Shalmanova L, Hammad A, Christmas SE. Induction of IL-8 (CXCL8) and MCP-1 (CCL2) with oxidative stress and its inhibition with N-acetyl cysteine (NAC) in cell culture model using HK-2 cell. Transpl Immunol. 2016;35:40–6. 26908203 10.1016/j.trim.2016.02.003

[111] Mortimer PM, Mc Intyre SA, Thomas DC. Beyond the extra respiration of phagocytosis: NADPH oxidase 2 in adaptive immunity and inflammation. Front Immunol. 2021;12:733918. 34539670 10.3389/fimmu.2021.733918 PMC8440999

[112] Taylor JP. Hubert MT. The role of NADPH oxidases in infectious and inflammatory diseases. Redox Biol. 2021;48:102159. 34627721 10.1016/j.redox.2021.102159 PMC8487856

[113] Vlahos R, Stambas J, Bozinovski S, Broughton B, Drummond G. Inhibition of Nox2 oxidase activity ameliorates influenza A virus-induced lung inflamamtion. 2011;7:e1001271. 10.1371/journal.ppat.1001271 PMC3033375 21304882

[114] Kondati R, Thakur M, Mutyala D, Dorsey W, Batra S. Persistent environmental toxicants PCP, TCHQ, and HCB drive PANoptosis and RNA granule remodeling in human lung epithelial cells. Environ Toxicol Pharmacol. 2026;121:104862. 41197820 10.1016/j.etap.2025.104862

[115] Chen Y, Guan B, Lu J, Yan X, Huang C, Qiu Y, et al. Gypensapogenin I alleviates PANoptosis, ferroptosis, and oxidative stress in myocardial ischemic–reperfusion injury by targeting the NOX2/AMPK pathway. Front Cell Dev Biol. 2025;13:1623846. 40766780 10.3389/fcell.2025.1623846 PMC12321891

[116] Byun HS, Ju E, Park KA, Sohn K-C, Jung CS, Hong JH, et al. Rubiarbonol B induces RIPK1-dependent necroptosis via NOX1-derived ROS production. Cell Biol Toxicol. 2023;39:1677–96. 36163569 10.1007/s10565-022-09774-6

[117] Ma MW, Wang J, Dhandapani KM, Brann DW. NADPH oxidase 2 regulates NLRP3 inflammasome activation in the brain after traumatic brain injury. Oxid Med Cell Longev. 2017;2017:6057609. 28785377 10.1155/2017/6057609 PMC5529650

[118] Sokolovska A, Becker CE, Ip WE, Rathinam VA, Brudner M, Paquette N, et al. Activation of caspase-1 by the NLRP3 inflammasome regulates the NADPH oxidase NOX2 to control phagosome function. Nat Immunol. 2013;14:543–53. 23644505 10.1038/ni.2595 PMC3708594

[119] Sureshbabu A, Patino E, Ma KC, Laursen K, Finkelsztein EJ, Akchurin O, et al. RIPK3 promotes sepsis-induced acute kidney injury via mitochondrial dysfunction. JCI Insight. 2018;3:e98411. 29875323 10.1172/jci.insight.98411 PMC6124406

[120] Zhang X-J, Li L, Wang A-L, Guo H-X, Zhao H-P, Chi R-F, et al. GSK2795039 prevents RIP1-RIP3-MLKL-mediated cardiomyocyte necroptosis in doxorubicin-induced heart failure through inhibition of NADPH oxidase-derived oxidative stress. Toxicol Appl Pharmacol. 2023;463:116412. 36764612 10.1016/j.taap.2023.116412

[121] Zheng D, Liu J, Piao H, Zhu Z, Wei R, Liu K. ROS-triggered endothelial cell death mechanisms: focus on pyroptosis, parthanatos, and ferroptosis. Front Immunol. 2022;13:1039241. 36389728 10.3389/fimmu.2022.1039241 PMC9663996

[122] Sendtner N, Seitz R, Brandl N, Müller M, Gülow K. Reactive oxygen species across death pathways: gatekeepers of apoptosis, ferroptosis, pyroptosis, paraptosis, and beyond. Int J Mol Sci. 2025;26:10240. 41155529 10.3390/ijms262010240 PMC12564638

[123] Dikalov SI, Dikalova AE, Bikineyeva AT, Schmidt HH, Harrison DG, Griendling KK. Distinct roles of Nox1 and Nox4 in basal and angiotensin II-stimulated superoxide and hydrogen peroxide production. Free Radic Biol Med. 2008;45:1340–51. 18760347 10.1016/j.freeradbiomed.2008.08.013 PMC2630771

[124] Basuroy S, Bhattacharya S, Leffler CW, Parfenova H. Nox4 NADPH oxidase mediates oxidative stress and apoptosis caused by TNF-α in cerebral vascular endothelial cells. Am J Physiol Cell Physiol. 2009;296:C422–32. 19118162 10.1152/ajpcell.00381.2008 PMC2660262

[125] Palomino-Antolín A, Decouty-Pérez C, Farré-Alins V, Narros-Fernández P, Lopez-Rodriguez AB, Álvarez-Rubal M, et al. Redox regulation of microglial inflammatory response: fine control of NLRP3 inflammasome through Nrf2 and NOX4. Antioxidants. 2023;12:1729. 37760032 10.3390/antiox12091729 PMC10525647

[126] Kuroda J, Ago T, Matsushima S, Zhai P, Schneider MD, Sadoshima J. NADPH oxidase 4 (Nox4) is a major source of oxidative stress in the failing heart. Proc Natl Acad Sci USA. 2010;107:15565–70. 20713697 10.1073/pnas.1002178107 PMC2932625

[127] Zhao S, Chen F, Yin Q, Wang D, Han W, Zhang Y. Reactive oxygen species interact with NLRP3 inflammasomes and are involved in the inflammation of sepsis: from mechanism to treatment of progression. Front Physiol. 2020;ume 11:2020. 10.3389/fphys.2020.571810 PMC7723971 33324236

[128] Sun B, Wang X, Ji Z, Wang M, Liao Y-P, Chang CH, et al. NADPH oxidase-dependent NLRP3 inflammasome activation and its important role in lung fibrosis by multiwalled carbon nanotubes. Small. 2015;11:2087–97. 25581126 10.1002/smll.201402859 PMC4420651

[129] Zou W, Lu Q, Zhu X, Pan Y, Xu Q, Wang K. Procyanidin B2 protects TR-iBRB2 cells against hyperglyc emia stress by attenuating oxidative stress and inflammasome activation via regulation of redoxosomes/NF-kB signaling. Curr Mol Med. 2023;23:1095–103. 36263472 10.2174/1566524023666221017120334

[130] LI J, NEUROPATHY GROUP D, JIANG Y, ZHU X, ZHU X. 467-P: Hyperglyceamia-indued oxidative stress inflicted enteric glial cells of mice via the novel pathway redoxosomes/p66shc activation in vivo and in vitro. Diabetes. 2025;74:467-P.

[131] Peng C, Li X, Ao F, Li T, Guo J, Liu J, et al. Mitochondrial ROS driven by NOX4 upregulation promotes hepatocellular carcinoma cell survival after incomplete radiofrequency ablation by inducing of mitophagy via Nrf2/PINK1. J Transl Med. 2023;21:218. 36964576 10.1186/s12967-023-04067-w PMC10039571

[132] Dikalov SI, Nazarewicz RR, Bikineyeva A, Hilenski L, Lassegue B, Griendling KK, et al. Nox2-induced production of mitochondrial superoxide in angiotensin II-mediated endothelial oxidative stress and hypertension. Antioxid Redox Signal. 2014;20:281–94. 24053613 10.1089/ars.2012.4918 PMC3887459

[133] Maalouf RM, Eid AA, Gorin YC, Block K, Escobar GP, Bailey S, et al. Nox4-derived reactive oxygen species mediate cardiomyocyte injury in early type 1 diabetes. Am J Physiol Cell Physiol. 2012;302:C597–604. 22031600 10.1152/ajpcell.00331.2011 PMC3814247

[134] Helfinger V, Pálfi K, Weigert A, Schröder K. The NADPH oxidase Nox4 controls macrophage polarization in an NFκB-dependent manner. Oxid Med Cell Longev. 2019;2019:3264858. 31178956 10.1155/2019/3264858 PMC6501210

[135] Choi JY, Byeon HW, Park SO, Uyangaa E, Kim K, Eo SK. Inhibition of NADPH oxidase 2 enhances resistance to viral neuroinflammation by facilitating M1-polarization of macrophages at the extraneural tissues. J Neuroinflammation. 2024;21:115. 38698374 10.1186/s12974-024-03078-8 PMC11067137

[136] Stalin J, Garrido-Urbani S, Heitz F, Szyndralewiez C, Jemelin S, Coquoz O, et al. Inhibition of host NOX1 blocks tumor growth and enhances checkpoint inhibitor–based immunotherapy. Life Sci Alliance. 2019;2:e201800265. 31249132 10.26508/lsa.201800265 PMC6599972

[137] Palmieri EM, Gonzalez-Cotto M, Baseler WA, Davies LC, Ghesquière B, Maio N, et al. Nitric oxide orchestrates metabolic rewiring in M1 macrophages by targeting aconitase 2 and pyruvate dehydrogenase. Nat Commun. 2020;11:698. 32019928 10.1038/s41467-020-14433-7 PMC7000728

[138] Liu L, Lu Y, Martinez J, Bi Y, Lian G, Wang T, et al. Proinflammatory signal suppresses proliferation and shifts macrophage metabolism from Myc-dependent to HIF1 α dependent. Proc Natl Acad Sci USA. 2016;113:1564–9. 26811453 10.1073/pnas.1518000113 PMC4760828

[139] Lee K, Won HY, Bae MA, Hong J-H, Hwang ES. Spontaneous and aging-dependent development of arthritis in NADPH oxidase 2 deficiency through altered differentiation of CD11b+ and Th/Treg cells. Proc Natl Acad Sci USA. 2011;108:9548–53. 21593419 10.1073/pnas.1012645108 PMC3111328

[140] Roux C, Jafari SM, Shinde R, Duncan G, Cescon DW, Silvester J, et al. Reactive oxygen species modulate macrophage immunosuppressive phenotype through the upregulation of PD-L1. Proc Natl Acad Sci USA. 2019;116:4326–35. 30770442 10.1073/pnas.1819473116 PMC6410837

[141] Ford K, Hanley CJ, Mellone M, Szyndralewiez C, Heitz F, Wiesel P, et al. NOX4 inhibition potentiates immunotherapy by overcoming cancer-associated fibroblast-mediated CD8 T-cell exclusion from tumors. Cancer Res. 2020;80:1846–60. 32122909 10.1158/0008-5472.CAN-19-3158 PMC7611230

[142] Liu C, Liu L, Yang M, Li B, Yi J, Ai X, et al. A positive feedback loop between EZH2 and NOX4 regulates nucleus pulposus cell senescence in age-related intervertebral disc degeneration. Cell Div. 2020;15:2. 32025238 10.1186/s13008-020-0060-x PMC6995653

[143] Espinosa-Sotelo R, Fusté NP, Penuelas-Haro I, Alay A, Pons G, Almodóvar X, et al. Dissecting the role of the NADPH oxidase NOX4 in TGF-beta signaling in hepatocellular carcinoma. Redox Biol. 2023;65:102818. 37463530 10.1016/j.redox.2023.102818 PMC10372458

[144] Chen Y, Ai L, Zhang Y, Li X, Xu S, Yang W, et al. The EZH2-H3K27me3 axis modulates aberrant transcription and apoptosis in cyclophosphamide-induced ovarian granulosa cell injury. Cell Death Discov. 2023;9:413. 37963880 10.1038/s41420-023-01705-6 PMC10646043

[145] Guo P, Lim RC, Rajawasam K, Trinh T, Sun H, Zhang H. A methylation-phosphorylation switch controls EZH2 stability and hematopoiesis. eLife. 2024;13:e86168. 38346162 10.7554/eLife.86168 PMC10901513

[146] Sánchez-Ceinos J, Hussain S, Khan AW, Zhang L, Almahmeed W, Pernow J, et al. Repressive H3K27me3 drives hyperglycemia-induced oxidative and inflammatory transcriptional programs in human endothelium. Cardiovasc Diabetol. 2024;23:122. 38580969 10.1186/s12933-024-02196-0 PMC10998410

[147] Yang WH, Chi JT. Hippo pathway effectors YAP/TAZ as novel determinants of ferroptosis. Mol Cell Oncol. 2020;7:1699375. 31993503 10.1080/23723556.2019.1699375 PMC6961671

[148] Zhang Z, Luan Q, Hao W, Cui Y, Li Y, Li X. NOX4-derived ROS regulates aerobic glycolysis of breast cancer through YAP pathway. J Cancer. 2023;14:2562. 37670970 10.7150/jca.81099 PMC10475359

[149] Kwon A, Lee NY, Yu J-H, Choi MG, Park J, Koo JH. Mitochondrial stress activates YAP/TAZ through RhoA oxidation to promote liver injury. Cell Death Dis. 2024;15:51. 38225223 10.1038/s41419-024-06448-5 PMC10789791

[150] Zheng J, Yu H, Zhou A, Wu B, Liu J, Jia Y, et al. It takes two to tango: coupling of Hippo pathway and redox signaling in biological process. Cell Cycle. 2020;19:2760–75. 33016196 10.1080/15384101.2020.1824448 PMC7714424

[151] Thakur M, Mutyala D, Amoliga AA, Kondati R, Batra S. ROS-driven rewiring of Hippo-inflammation-polycomb axis by PFOA in 2D and 3D lung epithelial models. Toxicology. 2026;523:154426. 41654163 10.1016/j.tox.2026.154426

[152] Yang W-H, Huang Z, Wu J, Ding C-KC, Murphy SK, Chi J-T. A TAZ–ANGPTL4–NOX2 axis regulates ferroptotic cell death and chemoresistance in epithelial ovarian cancer. Mol Cancer Res. 2020;18:79–90. 31641008 10.1158/1541-7786.MCR-19-0691 PMC6942206

[153] Li Y-k, Gao A-b, Zeng T, Liu D, Zhang Q-f, Ran X-m, et al. ANGPTL4 accelerates ovarian serous cystadenocarcinoma carcinogenesis and angiogenesis in the tumor microenvironment by activating the JAK2/STAT3 pathway and interacting with ESM1. J Transl Med. 2024;22:46. 38212795 10.1186/s12967-023-04819-8 PMC10785435

[154] Xiong Y, Weng Y, Zhu S, Qin J, Feng J, Jing X, et al. NOX4 modulates breast cancer progression through cancer cell metabolic reprogramming and CD8+ T-cell antitumor activity. Front Immunol. 2025;16:1534936. 39991149 10.3389/fimmu.2025.1534936 PMC11842241

[155] Jin S, Zhou F, Katirai F, Li P-L. Lipid raft redox signaling: molecular mechanisms in health and disease. Antioxid Redox Signal. 2011;15:1043–83. 21294649 10.1089/ars.2010.3619 PMC3135227

[156] Malla R. Regulation of NADPH oxidase (Nox2) by lipid rafts in breast carcinoma cells. Int J Oncol. 2010;37:1483–93. 21042717 10.3892/ijo_00000801

[157] Eum SY, Andras I, Hennig B, Toborek M. NADPH oxidase and lipid raft-associated redox signaling are required for PCB153-induced upregulation of cell adhesion molecules in human brain endothelial cells. Toxicol Appl Pharmacol. 2009;240:299–305. 19632255 10.1016/j.taap.2009.07.022 PMC2760772

[158] Anagnostopoulou A, Camargo LL, Rodrigues D, Montezano AC, Touyz RM. Importance of cholesterol-rich microdomains in the regulation of Nox isoforms and redox signaling in human vascular smooth muscle cells. Sci Rep. 2020;10:17818. 33082354 10.1038/s41598-020-73751-4 PMC7575553

[159] Marques-da-Silva D, Lagoa R. Rafting on the evidence for lipid raft-like domains as hubs triggering environmental toxicants’ cellular effects. Molecules. 2023;28:6598. 37764374 10.3390/molecules28186598 PMC10536579

[160] Balakrishnan M, Kenworthy AK. Lipid peroxidation drives liquid–liquid phase separation and disrupts raft protein partitioning in biological membranes. J Am Chem Soc. 2024;146:1374–87. 38171000 10.1021/jacs.3c10132 PMC10797634

[161] Corrêa Costa-Beber L, Kazmirczak Moraes R, Marques Obelar Ramos J, Meira Martins LA, Toquetto AL, Fursel Pacheco J, et al. Aqueous PM(2.5) promotes lipid accumulation, classical macrophage polarization and heat shock response. Chemosphere. 2024;363:142987. 39094706 10.1016/j.chemosphere.2024.142987

[162] Cacciottolo M, Morgan TE, Saffari AA, Shirmohammadi F, Forman HJ, Sioutas C, et al. Traffic-related air pollutants (TRAP-PM) promote neuronal amyloidogenesis through oxidative damage to lipid rafts. Free Radic Biol Med. 2020;147:242–51. 31883973 10.1016/j.freeradbiomed.2019.12.023 PMC7075030

[163] Vilhardt F, van Deurs B. The phagocyte NADPH oxidase depends on cholesterol-enriched membrane microdomains for assembly. EMBO J. 2004;23:739–48. 14765128 10.1038/sj.emboj.7600066 PMC380990

[164] Li P-L, Gulbins E. Lipid rafts and redox signaling. Antioxid Redox Signal. 2007;9:1411–6. 17627466 10.1089/ars.2007.1736

[165] Chargaff E, West R. The biological significance of the thromboplastic protein of blood. J Biol Chem. 1946;166:189–97. 20273687

[166] Johnstone RM, Adam M, Hammond J, Orr L, Turbide C. Vesicle formation during reticulocyte maturation. Association of plasma membrane activities with released vesicles (exosomes). J Biol Chem. 1987;262:9412–20. 3597417

[167] Van der Pol E, Böing AN, Harrison P, Sturk A, Nieuwland R. Classification, functions, and clinical relevance of extracellular vesicles. Pharmacol Rev. 2012;64:676–705. 22722893 10.1124/pr.112.005983

[168] Zheng C, Zhang L, Sun Y, Ma Y, Zhang Y. Alveolar epithelial cell dysfunction and epithelial–mesenchymal transition in pulmonary fibrosis pathogenesis. Front Mol Biosci. 2025;12:1564176. 40343260 10.3389/fmolb.2025.1564176 PMC12058482

[169] Zhao Y, Qi Y, Xia J, Duan M, Hao C, Yao W. The role of the PI3K/AKT/mTOR pathway in mediating PD-L1 upregulation during fibroblast transdifferentiation. Int Immunopharmacol. 2024;142:113186. 39298817 10.1016/j.intimp.2024.113186

[170] Nyunoya T, Mebratu Y, Contreras A, Delgado M, Chand HS, Tesfaigzi Y. Molecular processes that drive cigarette smoke-induced epithelial cell fate of the lung. Am J Respir Cell Mol Biol. 2014;50:471–82. 24111585 10.1165/rcmb.2013-0348TR PMC4068939

[171] Huete-Acevedo J, Mas-Bargues C, Arnal-Forné M, Atencia-Rabadán S, Sanz-Ros J, Borrás C. Role of redox homeostasis in the communication between brain and liver through extracellular vesicles. Antioxidants. 2024;13:1493. 39765821 10.3390/antiox13121493 PMC11672896

[172] Burrello J, Biemmi V, Dei Cas M, Amongero M, Bolis S, Lazzarini E, et al. Sphingolipid composition of circulating extracellular vesicles after myocardial ischemia. Sci Rep. 2020;10:16182. 32999414 10.1038/s41598-020-73411-7 PMC7527456

[173] Ramasubramanian L, Jyothi H, Goldbloom-Helzner L, Light BM, Kumar P, Carney RP, et al. Development and characterization of bioinspired lipid raft nanovesicles for therapeutic applications. ACS Appl Mater Interfaces. 2022;14:54458–77. 36448709 10.1021/acsami.2c13868 PMC9756296

[174] Yang W-H, Ding C-KC, Sun T, Rupprecht G, Lin C-C, Hsu D, et al. The hippo pathway effector TAZ regulates ferroptosis in renal cell carcinoma. Cell Rep. 2019;28:2501–8.e4. 31484063 10.1016/j.celrep.2019.07.107 PMC10440760

[175] Bialik JF, Ding M, Speight P, Dan Q, Miranda MZ, Di Ciano-Oliveira C, et al. Profibrotic epithelial phenotype: a central role for MRTF and TAZ. Sci Rep. 2019;9:4323. 30867502 10.1038/s41598-019-40764-7 PMC6416270

[176] Han W, Li H, Villar VAM, Pascua AM, Dajani MI, Wang X, et al. Lipid rafts keep NADPH oxidase in the inactive state in human renal proximal tubule cells. Hypertension. 2008;51:481–7. 18195159 10.1161/HYPERTENSIONAHA.107.103275

[177] Montenegro MF, Valdivia A, Smolensky A, Verma K, Taylor WR, San Martín A. Nox4-dependent activation of cofilin mediates VSMC reorientation in response to cyclic stretching. Free Radic Biol Med. 2015;85:288–94. 25998423 10.1016/j.freeradbiomed.2015.05.011 PMC4517474

[178] Xu Z, Ding W, Deng X. PM2. 5, fine particulate matter: a novel player in the epithelial–mesenchymal transition? Front Physiol. 2019;10:1404. 31849690 10.3389/fphys.2019.01404 PMC6896848

[179] Fan X, Dong T, Yan K, Ci X, Peng L. PM2. 5 increases susceptibility to acute exacerbation of COPD via NOX4/Nrf2 redox imbalance-mediated mitophagy. Redox Biol. 2023;59:102587. 36608590 10.1016/j.redox.2022.102587 PMC9813701

[180] Wang X, Murugesan P, Zhang P, Xu S, Peng L, Wang C, et al. NADPH oxidase isoforms in COPD patients and acute cigarette smoke-exposed mice: induction of oxidative stress and lung inflammation. Antioxidants. 2022;11:1539. 10.3390/antiox11081539 PMC9405243 36009258

[181] Zou L, Xiong L, Wu T, Wei T, Liu N, Bai C, et al. NADPH oxidases regulate endothelial inflammatory injury induced by PM2. 5 via AKT/eNOS/NO axis. J Appl Toxicol. 2022;42:738–49. 34708887 10.1002/jat.4254

[182] Kang KA, Piao MJ, Fernando PDSM, Herath HMUL, Yi JM, Choi YH, et al. Particulate matter stimulates the NADPH oxidase system via AhR-mediated epigenetic modifications. Environ Pollut. 2024;347:123675. 38447650 10.1016/j.envpol.2024.123675

[183] Fernando PDSM, Piao MJ, Herath HMUL, Kang KA, Ha K-S, Chae S, et al. C-peptide ameliorates particulate matter 2.5-induced skin apoptosis by inhibiting NADPH oxidation. Biomol Ther. 2024;33:221. 10.4062/biomolther.2024.053 PMC11704398 39690967

[184] Lee HS, Park HY, Kwon SP, Kim B, Lee Y, Kim S, et al. NADPH oxidase-mediated activation of neutral sphingomyelinase is responsible for diesel particulate extract-induced keratinocyte apoptosis. Int J Mol Sci. 2020;21:1001. 10.3390/ijms21031001 PMC7037446 32028642

[185] Shin K-O, Kim S, Kim B, Park H-Y, Jung E, Kim G, et al. Euphorbia supina Extracts Block NADPH oxidase-mediated, ceramide-induced apoptosis initiated by diesel particulate matter. Pharmaceuticals. 2022;15:431. 35455428 10.3390/ph15040431 PMC9026628

[186] Kim JY, Kim JH, Kim YD, Seo JH. Ultrafine diesel exhaust particles induce apoptosis of oligodendrocytes by increasing intracellular reactive oxygen species through NADPH oxidase activation. Antioxidants. 2022;11:1031. 10.3390/antiox11051031 PMC9137819 35624895

[187] Orosz Z, Csiszar A, Labinskyy N, Smith K, Kaminski PM, Ferdinandy P, et al. Cigarette smoke-induced proinflammatory alterations in the endothelial phenotype: role of NAD(P)H oxidase activation. Am J Physiol Heart Circ Physiol. 2007;292:H130–9. 17213480 10.1152/ajpheart.00599.2006

[188] Ambrose JA, Barua RS. The pathophysiology of cigarette smoking and cardiovascular disease: an update. J Am Coll Cardiol. 2004;43:1731–7. 15145091 10.1016/j.jacc.2003.12.047

[189] Chan SMH, Brassington K, Almerdasi SA, Dobric A, De Luca SN, Coward-Smith M, et al. Inhibition of oxidative stress by apocynin attenuated chronic obstructive pulmonary disease progression and vascular injury by cigarette smoke exposure. Br J Pharmacol. 2023;180:2018–34. 36908040 10.1111/bph.16068 PMC10953324

[190] Li ZM, Xu SY, Feng YZ, Cheng YR, Xiong JB, Zhou Y, et al. The role of NOX4 in pulmonary diseases. J Cell Physiol. 2021;236:1628–37. 32780450 10.1002/jcp.30005

[191] Li M, Wang H, Lu Y, Cai J. Luteolin suppresses inflammation and oxidative stress in chronic obstructive pulmonary disease through inhibition of the NOX4-mediated NF-κB signaling pathway. Immun Inflamm Dis. 2023;11:e820. 37102667 10.1002/iid3.820 PMC10134768

[192] Sul OJ, Park SH, Choi HW, Kim DJ, Ra SW. NADPH oxidase-dependent heme oxygenase-1 expression mediates cigarette smoke-induced ferroptosis via intracellular Fe (II) accumulation. Free Radic Biol Med. 2025;237:131–46. 10.1016/j.freeradbiomed.2025.05.423 40456495

[193] Xiang G, Gong L, Wang K, Sun X, Liu Z, Cai Q. Suppression of NOX2-derived reactive oxygen species (ROS) reduces epithelial-to-MesEnchymal transition through blocking SiO2-regulated JNK activation. Toxics. 2025;13:365. 40423444 10.3390/toxics13050365 PMC12115725

[194] Wang W, Liang X, Liu X, Bai J, Zhang W, Li W, et al. NOX4 blockade suppresses titanium nanoparticle-induced bone destruction via activation of the Nrf2 signaling pathway. J Nanobiotechnology. 2022;20:241. 35606794 10.1186/s12951-022-01413-w PMC9125939

[195] Sun X, Yang Y, Shi J, Wang C, Yu Z, Zhang H. NOX4-and Nrf2-mediated oxidative stress induced by silver nanoparticles in vascular endothelial cells. J Appl Toxicol. 2017;37:1428–37. 28815642 10.1002/jat.3511

[196] Liu X, Chen R, Peng Y, Zhou Y, Xia M, Wu X, et al. Perfluorooctanoic acid (PFOA) induces cardiotoxicity by activating the Keap1/Nrf2 pathway in zebrafish (*Danio rerio*) embryos. Ecotoxicol Environ Saf. 2024;285:117098. 39366304 10.1016/j.ecoenv.2024.117098

[197] Zhao M, Jiang Q, Wang W, Geng M, Wang M, Han Y, et al. The roles of reactive oxygen species and nitric oxide in perfluorooctanoic acid-induced developmental cardiotoxicity and l-Carnitine mediated protection. Int J Mol Sci. 2017;18:1229. 10.3390/ijms18061229 PMC5486052 28594376

[198] Jiang Q, Lust RM, Strynar MJ, Dagnino S, DeWitt JC. Perflurooctanoic acid induces developmental cardiotoxicity in chicken embryos and hatchlings. Toxicology. 2012;293:97–106. 22273728 10.1016/j.tox.2012.01.005

[199] Guo Y, Yuan J, Ni H, Ji J, Zhong S, Zheng Y, et al. Perfluorooctanoic acid-induced developmental cardiotoxicity in chicken embryo: Roles of miR-490-5p. Environ Pollut. 2022;312:120022. 36028080 10.1016/j.envpol.2022.120022

[200] Wang Q, Chen W, Zhang B, Gao Z, Zhang Q, Deng H, et al. Perfluorooctanoic acid induces hepatocellular endoplasmic reticulum stress and mitochondrial-mediated apoptosis in vitro via endoplasmic reticulum-mitochondria communication. Chem Biol Interact. 2022;354. 109844. 35123991 10.1016/j.cbi.2022.109844

[201] Heymes C, Bendall JK, Ratajczak P, Cave AC, Samuel J-L, Hasenfuss G, et al. Increased myocardial NADPH oxidase activity in human heart failure. JACC. 2003;41:2164–71. 12821241 10.1016/s0735-1097(03)00471-6

[202] Altenhöfer S, Radermacher KA, Kleikers PW, Wingler K, Schmidt HH. Evolution of NADPH oxidase inhibitors: selectivity and mechanisms for target engagement. Antioxid Redox Signal. 2015;23:406–27. 24383718 10.1089/ars.2013.5814 PMC4543484

[203] Tazzeo T, Worek F, Janssen L. The NADPH oxidase inhibitor diphenyleneiodonium is also a potent inhibitor of cholinesterases and the internal Ca(2+) pump. Br J Pharmacol. 2009;158:790–6. 19788497 10.1111/j.1476-5381.2009.00394.x PMC2765598

[204] Suh KS, Choi EM, Kim YJ, Hong SM, Park SY, Rhee SY, et al. Perfluorooctanoic acid induces oxidative damage and mitochondrial dysfunction in pancreatic β-cells. Mol Med Rep. 2017;15:3871–8. 28440430 10.3892/mmr.2017.6452

[205] Elumalai S, Karunakaran U, Won KC, Chung SM, Moon JS. Perfluorooctane sulfonate-induced oxidative stress contributes to pancreatic β-apoptosis by inhibiting cyclic adenosine monophosphate pathway: prevention by pentoxifylline. Environ Pollut. 2023;320:120959. 36621715 10.1016/j.envpol.2022.120959

[206] Thota S, Begum R, Mutyala D, Bidarimath N, Thakur M, Sarkar B, et al. Unraveling the Hsp70-ROS-autophagy axis in pentachlorophenol-challenged lung and liver epithelial cells. Arch Toxicol. 2025;99:2039–62. 40189663 10.1007/s00204-025-03983-8

[207] Sule RO, Condon L, Gomes AV. A common feature of pesticides: oxidative stress—the role of oxidative stress in pesticide-induced toxicity. Oxid Med Cell Longev. 2022;2022:5563759. 35096268 10.1155/2022/5563759 PMC8791758

[208] Chiappini F, Pontillo C, Randi AS, Alvarez L, Kleiman de Pisarev DL. Reactive oxygen species and extracellular signal-regulated kinase 1/2 mediate hexachlorobenzene-induced cell death in FRTL-5 rat thyroid cells. Toxicol Sci. 2013;134:276–90. 23708402 10.1093/toxsci/kft117

[209] Tijani AS, Farombi EO, Olori DO. Thymol coadministration abrogates hexachlorobenzene-induced reproductive toxicities in male rats. Hum Exp Toxicol. 2023;42:09603271221149201. 10.1177/09603271221149201 36606752

[210] Martello LB, Tjeerdema RS. Combined effects of pentachlorophenol and salinity stress on chemiluminescence activity in two species of abalone. Aquat Toxicol. 2001;51:351–62. 11090895 10.1016/s0166-445x(00)00110-7

[211] Bagaitkar J, Stoyan I, Pech N, Huang G, Dinauer MC. NADPH Oxidase Limits Sterile Inflammation By Modulating IL-1/G-CSF-Driven Neutrophilic Inflammation. Blood. 2014;124:458.

[212] Hasanuzzaman M, Raihan MRH, Masud AAC, Rahman K, Nowroz F, Rahman M, et al. Regulation of reactive oxygen species and antioxidant defense in plants under salinity. Int J Mol Sci. 2021;22:9326. 10.3390/ijms22179326 PMC8430727 34502233

[213] Paik Y-H, Iwaisako K, Seki E, Inokuchi S, Schnabl B, Österreicher CH, et al. The nicotinamide adenine dinucleotide phosphate oxidase (NOX) homologs NOX1 and NOX2/gp91phox mediate hepatic fibrosis in mice. Hepatology. 2011;53:1730–41. 21384410 10.1002/hep.24281 PMC3082608

[214] Mustieles V, Perez-Carrascosa FM, Leon J, Lange T, Bonde J-P, Gomez-Pena C, et al. Adipose tissue redox microenvironment as a potential link between persistent organic pollutants and the 16-year incidence of non-hormone-dependent cancer. Environ Sci Technol. 2021;55:9926–37. 34180659 10.1021/acs.est.0c08180 PMC8474112

[215] Hecker L, Vittal R, Jones T, Jagirdar R, Luckhardt TR, Horowitz JC, et al. NADPH oxidase-4 mediates myofibroblast activation and fibrogenic responses to lung injury. Nat Med. 2009;15:1077–81. 19701206 10.1038/nm.2005 PMC2743335

[216] Cucoranu I, Clempus R, Dikalova A, Phelan PJ, Ariyan S, Dikalov S, et al. NAD (P) H oxidase 4 mediates transforming growth factor-β1–induced differentiation of cardiac fibroblasts into myofibroblasts. Circ Res. 2005;97:900–7. 16179589 10.1161/01.RES.0000187457.24338.3D

[217] Zhu Y, Kalen AL, Li L, Lehmler HJ, Robertson LW, Goswami PC, et al. Polychlorinated-biphenyl-induced oxidative stress and cytotoxicity can be mitigated by antioxidants after exposure. Free Radic Biol Med. 2009;47:1762–71. 19796678 10.1016/j.freeradbiomed.2009.09.024 PMC2785439

[218] Sharifi-Rad M, Anil Kumar NV, Zucca P, Varoni EM, Dini L, Panzarini E, et al. Lifestyle, oxidative stress, and antioxidants: back and forth in the pathophysiology of chronic diseases. Front Physiol. 2020;11:552535. 10.3389/fphys.2020.00694 PMC7347016 32714204

[219] Skonieczna M, Hejmo T, Poterala-Hejmo A, Cieslar-Pobuda A, Buldak RJ. NADPH oxidases: insights into selected functions and mechanisms of action in cancer and stem cells. Oxid Med Cell Longev. 2017;2017:9420539. 28626501 10.1155/2017/9420539 PMC5463201

[220] Kato K, Hecker L. NADPH oxidases: pathophysiology and therapeutic potential in age-associated pulmonary fibrosis. Redox Biol. 2020;33:101541. 32360174 10.1016/j.redox.2020.101541 PMC7251244

[221] Piera-Velazquez S, Jimenez SA. Oxidative stress induced by reactive oxygen species (ROS) and NADPH oxidase 4 (NOX4) in the pathogenesis of the fibrotic process in systemic sclerosis: a promising therapeutic target. J Clin Med. 2021;10:4791. 34682914 10.3390/jcm10204791 PMC8539594

[222] Li D, Cong Z, Yang C, Zhu X. Inhibition of LPS-induced Nox2 activation by VAS2870 protects alveolar epithelial cells through eliminating ROS and restoring tight junctions. Biochem Biophys Res Commun. 2020;524:575–81. 32019675 10.1016/j.bbrc.2020.01.134

[223] Cavinato L, Genise E, Luly FR, Di Domenico EG, Del Porto P, Ascenzioni F. Escaping the phagocytic oxidative burst: the role of SODB in the survival of *Pseudomonas aeruginosa* within macrophages. Front Microbiol. 2020;11:326. 32210934 10.3389/fmicb.2020.00326 PMC7077434

[224] Ma M, Shi F, Zhai R, Wang H, Li K, Xu C, et al. TGF-β promote epithelial–mesenchymal transition via NF-κB/NOX4/ROS signaling pathway in lung cancer cells. Mol Biol Rep. 2021;48:2365–75. 33792826 10.1007/s11033-021-06268-2

[225] Thannickal VJ, Toews GB, White ES, Lynch JP 3rd, Martinez FJ. Mechanisms of pulmonary fibrosis. Annu Rev Med. 2004;55:395–417. 14746528 10.1146/annurev.med.55.091902.103810

[226] Bezerra FS, Lanzetti M, Nesi RT, Nagato AC, Silva CPE, Kennedy-Feitosa E, et al. Oxidative stress and inflammation in acute and chronic lung injuries. Antioxidants. 2023;12:548. 10.3390/antiox12030548 PMC10045332 36978796

[227] Celli BR, Wedzicha JA. Update on clinical aspects of chronic obstructive pulmonary disease. N Engl J Med. 2019;381:1257–66. 31553837 10.1056/NEJMra1900500

[228] Vlahos R, Bozinovski S, Jones JE, Powell J, Gras J, Lilja A, et al. Differential protease, innate immunity, and NF-kappaB induction profiles during lung inflammation induced by subchronic cigarette smoke exposure in mice. Am J Physiol Lung Cell Mol Physiol. 2006;290:L931–45. 16361358 10.1152/ajplung.00201.2005

[229] Zhang P, Zhang Y, Wang L, Wang X, Xu S, Zhai Z, et al. Reversal of NADPH oxidase-dependent early oxidative and inflammatory responses in chronic obstructive pulmonary disease by Puerarin. Oxid Med Cell Longev. 2022;2022:5595781. 35651727 10.1155/2022/5595781 PMC9151001

[230] Seimetz M, Sommer N, Bednorz M, Pak O, Veith C, Hadzic S, et al. Author Correction: NADPH oxidase subunit NOXO1 is a target for emphysema treatment in COPD. Nat Metab. 2020;2:648. 32694792 10.1038/s42255-020-0248-z

[231] Rajesh R, Atallah R, Bärnthaler T. Dysregulation of metabolic pathways in pulmonary fibrosis. Pharmacol Ther. 2023;246:108436. 37150402 10.1016/j.pharmthera.2023.108436

[232] Martin-Garrido A, Brown DI, Lyle AN, Dikalova A, Seidel-Rogol B, Lassègue B, et al. NADPH oxidase 4 mediates TGF-β-induced smooth muscle α-actin via p38MAPK and serum response factor. Free Radic Biol Med. 2011;50:354–62. 21074607 10.1016/j.freeradbiomed.2010.11.007 PMC3032946

[233] Sato N, Takasaka N, Yoshida M, Tsubouchi K, Minagawa S, Araya J, et al. Metformin attenuates lung fibrosis development via NOX4 suppression. Respir Res. 2016;17:107. 27576730 10.1186/s12931-016-0420-x PMC5006432

[234] Louzada RA, Corre R, El Hassani RA, Meziani L, Jaillet M, Cazes A, et al. NADPH oxidase DUOX1 sustains TGF-β1 signaling and promotes lung fibrosis. Eur Respir J. 2021;57:1901949. 10.1183/13993003.01949-2019 32764116

[235] Wang P, Zhao Q, Zhu X, Cao S, Williams JP, An J. Ozone therapy ameliorates lipopolysaccharide-induced acute lung injury in mice by inhibiting the NLRP3/ASC/caspase-1 axis. Shock. 2025;63:487–94. 10.1097/SHK.0000000000002525 PMC11882185 39637244

[236] Harijith A, Basa P, Ha A, Thomas J, Jafri A, Fu P, et al. NOX4 mediates epithelial cell death in hyperoxic acute lung injury through mitochondrial reactive oxygen species. Front Pharmacol. 2022;13:880878. 35662702 10.3389/fphar.2022.880878 PMC9160661

[237] Zhang L, Zhang Y, Qin X, Jiang X, Zhang J, Mao L, et al. Recombinant ACE2 protein protects against acute lung injury induced by SARS-CoV-2 spike RBD protein. Crit Care. 2022;26:171. 35681221 10.1186/s13054-022-04034-9 PMC9178547

[238] Cai B, Liu M, Li J, Xu D, Li J. Cigarette smoke extract amplifies NADPH oxidase-dependent ROS production to inactivate PTEN by oxidation in BEAS-2B cells. Food Chem Toxicol. 2021;150:112050. 33577944 10.1016/j.fct.2021.112050

[239] Meganathan V, Hamilton CE, Natarajan K, Keshava S, Boggaram V. NADPH and xanthine oxidases control induction of inflammatory mediator expression by organic dust in the lung. FASEB J. 2022;36:e22381. 35661421 10.1096/fj.202100732R

[240] Sorce S, Krause K-H. NOX enzymes in the central nervous system: from signaling to disease. Antioxid Redox Signal. 2009;11:2481–504. 19309263 10.1089/ars.2009.2578

[241] Diebold I, Petry A, Hess J, Görlach A. The NADPH oxidase subunit NOX4 is a new target gene of the hypoxia-inducible factor-1. Mol Biol Cell. 2010;21:2087–96. 20427574 10.1091/mbc.E09-12-1003 PMC2883952

[242] Lin Z, Ying C, Si X, Xue N, Liu Y, Zheng R, et al. NOX4 exacerbates Parkinson’s disease pathology by promoting neuronal ferroptosis and neuroinflammation. Neural Regen Res. 2025;20:2038–52. 38993139 10.4103/NRR.NRR-D-23-01265 PMC11691449

[243] Tu D, Velagapudi R, Gao Y, Hong J-S, Zhou H, Gao H-M. Activation of neuronal NADPH oxidase NOX2 promotes inflammatory neurodegeneration. Free Radic Biol Med. 2023;200:47–58. 36870375 10.1016/j.freeradbiomed.2023.03.001 PMC10164140

[244] Alam SS, Watanabe Y, Steeno BL, Dutta S, Szilagyi HA, Wei A, et al. Neuronal NADPH oxidase is required for neurite regeneration of Aplysia bag cell neurons. J Neurochem. 2023;167:505–19. 37818836 10.1111/jnc.15977 PMC10842957

[245] Malkov A, Popova I, Ivanov A, Jang SS, Yoon SY, Osypov A, et al. Aβ initiates brain hypometabolism, network dysfunction and behavioral abnormalities via NOX2-induced oxidative stress in mice. Commun Biol. 2021;4:1054. 34504272 10.1038/s42003-021-02551-x PMC8429759

[246] Brunetti L, Francavilla F, Santo A, Vitone D, Leopoldo M, Lacivita E. Beyond radical scavengers: focus on NADPH Oxidases (NOX) Inhibitors as New Agents for Antioxidant Therapy in Alzheimer’s Disease. Antioxidants. 2026;15:17. 10.3390/antiox15010017 PMC12837194 41596076

[247] Mukhina KA, Kechko OI, Osypov AA, Petrushanko IY, Makarov AA, Mitkevich VA, et al. Short-term inhibition of NOX2 prevents the development of Aβ-induced pathology in mice. Antioxidants. 2025;14:663. 40563297 10.3390/antiox14060663 PMC12189101

[248] Casas AI, Geuss E, Kleikers PW, Mencl S, Herrmann AM, Buendia I, et al. NOX4-dependent neuronal autotoxicity and BBB breakdown explain the superior sensitivity of the brain to ischemic damage. Proc Natl Acad Sci USA. 2017;114:12315–20. 29087944 10.1073/pnas.1705034114 PMC5699031

[249] Ma MW, Wang J, Zhang Q, Wang R, Dhandapani KM, Vadlamudi RK, et al. NADPH oxidase in brain injury and neurodegenerative disorders. Mol Neurodegener. 2017;12:7. 28095923 10.1186/s13024-017-0150-7 PMC5240251

[250] Bae YS, Lee JH, Choi SH, Kim S, Almazan F, Witztum JL, et al. Macrophages generate reactive oxygen species in response to minimally oxidized low-density lipoprotein: Toll-like receptor 4–and spleen tyrosine kinase–dependent activation of NADPH oxidase 2. Circ Res. 2009;104:210–8. 19096031 10.1161/CIRCRESAHA.108.181040 PMC2720065

[251] Zhang L, Xiao Y, Wang D, Han X, Zhou R, Zhang H, et al. NOX2/NLRP3-inflammasome-dependent microglia activation promotes as (III)-induced learning and memory impairments in developmental rats. Toxics. 2025;13:538. 40710983 10.3390/toxics13070538 PMC12299122

[252] Maimaiti Y, Su T, Zhang Z, Ma L, Zhang Y, Xu H. NOX4-mediated astrocyte ferroptosis in Alzheimer’s disease. Cell Biosci. 2024;14:88. 38956702 10.1186/s13578-024-01266-w PMC11218381

[253] Park MW, Cha HW, Kim J, Kim JH, Yang H, Yoon S, et al. NOX4 promotes ferroptosis of astrocytes by oxidative stress-induced lipid peroxidation via the impairment of mitochondrial metabolism in Alzheimer’s diseases. Redox Biol. 2021;41:101947. 33774476 10.1016/j.redox.2021.101947 PMC8027773

[254] Kim J, Moon J-S. Molecular roles of NADPH oxidase-mediated oxidative stress in Alzheimer’s disease: isoform-specific contributions. Int J Mol Sci. 2024;25:12299. 39596364 10.3390/ijms252212299 PMC11594809

[255] Negi S, Khurana N, Duggal N. The misfolding mystery: α-synuclein and the pathogenesis of Parkinson’s disease. Neurochem Int. 2024;177:105760. 38723900 10.1016/j.neuint.2024.105760

[256] Pullara F, Forsmann MC, General IJ, Ayoob JC, Furbee E, Castro SL, et al. NADPH oxidase 2 activity disrupts Calmodulin/CaMKIIα complex via redox modifications of CaMKIIα-contained Cys30 and Cys289: Implications in Parkinson’s disease. Redox Biol. 2024;75:103254. 38968922 10.1016/j.redox.2024.103254 PMC11278932

[257] Bido S, Muggeo S, Massimino L, Marzi MJ, Giannelli SG, Melacini E, et al. Microglia-specific overexpression of α-synuclein leads to severe dopaminergic neurodegeneration by phagocytic exhaustion and oxidative toxicity. Nat Commun. 2021;12:6237. 34716339 10.1038/s41467-021-26519-x PMC8556263

[258] Chandran R, Kim T, Mehta SL, Udho E, Chanana V, Cengiz P, et al. A combination antioxidant therapy to inhibit NOX2 and activate Nrf2 decreases secondary brain damage and improves functional recovery after traumatic brain injury. J Cereb Blood Flow Metab. 2018;38:1818–27. 29083257 10.1177/0271678X17738701 PMC6168911

[259] Boonpraman N, Yoon S, Kim CY, Moon J-S, Yi SS. NOX4 as a critical effector mediating neuroinflammatory cytokines, myeloperoxidase and osteopontin, specifically in astrocytes in the hippocampus in Parkinson’s disease. Redox Biol. 2023;62:102698. 37058998 10.1016/j.redox.2023.102698 PMC10123376

[260] Surace MJ, Block ML. Targeting microglia-mediated neurotoxicity: the potential of NOX2 inhibitors. Cell Mol Life Sci. 2012;69:2409–27. 22581365 10.1007/s00018-012-1015-4 PMC3677079

[261] Ma Y, Zhang X, Xu J, Luo R, Li S, Su H, et al. Complement receptor 3 regulates microglial exosome release and related neurotoxicity via NADPH oxidase in neuroinflammation associated with parkinson’s disease. Antioxidants. 2025;14:963. 40867858 10.3390/antiox14080963 PMC12383149

[262] Kasai S, Shimizu S, Tatara Y, Mimura J, Itoh K. Regulation of Nrf2 by mitochondrial reactive oxygen species in physiology and pathology. Biomolecules. 2020;10:320. 32079324 10.3390/biom10020320 PMC7072240

[263] Magnusson A, Wu R, Demirel I. *Porphyromonas gingivalis* triggers microglia activation and neurodegenerative processes through NOX4. Front Cell Infect Microbiol. 2024;14:1451683. 39469453 10.3389/fcimb.2024.1451683 PMC11513391

[264] Li S, Guo Z, Liu J, Ma Y, Zhang X, Hou L, et al. CD11b-NOX2 mutual regulation-mediated microglial exosome release contributes to rotenone-induced inflammation and neurotoxicity in BV2 microglia and primary cultures. Free Radic Biol Med. 2024;224:436–46. 39265792 10.1016/j.freeradbiomed.2024.09.008

[265] Li Q, Spencer NY, Pantazis NJ, Engelhardt JF. Alsin and SOD1G93A proteins regulate endosomal reactive oxygen species production by glial cells and proinflammatory pathways responsible for neurotoxicity. J Biol Chem. 2011;286:40151–62. 21937428 10.1074/jbc.M111.279711 PMC3220533

[266] Li W, Yang S. Targeting oxidative stress for the treatment of ischemic stroke: Upstream and downstream therapeutic strategies. Brain Circ. 2016;2:153–63. 30276293 10.4103/2394-8108.195279 PMC6126224

[267] Kleinschnitz C, Grund H, Wingler K, Armitage ME, Jones E, Mittal M, et al. Poststroke inhibition of induced NADPH oxidase type 4 prevents oxidative stress and neurodegeneration. PLoS Biol. 2010;8:e1000479. 20877715 10.1371/journal.pbio.1000479 PMC2943442

[268] Hook JS, Cao M, Potera RM, Alsmadi NZ, Schmidtke DW, Moreland JG. Nox2 regulates platelet activation and NET formation in the lung. Front Immunol. 2019;10:1472. 31338092 10.3389/fimmu.2019.01472 PMC6626916

[269] Xie J, Hong E, Ding B, Jiang W, Zheng S, Xie Z, et al. Inhibition of NOX4/ROS suppresses neuronal and blood‒brain barrier injury by attenuating oxidative stress after intracerebral hemorrhage. Front Cell Neurosci. 2020;14:578060. 33281556 10.3389/fncel.2020.578060 PMC7691600

[270] Deliyanti D, Wilkinson-Berka JL. Inhibition of NOX1/4 with GKT137831: a potential novel treatment to attenuate neuroglial cell inflammation in the retina. J Neuroinflammation. 2015;12:136. 26219952 10.1186/s12974-015-0363-z PMC4518508

[271] Harraz MM, Marden JJ, Zhou W, Zhang Y, Williams A, Sharov VS, et al. SOD1 mutations disrupt redox-sensitive Rac regulation of NADPH oxidase in a familial ALS model. J Clin Investig. 2008;118:659–70. 18219391 10.1172/JCI34060 PMC2213375

[272] Zhao W, Beers DR, Henkel JS, Zhang W, Urushitani M, Julien JP, et al. Extracellular mutant SOD1 induces microglial-mediated motoneuron injury. Glia. 2010;58:231–43. 19672969 10.1002/glia.20919 PMC2784168

[273] Sala G, Arosio A, Conti E, Beretta S, Lunetta C, Riva N, et al. Riluzole selective antioxidant effects in cell models expressing amyotrophic lateral sclerosis endophenotypes. Clin Psychopharmacol Neurosci. 2019;17:438. 31352711 10.9758/cpn.2019.17.3.438 PMC6705104

[274] Tamaki Y, Ross JP, Alipour P, Castonguay C, Li B, Catoire H, et al. Spinal cord extracts of amyotrophic lateral sclerosis spread TDP-43 pathology in cerebral organoids. PLoS Genet. 2023;19:e1010606. 36745687 10.1371/journal.pgen.1010606 PMC9934440

[275] Hu C-F, Wu S-P, Lin G-J, Shieh C-C, Hsu C-S, Chen J-W, et al. Microglial Nox2 plays a key role in the pathogenesis of experimental autoimmune encephalomyelitis. Front Immunol. 2021;12:638381. 33868265 10.3389/fimmu.2021.638381 PMC8050344

[276] Singh PK, Saadi A, Sheeni Y, Shekh-Ahmad T. Specific inhibition of NADPH oxidase 2 modifies chronic epilepsy. Redox Biol. 2022;58:102549. 36459714 10.1016/j.redox.2022.102549 PMC9712695

[277] Singh PK, Maurya S, Saadi A, Shekh-Ahmad T. Targeting NOX2 mitigates seizure susceptibility, oxidative stress, and neuroinflammation in the pentylenetetrazol seizure model. Free Radic Biol Med. 2025. 10.1016/j.freeradbiomed.2025.05.386 40345502

[278] Fan LM, Liu F, Du J, Geng L, Li J-M. Inhibition of endothelial Nox2 activation by LMH001 protects mice from angiotensin II-induced vascular oxidative stress, hypertension and aortic aneurysm. Redox Biol. 2022;51:102269. 35276443 10.1016/j.redox.2022.102269 PMC8908273

[279] Szekeres FL, Walum E, Wikström P, Arner A. A small molecule inhibitor of Nox2 and Nox4 improves contractile function after ischemia–reperfusion in the mouse heart. Sci Rep. 2021;11:11970. 34099836 10.1038/s41598-021-91575-8 PMC8184855

[280] Hahner F, Moll F, Warwick T, Hebchen DM, Buchmann GK, Epah J, et al. Nox4 promotes endothelial differentiation through chromatin remodeling. Redox Biol. 2022;55:102381. 35810713 10.1016/j.redox.2022.102381 PMC9287364

[281] Camargo LL, Montezano AC, Hussain M, Wang Y, Zou Z, Rios FJ, et al. Central role of c-Src in NOX5-mediated redox signaling in vascular smooth muscle cells in human hypertension. Cardiovasc Res. 2022;118:1359–73. 34320175 10.1093/cvr/cvab171 PMC8953456

[282] Vendrov AE, Sumida A, Canugovi C, Lozhkin A, Hayami T, Madamanchi NR, et al. NOXA1-dependent NADPH oxidase regulates redox signaling and phenotype of vascular smooth muscle cell during atherogenesis. Redox Biol. 2019;21:101063. 30576919 10.1016/j.redox.2018.11.021 PMC6302039

[283] Hancock M, Hafstad AD, Nabeebaccus AA, Catibog N, Logan A, Smyrnias I, et al. Myocardial NADPH oxidase-4 regulates the physiological response to acute exercise. eLife. 2018;7:e41044. 30589411 10.7554/eLife.41044 PMC6307857

[284] Schröder K, Zhang M, Benkhoff S, Mieth A, Pliquett R, Kosowski J, et al. Nox4 is a protective reactive oxygen species generating vascular NADPH oxidase. Circ Res. 2012;110:1217–25. 22456182 10.1161/CIRCRESAHA.112.267054

[285] Ding J, Yu M, Jiang J, Luo Y, Zhang Q, Wang S, et al. Angiotensin II decreases endothelial nitric oxide synthase phosphorylation via AT1R Nox/ROS/PP2A pathway. Front Physiol. 2020;11:566410. 33162896 10.3389/fphys.2020.566410 PMC7580705

[286] Münzel T, Camici GG, Maack C, Bonetti NR, Fuster V, Kovacic JC. Impact of oxidative stress on the heart and vasculature: part 2 of a 3-part series. J Am Coll Cardiol. 2017;70:212–29. 28683969 10.1016/j.jacc.2017.05.035 PMC5663297

[287] Go Y-M, Son DJ, Park H, Orr M, Hao L, Takabe W, et al. Disturbed flow enhances inflammatory signaling and atherogenesis by increasing thioredoxin-1 level in endothelial cell nuclei. PLoS ONE. 2014;9:e108346. 25265386 10.1371/journal.pone.0108346 PMC4180949

[288] Valente AJ, Irimpen AM, Siebenlist U, Chandrasekar B. OxLDL induces endothelial dysfunction and death via TRAF3IP2: inhibition by HDL3 and AMPK activators. Free Radic Biol Med. 2014;70:117–28. 24561578 10.1016/j.freeradbiomed.2014.02.014 PMC4006317

[289] Judkins CP, Diep H, Broughton BR, Mast AE, Hooker EU, Miller AA, et al. Direct evidence of a role for Nox2 in superoxide production, reduced nitric oxide bioavailability, and early atherosclerotic plaque formation in ApoE-/- mice. Am J Physiol Heart Circ Physiol. 2010;298:H24–32. 19837950 10.1152/ajpheart.00799.2009

[290] Wang Y, Liu XY, Wang Y, Zhao WX, Li FD, Guo PR, et al. NOX2 inhibition stabilizes vulnerable plaques by enhancing macrophage efferocytosis via MertK/PI3K/AKT pathway. Redox Biol. 2023;64:102763. 37354827 10.1016/j.redox.2023.102763 PMC10320254

[291] Douglas G, Bendall JK, Crabtree MJ, Tatham AL, Carter EE, Hale AB, et al. Endothelial-specific Nox2 overexpression increases vascular superoxide and macrophage recruitment in ApoE−/− mice. Cardiovasc Res. 2012;94:20–9. 22287576 10.1093/cvr/cvs026 PMC3307381

[292] Alves-Lopes R, Lacchini S, Neves KB, Harvey A, Montezano AC, Touyz RM. Vasoprotective effects of NOX4 are mediated via polymerase and transient receptor potential melastatin 2 cation channels in endothelial cells. J Hypertens. 2023;41:1389–400. 37272080 10.1097/HJH.0000000000003478 PMC10399938

[293] Langbein H, Brunssen C, Hofmann A, Cimalla P, Brux M, Bornstein SR, et al. NADPH oxidase 4 protects against development of endothelial dysfunction and atherosclerosis in LDL receptor deficient mice. Eur Heart J. 2016;37:1753–61. 26578199 10.1093/eurheartj/ehv564 PMC4900759

[294] Nan X, Can X, Hui L. Inhibition of NOX4 prevents the development of atherosclerosis in Ldlr (-/-) mice. Int J Clin Exp Med. 2019;12:11265–72.

[295] Schürmann C, Rezende F, Kruse C, Yasar Y, Löwe O, Fork C, et al. The NADPH oxidase Nox4 has anti-atherosclerotic functions. Eur Heart J. 2015;36:3447–56. 26385958 10.1093/eurheartj/ehv460 PMC4751217

[296] Yu W, Li S, Wu H, Hu P, Chen L, Zeng C, et al. Endothelial Nox4 dysfunction aggravates atherosclerosis by inducing endoplasmic reticulum stress and soluble epoxide hydrolase. Free Radic Biol Med. 2021;164:44–57. 33418110 10.1016/j.freeradbiomed.2020.12.450

[297] Furmanik M, Chatrou M, van Gorp R, Akbulut A, Willems B, Schmidt H, et al. Reactive oxygen-forming Nox5 links vascular smooth muscle cell phenotypic switching and extracellular vesicle-mediated vascular calcification. Circ Res. 2020;127:911–27. 32564697 10.1161/CIRCRESAHA.119.316159

[298] Guzik TJ, Chen W, Gongora MC, Guzik B, Lob HE, Mangalat D, et al. Calcium dependent Nox5 NADPH oxidase contributes to vascular oxidative stress in human coronary artery disease. J Am Coll Cardiol. 2008;52:1803. 19022160 10.1016/j.jacc.2008.07.063 PMC2593790

[299] Kamhieh-Milz J, Chen L, Goettsch C, Pfefferkorn AM, Hofmann A, Brunssen C, et al. Ang II promotes ET-1 production by regulating NOX2 activity through transcription factor Oct-1. Arterioscler Thromb Vasc Biol. 2023;43:1429–40. 37381986 10.1161/ATVBAHA.122.318764

[300] Harrison CB, Trevelin SC, Richards DA, Santos CX, Sawyer G, Markovinovic A, et al. Fibroblast Nox2 (NADPH oxidase-2) regulates ANG II (angiotensin II)–induced vascular remodeling and hypertension via paracrine signaling to vascular smooth muscle cells. Arterioscler Thromb Vasc Biol. 2021;41:698–710. 33054395 10.1161/ATVBAHA.120.315322 PMC7837692

[301] Pavlov TS, Palygin O, Isaeva E, Levchenko V, Khedr S, Blass G, et al. NOX4-dependent regulation of ENaC in hypertension and diabetic kidney disease. FASEB J. 2020;34:13396–408. 32799394 10.1096/fj.202000966RR PMC7722042

[302] Siques P, Pena E, Brito J, El Alam S. Oxidative stress, kinase activation, and inflammatory pathways involved in effects on smooth muscle cells during pulmonary artery hypertension under hypobaric hypoxia exposure. Front Physiol. 2021;12:690341. 34434114 10.3389/fphys.2021.690341 PMC8381601

[303] Looi YH, Grieve DJ, Siva A, Walker SJ, Anilkumar N, Cave AC, et al. Involvement of Nox2 NADPH oxidase in adverse cardiac remodeling after myocardial infarction. Hypertension. 2008;51:319–25. 18180403 10.1161/HYPERTENSIONAHA.107.101980

[304] Liu Y, Wu Z, Ji M, Huang T, Meng P, Xu T, et al. NADPH oxidase 2 inhibitor GSK2795039 prevents against cardiac remodeling after MI through reducing oxidative stress and mitochondrial dysfunction. Eur J Pharmacol. 2025;997:177483. 40057160 10.1016/j.ejphar.2025.177483

[305] Vendrov AE, Xiao H, Lozhkin A, Hayami T, Hu G, Brody MJ, et al. Cardiomyocyte NOX4 regulates resident macrophage-mediated inflammation and diastolic dysfunction in stress cardiomyopathy. Redox Biol. 2023;67:102937. 37871532 10.1016/j.redox.2023.102937 PMC10598408

[306] Zhao H-P, Ma Y, Zhang X-J, Guo H-X, Yang B, Chi R-F, et al. NADPH oxidase 2 inhibitor GSK2795039 prevents doxorubicin-induced cardiac atrophy by attenuating cardiac sympathetic nerve terminal abnormalities and myocyte autophagy. Eur J Pharmacol. 2024;967:176351. 38290568 10.1016/j.ejphar.2024.176351

[307] Cully TR, Rodney GG. Nox4–RyR1–Nox2: Regulators of microdomain signaling in skeletal muscle. Redox Biol. 2020;36:101557. 32506037 10.1016/j.redox.2020.101557 PMC7283154

[308] Zhang M, Brewer AC, Schröder K, Santos CX, Grieve DJ, Wang M et al. NADPH oxidase-4 mediates protection against chronic load-induced stress in mouse hearts by enhancing angiogenesis. Proc Natl Acad Sci USA. 2010;107:18121–6. 10.1073/pnas.1009700107 PMC2964252 20921387

[309] Zhao G-J, Zhao C-L, Ouyang S, Deng K-Q, Zhu L, Montezano AC, et al. Ca2+-dependent NOX5 (NADPH Oxidase 5) exaggerates cardiac hypertrophy through reactive oxygen species production. Hypertension. 2020;76:827–38. 32683902 10.1161/HYPERTENSIONAHA.120.15558

[310] Ma Y, Zhao H-P, Yang L-G, Li L, Wang A-L, Zhang X-J, et al. NADPH oxidase 2 mediates cardiac sympathetic denervation and myocyte autophagy, resulting in cardiac atrophy and dysfunction in doxorubicin-induced cardiomyopathy. Sci Rep. 2024;14:6971. 38521855 10.1038/s41598-024-57090-2 PMC10960835

[311] Cheng D, Chen L, Tu W, Wang H, Wang Q, Meng L, et al. Protective effects of valsartan administration on doxorubicin‑induced myocardial injury in rats and the role of oxidative stress and NOX2/NOX4 signaling. Mol Med Rep. 2020;22:4151–62. 33000246 10.3892/mmr.2020.11521 PMC7533445

[312] Zeng H, Zou P, Chen Y, Zhang P, Shao L. NOX4 aggravates doxorubicin-induced cardiomyocyte pyroptosis by increasing reactive oxygen species content and activating the NLRP3 inflammasome. Cardiovasc Diagn Ther. 2024;14:84–100. 38434559 10.21037/cdt-23-142 PMC10904297

[313] Cappetta D, De Angelis A, Sapio L, Prezioso L, Illiano M, Quaini F, et al. Oxidative stress and cellular response to doxorubicin: a common factor in the complex milieu of anthracycline cardiotoxicity. Oxid Med Cell Longev. 2017;2017:1521020. 29181122 10.1155/2017/1521020 PMC5664340

[314] Zhao QD, Viswanadhapalli S, Williams P, Shi Q, Tan C, Yi X, et al. NADPH oxidase 4 induces cardiac fibrosis and hypertrophy through activating Akt/mTOR and NFκB signaling pathways. Circulation. 2015;131:643–55. 25589557 10.1161/CIRCULATIONAHA.114.011079 PMC4568756

[315] Zhang D, Li Y, Wang W, Lang X, Zhang Y, Zhao Q, et al. NOX1 promotes myocardial fibrosis and cardiac dysfunction by activating the TLR2/NF-κB pathway in diabetic cardiomyopathy. Front Pharmacol. 2022;13:928762. 36225554 10.3389/fphar.2022.928762 PMC9549956

[316] Jiang Z, Wu L, van der Leeden B, van Rossum AC, Niessen HW, Krijnen PA. NOX2 and NOX5 are increased in cardiac microvascular endothelium of deceased COVID-19 patients. Int J Cardiol. 2023;370:454–62. 36332749 10.1016/j.ijcard.2022.10.172 PMC9625847

[317] Herranz-Iturbide M, Penuelas-Haro I, Espinosa-Sotelo R, Bertran E, Fabregat I. The TGF-β/NADPH oxidases axis in the regulation of liver cell biology in health and disease. Cells. 2021;10:2312. 34571961 10.3390/cells10092312 PMC8470857

[318] Fang FC. Antimicrobial actions of reactive oxygen species. MBio. 2011;2:00141–11. 10.1128/mbio. PMC3171981 21896680

[319] Greatorex S, Kaur S, Xirouchaki CE, Goh PK, Wiede F, Genders AJ, et al. Mitochondria-and NOX4-dependent antioxidant defense mitigates progression to nonalcoholic steatohepatitis in obesity. J Clin Investig. 2024;134:e162533. 10.1172/JCI162533 PMC10849767 38060313

[320] Matsumoto M, Zhang J, Zhang X, Liu J, Jiang JX, Yamaguchi K, et al. The NOX1 isoform of NADPH oxidase is involved in dysfunction of liver sinusoids in nonalcoholic fatty liver disease. Free Radic Biol Med. 2018;115:412–20. 29274380 10.1016/j.freeradbiomed.2017.12.019 PMC5969997

[321] Stanton MC, Chen SC, Jackson JV, Rojas-Triana A, Kinsley D, Cui L, et al. Inflammatory Signals shift from adipose to liver during high fat feeding and influence the development of steatohepatitis in mice. J Inflamm. 2011;8:8. 10.1186/1476-9255-8-8 PMC3070617 21410952

[322] Lee H, Jose PA. Coordinated contribution of NADPH oxidase-and mitochondria-derived reactive oxygen species in metabolic syndrome and its implication in renal dysfunction. Front Pharmacol. 2021;12:670076. 34017260 10.3389/fphar.2021.670076 PMC8129499

[323] Gabbia D, Cannella L, De Martin S. The role of oxidative stress in NAFLD–NASH–HCC transition—focus on NADPH oxidases. Biomedicines. 2021;9:687. 34204571 10.3390/biomedicines9060687 PMC8235710

[324] Larion S, Padgett CA, Mintz JD, Thompson JA, Butcher JT, Belin de Chantemèle EJ, et al. NADPH oxidase 1 promotes hepatic steatosis in obese mice and is abrogated by augmented skeletal muscle mass. Am J Physiol Gastrointest Liver Physiol. 2024;326:G264–73. 38258487 10.1152/ajpgi.00153.2023 PMC11211036

[325] Loffredo L, Del Ben M, Perri L, Carnevale R, Nocella C, Catasca E, et al. Effects of dark chocolate on NOX-2-generated oxidative stress in patients with nonalcoholic steatohepatitis. Aliment Pharmacol Ther. 2016;44:279–86. 27265388 10.1111/apt.13687

[326] Loffredo L, Zicari AM, Perri L, Carnevale R, Nocella C, Angelico F, et al. Does Nox2 overactivate in children with nonalcoholic fatty liver disease? Antioxid Redox Signal. 2019;30:1325–30. 30019598 10.1089/ars.2018.7596

[327] Del Ben M, Polimeni L, Carnevale R, Bartimoccia S, Nocella C, Baratta F, et al. NOX2-generated oxidative stress is associated with severity of ultrasound liver steatosis in patients with nonalcoholic fatty liver disease. BMC Gastroenterol. 2014;14:81. 24758604 10.1186/1471-230X-14-81 PMC4014405

[328] Nascè A, Gariani K, Jornayvaz FR, Szanto I. NADPH oxidases connecting fatty liver disease, insulin resistance and type 2 diabetes: current knowledge and therapeutic outlook. Antioxidants. 2022;11:1131. 35740032 10.3390/antiox11061131 PMC9219746

[329] McMahan RH, Hulsebus HJ, Najarro KM, Giesy LE, Frank DN, Kovacs EJ. Changes in gut microbiome correlate with intestinal barrier dysfunction and inflammation following a 3-day ethanol exposure in aged mice. Alcohol. 2023;107:136–43. 36150609 10.1016/j.alcohol.2022.08.011 PMC10228642

[330] Chen L, Chu H, Hu L, Li Z, Yang L, Hou X. The role of NADPH oxidase 1 in alcohol-induced oxidative stress injury of intestinal epithelial cells. Cell Biol Toxicol. 2023;39:2345–64. 35639301 10.1007/s10565-022-09725-1 PMC10547661

[331] Yamashina S, Takei Y, Ikejima K, Enomoto N, Kitamura T, Sato N. Ethanol-induced sensitization to endotoxin in Kupffer cells is dependent upon oxidative stress. Alcohol Clin Exp Res. 2005;29:246s–50s. 16385231 10.1097/01.alc.0000191128.54871.40

[332] Zhou M, Zhao X, Liao L, Deng Y, Liu M, Wang J, et al. Forsythiaside A regulates activation of hepatic stellate cells by inhibiting NOX4-dependent ROS. Oxid Med Cell Longev. 2022;2022:9938392. 35035671 10.1155/2022/9938392 PMC8754607

[333] Boudreau HE, Emerson SU, Korzeniowska A, Jendrysik MA, Leto TL. Hepatitis C virus (HCV) proteins induce NADPH oxidase 4 expression in a transforming growth factor beta-dependent manner: a new contributor to HCV-induced oxidative stress. J Virol. 2009;83:12934–46. 19812163 10.1128/JVI.01059-09 PMC2786832

[334] Sancho P, Mainez J, Crosas-Molist E, Roncero C, Fernandez-Rodriguez CM, Pinedo F, et al. NADPH oxidase NOX4 mediates stellate cell activation and hepatocyte cell death during liver fibrosis development. 2012;7:e45285. 10.1371/journal.pone.0045285 PMC3458844 23049784

[335] Lv F, Li N, Kong M, Wu J, Fan Z, Miao D, et al. CDKN2a/p16 antagonizes hepatic stellate cell activation and liver fibrosis by modulating ROS levels. Front Cell Dev Biol. 2020;8:176. 32266258 10.3389/fcell.2020.00176 PMC7105638

[336] Lan T, Kisseleva T, Brenner DA. Deficiency of NOX1 or NOX4 prevents liver inflammation and fibrosis in mice through inhibition of hepatic stellate cell activation. PLoS ONE. 2015;10:e0129743. 26222337 10.1371/journal.pone.0129743 PMC4519306

[337] Jiang JX, Chen X, Serizawa N, Szyndralewiez C, Page P, Schröder K, et al. Liver fibrosis and hepatocyte apoptosis are attenuated by GKT137831, a novel NOX4/NOX1 inhibitor in vivo. Free Radic Biol Med. 2012;53:289–96. 22618020 10.1016/j.freeradbiomed.2012.05.007 PMC3392471

[338] Cui W, Matsuno K, Iwata K, Ibi M, Matsumoto M, Zhang J, et al. NOX1/nicotinamide adenine dinucleotide phosphate, reduced form (NADPH) oxidase promotes proliferation of stellate cells and aggravates liver fibrosis induced by bile duct ligation. Hepatology. 2011;54:949–58. 21618578 10.1002/hep.24465

[339] Jiang JX, Venugopal S, Serizawa N, Chen X, Scott F, Li Y, et al. Reduced nicotinamide adenine dinucleotide phosphate oxidase 2 plays a key role in stellate cell activation and liver fibrogenesis in vivo. Gastroenterology. 2010;139:1375–84. 20685364 10.1053/j.gastro.2010.05.074 PMC2949521

[340] Nie Y, Liu Q, Zhang W, Wan Y, Huang C, Zhu X. Ursolic acid reverses liver fibrosis by inhibiting NOX4/NLRP3 inflammasome pathways and bacterial dysbiosis. Gut Microbes. 2021;13:1972746. 34530693 10.1080/19490976.2021.1972746 PMC8451456

[341] Nishio T, Hu R, Koyama Y, Liang S, Rosenthal SB, Yamamoto G, et al. Activated hepatic stellate cells and portal fibroblasts contribute to cholestatic liver fibrosis in MDR2 knockout mice. J Hepatol. 2019;71:573–85. 31071368 10.1016/j.jhep.2019.04.012

[342] Kahveci AS, Barnatan TT, Kahveci A, Adrian AE, Arroyo J, Eirin A, et al. Oxidative stress and mitochondrial abnormalities contribute to decreased endothelial nitric oxide synthase expression and renal disease progression in early experimental polycystic kidney disease. Int J Mol Sci. 2020;21:1994. 32183375 10.3390/ijms21061994 PMC7139316

[343] Jha JC, Dai A, Garzarella J, Charlton A, Urner S, Østergaard JA, et al. Independent of renox, NOX5 promotes renal inflammation and fibrosis in diabetes by activating ROS-sensitive pathways. Diabetes. 2022;71:1282–98. 35275988 10.2337/db21-1079

[344] Jandeleit-Dahm KA, Kankanamalage HR, Dai A, Meister J, Lopez-Trevino S, Cooper ME, et al. Endothelial NOX5 obliterates the reno-protective effect of Nox4 deletion by promoting renal fibrosis via activation of EMT and ROS-sensitive pathways in diabetes. Antioxidants. 2024;13:396. 38671844 10.3390/antiox13040396 PMC11047703

[345] Thallas-Bonke V, Tan SM, Lindblom RS, Snelson M, Granata C, Jha JC, et al. Targeted deletion of nicotinamide adenine dinucleotide phosphate oxidase 4 from proximal tubules is dispensable for diabetic kidney disease development. Nephrol Dial Transplant. 2021;36:988–97. 33367789 10.1093/ndt/gfaa376

[346] Shi Q, Lee D-Y, Féliers D, Abboud HE, Bhat MA, Gorin Y. Interplay between RNA-binding protein HuR and Nox4 as a novel therapeutic target in diabetic kidney disease. Mol Metab. 2020;36:100968. 32240965 10.1016/j.molmet.2020.02.011 PMC7115155

[347] Feng X, Wang S, Sun Z, Dong H, Yu H, Huang M, et al. Ferroptosis enhanced diabetic renal tubular injury via HIF-1α/HO-1 pathway in db/db mice. Front Endocrinol. 2021;12:626390. 10.3389/fendo.2021.626390 PMC7930496 33679620

[348] Yang K-J, Choi WJ, Chang Y-K, Park CW, Kim SY, Hong YA. Inhibition of xanthine oxidase protects against diabetic kidney disease through the amelioration of oxidative stress via VEGF/VEGFR axis and NOX-FoxO3a-eNOS signaling pathway. Int J Mol Sci. 2023;24:3807. 36835220 10.3390/ijms24043807 PMC9961241

[349] Gewin L, Zent R. How does TGF-β mediate tubulointerstitial fibrosis? Semin Nephrol. 2012;32:228–35. 10.1016/j.semnephrol.2012.04.001 PMC4948283 22835453

[350] Zhang H, Lai F, Cheng X, Wang Y. Involvement of NADPH oxidases in the Na/K‑ATPase/Src/ROS oxidant amplification loop in renal fibrosis. Mol Med Rep. 2023;28:161. 37417374 10.3892/mmr.2023.13048 PMC10407618

[351] Nakano T, Watanabe H, Imafuku T, Tokumaru K, Fujita I, Arimura N, et al. Indoxyl sulfate contributes to mTORC1-induced renal fibrosis via the OAT/NADPH oxidase/ROS pathway. Toxins. 2021;13:909. 34941746 10.3390/toxins13120909 PMC8706756

[352] Xia Z, Wei Z, Li X, Liu Y, Gu X, Huang S, et al. C/EBPα aggravates renal fibrosis in CKD through the NOX4-ROS-apoptosis pathway in tubular epithelial cells. Biochim Biophys Acta Mol Basis Dis. 2024;1870:167039. 38281712 10.1016/j.bbadis.2024.167039

[353] Yoo J-Y, Cha DR, Kim B, An EJ, Lee SR, Cha JJ, et al. LPS-induced acute kidney injury is mediated by Nox4-SH3YL1. Cell Rep. 2020;33:108245. 10.1016/j.celrep.2020.108245 33086058

[354] Chiang C-H, Chen C, Fang S-Y, Lin S-C, Chen J-W, Chang T-T. Xanthine oxidase/NADPH oxidase inhibition by hydralazine attenuates acute kidney injury and prevents the transition of acute kidney injury to chronic kidney disease. Life Sci. 2023;327:121863. 37331504 10.1016/j.lfs.2023.121863

[355] Zheng Z, Xu K, Li C, Qi C, Fang Y, Zhu N, et al. NLRP3 associated with chronic kidney disease progression after ischemia/reperfusion-induced acute kidney injury. Cell Death Discov. 2021;7:324. 34716316 10.1038/s41420-021-00719-2 PMC8556399

[356] Su J-Q, Wu X-Q, Wang Q, Xie B-Y, Xiao C-Y, Su H-Y, et al. The microbial metabolite trimethylamine N-oxide and the kidney diseases. Front Cell Infect Microbiol. 2025;15:1488264. 40134790 10.3389/fcimb.2025.1488264 PMC11933022

[357] Lee J, Lee J, Kim K, Lee J, Jung Y, Hyeon JS, et al. Antibiotic-induced intestinal microbiota depletion can attenuate the acute kidney injury to chronic kidney disease transition via NADPH oxidase 2 and trimethylamine-N-oxide inhibition. Kidney Int. 2024;105:1239–53. 38431216 10.1016/j.kint.2024.01.040

[358] Walton SD, Dasinger JH, Burns EC, Cherian-Shaw M, Abais-Battad JM, Mattson DL. Functional NADPH oxidase 2 in T cells amplifies salt-sensitive hypertension and associated renal damage. Am J Physiol Ren Physiol. 2023;325:F214–23. 10.1152/ajprenal.00014.2023 PMC10396224 37318993

[359] Toba H, Jin D, Takai S. Early temporal suppression of SPARC inhibits oxidative stress, inflammation, and fibrosis in the chronic phase after renal ischemia/reperfusion. J Pharmacol Sci. 2025;159:57–63. 10.1016/j.jphs.2025.07.003 40866017

[360] Walton SD, Burns-Ray EC, Dasinger JH, Hasan S, Baldwin KE, Cherian-Shaw M, et al. Exacerbation of Dahl SS hypertension and renal damage by NOX2 in CD4+ T cells. Hypertension. 2025;82:2098–111. 41058567 10.1161/HYPERTENSIONAHA.125.25528 PMC12673866

[361] Deliyanti D, Alrashdi SF, Touyz RM, Kennedy CR, Jha JC, Cooper ME, et al. Nox (NADPH oxidase) 1, Nox4, and Nox5 promote vascular permeability and neovascularization in retinopathy. Hypertension. 2020;75:1091–101. 32114846 10.1161/HYPERTENSIONAHA.119.14100

[362] Takiguchi K, Shimizu H, Shoda K, Shiraishi K, Furuya S, Hosomura N, et al. The expression and role of NADPH Oxidase 2 in colon cancer. Anticancer Res. 2023;43:2601–8. 37247898 10.21873/anticanres.16427

[363] Banskota S, Regmi SC, Kim JA. NOX1 to NOX2 switch deactivates AMPK and induces invasive phenotype in colon cancer cells through overexpression of MMP-7. Mol Cancer. 2015;14:123. 26116564 10.1186/s12943-015-0379-0 PMC4482031

[364] Grauers Wiktorin H, Aydin E, Kiffin R, Vilhav C, Bourghardt Fagman J, Kaya M, et al. Impact of surgery-induced myeloid-derived suppressor cells and the NOX2/ROS axis on postoperative survival in human pancreatic cancer. Cancer Res Commun. 2024;4:1135–49. 38598844 10.1158/2767-9764.CRC-23-0447 PMC11044860

[365] Zhang X, Jin B, Huang C. The PI3K/Akt pathway and its downstream transcription factors as targets for chemoprevention. Curr Cancer Drug Targets. 2007;7:305–16. 17979625 10.2174/156800907780809741

[366] Shiau D-J, Kuo W-T, Davuluri GVN, Shieh C-C, Tsai P-J, Chen C-C, et al. Hepatocellular carcinoma-derived high mobility group box 1 triggers M2 macrophage polarization via a TLR2/NOX2/autophagy axis. Sci Rep. 2020;10:13582. 32788720 10.1038/s41598-020-70137-4 PMC7423894

[367] Deng J, Guo Y, Hu X, Du J, Gu J, Kong L, et al. High glucose promotes pancreatic ductal adenocarcinoma gemcitabine resistance and invasion through modulating ROS/MMP-3 signaling pathway. Oxid Med Cell Longev. 2022;2022:3243647. 36211828 10.1155/2022/3243647 PMC9536998

[368] Hervieu M, Legrand AJ, Floquet E, Idziorek T, Spriet C, Monté D, et al. PARP-1 inhibition increases oxidative stress in Ets-1-expressing MDA-MB-231 breast cancer cells. Cancer Rep. 2025;8:e70119. 10.1002/cnr2.70119 PMC11706972 39778077

[369] Ijurko C, Romo-González M, García-Calvo C, Sardina JL, Sánchez-Bernal C, Sánchez-Yagüe J, et al. NOX2 control over energy metabolism plays a role in acute myeloid leukemia prognosis and survival. Free Radic Biol Med. 2023;209:18–28. 37806599 10.1016/j.freeradbiomed.2023.10.013

[370] Liu Y, Wu C, Wang Q, Zhao X, Jiang X. NOX4 as an emerging prognostic and immunological biomarker across pancancer analyses. Sci Rep. 2025;15:18812. 40442199 10.1038/s41598-025-03499-2 PMC12123023

[371] Liu W-J, Wang L, Zhou F-M, Liu S-W, Wang W, et al. Elevated NOX4 promotes tumorigenesis and acquired EGFR-TKIs resistance by enhancing IL-8/PD-L1 signaling in NSCLC. Drug Resist Update. 2023;70:100987. 10.1016/j.drup.2023.100987 37392558

[372] Liao YC, Wu SY, Huang YF, Lo PC, Chan TY, Chen CA, et al. NOX2-deficient neutrophils facilitate joint inflammation through higher pro-inflammatory and weakened immune checkpoint activities. Front Immunol. 2021;12:743030. 34557202 10.3389/fimmu.2021.743030 PMC8452958

[373] Grauers Wiktorin H, Aydin E, Hellstrand K, Martner A. NOX2-derived reactive oxygen species in cancer. Oxid Med Cell Longev. 2020;2020:7095902. 33312338 10.1155/2020/7095902 PMC7721506

[374] Chen C, Dong Q, Wang H, Dong S, Wang S, Lin W, et al. The association between NADPH oxidase (NOX) polymorphisms with immunohistochemistry and survival in diffuse large B-cell lymphoma patients. Ann Hematol. 2025;104:407–20. 39774928 10.1007/s00277-024-06144-6

[375] Liu D, Wu N, Sun H, Dong M, Guo T, Chi P, et al. ABCG2 and NCF4 polymorphisms are associated with clinical outcomes in diffuse large B-cell lymphoma patients treated with R-CHOP. Oncotarget. 2017;8:58292. 28938556 10.18632/oncotarget.16869 PMC5601652

[376] Zhong Y-L, Wang B-B, Chu K-F, Wang A-J, Zhao T, Feng J-J. High-entropy intermetallic alloy/carbon nanoflower cascade nanozyme for multimodal synergistic cancer therapy. J Colloid Interface Sci. 2025:138753. 10.1016/j.jcis.2025.138753 40845442

[377] Wang Z, Jing X, Zhang D, Yu Z, Zhang H, Liu D, et al. Dual-mode hybrid discharge plasma-activated injectable hydrosol for enhanced immunotherapeutic cancer therapy. Adv Healthcare Mater. 2026;15:e02779. 10.1002/adhm.202502779 40914824

[378] Fenniche S, Oukabli M, Oubaddou Y, Chahdi H, Damiri A, Alghuzlan A, et al. A comparative analysis of NOX4 protein expression in malignant and nonmalignant thyroid tumors. Curr Issues Mol Biol. 2023;45:5811–23. 37504283 10.3390/cimb45070367 PMC10378117

[379] Ijurko C, Romo-González M, Prieto-Bermejo R, Díez-Campelo M, Vidriales M-B, Muntión S, et al. Targeting NOX2 and glycolytic metabolism as a therapeutic strategy in acute myeloid leukemia. Biomark Res. 2024;12:128. 39468687 10.1186/s40364-024-00674-x PMC11520369

[380] Peñuelas-Haro I, Espinosa-Sotelo R, Crosas-Molist E, Herranz-Itúrbide M, Caballero-Díaz D, Alay A, et al. The NADPH oxidase NOX4 regulates redox and metabolic homeostasis preventing HCC progression. Hepatology. 2023;78:416–33. 35920301 10.1002/hep.32702 PMC10344438

[381] Kwon J, Wang A, Burke DJ, Boudreau HE, Lekstrom KJ, Korzeniowska A, et al. Peroxiredoxin 6 (Prdx6) supports NADPH oxidase1 (Nox1)-based superoxide generation and cell migration. Free Radical Biology and Medicine. 2016;1;96:99–115. 10.1016/j.freeradbiomed.2016.04.009 PMC4929831 27094494

[382] Lagal DJ, López-Grueso MJ, Pedrajas JR, Leto TL, Bárcena JA, Requejo-Aguilar R, et al. Loss of PRDX6 aborts proliferative and migratory signaling in hepatocarcinoma cell lines. Antioxidants. 2023;12:1153. 10.3390/antiox12061153 PMC10294896 37371884

[383] Arevalo JA, Vázquez-Medina JP. The Role of Peroxiredoxin 6 in Cell Signaling. Antioxidants (Basel). 2018;7:172. 10.3390/antiox7120172 PMC6316032 30477202

[384] Valente AJ, El Jamali A, Epperson TK, Gamez MJ, Pearson DW, Clark RA. NOX1 NADPH oxidase regulation by the NOXA1 SH3 domain. Free Radical Biology and Medicine. 2007;43:384–96. 10.1016/j.freeradbiomed.2007.04.022 17602954

[385] Park JH, Yoon SG, Ghee JY, Yoo JA, Cha JJ, Kang YS, et al. Pan-Nox inhibitor treatment improves renal function in aging murine diabetic kidneys. Kidney Res Clin Pract. 2023;43:763. 37559225 10.23876/j.krcp.23.004 PMC11615449

[386] Chauhan W, Sudharshan S, Ferdowsi S, Sohel A, Zennadi R. Red blood cell Rpl13a small noncoding nucleolar RNAs guides 2ʹ-O-methylation on peroxidasin messenger RNA promoting venous thrombosis in aging. J Thromb Hemost. 2025;S1538-7836:00135-7. 10.1016/j.jtha.2025.02.036 PMC12353407 40058703

[387] Chauhan W, Ferdowsi S, Sudharshan S, Zennadi R. Rpl13a snoRNAs-regulated NADPH oxidase 1-dependent ROS generation: a novel RBC pathway mediating complement C3a deposition and triggering thrombosis in aging and venous blood clotting disorders. Free Radic Biol Med. 2025;230:138–50. 39938620 10.1016/j.freeradbiomed.2025.02.008 PMC11936428

[388] Shi X, Li P, Herb M, Liu H, Wang M, Wang X, et al. Pathological high intraocular pressure induces glial cell reactive proliferation contributing to neuroinflammation of the blood‒retinal barrier via the NOX2/ET-1 axis-controlled ERK1/2 pathway. J Neuroinflammation. 2024;21:105. 38649885 10.1186/s12974-024-03075-x PMC11034147

[389] Fan LM, Geng L, Cahill-Smith S, Liu F, Douglas G, Mckenzie C-A, et al. Nox2 contributes to age-related oxidative damage to neurons and the cerebral vasculature. J Clin Investig. 2019;129:3374–86. 31329158 10.1172/JCI125173 PMC6668817

[390] Bugara K, Pacwa A, Smedowski A. Molecular pathways in experimental glaucoma models. Front Neurosci. 2024;18:1363170. 38562304 10.3389/fnins.2024.1363170 PMC10982327

[391] Lee H-Y, Zeeshan HMA, Kim H-R, Chae H-J. Corrigendum to “Nox 4 regulates the eNOS uncoupling process in aging endothelial cells”[Free Rad. Biol. Med. 113 (2017) 26–35]. Free Radic Biol Med. 2025;232:450–1. 40089445 10.1016/j.freeradbiomed.2025.03.001

[392] Shin KO, Uchida Y, Park K. Diesel particulate extract accelerates premature skin aging in human fibroblasts via ceramide-1-phosphate-mediated signaling pathway. Int J Mol Sci. 2022;23:2691. 10.3390/ijms23052691 PMC8910364 35269833

[393] Cortés M, Olate P, Rodriguez R, Diaz R, Martínez A, Hernández G, et al. Human microbiome as an immunoregulatory axis: mechanisms, dysbiosis, and therapeutic modulation. Microorganisms. 2025;13:2147. 41011478 10.3390/microorganisms13092147 PMC12472381

[394] van der Post S, Birchenough GM, Held JM. NOX1-dependent redox signaling potentiates colonic stem cell proliferation to adapt to the intestinal microbiota by linking EGFR and TLR activation. Cell Rep. 2021;35:108949. 10.1016/j.celrep.2021.108949 PMC10327654 33826887

[395] Sharma A, Singh S, Ahmad S, Gulzar F, Schertzer JD, Tamrakar AK. NOD1 activation induces oxidative stress via NOX1/4 in adipocytes. Free Radic Biol Med. 2021;162:118–28. 33279617 10.1016/j.freeradbiomed.2020.11.036

[396] Pu Q, Lin P, Gao P, Wang Z, Guo K, Qin S, et al. Gut microbiota regulate gut–lung axis inflammatory responses by mediating ILC2 compartmental migration. J Immunol. 2021;207:257–67. 34135060 10.4049/jimmunol.2001304 PMC8674377

[397] Lou X, Xue J, Shao R, Yang Y, Ning D, Mo C, et al. Fecal microbiota transplantation and short-chain fatty acids reduce sepsis mortality by remodeling antibiotic-induced gut microbiota disturbances. Front Immunol. 2023;13:1063543. 36713461 10.3389/fimmu.2022.1063543 PMC9874322

[398] Budden KF, Gellatly SL, Wood DL, Cooper MA, Morrison M, Hugenholtz P, et al. Emerging pathogenic links between microbiota and the gut–lung axis. Nat Rev Microbiol. 2017;15:55–63. 27694885 10.1038/nrmicro.2016.142

[399] Gurczynski SJ, Lipinski JH, Strauss J, Alam S, Huffnagle GB, Ranjan P, et al. Horizontal transmission of gut microbiota attenuates mortality in lung fibrosis. JCI insight. 2023;9:e164572. 38015634 10.1172/jci.insight.164572 PMC10911107

[400] Rutkowski N, Yang B, Gray-Gaillard E, Ruta A, Mejías JC, Patatanian M, et al. Antibiotic-induced microbiota depletion impairs the pro-regenerative response to a biological scaffold. Proceedings of the National Academy of Sciences. 2025;122:e2510841122. 10.1073/pnas.2510841122 PMC12772165 41428865

[401] Miller BM, Liou MJ, Zhang LF, Nguyen H, Litvak Y, Schorr E-M, et al. Anaerobic respiration of NOX1-derived hydrogen peroxide licenses bacterial growth at the colonic surface. Cell Host Microbe. 2020;28:789–97.e5. 33301718 10.1016/j.chom.2020.10.009 PMC7758056

[402] Leoni G, Alam A, Neumann PA, Lambeth JD, Cheng G, McCoy J, et al. Annexin A1, formyl peptide receptor, and NOX1 orchestrate epithelial repair. J Clin Investig. 2013;123:443–54. 23241962 10.1172/JCI65831 PMC3533303

[403] Matziouridou C, Rocha S, Haabeth O, Rudi K, Carlsen H, Kielland A. iNOS-and NOX1-dependent ROS production maintains bacterial homeostasis in the ileum of mice. Mucosal Immunol. 2018;11:774–84. 29210363 10.1038/mi.2017.106

[404] Kohn DB, Booth C, Kang EM, Pai S-Y, Shaw KL, Santilli G, et al. Lentiviral gene therapy for X-linked chronic granulomatous disease. Nat Med. 2020;26:200–6. 31988463 10.1038/s41591-019-0735-5 PMC7115833

[405] Sobrino S, Magnani A, Semeraro M, Martignetti L, Cortal A, Denis A, et al. Severe hematopoietic stem cell inflammation compromises chronic granulomatous disease gene therapy. Cell Rep Med. 2023;4:100919. 10.1016/j.xcrm.2023.100919 PMC9975109 36706754

[406] Bzhilyanskaya V, Ma L, Liu S, Fox LR, Whittaker MN, Meis RJ, et al. High-fidelity PAMless base editing of hematopoietic stem cells to treat chronic granulomatous disease. Sci Transl Med. 2024;16:eadj6779. 39413163 10.1126/scitranslmed.adj6779 PMC11753194

[407] Buggisch M, Ateghang B, Ruhe C, Strobel C, Lange S, Wartenberg M, et al. Stimulation of ES-cell-derived cardiomyogenesis and neonatal cardiac cell proliferation by reactive oxygen species and NADPH oxidase. J Cell Sci. 2007;120:885–94. 17298980 10.1242/jcs.03386

[408] Lee JE, Cho KE, Lee KE, Kim J, Bae YS. Nox4-mediated cell signaling regulates differentiation and survival of neural crest stem cells. Mol Cells. 2014;37:907–11. 25410908 10.14348/molcells.2014.0244 PMC4275708

[409] Duelen R, Costamagna D, Gilbert G, De Waele L, Goemans N, Desloovere K, et al. Human iPSC model reveals a central role for NOX4 and oxidative stress in Duchenne cardiomyopathy. Stem Cell Rep. 2022;17:352–68. 10.1016/j.stemcr.2021.12.019 PMC8828550 35090586

[410] Li J, Stouffs M, Serrander L, Banfi B, Bettiol E, Charnay Y, et al. The NADPH oxidase NOX4 drives cardiac differentiation: Role in regulating cardiac transcription factors and MAP kinase activation. Mol Biol Cell. 2006;17:3978–88. 16775014 10.1091/mbc.E05-06-0532 PMC1556380

[411] Zheng H, Xu N, Zhang Z, Wang F, Xiao J, Ji X. Setanaxib (GKT137831) ameliorates doxorubicin-induced cardiotoxicity by inhibiting the NOX1/NOX4/reactive oxygen species/MAPK pathway. Front Pharmacol. 2022;13:823975. 35444554 10.3389/fphar.2022.823975 PMC9014097

[412] Chen JR, Lazarenko OP, Blackburn ML, Chen JF, Randolph CE, Zabaleta J, et al. Nox4 expression in osteo-progenitors controls bone development in mice during early life. Commun Biol. 2022;5:583. 35701603 10.1038/s42003-022-03544-0 PMC9198054

[413] Cao Z, Liu G, Zhang H, Wang M, Xu Y. Nox4 promotes osteoblast differentiation through TGF-beta signaling pathway. Free Radic Biol Med. 2022;193:595–609. 36372285 10.1016/j.freeradbiomed.2022.11.016

[414] Vandergriff A, Huang K, Shen D, Hu S, Hensley MT, Caranasos TG, et al. Targeting regenerative exosomes to myocardial infarction using cardiac homing peptide. Theranostics. 2018;8:1869–78. 29556361 10.7150/thno.20524 PMC5858505

[415] Zhong Z, Tian Y, Luo X, Zou J, Wu L, Tian J. Extracellular vesicles derived from human umbilical cord mesenchymal stem cells protect against DOX-induced heart failure through the miR-100-5p/NOX4 pathway. Front Bioeng Biotechnol. 2021;9:703241. 34513812 10.3389/fbioe.2021.703241 PMC8424184

[416] Choi DH, Choi IA, Lee J. Role of NADPH oxidases in stroke recovery. Antioxidants. 2024;13:1065. 10.3390/antiox13091065 PMC11428334 39334724

[417] Khayrullina G, Bermudez S, Byrnes KR. Inhibition of NOX2 reduces locomotor impairment, inflammation, and oxidative stress after spinal cord injury. J Neuroinflammation. 2015;12:172. 26377802 10.1186/s12974-015-0391-8 PMC4574142

[418] To EE, Luong R, Diao J, O’Leary JJ, Brooks DA, Vlahos R, et al. Novel endosomal NOX2 oxidase inhibitor ameliorates pandemic influenza A virus-induced lung inflammation in mice. Respirology. 2019;24:1011–7. 30884042 10.1111/resp.13524 PMC6972593

[419] Khayrullina G, Bermudez S, Hopkins D, Yauger Y, Byrnes KR. Differential effects of NOX2 and NOX4 inhibition after rodent spinal cord injury. PLoS ONE. 2023;18:e0281045. 36897852 10.1371/journal.pone.0281045 PMC10004500

[420] To EE, Erlich JR, Liong F, Liong S, Luong R, Oseghale O, et al. Therapeutic targeting of endosome and mitochondrial reactive oxygen species protects mice from influenza virus morbidity. Front Pharmacol. 2022;13:870156. 35401240 10.3389/fphar.2022.870156 PMC8984148

[421] André-Lévigne D, Modarressi A, Pepper MS, Pittet-Cuénod B. Reactive oxygen species and NOX enzymes are emerging as key players in cutaneous wound repair. Int J Mol Sci. 2017;18:2149. 29036938 10.3390/ijms18102149 PMC5666831

[422] Blaise O, Duchesne C, Capuzzo E, Nahori M-A, Fernandes J, Connor MG, et al. Infected wound repair correlates with collagen I induction and NOX2 activation by cold atmospheric plasma. NPJ Regen Med. 2024;9:28. 39358383 10.1038/s41536-024-00372-0 PMC11447178

[423] Korkmaz, Ulrich HI, Çelik MM, Van G, Wieringen, Van WN, et al. NOX2 expression is increased in keratinocytes after burn injury. J Burn Care Res. 2020;41:427–32. 31602477 10.1093/jbcr/irz162 PMC7030073

[424] Dominik L, Ali M, Karl-Heinz K, Brigitte P-C. NADPH oxidase 4 deficiency leads to impaired wound repair and reduced dityrosine-crosslinking, but does not affect myofibroblast formation. Free Radic Biol Med. 2016;96:374–84. 27140231 10.1016/j.freeradbiomed.2016.04.194

[425] Reutens AT, Jandeleit-Dahm K, Thomas M, Salim A, De Livera AM, Bach LA, et al. A physician-initiated double-blind, randomized, placebo-controlled, phase 2 study evaluating the efficacy and safety of inhibition of NADPH oxidase with the first-in-class Nox-1/4 inhibitor, GKT137831, in adults with type 1 diabetes and persistently elevated urinary albumin excretion: Protocol and statistical considerations. Contemp Clin trials. 2020;90:105892. 31740428 10.1016/j.cct.2019.105892

[426] Invernizzi P, Carbone M, Jones D, Levy C, Little N, Wiesel P, et al. Setanaxib, a first-in-class selective NADPH oxidase 1/4 inhibitor for primary biliary cholangitis: a randomized, placebo-controlled, phase 2 trial. Liver Int. 2023;43:1507–22. 37183520 10.1111/liv.15596

[427] Sato S, Yanagihara T, Kolb MR. Therapeutic targets and early stage clinical trials for pulmonary fibrosis. Expert Opin Investig Drugs. 2019;28:19–28. 30513000 10.1080/13543784.2019.1554054

[428] Gray SP, Jha JC, Kennedy K, Van Bommel E, Chew P, Szyndralewiez C, et al. Combined NOX1/4 inhibition with GKT137831 in mice provides dose-dependent reno-and atheroprotection even in established microand macrovascular disease. Diabetologia. 2017;60:927–37. 28160092 10.1007/s00125-017-4215-5

[429] Cha JJ, Min HS, Kim KT, Kim JE, Ghee JY, Kim HW, et al. APX-115, a first-in-class pan-NADPH oxidase (Nox) inhibitor, protects db/db mice from renal injury. Lab Investig. 2017;97:419–31. 28165467 10.1038/labinvest.2017.2

[430] Lu WJ, Li JY, Chen RJ, Huang LT, Lee TY, Lin KH. VAS2870 and VAS3947 attenuate platelet activation and thrombus formation via a NOX-independent pathway downstream of PKC. Sci Rep. 2019;9:18852. 31827142 10.1038/s41598-019-55189-5 PMC6906488

[431] Gianni D, Taulet N, Zhang H, DerMardirossian C, Kister J, Martinez L, et al. A novel and specific NADPH oxidase-1 (Nox1) small-molecule inhibitor blocks the formation of functional invadopodia in human colon cancer cells. ACS Chem Biol. 2010;5:981–93. 20715845 10.1021/cb100219n PMC2955773

[432] Zavadskis S, Weidinger A, Hanetseder D, Banerjee A, Schneider C, Wolbank S, et al. Effect of diphenyleneiodonium chloride on intracellular reactive oxygen species metabolism with emphasis on NADPH oxidase and mitochondria in two therapeutically relevant human cell types. Pharmaceutics. 2020;13:10. 10.3390/pharmaceutics13010010 PMC7823933 33374729

[433] Somanna NK, Valente AJ, Krenz M, Fay WP, Delafontaine P, Chandrasekar B. The Nox1/4 dual inhibitor GKT137831 or Nox4 knockdown inhibits angiotensin-II-induced adult mouse cardiac fibroblast proliferation and migration. AT1 physically associates with Nox4. J Cell Physiol. 2016;231:1130–41. 26445208 10.1002/jcp.25210 PMC5237386

[434] Sivarupa R, Dhat R, Sitasawad SL. Nox4 inhibition with GKT137831 protects against diabetic cardiomyopathy: Insights from functional and transcriptomic analysis. Pharmacol Res Rep. 2025;4:100053.

[435] Wilkinson-Berka JL, Deliyanti D, Rana I, Miller AG, Agrotis A, Armani R, et al. NADPH oxidase, NOX1, mediates vascular injury in ischemic retinopathy. Antioxid Redox Signal. 2014;20:2726–40. 24053718 10.1089/ars.2013.5357 PMC4026404

[436] Al-Shabrawey M, Bartoli M, El-Remessy AB, Platt DH, Matragoon S, Behzadian MA, et al. Inhibition of NAD(P)H oxidase activity blocks vascular endothelial growth factor overexpression and neovascularization during ischemic retinopathy. Am J Pathol. 2005;167:599–607. 16049343 10.1016/S0002-9440(10)63001-5 PMC1603550

[437] Liao J, Lai Z, Huang G, Lin J, Huang W, Qin Y, et al. Setanaxib mitigates oxidative damage following retinal ischemia‒reperfusion via NOX1 and NOX4 inhibition in retinal ganglion cells. Biomed Pharmacother. 2024;170:116042. 38118351 10.1016/j.biopha.2023.116042

[438] Gorin Y, Cavaglieri RC, Khazim K, Lee D-Y, Bruno F, Thakur S, et al. Targeting NADPH oxidase with a novel dual Nox1/Nox4 inhibitor attenuates renal pathology in type 1 diabetes. Am J Physiol Ren Physiol. 2015;308:F1276–87. 10.1152/ajprenal.00396.2014 PMC4451325 25656366

[439] Lee ES, Kim HM, Lee SH, Ha KB, Bae YS, Lee SJ, et al. APX-115, a pan-NADPH oxidase inhibitor, protects development of diabetic nephropathy in podocyte specific NOX5 transgenic mice. Free Radic Biol Med. 2020;161:92–101. 33011273 10.1016/j.freeradbiomed.2020.09.024

[440] Kwon G, Uddin MJ, Lee G, Jiang S, Cho A, Lee JH, et al. A novel pan-Nox inhibitor, APX-115, protects kidney injury in streptozotocin-induced diabetic mice: possible role of peroxisomal and mitochondrial biogenesis. Oncotarget. 2017;8:74217. 29088780 10.18632/oncotarget.18540 PMC5650335

[441] Wingler K, Altenhoefer SA, Kleikers PW, Radermacher KA, Kleinschnitz C, Schmidt HH. VAS2870 is a pan-NADPH oxidase inhibitor. Cell Mol Life Sci. 2012;69:3159–60. 22875281 10.1007/s00018-012-1107-1 PMC11114962

[442] Reis J, Massari M, Marchese S, Ceccon M, Aalbers FS, Corana F, et al. A closer look into NADPH oxidase inhibitors: validation and insight into their mechanism of action. Redox Biol. 2020;32:101466. 32105983 10.1016/j.redox.2020.101466 PMC7042484

[443] Yang J, Li J, Wang Q, Xing Y, Tan Z, Kang Q. Novel NADPH oxidase inhibitor VAS2870 suppresses TGF‑β‑dependent epithelial‑to‑mesenchymal transition in retinal pigment epithelial cells. Int J Mol Med. 2018;42:123–30. 29620174 10.3892/ijmm.2018.3612 PMC5979836

[444] Banskota S, Wang H, Kwon YH, Gautam J, Haq S, Grondin J, et al. Inhibition of NADPH oxidase (NOX) 2 mitigates colitis in mice with impaired macrophage AMPK function. Biomedicines. 2023;11:1443. 10.3390/biomedicines11051443 PMC10216132 37239114

[445] ten Freyhaus H, Huntgeburth M, Wingler K, Schnitker J, Bäumer AT, Vantler M, et al. Novel Nox inhibitor VAS2870 attenuates PDGF-dependent smooth muscle cell chemotaxis, but not proliferation. Cardiovasc Res. 2006;71:331–41. 16545786 10.1016/j.cardiores.2006.01.022

[446] Juric M, Rawat V, Amaradhi R, Zielonka J, Ganesh T. Novel NADPH oxidase-2 inhibitors as potential anti-inflammatory and neuroprotective agents. Antioxidants. 2023;12:1660. 37759963 10.3390/antiox12091660 PMC10525516

[447] Wang H, Dawber RS, Zhang P, Walko M, Wilson AJ, Wang X. Peptide-based inhibitors of protein–protein interactions: biophysical, structural and cellular consequences of introducing a constraint. Chem Sci. 2021;12:5977–93. 33995995 10.1039/d1sc00165e PMC8098664

[448] Chen Q, Devine I, Walker S, Pham H, Ondrasik R, Patel H, et al. Nox2ds-Tat, a peptide inhibitor of NADPH oxidase, exerts cardioprotective effects by attenuating reactive oxygen species during ischemia/reperfusion injury. Am J Biomed Sci. 2016;8:208–27.

[449] Scalia E, Chirco A, Calugi L, Lenci E, Pagano PJ, Pula G, et al. Development of new peptidomimetic NADPH oxidase inhibitors with antithrombotic properties. ChemMedChem. 2024;19:e202400330. 38924475 10.1002/cmdc.202400330

[450] Treuer AV, Faúndez M, Ebensperger R, Hovelmeyer E, Vergara-Jaque A, Perera-Sardiña Y, et al. New NADPH oxidase 2 inhibitors display potent activity against oxidative stress by targeting p22phox-p47phox interactions. Antioxidants. 2023;12:1441. 37507978 10.3390/antiox12071441 PMC10376059

[451] Li J, Newburger PE, Gounis M, Dargon P, Zhang X, Messina LM. Local arterial nanoparticle delivery of siRNA for NOX2 knockdown to prevent restenosis in an atherosclerotic rat model. Gene Ther. 2010;17:1279–87. 20485380 10.1038/gt.2010.69 PMC2955163

[452] Lee YA, Shin MH. Involvement of NOX2-derived ROS in human hepatoma HepG2 cell death induced by Entamoeba histolytica. Parasites Hosts Dis. 2023;61:388. 38043534 10.3347/PHD.23094 PMC10693973

[453] Adu-Gyamfi M, Goettsch C, Kamhieh-Milz J, Chen L, Pfefferkorn AM, Hofmann A, et al. The role of NOX2-derived reactive oxygen species in the induction of endothelin-converting enzyme-1 by angiotensin II. Antioxidants. 2024;13:500. 38671948 10.3390/antiox13040500 PMC11047448

[454] Li YJ, Zhao W, Yu XJ, Li FX, Liu ZT, Li L, et al. Activation of p47phox as a mechanism of bupivacaine-induced burst production of reactive oxygen species and neural toxicity. Oxid Med Cell Longev. 2017;2017:8539026. 28751934 10.1155/2017/8539026 PMC5480047

[455] Xu F, Liu Y, Shi L, Liu W, Zhang L, Cai H, et al. NADPH oxidase p47phox siRNA attenuates adventitial fibroblasts proliferation and migration in apoE(-/-) mouse. J Transl Med. 2015;13:38. 25628043 10.1186/s12967-015-0407-2 PMC4312606

[456] Xiao W, Peng Y, Liu Y, Li Z, Li S, Zheng X. HSCARG inhibits NADPH oxidase activity through regulation of the expression of p47phox. PLoS ONE. 2013;8:e59301. 23527155 10.1371/journal.pone.0059301 PMC3602244

[457] Ni S, Li D, Wei H, Miao K-S, Zhuang C. PPARγ attenuates interleukin-1β-induced apoptosis by inhibiting NOX2/ROS/p38MAPK activation in osteoarthritis chondrocytes. Oxid Med Cell Longev. 2021;2021:5551338. 34055194 10.1155/2021/5551338 PMC8112933

[458] Wang X, Jiang M, He X, Zhang B, Peng W, Guo L. N‑acetyl cysteine inhibits the lipopolysaccharide‑induced inflammatory response in bone marrow mesenchymal stem cells by suppressing the TXNIP/NLRP3/IL‑1β signaling pathway. Mol Med Rep. 2020;22:3299–306. 32945495 10.3892/mmr.2020.11433 PMC7453581

[459] Huang W, Hutabarat RP, Chai Z, Zheng T, Zhang W, Li D. Antioxidant blueberry anthocyanins induce vasodilation via PI3K/Akt signaling pathway in high-glucose-induced human umbilical vein endothelial cells. Int J Mol Sci. 2020;21:1575. 32106617 10.3390/ijms21051575 PMC7084611

[460] Cai X, Yang C, Shao L, Zhu H, Wang Y, Huang X, et al. Targeting NOX 4 by petunidin improves anoxia/reoxygenation-induced myocardium injury. Eur J Pharmacol. 2020;888:173414. 32828742 10.1016/j.ejphar.2020.173414

[461] Demircan MB, Mgbecheta PC, Kresinsky A, Schnoeder TM, Schröder K, Heidel FH, et al. Combined activity of the redox-modulating compound setanaxib (GKT137831) with cytotoxic agents in the killing of acute myeloid leukemia cells. Antioxidants. 2022;11:513. 35326163 10.3390/antiox11030513 PMC8944474

[462] Hanley CJ, Mellone M, Ford K, Thirdborough SM, Mellows T, Frampton SJ, et al. Targeting the myofibroblastic cancer-associated fibroblast phenotype through inhibition of NOX4. J Natl Cancer Inst. 2018;110:109–20. 28922779 10.1093/jnci/djx121 PMC5903651

[463] Sampson N, Brunner E, Weber A, Puhr M, Schäfer G, Szyndralewiez C, et al. Inhibition of Nox4-dependent ROS signaling attenuates prostate fibroblast activation and abrogates stromal-mediated protumorigenic interactions. Int J Cancer. 2018;143:383–95. 29441570 10.1002/ijc.31316 PMC6067067

[464] Hollingsworth SA, Dror RO. Molecular dynamics simulation for all. Neuron. 2018;99:1129–43. 30236283 10.1016/j.neuron.2018.08.011 PMC6209097

[465] Sousa SF, Ribeiro AJ, Coimbra J, Neves R, Martins S, Moorthy N, et al. Protein‒ligand docking in the new millennium–a retrospective of 10 years in the field. Curr Med Chem. 2013;20:2296–314. 23531220 10.2174/0929867311320180002

[466] Jumper J, Evans R, Pritzel A, Green T, Figurnov M, Ronneberger O, et al. Highly accurate protein structure prediction with AlphaFold. Nature. 2021;596:583–9. 34265844 10.1038/s41586-021-03819-2 PMC8371605

[467] Liu Y, Liang S, Shi D, Zhang Y, Bai C, Ye RD. A predicted structure of NADPH Oxidase 1 identifies key components of ROS generation and strategies for inhibition. PLoS ONE. 2023;18:e0285206. 37134122 10.1371/journal.pone.0285206 PMC10155968

[468] Ochoa D, Hercules A, Carmona M, Suveges D, Gonzalez-Uriarte A, Malangone C, et al. Open Targets Platform: supporting systematic drug–target identification and prioritization. Nucleic Acids Res. 2021;49:D1302–D10. 33196847 10.1093/nar/gkaa1027 PMC7779013

[469] Xu J, Xu X, Li X, He S, Li D, Ji B. Cellular mechanics of wound formation in single cell layer under cyclic stretching. Biophys J. 2022;121:288–99. 34902328 10.1016/j.bpj.2021.12.015 PMC8790211

[470] Magnani F, Nenci S, Millana Fananas E, Ceccon M, Romero E, Fraaije MW, et al. Crystal structures and atomic model of NADPH oxidase. Proc Natl Acad Sci USA. 2017;114:6764–9. 28607049 10.1073/pnas.1702293114 PMC5495252

[471] Petit-Hartlein I, Vermot A, Thepaut M, Humm A-S, Dupeux F, Dupuy J, et al. X-ray structure and enzymatic study of a bacterial NADPH oxidase highlight the activation mechanism of eukaryotic NOX. eLife. 2024;13:RP93759. 38640072 10.7554/eLife.93759 PMC11031084

[472] Sumimoto H. Structure, regulation and evolution of Nox-family NADPH oxidases that produce reactive oxygen species. FEBS J. 2008;275:3249–77. 18513324 10.1111/j.1742-4658.2008.06488.x

[473] Wu T, Jiang W. Computational-aided drug design strategies for drug discovery and development against oral diseases. Front Pharmacol. 2025;16:1678652. 41282627 10.3389/fphar.2025.1678652 PMC12634332

[474] Genheden S, Ryde U. The MM/PBSA and MM/GBSA methods to estimate ligand-binding affinities. Expert Opin Drug Discov. 2015;10:449–61. 25835573 10.1517/17460441.2015.1032936 PMC4487606

[475] Laleu B, Gaggini F, Orchard M, Fioraso-Cartier L, Cagnon L, Houngninou-Molango S, et al. First in class, potent, and orally bioavailable NADPH oxidase isoform 4 (Nox4) inhibitors for the treatment of idiopathic pulmonary fibrosis. J Med Chem. 2010;53:7715–30. 20942471 10.1021/jm100773e

[476] Bonnefoy N, Fox TD. Directed alteration of *Saccharomyces cerevisiae* mitochondrial DNA by biolistic transformation and homologous recombination. In: Mitochondria: Practical Protocols. Springer; 2007:372.:153–66. Totowa, NJ: Humana Press. 10.1007/978-1-59745-365-3_11 PMC2771616 18314724

[477] Castellani RJ, Perry G. The complexities of the pathology–pathogenesis relationship in Alzheimer disease. Biochem Pharmacol. 2014;88:671–6. 24447936 10.1016/j.bcp.2014.01.009

[478] Sun Q-A, Hess DT, Wang B, Miyagi M, Stamler JS. Off-target thiol alkylation by the NADPH oxidase inhibitor 3-benzyl-7-(2-benzoxazolyl) thio-1, 2,3-triazolo [4,5-d] pyrimidine (VAS2870). Free Radic Biol Med. 2012;52:1897–902. 22406319 10.1016/j.freeradbiomed.2012.02.046 PMC3354571

[479] De Vivo M, Masetti M, Bottegoni G, Cavalli A. Role of molecular dynamics and related methods in drug discovery. J Med Chem. 2016;59:4035–61. 26807648 10.1021/acs.jmedchem.5b01684

[480] Ago T, Kuribayashi F, Hiroaki H, Takeya R, Ito T, Kohda D, et al. Phosphorylation of p47 phox directs phox homology domain from SH3 domain toward phosphoinositides, leading to phagocyte NADPH oxidase activation. Proc Natl Acad Sci USA. 2003;100:4474–9. 12672956 10.1073/pnas.0735712100 PMC153580

[481] Meijles DN, Fan LM, Howlin BJ, Li J-M. Molecular insights of p47phox phosphorylation dynamics in the regulation of NADPH oxidase activation and superoxide production. J Biol Chem. 2014;289:22759–70. 24970888 10.1074/jbc.M114.561159 PMC4132782

[482] Streeter J, Schickling BM, Jiang S, Stanic B, Thiel WH, Gakhar L, et al. Phosphorylation of Nox1 regulates association with NoxA1 activation domain. Circ Res. 2014;115:911–8. 25228390 10.1161/CIRCRESAHA.115.304267 PMC5025877

[483] Lewis EM, Sergeant S, Ledford B, Stull N, Dinauer MC, McPhail LC. Phosphorylation of p22phox on threonine 147 enhances NADPH oxidase activity by promoting p47phox binding. J Biol Chem. 2010;285:2959–67. 19948736 10.1074/jbc.M109.030643 PMC2823407

[484] Park JW. Attenuation of p47phox and p67phox membrane translocation as the inhibitory mechanism of S-nitrosothiol on the respiratory burst oxidase in human neutrophils. Biochem Biophys Res Commun. 1996;220:31–5. 8602852 10.1006/bbrc.1996.0351

[485] Palumbo S, Shin Y-J, Ahmad K, Desai AA, Quijada H, Mohamed M, et al. Dysregulated Nox4 ubiquitination contributes to redox imbalance and age-related severity of acute lung injury. Am J Physiol-Lung Cell Mol Physiol. 2017;312:L297–308. 28062482 10.1152/ajplung.00305.2016 PMC5374302

[486] Ohnuki J, Okazaki K-i. Integration of AlphaFold with molecular dynamics for efficient conformational sampling of transporter protein NarK. J Phys Chem B. 2024;128:7530–7. 39066727 10.1021/acs.jpcb.4c02726

[487] Varadi M, Anyango S, Deshpande M, Nair S, Natassia C, Yordanova G, et al. AlphaFold Protein Structure Database: massively expanding the structural coverage of protein-sequence space with high-accuracy models. Nucleic Acids Res. 2022;50:D439–44. 34791371 10.1093/nar/gkab1061 PMC8728224

[488] Newell AJ, Jima D, Reading B, Patisaul HB. Machine learning reveals common transcriptomic signatures across rat brain and placenta following developmental organophosphate ester exposure. Toxicol Sci. 2023;195:103–22. 37399109 10.1093/toxsci/kfad062 PMC10695431

[489] Yang Y, Dong M. Exploring the role of oxidative stress in carotid atherosclerosis: insights from transcriptomic data and single-cell sequencing combined with machine learning. Biol Direct. 2025;20:15. 39881407 10.1186/s13062-025-00600-7 PMC11780792

[490] Angermueller C, Pärnamaa T, Parts L, Stegle O. Deep learning for computational biology. Mol Syst Biol. 2016;12:MSB156651. 10.15252/msb.20156651 PMC4965871 27474269

[491] Libbrecht MW, Noble WS. Machine learning applications in genetics and genomics. Nat Rev Genet. 2015;16:321–32. 25948244 10.1038/nrg3920 PMC5204302

[492] Zilberter Y, Tabuena DR, Zilberter M. NOX-induced oxidative stress is a primary trigger of major neurodegenerative disorders. Prog Neurobiol. 2023;231:102539. 37838279 10.1016/j.pneurobio.2023.102539 PMC11758986

[493] Wu M, Xing Q, Duan H, Qin G, Sang N. Suppression of NADPH oxidase 4 inhibits PM(2.5)-induced cardiac fibrosis through ROS-P38 MAPK pathway. Sci Total Environ. 2022;837:155558. 35504386 10.1016/j.scitotenv.2022.155558

[494] Xie J, Li S, Ma X, Li R, Zhang H, Li J, et al. MiR-217-5p inhibits smog (PM2.5)-induced inflammation and oxidative stress response of mouse lung tissues and macrophages through targeting STAT1. Aging. 2022;14:6796–808. 36040387 10.18632/aging.204254 PMC9467388

[495] Hopkins AL. Network pharmacology: the next paradigm in drug discovery. Nat Chem Biol. 2008;4:682–90. 18936753 10.1038/nchembio.118

[496] Diebold BA, Bokoch GM. Molecular basis for Rac2 regulation of phagocyte NADPH oxidase. Nat Immunol. 2001;2:211–5. 11224519 10.1038/85259

[497] De Gassart A, Géminard C, Février B, Raposo G, Vidal M. Lipid raft-associated protein sorting in exosomes. Blood. 2003;102:4336–44. 12881314 10.1182/blood-2003-03-0871

[498] Skotland T, Sandvig K, Llorente A. Lipids in exosomes: current knowledge and the way forward. Prog Lipid Res. 2017;66:30–41. 28342835 10.1016/j.plipres.2017.03.001

[499] Théry C, Zitvogel L, Amigorena S. Exosomes: composition, biogenesis and function. Nat Rev Immunol. 2002;2:569–79. 12154376 10.1038/nri855

[500] Wu T, Du Y, Han J, Singh S, Xie C, Guo Y, et al. Urinary angiostatin-a novel putative marker of renal pathology chronicity in lupus nephritis. Mol Cell Proteom. 2013;12:1170–9. 10.1074/mcp.M112.021667 PMC3650329 23345539

[501] Yáñez-Mó M, Siljander PR, Andreu Z, Zavec AB, Borràs FE, Buzas EI, et al. Biological properties of extracellular vesicles and their physiological functions. J Extracell Vesicles. 2015;4:27066. 25979354 10.3402/jev.v4.27066 PMC4433489

[502] Castañé H, Baiges-Gaya G, Hernández-Aguilera A, Rodríguez-Tomàs E, Fernández-Arroyo S, Herrero P, et al. Coupling machine learning and lipidomics as a tool to investigate metabolic dysfunction-associated fatty liver disease. A general overview. Biomolecules. 2021;11:473. 10.3390/biom11030473 PMC8004861 33810079

[503] Zhang Z, Yu K, You Y, Jiang P, Wu Z, DeTure MA, et al. Comprehensive characterization of human brain-derived extracellular vesicles using multiple isolation methods: Implications for diagnostic and therapeutic applications. J Extracell Vesicles. 2023;12:12358. 37563857 10.1002/jev2.12358 PMC10415636

[504] Welsh JA, Goberdhan DCI, O’Driscoll L, Buzas EI, Blenkiron C, Bussolati B, et al. Minimal information for studies of extracellular vesicles (MISEV2023): from basic to advanced approaches. J Extracell Vesicles. 2024;13:e12404. 38326288 10.1002/jev2.12404 PMC10850029

[505] Skotland T, Ekroos K, Llorente A, Sandvig K. Quantitative lipid analysis of extracellular vesicle preparations: a perspective. J Extracell Vesicles. 2025;14:e70049. 40091364 10.1002/jev2.70049 PMC11911390

[506] Osteikoetxea X, Balogh A, Szabó-Taylor K, Németh A, Szabó TG, Pálóczi K, et al. Improved characterization of EV preparations based on protein to lipid ratio and lipid properties. PLoS ONE. 2015;10:e0121184. 25798862 10.1371/journal.pone.0121184 PMC4370721

[507] Simonsen JB. A liposome-based size calibration method for measuring microvesicles by flow cytometry. J Thromb Hemost. 2016;14:186–90. 10.1111/jth.13176 26509558

[508] Ostrowski M, Carmo NB, Krumeich S, Fanget I, Raposo G, Savina A, et al. Rab27a and Rab27b control different steps of the exosome secretion pathway. Nat Cell Biol. 2010;12:19–30. 19966785 10.1038/ncb2000

[509] Tan SS, Yin Y, Lee T, Lai RC, Yeo RWY, Zhang B, et al. Therapeutic MSC exosomes are derived from lipid raft microdomains in the plasma membrane. J Extracell Vesicles. 2013;2:22614. 10.3402/jev.v2i0.22614 PMC3873122 24371518

[510] Begum R, Mutyala D, Thota S, Bidarimath N, Batra S. Compartmentalization of proteasomes in lipid rafts and exosomes: unveiling molecular interactions in vaping-related cellular processes. Arch Toxicol. 2025;99:2493–505. 40289048 10.1007/s00204-025-03999-0

[511] Xu H, Ling M, Xue J, Dai X, Sun Q, Chen C, et al. Exosomal microRNA-21 derived from bronchial epithelial cells is involved in aberrant epithelium-fibroblast cross-talk in COPD induced by cigarette smoking. Theranostics. 2018;8:5419. 30555555 10.7150/thno.27876 PMC6276085

[512] Pergoli L, Cantone L, Favero C, Angelici L, Iodice S, Pinatel E, et al. Extracellular vesicle-packaged miRNA release after short-term exposure to particulate matter is associated with increased coagulation. Part Fiber Toxicol. 2017;14:32. 10.1186/s12989-017-0214-4 PMC5594543 28899404

[513] D’Amico G, Santonocito R, Vitale AM, Scalia F, Marino Gammazza A, Campanella C, et al. Air pollution: role of extracellular vesicles-derived noncoding RNAs in environmental stress response. Cells. 2023;12:1498. 37296619 10.3390/cells12111498 PMC10252408

[514] Fulzele S, Mendhe B, Khayrullin A, Johnson M, Kaiser H, Liu Y, et al. Muscle-derived miR-34a increases with age in circulating extracellular vesicles and induces senescence of bone marrow stem cells. Aging. 2019;11:1791. 30910993 10.18632/aging.101874 PMC6461183

[515] Weilner S, Schraml E, Wieser M, Messner P, Schneider K, Wassermann K, et al. Secreted microvesicular miR-31 inhibits osteogenic differentiation of mesenchymal stem cells. Aging Cell. 2016;15:744–54. 27146333 10.1111/acel.12484 PMC4933673

[516] Aoyama, Paik T, Watanabe YH, Laleu S, Gaggini B, Fioraso F, et al. Nicotinamide adenine dinucleotide phosphate oxidase in experimental liver fibrosis: GKT137831 as a novel potential therapeutic agent. Hepatology. 2012;56:2316–27. 22806357 10.1002/hep.25938 PMC3493679

[517] Jones D, Carbone M, Invernizzi P, Little N, Nevens F, Swain MG, et al. Impact of setanaxib on quality of life outcomes in primary biliary cholangitis in a phase 2 randomized controlled trial. Hepatol Commun. 2023;7:e0057. 36809195 10.1097/HC9.0000000000000057 PMC9949832

[518] Celano L, Carabio C, Da Silva YKC, Alexandre-Moreira MS, Lima LM, Cerecetto H, et al. Synthetic aromatic nitroalkenes as scavenger of reactive species and anti-inflammatory agents. Free Radic Biol Med. 2012;53:S119–S20.

[519] Hansen HS, Vana V. Non-endocannabinoid N-acylethanolamines and 2-monoacylglycerols in the intestine. Br J Pharmacol. 2019;176:1443–54. 29473944 10.1111/bph.14175 PMC6487557

[520] Yan Q, Gao K, Chi Y, Li K, Zhu Y, Wan Y, et al. NADPH oxidase-mediated upregulation of connexin43 contributes to podocyte injury. Free Radic Biol Med. 2012;53:1286–97. 22824863 10.1016/j.freeradbiomed.2012.07.012

[521] Regier DS, Greene DG, Sergeant S, Jesaitis AJ, McPhail LC. Phosphorylation of p22phox is mediated by phospholipase D-dependent and -independent mechanisms. Correlation of NADPH oxidase activity and p22phox phosphorylation. J Biol Chem. 2000;275:28406–12. 10.1074/jbc.M004703200 10893420

[522] Liu J. Activation de la NADPH oxydase phagocytaire (NOX2) dans les neutrophiles humains: étude de la phosphorylation de la p47phox lors de la phagocytose. Paris: Université Paris Cité; 2024.

[523] Lin M, Jin Y, Wang F, Meng Y, Huang J, Qin X, et al. MARCH9 mediates NOX2 ubiquitination to alleviate NLRP3 inflammasome-dependent pancreatic cell pyroptosis in acute pancreatitis. Pancreas. 2023;52:e62–9. 37378901 10.1097/MPA.0000000000002225

[524] Fang S, Cheng Y, Deng F, Zhang B. RNF34 ablation promotes cerebrovascular remodeling and hypertension by increasing NADPH-derived ROS generation. Neurobiol Dis. 2021;156:105396. 34015492 10.1016/j.nbd.2021.105396

[525] Yuan Z, Liu M, Zhang L, Jia L, Hao S, Su D, et al. Notch1 hyperactivity drives ubiquitination of NOX2 and dysfunction of CD8+ regulatory T cells in patients with systemic lupus erythematosus. Rheumatology. 2024;64:1500–12. 10.1093/rheumatology/keae231 38652598

[526] Qian J, Chen F, Kovalenkov Y, Pandey D, Moseley MA, Foster MW, et al. Nitric oxide reduces NADPH oxidase 5 (Nox5) activity by reversible S-nitrosylation. Free Radic Biol Med. 2012;52:1806–19. 22387196 10.1016/j.freeradbiomed.2012.02.029 PMC3464050

[527] Selemidis S, Dusting GJ, Peshavariya HM, Kemp-Harper BK, Drummond GR. Nitric oxide suppresses NADPH oxidase-dependent superoxide production by S-nitrosylation in human endothelial cells. Cardiovasc Res. 2007;75:349–58. 10.1016/j.cardiores.2007.03.030 17568572

[528] Xia N, Tenzer S, Lunov O, Karl M, Simmet T, Daiber A, et al. Regulation of NADPH oxidase-mediated superoxide production by acetylation and deacetylation. Front Physiol. 2021;12:693702. 34456745 10.3389/fphys.2021.693702 PMC8387964

[529] Ding B-Y, Xie C-N, Xie J-Y, Gao Z-W, Fei X-W, Hong E-H, et al. Knockdown of NADPH oxidase 4 reduces mitochondrial oxidative stress and neuronal pyroptosis following intracerebral hemorrhage. Neural Regen Res. 2023;18:1734. 36751799 10.4103/1673-5374.360249 PMC10154488

[530] Zeng C, Duan F, Hu J, Luo B, Huang B, Lou X, et al. NLRP3 inflammasome-mediated pyroptosis contributes to the pathogenesis of non-ischemic dilated cardiomyopathy. Redox Biol. 2020;34:101523. 10.1016/j.redox.2020.101523 PMC7327979 32273259

[531] Wang F, Liang Q, Ma Y, Sun M, Li T, Lin L, et al. Silica nanoparticles induce pyroptosis and cardiac hypertrophy via ROS/NLRP3/Caspase-1 pathway. Free Radic Biol Med. 2022;182:171–81. 35219847 10.1016/j.freeradbiomed.2022.02.027

[532] Li YS, Xia J, Chen CY, Ren SH, He MR. Upregulated dual oxidase 1-induced oxidative stress and caspase-1-dependent pyroptosis reflect the etiologies of heart failure. BMC Mol Cell Biol. 2024;25:16. 38750444 10.1186/s12860-024-00506-8 PMC11094974

[533] Liu S, Tao J, Duan F, Li H, Tan H. HHcy induces pyroptosis and atherosclerosis via the lipid raft-mediated NOX-ROS-NLRP3 inflammasome pathway in apoE−/− mice. Cells. 2022;11:2438. 35954287 10.3390/cells11152438 PMC9368640

[534] Eom JW, Lim JW, Kim H. Lutein induces reactive oxygen species-mediated apoptosis in gastric cancer AGS cells via NADPH oxidase activation. Molecules. 2023;28:1178. 36770846 10.3390/molecules28031178 PMC9919728

[535] Liu R, Duan T, Yu L, Tang Y, Liu S, Wang C, et al. Acid sphingomyelinase promotes diabetic cardiomyopathy via NADPH oxidase 4 mediated apoptosis. Cardiovasc Diabetol. 2023;22:25. 36732747 10.1186/s12933-023-01747-1 PMC9896821

[536] Tan L, Liu L, Yao J, Piao C. miR-145-5p attenuates inflammatory response and apoptosis in myocardial ischemia-reperfusion injury by inhibiting (NADPH) oxidase homolog 1. Exp Anim. 2021;70:311–21. 10.1538/expanim.20-0160 PMC8390312 33658472

[537] Han J, Jin C, Zhong Y, Zhu J, Liu Q, Sun D, et al. Involvement of NADPH oxidase in patulin-induced oxidative damage and cytotoxicity in HEK293 cells. Food Chem Toxicol. 2021;150:112055. 33577942 10.1016/j.fct.2021.112055

[538] Kim S, Lee H, Lim JW, Kim H. Astaxanthin induces NADPH oxidase activation and receptor-interacting protein kinase 1-mediated necroptosis in gastric cancer AGS cells. Mol Med Rep. 2021;24:837. 34608499 10.3892/mmr.2021.12477 PMC8503742

[539] Ladik M, Valenta H, Erard M, Vandenabeele P, Riquet FB. From TNF-induced signaling to NADPH oxidase enzyme activity: methods to investigate protein complexes involved in regulated cell death modalities. Front Cell Death. 2023;2:1127330.

[540] Yao W, Liao H, Pang M, Pan L, Guan Y, Huang X, et al. Inhibition of the NADPH oxidase pathway reduces ferroptosis during septic renal injury in diabetic mice. Oxid Med Cell Longev. 2022;2022:1193734. 35265258 10.1155/2022/1193734 PMC8898803

[541] Wu C, Xu D, Ge M, Luo J, Chen L, Chen Z, et al. Blocking glutathione regeneration: inorganic NADPH oxidase nanozyme catalyst potentiates tumoral ferroptosis. Nano Today. 2022;46:101574.

[542] Vlad M-L, Mares RG, Jakobsson G, Manea S-A, Lazar A-G, Preda MB, et al. Therapeutic S100A8/A9 inhibition reduces NADPH oxidase expression, reactive oxygen species production and NLRP3 inflammasome priming in the ischemic myocardium. Eur J Pharmacol. 2025;996:177575. 40180274 10.1016/j.ejphar.2025.177575

[543] Gordon R, Singh N, Lawana V, Ghosh A, Harischandra DS, Jin H, et al. Protein kinase Cδ upregulation in microglia drives neuroinflammatory responses and dopaminergic neurodegeneration in experimental models of Parkinson's disease. Neurobiology of disease. 2016;93:96–114. 10.1016/j.nbd.2016.04.008 PMC4995107 27151770

[544] Meyer C, Holtkamp C, Harm T, Grego E, Showman L, Rao NS, et al. Off-target effects of the NADPH oxidase inhibitor mitoapocynin-encapsulated nanoparticles and free-drug oral treatment in a rat DFP model of neurotoxicity. J Drug Target. 2025;33:1869–79. 10.1080/1061186X.2025.2523995 PMC12354291 40569814

[545] Gage M, Putra M, Wachter L, Dishman K, Gard M, Gomez-Estrada C, et al. Saracatinib, a Src tyrosine kinase inhibitor, as a disease modifier in the rat DFP model: sex differences, Neurobehavior, gliosis, neurodegeneration, and nitro-oxidative stress. Antioxidants. 2021;11:61. 35052568 10.3390/antiox11010061 PMC8773289

[546] Tian L, Tang P, Liu J, Liu Y, Hou L, Zhao J, et al. Microglial gp91phox-mediated neuroinflammation and ferroptosis contributes to learning and memory deficits in rotenone-treated mice. Free Radic Biol Med. 2024;220:56–66. 38697489 10.1016/j.freeradbiomed.2024.04.240

[547] Noce B, Marchese S, Massari M, Lambona C, Reis J, Fiorentino F, et al. Design of Benzyl-triazolopyrimidine-Based NADPH Oxidase Inhibitors Leads to the Discovery of a Potent Dual Covalent NOX2/MAOB Inhibitor. J Med Chem. 2025;68:6292–311. 10.1021/acs.jmedchem.4c02644 PMC11956017 40042998

[548] Waldeck-Weiermair M, Yadav S, Kaynert J, Thulabandu VR, Pandey AK, Spyropoulos F, et al. Differential endothelial hydrogen peroxide signaling via Nox isoforms: critical roles for Rac1 and modulation by statins. Redox Biol. 2022;58:102539. 36401888 10.1016/j.redox.2022.102539 PMC9673117

[549] Elksnis A, Cen J, Wikström P, Carlsson P-O, Welsh N. Pharmacological inhibition of NOX4 improves mitochondrial function and survival in human beta-cells. Biomedicines. 2021;9:1865. 34944680 10.3390/biomedicines9121865 PMC8698703

[550] Schiffer TA, Carvalho LRRA, Guimaraes D, Boeder A, Wikström P, Carlström M. Specific NOX4 inhibition preserves mitochondrial function and dampens kidney dysfunction following ischemia–reperfusion-induced kidney injury. Antioxidants. 2024;13:489. 38671936 10.3390/antiox13040489 PMC11047485

[551] Tang P, Sheng J, Peng X, Zhang R, Xu T, Hu J, et al. Targeting NOX4 disrupts the resistance of papillary thyroid carcinoma to chemotherapeutic drugs and lenvatinib. Cell Death Discov. 2022;8:177. 35396551 10.1038/s41420-022-00994-7 PMC8990679

[552] Li J, Wang L, Wang B, Zhang Z, Jiang L, Qin Z, et al. NOX4 is a potential therapeutic target in septic acute kidney injury by inhibiting mitochondrial dysfunction and inflammation. Theranostics. 2023;13:2863. 37284448 10.7150/thno.81240 PMC10240817

[553] El Khoury M, Biondi O, Bruneteau G, Sapaly D, Bendris S, Bezier C, et al. NADPH oxidase 4 inhibition is a complementary therapeutic strategy for spinal muscular atrophy. Front Cell Neurosci. 2023;17:1242828. 37780204 10.3389/fncel.2023.1242828 PMC10536974

[554] Mu W, Shi Y-g, Jian Y-l, Li L, Zhou Y-f, Wang H, et al. NOX1 inhibition sensitizes HCC cells to sorafenib and radiotherapy by modulating ROS-mediated programmed cell death. Acta Pharmacol Sin. 2026;47:434–43. 10.1038/s41401-025-01623-6 PMC12811247 40775534

[555] Kim G-Y, Kim S, Park K, Lim H-J, Kim W-H. Gasoline exhaust particles induce MMP1 expression via Nox4-derived ROS-ATF3-linked pathway in human umbilical vein endothelial cells. Toxicology. 2025;511:154051. 39793954 10.1016/j.tox.2025.154051

[556] Lee AR, Jeong M, Koo K, Kim SJ, Pyo MJ, Hong Y, et al. Progranulin derivative attenuates lung neutrophilic infiltration from diesel exhaust particle exposure. Allergy. 2025;80:703–14. 39462225 10.1111/all.16362 PMC11891434

[557] Kim JY, Lee EY, Kim JH, Seo EJ, Eom SY, Seo JH. Diesel exhaust particles disrupt blood‒retina barrier integrity via TLR2 and TLR4 activation. BMB Rep. 2025;58:300–6. 40495488 10.5483/BMBRep.2025-0013 PMC12313399

[558] Su AL, Marques CF, Krzeminski J, El-Bayoumy K, Penning TM. Role of human Aldo-Keto reductases and nuclear factor erythroid 2-related factor 2 in the metabolic activation of 1,8-dinitropyrene and its metabolite 1-amino-8-nitropyrene via nitroreduction in human lung cells. Chem Res Toxicol. 2025;38:1227–38. 40633532 10.1021/acs.chemrestox.5c00101 PMC12558370

[559] Kim JY, Kim A, Kim J-H, Gil Y-C, Kim Y-D, Shin D-I, et al. Ferroptosis in the substantia nigra pars compacta of mice: triggering role of ultrafine diesel exhaust particles and mitigation by α-lipoic acid. Neurochem Res. 2025;50:37. 10.1007/s11064-024-04278-7 39601947

[560] Hu T, Zhu P, Liu Y, Zhu H, Geng J, Wang B, et al. PM2. 5 induces endothelial dysfunction by activating NLRP3 inflammasome. Environ Toxicol. 2021;36:1886–93. 34173703 10.1002/tox.23309

[561] Jung I, Park M, Jeong M-H, Park K, Kim W-H, Kim G-Y. Transcriptional analysis of gasoline engine exhaust particulate matter 2.5-exposed human umbilical vein endothelial cells reveals the different gene expression patterns related to the cardiovascular diseases. Biochem Biophys Rep. 2022;29:101190. 34988296 10.1016/j.bbrep.2021.101190 PMC8695280

[562] Xu X, Xu H, Liu S, Wang H, Hu M, Song L. MAPK/AP-1 pathway activation mediates AT1R upregulation and vascular endothelial cells dysfunction under PM2. 5 exposure. Ecotoxicol Environ Saf. 2019;170:188–94. 30529618 10.1016/j.ecoenv.2018.11.124

[563] Pasaribu SM, Purba A, Perangin-angin G, Siahaan JM. Literature review: Relationship between rubber factory pollution exposure and reactive oxygen species (ROS) levels in workers. Bul Farmatera. 2025;10:230–8.

[564] Watabe Y, Chuang VTG, Sakai H, Ito C, Enoki Y, Kohno M, et al. Carbon monoxide alleviates endotoxin-induced acute lung injury via NADPH oxidase inhibition in macrophages and neutrophils. Biochem Pharmacol. 2025;233:116782. 39880317 10.1016/j.bcp.2025.116782

[565] Yang T, Peleli M, Zollbrecht C, Giulietti A, Terrando N, Lundberg JO, et al. Inorganic nitrite attenuates NADPH oxidase-derived superoxide generation in activated macrophages via a nitric oxide-dependent mechanism. Free Radic Biol Med. 2015;83:159–66. 25724690 10.1016/j.freeradbiomed.2015.02.016

[566] Zhang W, Hu X, Cheng W, Zhang L, Chen Y, Fu Q, et al. Harnessing nanomaterials to precisely regulate the immunosuppressive tumor microenvironment for enhanced immunotherapy. BMEMat. 2026;4:e70019.

[567] Ge X, Yin Y, Wang X, Li X, Ouyang J, Na N. Mimicking NADPH oxidase and lipoxygenase by biodegradable single-site catalyst via cascade reaction to trigger tumor-specific ferroptosis. Chem Sci. 2025;16:14668–80. 10.1039/d5sc02512e PMC12260917 40671752

[568] Zhang R, Luan T. Combined molecular toxicity mechanism of persistent organic pollutant mixtures. In: Toxicological Assessment of Combined Chemicals in the Environment. Hoboken (NJ): John Wiley & Sons, Inc.;2025. p. 183–94.

[569] Ahmad MB, Islam AU, Hassan S, Tarfeen N, Nisa KU, Nisar K, et al. Unraveling the toxic link between pesticides and brain cancer: a review on molecular mechanisms, signaling pathways and future research trends. Nucleus. 2026;69:189–204.

[570] Kafshgiri SK, Attaranzadeh A, Safarpour H, Ghasemi F, Miri-Moghaddam E. Effects of alpha-lipoic acid on glyphosate-treated human granulosa cells: a cross-sectional study. Health Sci Rep. 2025;8:e70946. 40567935 10.1002/hsr2.70946 PMC12188277

[571] Kassan M, Galán M, Partyka M, Saifudeen Z, Henrion D, Trebak M, et al. Endoplasmic reticulum stress is involved in cardiac damage and vascular endothelial dysfunction in hypertensive mice. Arterioscler Thromb Vasc Biol. 2012;32:1652–61. 22539597 10.1161/ATVBAHA.112.249318 PMC5374512

[572] Huang J, Canadien V, Lam GY, Steinberg BE, Dinauer MC, Magalhaes MA, et al. Activation of antibacterial autophagy by NADPH oxidases. Proc Natl Acad Sci USA. 2009;106:6226–31. 19339495 10.1073/pnas.0811045106 PMC2664152

[573] Zhu X, Shen K, Bai Y, Zhang A, Xia Z, Chao J, et al. NADPH oxidase activation is required for pentylenetetrazole kindling-induced hippocampal autophagy. Free Radic Biol Med. 2016;94:230–42. 26969791 10.1016/j.freeradbiomed.2016.03.004

[574] Yang C-S, Lee J-S, Rodgers M, Min C-K, Lee J-Y, Kim HJ, et al. Autophagy protein Rubicon mediates phagocytic NADPH oxidase activation in response to microbial infection or TLR stimulation. Cell Host Microbe. 2012;11:264–76. 22423966 10.1016/j.chom.2012.01.018 PMC3616771

[575] Teng R-J, Du J, Welak S, Guan T, Eis A, Shi Y, et al. Cross talk between NADPH oxidase and autophagy in pulmonary artery endothelial cells with intrauterine persistent pulmonary hypertension. Am J Physiol Lung Cell Mol Physiol. 2012;302:L651–63. 22245997 10.1152/ajplung.00177.2011 PMC3330765

[576] Sun T, Chen Q, Zhu SY, Wu Q, Liao CR, Wang Z, et al. Hydroxytyrosol promotes autophagy by regulating SIRT1 against advanced oxidation protein product‑induced NADPH oxidase and inflammatory response. Int J Mol Med. 2019;44:1531–40. 31432093 10.3892/ijmm.2019.4300

[577] Sanders YY, Liu H, Liu G, Thannickal VJ. Epigenetic mechanisms regulate NADPH oxidase-4 expression in cellular senescence. Free Radical Biology and Medicine. 2015;79:197–205. 10.1016/j.freeradbiomed.2014.12.008 25526894

[578] Li W, Kou J, Qin J, Li L, Zhang Z, Pan Y, et al. NADPH levels affect cellular epigenetic state by inhibiting HDAC3–Ncor complex. Nat Metab. 2021;3:75–89. 33462516 10.1038/s42255-020-00330-2

[579] Chen X, Mutzel P, Hierdeis K, Masgrau-Alsina S, Sperandio M, Trefz J, et al. Crosstalk between NADPH oxidases and the Hippo pathway coactivator Yes-Associated Protein promotes the cardiac response to hypoxia. Eur Heart J. 2022;43:ehac544.2874.

[580] Wu W, Ziemann M, Huynh K, She G, Pang Z-D, Zhang Y, et al. Activation of Hippo signaling pathway mediates mitochondria dysfunction and dilated cardiomyopathy in mice. Theranostics. 2021;11:8993. 34522223 10.7150/thno.62302 PMC8419046

[581] Wu W, Lu Q, Ma S, Du J-C, Huynh K, Duong T, et al. Mitochondrial damage in a Takotsubo syndrome-like mouse model mediated by activation of β-adrenoceptor-Hippo signaling pathway. Am J Physiol Heart Circ Physiol. 2023;324:H528–41. 36867446 10.1152/ajpheart.00459.2022

[582] Ren Y, Zhao X. Bone marrow mesenchymal stem cells-derived exosomal lncRNA GAS5 mitigates heart failure by inhibiting UL3/Hippo pathway-mediated ferroptosis. Eur J Med Res. 2024;29:303. 38812041 10.1186/s40001-024-01880-x PMC11137962

[583] Ding M, Zhu Y, Xu X, He H, Jiang T, Mo X, et al. Naringenin inhibits acid sphingomyelinase-mediated membrane raft clustering to reduce NADPH oxidase activation and vascular inflammation. J Agric Food Chem. 2024;72:7130–9. 38516841 10.1021/acs.jafc.3c07874

[584] Giniatullin AR, Mukhutdinova KA, Petrov AM. Mechanism of purinergic regulation of neurotransmission in mouse neuromuscular junction: the role of redox signaling and lipid rafts. Neurochem Res. 2024;49:2021–37. 38814360 10.1007/s11064-024-04153-5

[585] Bujko K, Adamiak M, Konopko A, Chumak V, Ratajczak J, Brzezniakiewicz-Janus K, et al. Defect in migration of HSPCs in Nox-2 deficient mice explained by impaired activation of Nlrp3 inflammasome and impaired formation of membrane lipid rafts. Stem Cell Rev Rep. 2025;21:45–58. 39134888 10.1007/s12015-024-10775-7 PMC11762604

[586] Curtis WM, Seeds WA, Mattson MP, Bradshaw PC. NADPH and mitochondrial quality control as targets for a circadian-based fasting and exercise therapy for the treatment of Parkinson’s disease. Cells. 2022;11:2416. 35954260 10.3390/cells11152416 PMC9367803

[587] Sengupta S, Lee Y, Tao JQ, Akolia I, Louneva N, Forrest K, et al. Circadian control of pulmonary endothelial signaling occurs via the NADPH oxidase 2-NLRP3 pathway. J Biol rhythms. 2025;40:555–73. 40947518 10.1177/07487304251363656 PMC12499374

[588] Guo Y, Wang J, Zhang D, Tang Y, Cheng Q, Li J, et al. Diabetes-associated sleep fragmentation impairs liver and heart function via SIRT1-dependent epigenetic modulation of NADPH oxidase 4. Acta Pharm Sin B. 2025;15:1480–96. 40370565 10.1016/j.apsb.2024.12.031 PMC12069238

