# Supplementary material for: NADPH oxidases in immunometabolism and disease pathology: mechanistic networks, pollutant triggers, and therapeutic frontiers
Source: Cell Mol Immunol. 2026 Aug 24;23(9):1055–109. doi: 10.1038/s41423-026-01447-2 (PMC13527155; doi:10.1038/s41423-026-01447-2)
Supplement: Supplementary file 1 [file 41423_2026_1447_MOESM1_ESM.docx]

**Supplementary Table 01: List of abbreviations**

| **Abbreviation** | **Full Form** |
| --- | --- |
| **4-HNE** | 4-Hydroxynonenal |
| **AD** | Alzheimer's Disease |
| **AgNP** | Silver Nanoparticle |
| **AhR** | Aryl Hydrocarbon Receptor |
| **AKI** | Acute Kidney Injury |
| **ALD** | Alcohol-Associated Liver Disease |
| **ALI** | Acute Lung Injury |
| **ALS** | Amyotrophic Lateral Sclerosis |
| **AML** | Acute Myeloid Leukemia |
| **AMPK** | AMP-Activated Protein Kinase |
| **Ang II** | Angiotensin II |
| **ANGPTL4** | Angiopoietin-Like 4 |
| **AP-1** | Activator Protein 1 |
| **APX-115** | 3-Phenyl-1-(pyridin-2-yl)-4-propyl-1,5-hydroxypyrazol HCl (pan-NOX inhibitor) |
| **APP** | Amyloid Precursor Protein |
| **ApoE** | Apolipoprotein E |
| **ARDS** | Acute Respiratory Distress Syndrome |
| **BAL** | Bronchoalveolar Lavage |
| **BBB** | Blood-Brain Barrier |
| **BDL** | Bile Duct Ligation |
| **BDNF** | Brain-Derived Neurotrophic Factor |
| **BEAS-2B** | Human Bronchial Epithelial Cell Line |
| **BIRC5** | Baculoviral IAP Repeat Containing 5 (Survivin) |
| **CAF** | Cancer-Associated Fibroblast |
| **CGD** | Chronic Granulomatous Disease |
| **CHOP** | CCAAT/Enhancer-Binding Protein Homologous Protein |
| **CKD** | Chronic Kidney Disease |
| **CNS** | Central Nervous System |
| **COPD** | Chronic Obstructive Pulmonary Disease |
| **COX-2** | Cyclooxygenase-2 |
| **CR3** | Complement Receptor 3 |
| **CS** | Cigarette Smoke |
| **CSC** | Colonic Stem Cell |
| **CSE** | Cigarette Smoke Extract |
| **CSF** | Cerebrospinal Fluid |
| **CYBA** | Cytochrome b-245 Alpha Chain (encodes p22phox) |
| **CYBB** | Cytochrome b-245 Beta Chain (encodes NOX2/gp91phox) |
| **CYBC1** | Cytochrome b-245 Chaperone 1 (encodes EROS) |
| **DEN** | Diethylnitrosamine |
| **DHE** | Dihydroethidium |
| **DLBCL** | Diffuse Large B-Cell Lymphoma |
| **DN** | Diabetic Nephropathy |
| **DOX** | Doxorubicin |
| **DPI** | Diphenyleneiodonium |
| **DUOX1** | Dual Oxidase 1 |
| **DUOX2** | Dual Oxidase 2 |
| **DUOXA1** | Dual Oxidase Maturation Factor 1 |
| **DUOXA2** | Dual Oxidase Maturation Factor 2 |
| **EAE** | Experimental Autoimmune Encephalomyelitis |
| **ECM** | Extracellular Matrix |
| **EGFR** | Epidermal Growth Factor Receptor |
| **EMT** | Epithelial-Mesenchymal Transition |
| **eNOS** | Endothelial Nitric Oxide Synthase |
| **ER** | Endoplasmic Reticulum |
| **EROS** | Essential for Reactive Oxygen Species (CYBC1) |
| **ERK** | Extracellular Signal-Regulated Kinase |
| **ESR** | Electron Spin Resonance |
| **EV** | Extracellular Vesicle |
| **EZH2** | Enhancer of Zeste Homolog 2 |
| **FAD** | Flavin Adenine Dinucleotide |
| **FFAR2/3** | Free Fatty Acid Receptor 2/3 |
| **G3BP1** | G3BP Stress Granule Assembly Factor 1 |
| **GDF6** | Growth Differentiation Factor 6 |
| **GI** | Gypensapogenin I |
| **GKT136901** | Dual NOX1/NOX4 Inhibitor (Research Compound) |
| **GKT137831** | Setanaxib — Dual NOX1/NOX4 Inhibitor |
| **GPX3** | Glutathione Peroxidase 3 |
| **gp91ds-tat** | NOX2-Derived Inhibitory Peptide Conjugated to TAT Cell-Penetrating Sequence |
| **GPI** | Glycosylphosphatidylinositol |
| **GRK2** | G-Protein Coupled Receptor Kinase 2 |
| **GRP78** | Glucose-Regulated Protein 78 (BiP) |
| **GSH** | Glutathione |
| **GW182** | TNRC6A — P-body Component |
| **H3K27me3** | Trimethylation of Histone H3 at Lysine 27 |
| **HBV** | Hepatitis B Virus |
| **HCB** | Hexachlorobenzene |
| **HCC** | Hepatocellular Carcinoma |
| **HCV** | Hepatitis C Virus |
| **HF** | Heart Failure |
| **HIF-1α** | Hypoxia-Inducible Factor 1-Alpha |
| **HIF-2α** | Hypoxia-Inducible Factor 2-Alpha |
| **hiPSC** | Human Induced Pluripotent Stem Cell |
| **HK-2** | Human Proximal Tubular Cell Line (Human Kidney-2) |
| **HMGB1** | High Mobility Group Box 1 |
| **HO-1** | Heme Oxygenase-1 |
| **HSC** | Hepatic Stellate Cell |
| **HSPCs** | Hematopoietic Stem and Progenitor Cells |
| **HUVECs** | Human Umbilical Vein Endothelial Cells |
| **I/R** | Ischemia/Reperfusion |
| **ILC2** | Group 2 Innate Lymphoid Cell |
| **iNOS** | Inducible Nitric Oxide Synthase |
| **IPF** | Idiopathic Pulmonary Fibrosis |
| **iPSC** | Induced Pluripotent Stem Cell |
| **ITC** | Isothermal Titration Calorimetry |
| **JNK** | c-Jun N-Terminal Kinase |
| **KC** | Keratinocyte Chemoattractant (Murine Homolog of IL-8/CXCL8) |
| **KO** | Knockout |
| **LATS1/2** | Large Tumor Suppressor Kinase 1/2 |
| **LDH** | Lactate Dehydrogenase |
| **LDL** | Low-Density Lipoprotein |
| **LPS** | Lipopolysaccharide |
| **LTP** | Long-Term Potentiation |
| **M1** | Classically Activated (Pro-inflammatory) Macrophage Phenotype |
| **M2** | Alternatively Activated (Anti-inflammatory) Macrophage Phenotype |
| **MAM** | Mitochondria-Associated Endoplasmic Reticulum Membrane |
| **MAPK** | Mitogen-Activated Protein Kinase |
| **MDA** | Malondialdehyde |
| **MD** | Molecular Dynamics |
| **MISEV** | Minimal Information for Studies of Extracellular Vesicles |
| **MLE-12** | Murine Lung Epithelial Cell Line (Type II Pneumocytes) |
| **ML171** | 2-Acetylphenothiazine (NOX1-selective Inhibitor) |
| **MM-PBSA/MM-GBSA** | Molecular Mechanics Poisson-Boltzmann/Generalized Born Surface Area |
| **MPO** | Myeloperoxidase |
| **MSC** | Mesenchymal Stem Cell |
| **MSC-EV** | Mesenchymal Stem Cell-Derived Extracellular Vesicle |
| **MST1/2** | Mammalian Sterile 20-Like Kinase 1/2 |
| **mtDNA** | Mitochondrial DNA |
| **mtROS** | Mitochondrial Reactive Oxygen Species |
| **MVB** | Multivesicular Body |
| **NAC** | N-Acetylcysteine |
| **NADPH** | Nicotinamide Adenine Dinucleotide Phosphate |
| **NAFLD** | Non-Alcoholic Fatty Liver Disease |
| **NASH** | Non-Alcoholic Steatohepatitis |
| **NCF1** | Neutrophil Cytosol Factor 1 (p47phox) |
| **NCF2** | Neutrophil Cytosol Factor 2 (p67phox) |
| **NCF4** | Neutrophil Cytosol Factor 4 (p40phox) |
| **NF-κB** | Nuclear Factor Kappa-Light-Chain-Enhancer of Activated B Cells |
| **NLRP3** | NOD-Like Receptor Protein 3 |
| **NOD** | Nucleotide-Binding Oligomerization Domain |
| **NOX** | NADPH Oxidase |
| **NOX1** | NADPH Oxidase 1 |
| **NOX2** | NADPH Oxidase 2 (gp91phox) |
| **NOX3** | NADPH Oxidase 3 |
| **NOX4** | NADPH Oxidase 4 |
| **NOX5** | NADPH Oxidase 5 |
| **NOXA1** | NOX Activator 1 |
| **NOXO1** | NOX Organizer 1 |
| **NP** | Nucleus Pulposus |
| **Nrf2** | Nuclear Factor Erythroid 2-Related Factor 2 |
| **NSCLC** | Non-Small Cell Lung Cancer |
| **NTA** | Nanoparticle Tracking Analysis |
| **O₂⁻** | Superoxide Anion |
| **OPN** | Osteopontin |
| **OPE** | Organophosphate Ester |
| **OXPHOS** | Oxidative Phosphorylation |
| **p22phox** | 22 kDa Phagocyte Oxidase Membrane Subunit (CYBA) |
| **p40phox** | 40 kDa Phagocyte Oxidase Cytosolic Subunit (NCF4) |
| **p47phox** | 47 kDa Phagocyte Oxidase Cytosolic Subunit (NCF1) |
| **p67phox** | 67 kDa Phagocyte Oxidase Cytosolic Subunit (NCF2) |
| **PANoptosis** | Integrated Inflammatory Programmed Cell Death Combining Pyroptosis, Apoptosis, and Necroptosis |
| **PAMP** | Pathogen-Associated Molecular Pattern |
| **PBC** | Primary Biliary Cholangitis |
| **PCP** | Pentachlorophenol |
| **PCNA** | Proliferating Cell Nuclear Antigen |
| **PDAC** | Pancreatic Ductal Adenocarcinoma |
| **PD** | Parkinson's Disease |
| **PD-L1** | Programmed Death-Ligand 1 |
| **PFAS** | Per- and Polyfluoroalkyl Substances |
| **PFOA** | Perfluorooctanoic Acid |
| **PFOS** | Perfluorooctane Sulfonate |
| **PK** | Pharmacokinetics |
| **PM₂.₅** | Fine Particulate Matter (≤2.5 μm Aerodynamic Diameter) |
| **POP** | Persistent Organic Pollutant |
| **PPI** | Protein-Protein Interaction |
| **PPP** | Pentose Phosphate Pathway |
| **PRC2** | Polycomb Repressive Complex 2 |
| **PRDX6** | Peroxiredoxin 6 |
| **PRR** | Pattern Recognition Receptor |
| **PTEN** | Phosphatase and Tensin Homolog |
| **PTM** | Post-Translational Modification |
| **PX** | Phox Homology (Domain) |
| **RAC1/2** | Ras-Related C3 Botulinum Toxin Substrate 1/2 |
| **ROCK2** | Rho-Associated Coiled-Coil Kinase 2 |
| **RONS** | Reactive Oxygen and Nitrogen Species |
| **ROS** | Reactive Oxygen Species |
| **Rpl13a** | Ribosomal Protein L13a |
| **SCFA** | Short-Chain Fatty Acid |
| **SERCA2a** | Sarcoplasmic/Endoplasmic Reticulum Calcium ATPase 2a |
| **SHR** | Spontaneously Hypertensive Rat |
| **sNOX2-dp** | Soluble NOX2-Derived Peptide (Serum Biomarker) |
| **snoRNA** | Small Nucleolar RNA |
| **SOD** | Superoxide Dismutase |
| **SOD1** | Superoxide Dismutase 1 |
| **SOD2** | Superoxide Dismutase 2 (Manganese SOD) |
| **SPARC** | Secreted Protein Acidic and Rich in Cysteine |
| **SPR** | Surface Plasmon Resonance |
| **SS** | Dahl Salt-Sensitive (Rat) |
| **SSCD247⁻/⁻** | T Cell-Deficient Dahl SS Rat (CD247 Gene Deleted) |
| **SSp67phox⁻/⁻** | NOX2-Deficient Dahl SS Rat (p67phox Subunit Deleted) |
| **STZ** | Streptozotocin |
| **TAM** | Tumor-Associated Macrophage |
| **TAZ** | Transcriptional Co-activator with PDZ-Binding Motif (WWTR1) |
| **TCGA** | The Cancer Genome Atlas |
| **TCHQ** | Tetrachlorohydroquinone |
| **TDP-43** | TAR DNA-Binding Protein 43 (TARDBP) |
| **TG15-132** | Novel Brain-Permeable Selective NOX2 Inhibitor |
| **TGF-β** | Transforming Growth Factor Beta |
| **TLR** | Toll-Like Receptor |
| **TMAO** | Trimethylamine N-Oxide |
| **TME** | Tumor Microenvironment |
| **TUNEL** | Terminal Deoxynucleotidyl Transferase dUTP Nick End Labeling |
| **TXNIP** | Thioredoxin-Interacting Protein |
| **UUO** | Unilateral Ureteral Obstruction |
| **VAS2870** | Pan-NOX Inhibitor (Tool Compound) |
| **VAS3947** | Pan-NOX Inhibitor Analogue of VAS2870 |
| **VEGF** | Vascular Endothelial Growth Factor |
| **XO** | Xanthine Oxidase |
| **YAP** | Yes-Associated Protein |
| **YAP/TAZ** | Yes-Associated Protein/Transcriptional Co-activator with PDZ-Binding Motif |
